# Qualitative dyadic analysis in care partnership research: a scoping review

**DOI:** 10.1186/s12874-025-02722-y

**Published:** 2025-12-11

**Authors:** Andrea S. E. Parks, Lesley Gotlib Conn, Bahar Aria, Manisha Reza Paul, Allan Li, Agessandro Abrahao, Lorne Zinman, Joanna E. M. Sale

**Affiliations:** 1 Institute of Health Policy, Management and Evaluation, Dalla Lana School of Public Health, University of Toronto, 4th Floor – 155 College Street, Toronto, ON M5T 3M6 Canada; 2 Sunnybrook Research Institute, 2075 Bayview Avenue,, Toronto, ON M4N 3M5 Canada; 3 Musculoskeletal Health and Outcomes Research, Li Ka Shing Knowledge Institute, St. Michael’s Hospital, Unity Health Toronto, 30 Bond Street, Toronto, ON M5B 1W8 Canada; 4 Sunnybrook Health Sciences Centre, Neurology Division, 2075 Bayview Avenue,, Toronto, ON M4N 3M5 Canada; 5 Division of Neurology, Department of Medicine, Temerty Faculty of Medicine, University of Toronto, C. David Naylor Building, 6 Queen’s Park Crescent West, 3rd floor, Toronto, ON M5S 3H2 Canada; 6 Department of Surgery, Faculty of Medicine, University of Toronto, 5th Floor – 149 College Street, Toronto, ON M5B 1W8 Canada

**Keywords:** Dyadic analysis, Care partnerships, Qualitative health research, Dyadic methods, Research methodology

## Abstract

**Background:**

Chronic illness impacts not only individuals affected by it, but also those who care for them. Care partnerships recognize that health conditions are often shared, dyadic experiences. Qualitative dyadic analysis, which foregrounds the dyad as the unit of analysis, is a method that can enhance understanding of illness as a joint experience. However, when perspectives of dyad members are collected separately, their subsequent analysis as a unit can be challenging.

**Objective:**

To review and summarize qualitative literature where data have been collected through separate individual interviews with patient and care partner dyads and analyzed at the dyadic level.

**Methods:**

A scoping review guided by Joanna Briggs Institute methodology was undertaken. Databases (Ovid’s Medline, Embase, and PsycINFO; EBSCO CINAHL; and ProQuest Sociological Abstracts) were searched in February 2024. Eligible articles included peer-reviewed literature published in English from 2010 onwards documenting qualitative dyadic analysis of individual interviews collected from patient and care partner dyads. Title and abstracts were screened and the full text of all potentially eligible articles was reviewed by two independent reviewers. Data were extracted using a table and results were summarized using frequency counts and qualitative content analysis.

**Results:**

7,494 records were identified and screened. 113 reports of 112 unique studies fulfilled eligibility criteria and were included. Numerous methodologies and analytic methods were reported, many of which incorporated methods from different qualitative traditions, often with variable sequencing of analytic steps that were infrequently well described. Studies were not routinely conceptualized at the dyadic level and underlying epistemological assumptions were rarely discussed despite their essential role in grounding dyadic analysis.

**Conclusions:**

When conducting qualitative dyadic analysis, researchers should consider dyadic study conceptualization from study outset. The purpose of the analysis, the analytic steps taken, and their alignment with underlying epistemology and other incorporated methodologies should be clearly documented and reported.

**Supplementary Information:**

The online version contains supplementary material available at 10.1186/s12874-025-02722-y.

## Background

There is growing awareness that chronic illness not only impacts the individual affected by it, but also those who provide care and support to these individuals [1]. Care partnerships frame illness as a dyadic experience, recognizing the interdependent relationship between the person with a health condition and their care partner [2]. Often family members or close friends, care partners provide unpaid care and emotional, physical, or cognitive support to an individual with a health condition in the context of a close personal, rather than professional, relationship [3]. Each individual’s unique experience, including their perceptions of their partner, impacts their relationship and shared experience of illness [4]. Despite increasing recognition that illness is a joint experience shared by patients and their care partners, most illness experience research focuses on individuals with the illness or their care partners in isolation [1, 5].

Dyadic methodologies embrace the interdependent relationship between individuals as a source of information rather than trying to control for it [6] and foreground the dyadic relationship throughout study design and analysis [7]. Qualitative analysis at the individual level treats each participant as the study unit, with data collected from each individual analyzed to understand their unique experience, which is then compared to other study participants, to provide a broad, in-depth understanding of the phenomenon under investigation [8]. In contrast, qualitative analysis at the dyadic level means the unit of analysis is the dyad- two individuals who share a pre-existing relationship with each other [9]- and the experiences of both individuals of the dyadic unit are analyzed together, synthesizing their accounts, to understand their shared experience [7]. When individuals constitute the unit of analysis, the understanding of a phenomena that involves two sides is limited, whereas the synthesis of two accounts of a shared experience provides a richer understanding through the additional perspective of the dyad [7]. Qualitative dyadic analysis therefore offers a strategy to enhance understanding of illness as a joint experience and of care partnership dynamics.

**Fig. 1:**
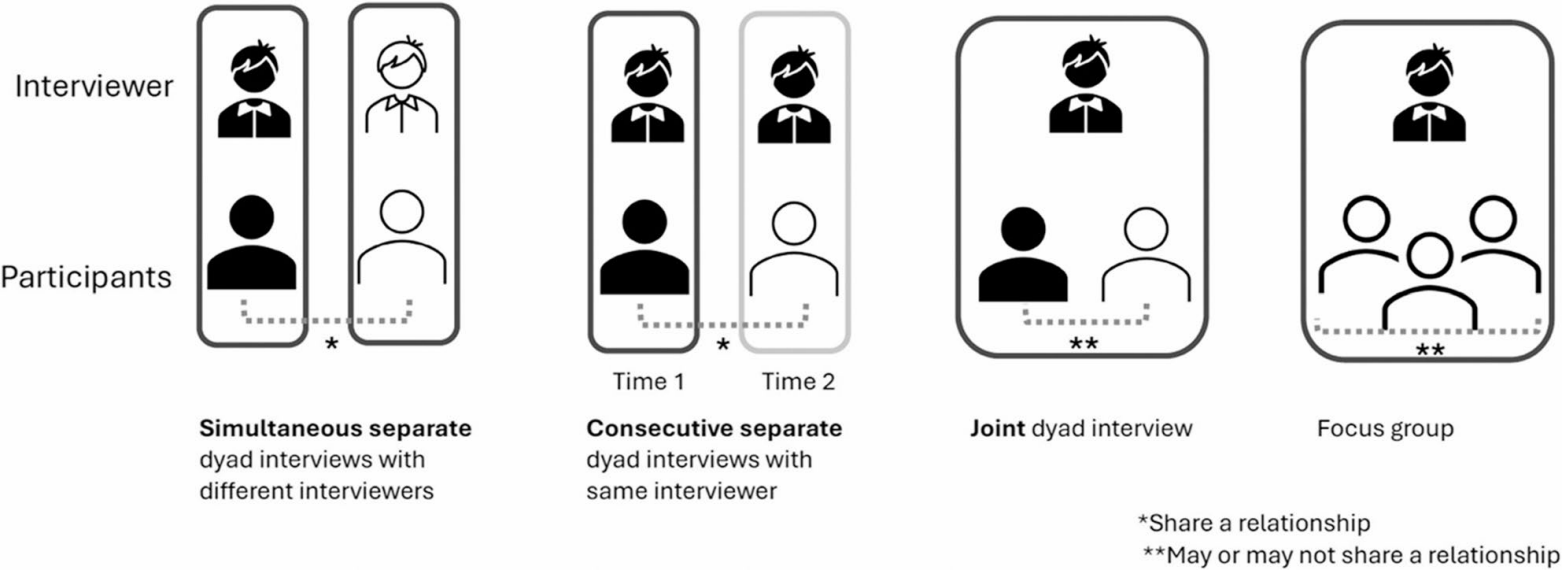
Types of interview formats

There are numerous publications within the qualitative health literature addressing data collection from dyads including potential ethical issues associated with studying individuals who share a relationship (for example [6, 10–12]). Separate interviews allow each participant to express their own views without having to consider the reaction of the other [11]. Their subsequent analysis as a unit facilitates insight into their joint lived experience beyond what can be discerned from each individual [7]. Joint interviews, by contrast, involve interviewing both dyad members together to explore how the pair jointly construct a narrative about the same event or phenomenon [11, 13]. Joint interviews exist on a continuum between individual interviews and focus groups and typically, though not always, involve two individuals with a pre-existing relationship [11, 14] (Fig. 1). Either approach can be employed in qualitative dyadic research, with the choice between them guided by the study’s objectives. Joint interviews may be preferrable when the aim is to analyze interactions between dyad members, whereas separate interviews may be more suitable for exploring sensitive topics that participants might be reluctant to discuss in each other’s presence [7, 11].

When perspectives of dyad members are collected separately, there are inherent challenges to analyzing them together [15], particularly in handling divergent and convergent data within and between accounts [16–18]. The researcher is in a unique position to make comparisons and observations across accounts [16] and must decide how to integrate similarities, inconsistencies, and contradictions, while remaining consistent with their chosen epistemological framework [18, 19]. Furthermore, it is important to consider whether the analysis will remain descriptive, or be extended to an interpretive level, and whether this aligns with the chosen data analysis approach, particularly when considering how dissonant data within a dyad will be analyzed [17].

Despite the added complexity of analyzing multiple perspectives, novel findings about relational dynamics, decision making, and how contradiction and agreement shape relationships may emerge [7, 8, 15, 20]. Such an approach offers a unique methodology to capture the multidimensional nature of experiences of health and illness. However, the analytic possibilities of interviews of individuals who are in direct relationships with each other are rarely fully exploited [16]. For qualitative studies that do include a dyadic analysis, approaches are highly variable and often poorly described [9, 20]. Despite the importance of foregrounding the dyadic relationship throughout study design, few studies address if or how they were conceptualized at a dyadic level [7].

The overall aim of this study was to review and summarize qualitative literature where data have been collected through separate individual interviews from patient and care partner dyads and analyzed at the dyadic level. This review provides insight into when and how this methodology has been used in research involving care partnerships and the various methods of analysis employed. Our primary objectives were to: (1) identify methodological approaches and analytic methods used for qualitative dyadic analysis when dyad partners are interviewed separately; (2) explore their analytic advantages, challenges, justifications, and underlying epistemological assumptions; and (3) examine if and how studies reporting a dyadic analysis are conceptualized at a dyadic level. Given it is not uncommon for researchers to have multiperspective data available to them that was not initially collected with the intention of being analyzed at a dyadic level [15], a secondary objective was to identify examples of secondary dyadic analyses of multiperspective data from patients and care partners and their associated advantages and limitations reported by the authors.

## Methods

This review was guided by the Joanna Briggs Institute (JBI) methodology for scoping reviews [21] and is reported in accordance with the Preferred Reporting Items for Systematic reviews and Meta-Analyses extension for Scoping Reviews (PRIMSA-ScR) checklist [22] (Appendix A, Additional File 1). The full protocol was drafted following JBI Best Practices [23] and the PRISMA Protocols statement [24] and was registered in [removed for review]. Our study did not require an ethical board approval because it did not directly involve humans or animals.

### Inclusion criteria

Eligible studies included those published in full in English in peer reviewed journals from 2010 to the start of the study (February 2024), excluding opinion pieces. We established 2010 as a cut-off because Eisikovits and Koren’s [7] seminal paper on qualitative dyadic analysis, which is largely credited for a recent interest in qualitative dyadic studies [25], was published that year, and we were interested in research that was published at that time or thereafter. All articles had to report a primary or secondary qualitative analysis, at the level of the dyad, of semi-structured interview data collected from dyad members independently and separately. Reviews and methodological papers focusing on qualitative dyadic analysis, in which dyad members were interviewed separately, were also eligible [21]. Studies with no details concerning the methods of dyadic analysis, those using structured clinical interviews, surveys, or questionnaires, and those in which data were analyzed exclusively at the aggregate or group level were excluded. Dyads had to be composed of adults in a care partnership, where one member had a health condition and shared a close relationship with the other member who provided care or emotional, physical, or cognitive support within the context of a personal, rather than professional, relationship [2, 3]. Care partnerships were not excluded if the care partner held a professional caregiving role outside the dyad, provided their role within the dyad was as an unpaid caregiver.

### Search strategy

We developed comprehensive search strategies for MEDLINE, EMBASE, APA PsycInfo, the Cumulative Index to Nursing and Allied Health Literature (CINAHL), and Sociological Abstracts (Appendix B, Additional File 1) following the three-step method recommended by the JBI [21, 26]. Concept mapping was used to break the review question into two concepts: (1) studies using a dyadic approach to data collection and/or analysis and (2) care partners. Search terms for each concept were derived from text words in titles and abstracts and index terms of relevant articles identified in initial scoping searches. Validated database-specific search filters designed to locate qualitative research were also included [27–29]. Filters were adapted for EMBASE and Sociological Abstracts with guidance from an information specialist, as validated filters for these databases were unavailable. The search was limited to human participants and to articles published from 2010 onwards. The final search strategy was validated by testing its ability to identify known relevant articles identified through initial scoping searches and peer reviewed by a second information specialist using the Peer Review of Electronic Search Strategies (PRESS) Checklist [30] prior to its execution on February 8, 2024. The reference lists of included articles were reviewed for additional eligible articles not captured in the search.

### Source of evidence screening and selection

Following deduplication in EndNote [31], all material generated by the search algorithm was imported into Covidence [32]. All titles and abstracts were screened for eligibility and non-English articles were removed by the first author [33]. Full text review proceeded with each article independently reviewed by two reviewers following a definitions and elaboration document (Appendix C, Additional File 1) to ensure consistency amongst the reviewers, with greater than 80% agreement achieved prior to independent review of the remaining texts. Discrepancies, uncertainties, and all conflicts were resolved through discussion.

### Data extraction

The first author extracted data from eligible studies using an extraction form based on the template provided by the JBI [21]. The following data items were extracted: study authors and location; study objectives; illness or disease process; methodological approaches; methods of analysis, advantages and challenges associated with dyadic analysis; epistemological position; data collection method; saturation considerations; and steps reported to establish methodological rigour. For reasons of feasibility, the first author extracted all data with verification by a second reviewer for accuracy and completeness for fifty articles [21].

### Analysis and presentation of results

Frequency counts were used to summarize data relating to study characteristics including their methodological approaches and methods of analysis. To understand the sources of guidance authors drew upon for dyadic study conceptualization and analysis, we identified all cited dyadic methodological works within the included studies and calculated how frequently each was referenced across the sample. To compare the methods of analysis, the analytic steps reported in each primary research study were summarized staying as close to the original wording as possible. If authors had published additional details about the analysis in a methodological paper, these were also included in the summary. Using an inductive approach to qualitative content analysis [34], analytic summaries were coded in three ways according to: (1) the sequence of analytic steps; (2) the step(s) that facilitated the examination of the dyad as the unit of analysis; and (3) justifications for dyadic analysis provided by the authors. An example of this process is provided in Appendix D, Additional File 1. Guiding epistemological frameworks and analytic advantages and challenges reported by the authors were narratively summarized. To synthesize descriptive results regarding dyadic study conceptualization, a template (Appendix E, Additional File 1) was developed based on recommendations for qualitative dyadic study conceptualization in the literature [7–9, 12, 15, 16, 19, 35–37]. For example, studies that included research questions or aims focussing on a shared experience important to both dyad members or their relationship were classified as having a dyadic aim [7, 8, 36, 37], whereas those that did not were classified as having a non-dyadic aim. The template focused on the following steps of the research process: study design, participant selection, data collection, and aspects of methodological rigour. All graphs were created using Microsoft Excel (Version 16.97.2, Microsoft Corporation).

## Results

**Fig. 2:**
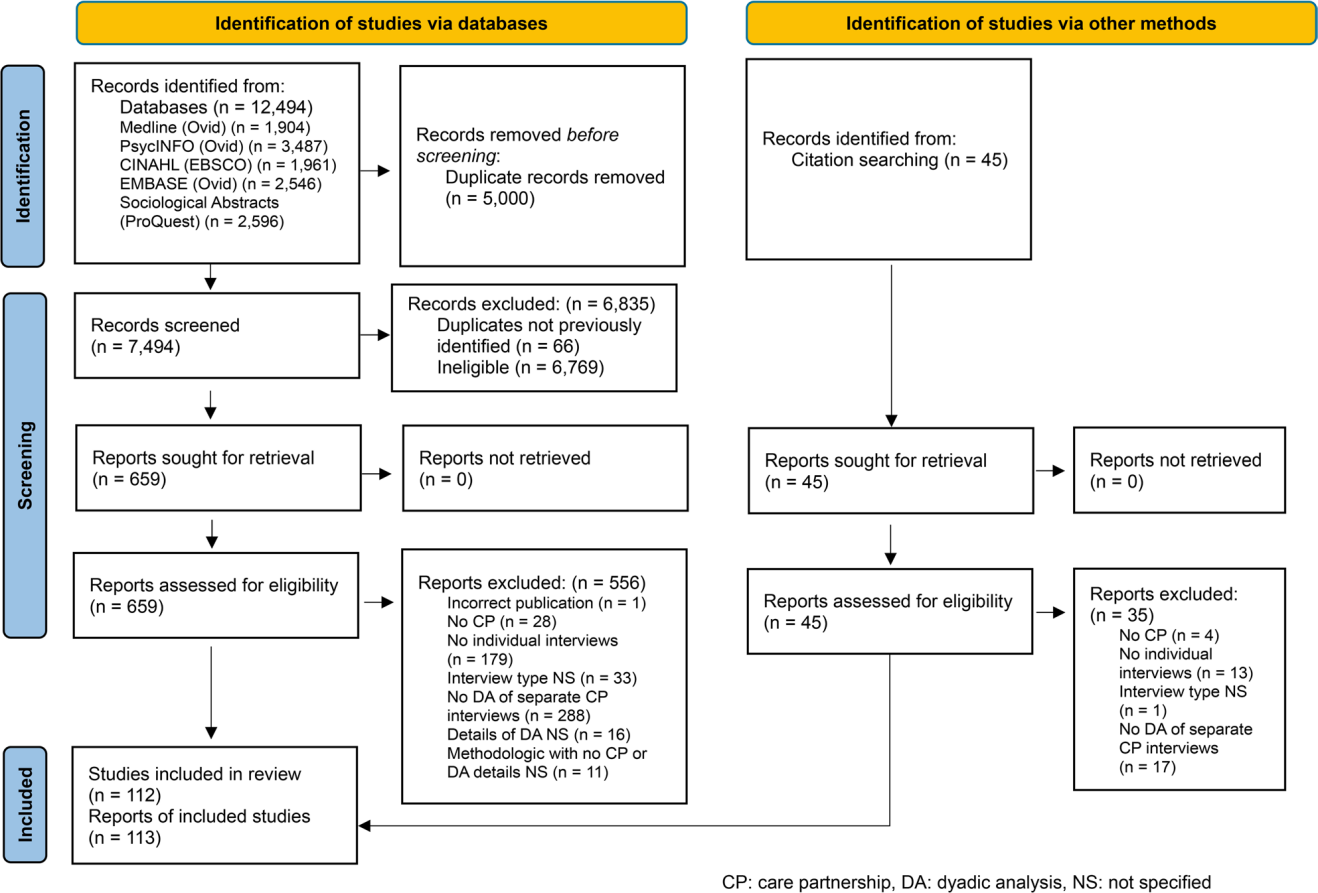
PRISMA flow chart

A total of 7,494 citations and 704 potentially relevant full-text articles were screened. Subsequently, 113 reports of 112 unique studies [9, 12, 20, 38–147] (Supplementary Table 1, Additional File 1), fulfilled the eligibility criteria and were included. Two articles [60, 61] reported results from a single analysis of the same data set and were treated as one study. Two articles reported secondary analyses [101, 109] (case studies) of already published data [100, 110]; however, each was treated as a separate study since their methods differed and this was central to our research questions. In eight instances, two articles appeared to report findings from the same sample [48, 53]; [63, 64]; [79, 125]; [90, 120]; [83, 84]; [105, 106]; [132, 133]; [141, 142]. Since the authors did not explicitly indicate that the findings were from a previously published study and the reported methods differed, each was treated as a separate study. Figure 2 details the selection process. Reasons for exclusion included incorrect publication type (0.2%; *n* = 1), care partnership not studied (5%; *n* = 32), no individual interviews (32%; *n* = 192), interview type not specified (6%; *n* = 34), no dyadic analysis of separate care partner interviews (52%; *n* = 305), details of dyadic analysis not specified (3%; *n* = 16), methodologic with no care partnerships or dyadic analysis details not specified (2%; *n* = 11). Further details regarding the excluded sources reviewed in full are provided in Appendix F, Additional File 1. A summary of the characteristics of included studies is provided in Table 1 and additional details are reported in Supplementary Tables 1–3, Additional File 1.

**Table 1:** Description of characteristics of included articles

Article characteristics	n/113 (%)
Type of evidence source	
Primary research study	107 (94.7)
Methodological study	6 (5.3)
Type of analysis	
Primary	99 (87.6)
Secondary	8 (7.1)
Methodological	6 (5.3)
Year of publication	
2010–2014	21 (18.6)
2015–2019	42 (37.2)
2020–2024^*^	50 (44.2)

Characteristics of primary research studies	n/106 ^†^ (%)
Region where study conducted	
North America	45 (42.5)
Europe	39 (36.8)
Africa	8 (7.5)
Asia	7 (6.6)
Australia	4 (3.8)
Middle East	3 (2.8)
Type of illness, disease process or injury	
Neurological	25 (23.6)
Cancer	23 (21.7)
Infectious disease	16 (15.1)
Cardiac	9 (8.5)
Psychiatric	5 (4.7)
Nephrological	4 (3.8)
Endocrine	3 (2.8)
Musculoskeletal/rheumatologic	3 (2.8)
Reproductive	2 (1.9)
Respiratory	2 (1.9)
Multisystem	1 (0.9)
Physical illness or disability NOS	7 (6.6)
Multiple medical conditions	6 (5.7)
Study design	
Qualitative (single time point)	96 (90.6)
Qualitative longitudinal	6 (5.7)
Mixed methods	4 (3.8)
Dyadic or multiperspective methodology^‡^ reported	
Yes	20 (18.9)
No	86 (81.1)
*Other methodological approach reported* ^§^	*39 (36.8)*
*No methodological approach reported* ^§^	*47 (44.3)*

*NOS* Not otherwise specified

^*^Searches completed during the first week of February, 2024

^†^2 articles reported results from same analysis and were considered as single study

^‡^Reported foregrounding dyad as the unit of analysis in study design

^§^Apart from qualitative design

### Characteristics of included studies

The majority of included articles reported primary research studies (95%; *n* = 107). Of the six methodological articles [9, 12, 20, 54, 92, 130], four were in reference to one or more primary research articles in the review [60, 61, 88, 131, 139]. Eight articles reported secondary analyses [42, 86, 96, 98, 99, 101, 109, 140], three of which included data originally collected with the intention of analyzing it at the dyadic level [99, 101, 109]. The authors of one secondary analysis noted that the data analyzed had been collected without the intention of analyzing it at the dyadic level [98], while the remaining four studies [42, 86, 96, 140] did not include such details.

The number of articles published between 2010 and 2014 (19%; *n* = 21) more than doubled between January 2020 and February 2024 (44%; *n* = 50). Just over three quarters of the primary research studies were conducted in North America (43%; *n* = 45) or Europe (37%; *n* = 39). Participants were primarily affected by chronic illnesses with neurological conditions (24%; *n* = 25) and cancer (22%; *n* = 23) being the most common. Articles were published in a wide variety of journals (*n* = 75) from across multiple disciplines (Table 2).

**Table 2:** Journals in which included articles were published

Number of articles published	Number of journals	Journal names
≥5	1	Qualitative Health Research (*n* = 8 articles)
4	2	Journal of Cardiovascular Nursing, Journal of Health Psychology
3	8	Aging & Mental Health, AIDS and Behavior, Biomed Central (BMC)^*^, Dementia, European Journal of Oncology Nursing, Journal of Clinical Nursing, PLoS ONE, Supportive Care in Cancer
2	9	British Medical Journal, Cancer Nursing, Culture, Health & Sexuality, Families, Relationships and Societies, Frontiers in Psychology, Journal of Cancer Survivorship, Psycho-Oncology, Social Science & Medicine, The Gerontologist
1	55	AIDS Care, AIDS Patient Care & STDs, American Journal of Hospice & Palliative Medicine, Applied Nursing Research, Autism in Adulthood, Brain Injury, British Journal of Health Psychology, British Journal of Social Work, Chronic Illness, Clinical Nursing Research, Communication Monographs, Couple and Family Psychology: Research and Practice, Diabetes Spectrum, Disability & Society, Disability and Health Journal, Disability and Rehabilitation, Drug and Alcohol Dependence, European Journal of Cancer Care, Family Process, Frontiers in Oncology, Frontiers in Psychiatry, Health, Health and Social Care in the Community, Health Communication, Health Expectations, Health Psychology, Hemodialysis International, International Journal of Care and Caring, International Journal of Nursing Studies, Journal of Aging Studies, Journal of Alzheimer’s Disease, Journal of Applied Gerontology, Journal of Cardiopulmonary Rehabilitation and Prevention, Journal of Gender Studies, Journal of Gerontological Social Work, Journal of Health and Social Behavior, Journal of HIV/AIDS & Social Services, Journal of Holistic Nursing, Journal of Psychosocial Oncology, Journal of Social and Personal Relationships, Journal of Social Welfare & Family Law, Journal of Viral Hepatitis, Journals of Gerontology: Social Sciences, Musculoskeletal Care, Neuroethics, Neuropsychological Rehabilitation, Patient Education and Counseling, Psychology & Health, Quality of Life Research, Research in Social and Administrative Pharmacy, Research on Aging, Scandinavian Journal of Occupational Therapy, Social Work in Health Care, Social Work in Public Health, Sociology of Health & Illness

^*^BMC Public Health *n* = 2, BMC Palliative Care *n* = 1

### Methodological approaches and analytic methods

**Fig. 3:**
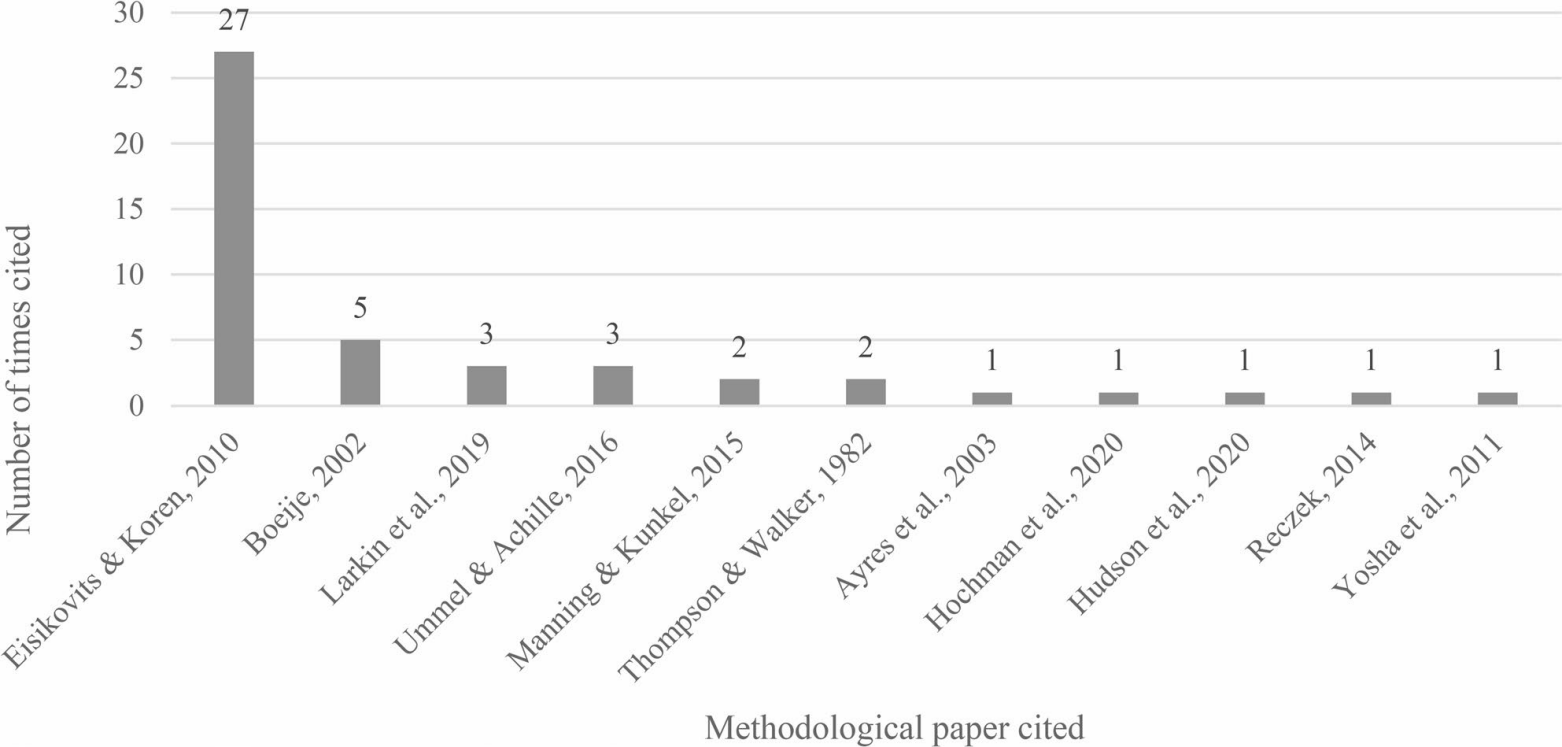
Dyadic methodological works cited in included studies

Forty of the included studies included a citation for at least one previously published methodological paper concerning dyadic study design or analysis that guided their approach (Fig. 3). Only two met eligibility criteria and were included in this review [9, 12]. The remaining nine [7, 15, 19, 25, 35–37, 148, 149] did not specifically address qualitative dyadic analysis of care partnerships as outlined in this review’s inclusion criteria; and in three cases [35, 37, 148], the studies were also published prior to 2010. Eisikovits and Koren [7], Ummel and Achille [12], and Larkin and colleagues [36] outline approaches to dyadic data collection and analysis within the phenomenological tradition, while Ayres and colleagues [148] and Boeije [35] describe approaches informed by case study research and grounded theory, respectively. Reczek [19] and Manning and Kunkel [25] examine methodological frameworks in family and communication studies. Hudson and colleagues [9], Yosha and colleagues [15], and Hochman and colleagues [149] each present their own distinct approaches to qualitative dyadic analysis. Thompson and Walker [37] discuss quantitative dyadic study conceptualization.

A specific qualitative methodology was reported in just over half of the included studies (56%; *n* = 59), with only 19% (*n* = 20) explicitly referencing a dyadic study design despite all 106 studies including a dyadic analysis (Supplementary Table 4, Additional File 1). Variable terminology was used to describe methodology that foregrounded the dyad as the primary unit of analysis including dyadic/multiperspectival design [66, 95, 97, 107], dyadic study design [45, 118, 138], dyadic research design [113], dyadic approach [123], multiperspective analysis methodology [60, 61], dyadic qualitative methodology [68], longitudinal qualitative partner study [72], qualitative approach that elicited both patient and caregiver perspectives [105], methodology that positioned partnerships as the primary unit of analysis [111], case study where the dyad was the case [56, 71, 101, 109], an original dyadic perspective [44], and a qualitative approach in a dyadic way [43]. A dyadic design used in combination with a second qualitative methodology (phenomenology [43, 44, 66, 95, 97, 107, 113], case study [56, 71, 98, 110], qualitative description [123]) was reported in 11% of included studies (*n* = 12), while a specific qualitative methodology, without acknowledging the dyad as a unit of analysis in the design, was reported in 32% (*n* = 34). No underlying methodology apart from a qualitative design was reported in 44% of included studies (*n* = 47), all of which included details regarding data collection and analysis but lacked clarity about how the methods connected to an underlying methodological orientation.

At least one other analytic method alongside dyadic analysis was reported in 93% of included studies (*n* = 98) (Supplementary Table 5, Additional File 1). Thematic analysis (20%; *n* = 21), procedures of grounded theory (14%; *n* = 15), and framework analysis (13%; *n* = 14) were the most common methods used in combination with dyadic analysis. Content analysis, interpretative phenomenological analysis, and interpretive description were also used in two or more studies. No additional method of analysis was reported in 8% of included studies (*n* = 8).

#### Sequence of analytic steps

**Fig. 4:**
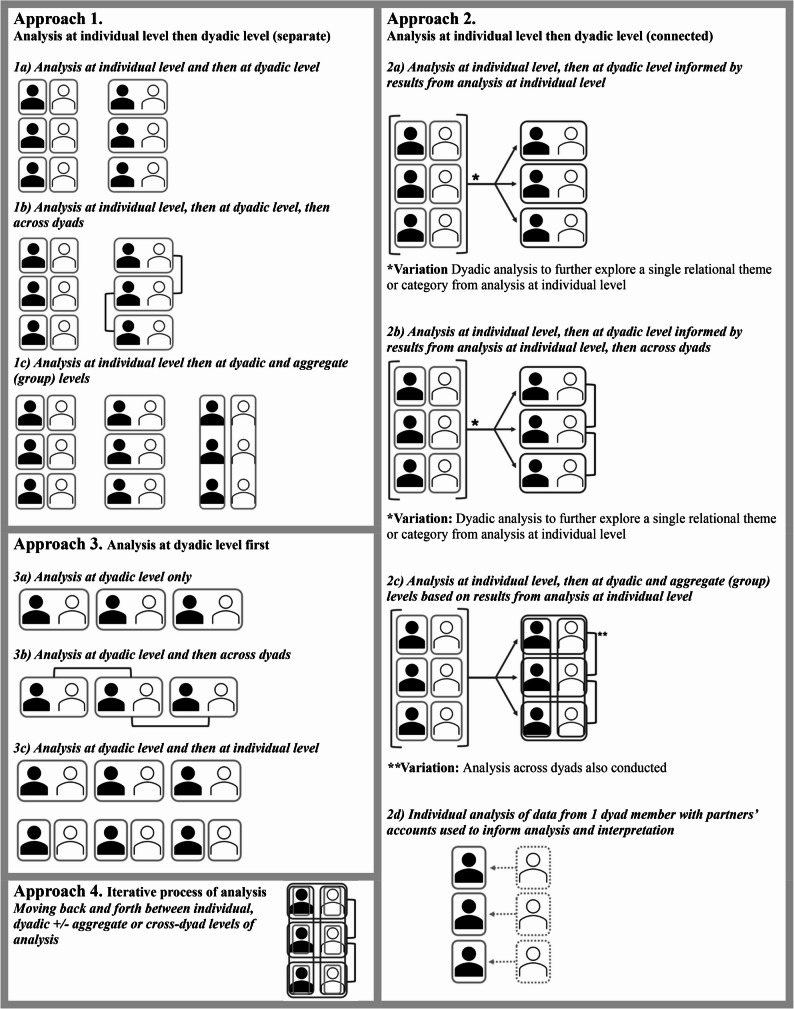
Approaches to sequence of individual and dyadic analyses

Data were analyzed at both the individual and dyadic levels in three quarters of studies (*n* = 79). While no contradictions were observed between the findings from individual and dyadic analyses, dyadic analysis often yielded new insights or resulted in more nuanced findings compared to individual level analysis alone [9, 38]. The order in which these two levels of analysis were conducted, and whether they were connected, varied. Sequences of analyses were inductively categorized into four overall approaches (Fig. 4).

One approach (Approach 1, Fig. 4) involved analysis at the individual level and then at the dyadic level without any clear connection between the two (17%; *n* = 18). An additional analytic step, either across dyads or at the aggregate (group) level, was conducted in four and six of these studies, respectively.

A second approach (Approach 2, Fig. 4), beginning with individual-level analysis, which informed the subsequent dyadic analysis, was the most common approach, implemented in 44% of included studies (*n* = 47). For example, after conducting content analysis at the individual level to identify themes, Demirtepe-Saygılı [68] performed a dyadic analysis to compare all themes within each dyad. Several studies followed a similar process but used dyadic analysis to explore only one or two relational themes or categories developed in the individual-level analysis [38, 55, 88, 98, 116, 140]. A second variation involved analyzing one set of dyad members’ data individually and then extending the analysis by using each of their partners’ accounts to inform the analysis [53, 70] (2d, Fig. 4). As in the first approach, many studies using this second approach included an additional step, where data were analyzed across dyads (*n* = 15) or at the group level (*n* = 8) (2b and 2c, Fig. 4).

A third approach began with analysis at the dyadic level (27%; *n* = 29), (Approach 3, Fig. 4). Data were subsequently analyzed across dyads in approximately half of these studies (*n* = 15), or in two cases [132, 133], at the individual level. A fourth approach (Approach 4, Fig. 4), adopted in 4% of studies (*n* = 4) [47, 74, 91, 121], involved an explicitly iterative process of moving back and forth between individual and dyadic levels of analysis until a key set of themes were determined. 8% of studies (*n* = 8) included both individual and dyadic analyses, but their sequence, or whether they were connected, were not reported [43, 87, 90, 120, 129, 134, 138, 144].

The second approach aligned most closely with strategies described in six methodological articles [7, 9, 12, 35, 36, 149], while the third resembled that of Yosha and colleagues [15]. Approaches one and four did not align with previously published methodological guidance. The forty articles that referenced a dyadic methodological source often deviated from the guidance they cited.

#### Process of dyadic analysis

**Fig. 5:**
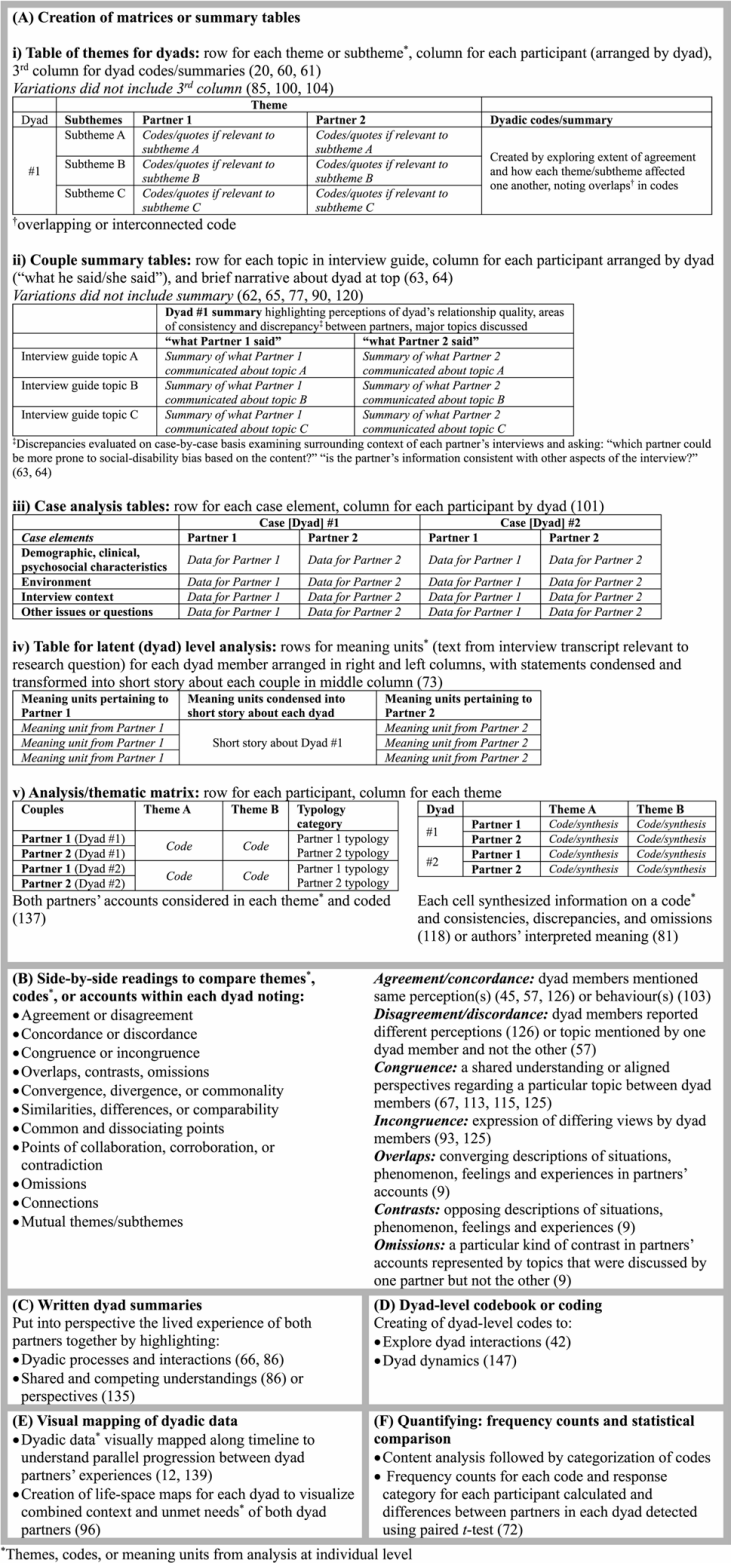
Approaches to dyadic analysis

No two methods for analyzing the data from each partner together as a dyad were exactly the same. The process varied regardless of the other analytic methods employed, although it was sometimes linked to them. For example, constant comparison of codes within dyads was described in studies in which grounded theory techniques were used [45, 58, 78]. Despite the extensive variability, there were commonalities in the procedures used, which were inductively grouped into six overall approaches (Fig. 5). Side-by-side reading of the paired data to compare and categorize themes, codes, or accounts within each dyad was the most frequent approach used in just over half of the included studies (54%; *n* = 57). Another approach involved the use of data matrices to visually compare each dyad member’s data, dyad by dyad (20%; *n* = 21). This strategy was often used in studies that employed framework analysis. Less common approaches included creation of a dyadic-level codebook (7%; *n* = 7), written dyad summaries (3%; *n* = 3), visual mapping of dyadic data (2%; *n* = 2), and frequency counts of codes with statistical comparison between dyad members (1%; *n* = 1). A combination of approaches was used in 7% of studies (*n* = 7) and transcripts from each dyad were analyzed together as a whole in 12% of studies (*n* = 13), but details about how this was done were not provided.

All of the approaches involved comparing partners within each dyad in some way. The first two (Approaches A and B, Fig. 5) were used to identify agreement or disagreement within the dyad, however variable terminology was used. Authors did not routinely define these terms or articulate how they were operationalized, and in cases where they did, this was often done inconsistently (Fig. 5). Authors rarely addressed how they analyzed topics discussed by one partner but not the other, and when they did, their approaches differed. Hudson and colleagues [9, 88] conceptualized such instances as “omissions,” a particular kind of contrast in partners’ accounts, while Catona and colleagues [57] labeled them as disagreements and Demirtepe-Saygılı [68] simply excluded topics discussed by only one dyad member from their analysis.

Written dyad summaries were used to put into perspective the lived experience of both partners by highlighting dyadic processes and interactions or shared and competing perspectives [66, 86, 135]. Dyadic coding involved creating codes to explore dyad-level interactions or dynamics [42, 147]. Visual mapping involved graphically depicting dyadic data to illustrate combined contexts by creating dyad life-space maps [96] or to mirror dyad partners’ experiences along a timeline [12, 139].

### Advantages and challenges of the analytic methods

Specific analytic advantages were not reported with the exception of two studies in which the authors noted that dyadic-level charting enabled systematic examination of individual and dyadic experiences [20, 104]. Challenges associated with establishing congruence or incongruence in dyadic data [39, 54, 115] and the analysis of topics discussed by one dyad member [9, 20, 54] were each noted in three studies. A separate but related limitation was reported in two studies [93, 98] in which the authors noted that the absence of specific questions during data collection focussing on the dyad relationship or facilitating comparison of each partner’s experience resulted in less analytic depth. Challenges associated with creating a single summary that captured the complexity of dyadic experiences, while also attempting to separate them into simplistic categories, all while maintaining a clear audit trail of this process were identified in one study [20]. Challenges related to confidentiality of the dyad, such as when seeking participant validation or in data presentation, were reported in three studies [9, 12, 104]. The time consuming and resource-intensive nature of dyadic analysis was reported in three studies [9, 20, 92], one of which also noted the lack of available software that might help mitigate this [20]. The challenge of some dyads requesting to be interviewed together was raised in one study [104]; however, how this was addressed during analysis or its implications were not elaborated.

In two studies [98, 140], the authors acknowledged that secondary analysis of data collected for a different purpose was an inherent limitation to their studies, but also argued that in the case where few studies have specifically addressed dyadic experience, findings of such analyses were important for knowledge advancement. No specific analytic advantages or challenges were described in other reports of secondary analyses [42, 86, 96, 99, 101, 109].

### Justifications for dyadic analysis and their alignment with underlying epistemological assumptions

Reasons provided for performing dyadic analysis were analyzed inductively into ten overarching justifications by the first author (Supplementary Fig. 1, Additional File 1). The most common rationales were to compare and contrast the experience of one partner with the other and to explore dyadic-level interactions and processes, reported in 22% (*n* = 27) and 14% (*n* = 17) of studies, respectively. Other justifications included understanding the experience of both partners as individuals and as a unit (13%; *n* = 16) or capturing and enhancing an understanding of experiences of the dyad (13%; *n* = 16). Although these justifications are similar, those categorized under the former emphasized understanding experience at both the individual and dyadic level, while those placed in the latter group focused on understanding experience at the dyadic level only. Reaching a deeper understanding of joint experiences greater than the sum of two parts was reported in 5% of studies (*n* = 6), suggesting that epistemologically some authors used dyadic analysis to create a new and deeper understanding exceeding the two individual parts. Dyadic analysis as a means to access a more complete view of reality, strengthen the overall analysis, or enhance trustworthiness and validity was reported in 5% of studies (*n* = 6).

A guiding epistemology was clearly reported in only 12% of studies (*n* = 13) (Supplementary Table 1, Additional File 1) and included interpretivist [38, 73, 88, 95], constructivist- [97, 139] or constructionist- [47, 126] interpretivist, naturalistic [39, 142], and realist [85, 104, 135] paradigms. Authors did not connect their rationale for dyadic analysis or choice of analytic methods with the underlying epistemological framework in any of the studies where the latter was reported.

### Dyadic study conceptualization

**Fig. 6:**
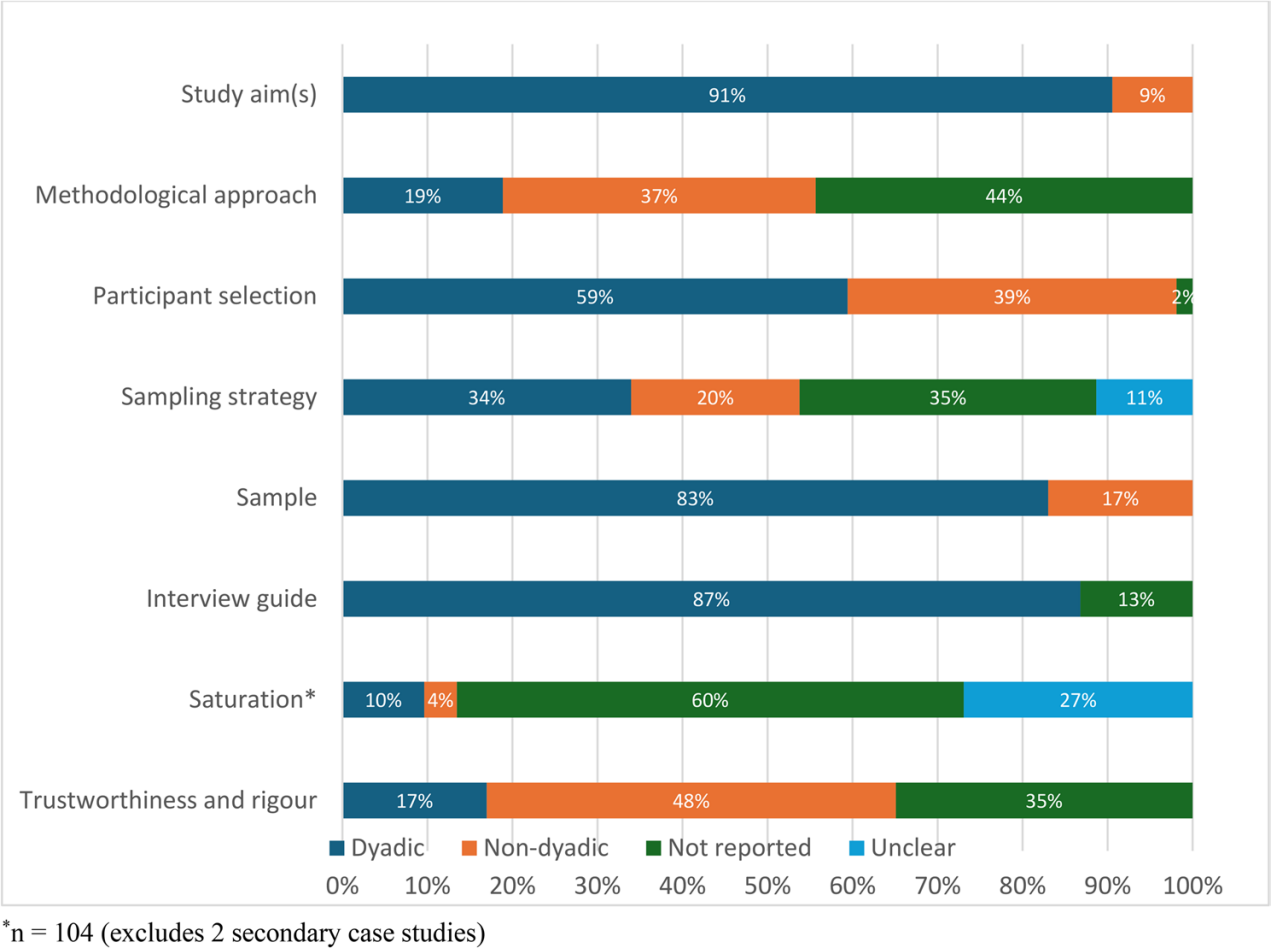
Dyadic conceptualization at different steps of the research process apart from analysis (*n* = 106)

The 106 studies were categorized using our template (Appendix E and Supplementary Table 6, Additional File 1) to explore if and how they were conceptualized at a dyadic level beyond analysis (Fig. 6). Study or research aims focused on the shared experience of dyad members or the relationship between them in 91% (*n* = 96) of studies; however, an overarching dyadic or multiperspective methodology was reported in only 19% (*n* = 20). Study inclusion or exclusion criteria were directed towards dyads and/or both dyad members were required to participate in 59% of studies (*n* = 63). The sampling strategy was directed at the dyad in only 34% (*n* = 36). The study sample was comprised of only dyads in 83% of studies (*n* = 88), while the remaining 17% also included either singletons or triads. Identical or similar interview guides were used for each dyad member in all studies that reported such details (87%; *n* = 92). Saturation at the dyadic level was reported in 10% of studies (*n* = 10). Methodological rigour in reference to dyadic data collection or analysis was reported in 17% of studies (*n* = 18). When considering only studies in which an underlying dyadic methodology was reported, sampling strategy, saturation, and methodological rigour were still not routinely considered at the dyadic level (Supplementary Fig. 2, Additional File 1).

## Discussion

This scoping review sought to identify how and why dyadic analyses are applied to data collected from patients and care partners through separate interviews in the peer-reviewed literature from 2010 onwards. A total of 113 articles, reporting 106 unique primary research studies and six methodological papers, were included in the review. A recent acceleration in studies including dyadic analysis of patient and care partner data was observed, with publications more than doubling over the last decade. Participants were predominantly impacted by chronic health conditions, reinforcing that chronic illness is a dyadic (or interpersonal) experience impacting both individuals living with it and those who care for them.

A wide array of qualitative methodologies and analytic methods were adopted. A dyadic analysis was included in all of the studies reviewed; however, only twenty reported a guiding dyadic methodology, often described using variable terminology. When reported, dyadic designs were generally used in combination with a second qualitative methodology. There was also considerable variability in how dyadic analysis was conducted. In most studies, data were analyzed at both the individual and dyadic levels, often with individual-level analysis subsequently informing the dyadic analysis, and sometimes with only relational themes being the focus of the dyad-level analysis. This is in keeping with approaches reported in methodological papers describing qualitative dyadic analysis not exclusive to care partnerships [7–9, 12, 35, 36, 149–151].

The recent surge in studies incorporating dyadic analysis highlights a growing interest in understanding illness as a joint experience that impacts both the individual who is ill and those involved in his or her care. Despite increasing recognition that illness is often a shared experience, the process of analyzing two related accounts together varied across studies and was often poorly described. Rationales for conducting a dyadic analysis were also variable, and while some suggested different epistemological stances, a guiding epistemology was not routinely reported. In some studies, the authors acknowledged that dyadic analysis yielded novel or more refined findings, highlighting its potential to generate knowledge beyond that generated from analysis at the individual level alone, though this was not routinely explored or emphasized.

The purpose and outcomes of multiperspective research are influenced by the researcher’s ontological and epistemological viewpoints [17], which also influence their analytical choices including the way similarities and differences in dyadic accounts are understood [16]. The lack of clarity regarding underlying epistemological assumptions in many studies included in this review may explain some of the ambiguity in their analytic methods, particularly in how similarities and differences were handled.

Given the significance of underlying epistemology to the outcomes of dyadic analysis [17], clearly defining epistemological assumptions, including their connection with analysis, could help address the challenge associated with analyzing dissonant data. From a positivist or postpositivist position, individual reports of joint experiences are viewed as potentially incomplete or unreliable [17]. Discrepancies are understood as gaps at a factual level, which can be resolved by reading accounts together to reach a more objective, comprehensive, valid, and trustworthy representation of experience [7, 16, 17]. Similar accounts may be viewed as “more true,” potentially limiting critical examination of dyad agreement [16, 54], whereas researchers may instinctively try to overcome dissonance in accounts by going into more analytical depth [17]. Grounded in the assumption that frequency equates to relevance, researchers adopting a positivist/postpositivist position may also place greater emphasis on topics mentioned more frequently by both dyad members [54].

From a constructivist, social constructionist, or interpretivist standpoint, differences in accounts are not analyzed to reach a single truth, but rather to understand partial truths or differences in socially constructed realities [19]. Contradictions are not viewed as factual inconsistencies, but rather as reflections of how dyad members engage with similar or different aspects of their joint experience [16], with the distinct ways they construct their accounts data in itself [19]. By considering the individual salience that the same experience holds for each dyad member, a deeper understanding of the shared phenomenon can be reached [16, 17, 19]. Less frequently discussed topics, or those discussed by one member but not the other, may reveal points of tension and hold greater analytical significance than those extensively discussed by both members [9, 54]. Exploration of such “omissions” may help identify which factors shape each individual’s experience and how they differ within the dyad [9]. What is left unsaid may be especially poignant in patient and care partner dyad research, where silences might be intensified when an ill person views others, such as a caregiver, as more knowing or when suffering feels incomprehensible and beyond words [152].

Challenges associated with creating a single summary capturing the complexity of dyadic experience while maintaining a clear audit trail were addressed by using matrices to compare individual accounts, creating broad summary codes, including detailed quotes, and reflexively documenting the analytic process [20]. While not acknowledged in any of the included studies, eighteen had samples that included triads or singletons raising analytic questions of how such non-dyadic data should be handled in a dyadic analysis. In one study, triads were analyzed by proceeding in pairs [85], while in another, data collected from unmatched participants were used to confirm data saturation at the individual-level, but were excluded in the subsequent dyadic analysis [45]. Considering two perspectives introduces complexity and while it is possible that saturation may be reached at the individual level, a larger sample of dyads may be required to reach it at the dyadic level [35]. The analytic implications of requests for joint interviews or the dropout of one dyad member but not the other [130] are key considerations in dyadic research. Further investigation of the analytic implications of including non-dyadic data, and whether it should be included at all, in dyadic studies is needed.

When disseminating results from a dyadic analysis based on data collected separately from each dyad member, maintaining confidentiality is especially critical to ethical dyadic research. If participants recognize their own statements, they may also infer what their partner said, potentially breaching the partner’s confidentiality by association [12, 36]. This is particularly relevant to analyses that emphasize incongruences or disagreements. However, measures taken to protect participant confidentiality when disseminating results were rarely reported. Careful decision-making about how data are presented is essential in dyadic studies and may involve strategies such as presenting sensitive extracts without attribution or reporting dyadic data at the general or group level [12, 36].

Qualitative dyadic research can be understood as both a study design, in which the dyadic relationship is considered throughout all stages of the research process and as a methodology that informs the selection of methods for data collection (separate or joint interviews) and analysis that synthesizes the experiences of both individuals within the dyadic unit together [7, 9, 12]. While most of the included studies had dyadic research aims and data collection methods, only 19% referenced a dyadic methodology and sampling, saturation, and methodological rigour were not routinely addressed at the dyadic level. Further exploration of methodological rigour in qualitative dyadic studies is needed, including how it aligns with sampling, data collection, saturation, methods of analysis, underlying epistemological assumptions, and ethical considerations.

Although not uncommon for researchers to have multiperspective data that was not initially collected with the intention of being analyzed at a dyadic level [15], we identified only one secondary analysis [98] in which the authors explicitly acknowledged that the data analyzed had been collected without the intention of analyzing it at the dyadic level. While acknowledging this as an inherent limitation, similar to previous assertions made by Yosha and colleagues [15], the authors argued that in cases where few studies have specifically addressed dyadic experience, findings of such analyses are valuable for advancing knowledge [98]. Researchers who have collected data from dyad members for separate purposes, such as in two distinct studies examining each member group as a whole, may consider whether and how a secondary analysis at the dyadic level could yield additional insights. However, it is crucial to consider the compatibility of the philosophical foundations and qualitative methodologies underpinning the original studies.

### Challenges, limitations and strengths

This review was limited to peer-reviewed studies published in English from 2010 onward and therefore did not include potentially relevant non-English articles or those published prior to 2010. Additionally, we did not address approaches to analysis across dyads (inter-dyad analysis), multiperspective or triadic analysis (where analysis extends beyond the dyad to more complex multi-member units), reporting and presentation of qualitative dyadic data, or ethical considerations of performing research with participants who share a relationship. These topics were beyond the scope of this study and could be explored in future reviews. Furthermore, we did not include research reporting dyadic analysis outside of adult care partnerships involving unpaid, non-professional caregivers or dyads that include children or individuals without health conditions. Within the literature we reviewed, we also did not differentiate between types of dyadic partnerships, for example parent-child or spousal relationships, and therefore did not examine whether there are methodological differences according to type of partnership. Examination of dyadic analysis across different partnership types is an important direction for future research. The use of inconsistent terminology in the literature may have led to the exclusion of studies that effectively included a dyadic analysis, despite our efforts to optimize search sensitivity.

This scoping review was a substantial undertaking and our results are current only as of February 2024. We believe it provides important information about qualitative dyadic analysis and is the most comprehensive review that we are currently aware of. Strengths of this review include its adherence to a systematic and rigorous approach in accordance with PRISMA-ScR guidelines [22] and its synthesis of a diverse range of approaches to qualitative dyadic analysis, including those not explicitly labeled as such.

## Conclusions and recommendations

Numerous approaches to qualitative dyadic analysis of patient and care partner data have been reported, often blending methods from different qualitative traditions, with inconsistent and poorly described analytic steps. Challenges related to handling dissonant data within dyads were identified. Few studies were conceptualized at the dyadic level and epistemological assumptions were rarely discussed despite their essential role in grounding dyadic analysis.

Key considerations for designing a dyadic study with data collected through separate interviews from patient and care partner dyads that were identified by the first author through the process of conducting this review are outlined in Table 3. We hope that this table will serve as a useful starting point for those interested in designing and conducting dyadic qualitative studies. Collaço and colleagues [20] also provide a detailed account of their approach making their work a valuable starting point for those seeking a step-by-step guide for qualitative dyadic analysis of care partnerships.

**Table 3:** Prompt questions to consider when designing a qualitative dyadic study of data collected through individual interviews with each dyad member separately

Research steps		Questions to consider		
**Study design**	Research aim(s)	Is the study objective concerned with the shared experience of dyad members or the relationship between them?		
	Methodological approach	Is the study conceptualized at a dyadic level (foregrounding the dyad as the unit of analysis) throughout its design? Will a second qualitative methodological approach, such as phenomenology, grounded theory, qualitative description, or case study, be integrated with the dyadic study design?		
	Epistemological assumptions	What underlying epistemology or theoretical paradigm is the study guided by and does the intended purpose of the dyadic analysis align with it? For example, through dyadic analysis is the aim to (17, 19):		***Suitable paradigm***
		• Understand and capture a more complete version of the truth?		*Positivism/postpositivism*
		• Understand how dyad members construct and interpret their own social reality within a shared reality?		*Social constructionism*
		• Enrich understanding and demonstrate potentially contradictory accounts?		*Constructivism*
		• Facilitate social change and identify mechanisms of oppression and inequality?		*Critical realism*
**Recruitment and sampling**	Participant selection	Will inclusion and/or exclusion criteria be unique for each dyad member or directed at both members as a unit? Will participation of both dyad members be required for inclusion in the study?		
	Participant recruitment	Will recruitment be sequential *(a stepwise approach where one dyad member is recruited who then identifies their partner who is subsequently recruited)* or concurrent *(dyad members recruited together)*?		
	Consent	Will consent be obtained from both dyad members together or separately? Will dyad members be given the chance to decline participation without their partner being present?		
	Sampling strategy	Will the sampling strategy be directed at the dyad *(for example*,* sampling for maximum variation across dyad characteristics such as type of partnership)*?		
	Sample	Will only dyads be included? • If data is collected from only one dyad member due to drop out of their partner, will the unmatched partner’s data still be included in the analysis? • If the data of an unmatched partner will not be used, have the ethical implications of collecting data and then excluding it been considered? • In cases where a dyad member shares a relationship with two individuals who both meet inclusion criteria, will one of the unit’s members be excluded? If a member is excluded, how will this decision be made?		
**Data collection**	Interview guide/questions	Will dyad members be asked the same, similar, or related questions? Will questions designed to allow for a more direct comparison of perspectives be included? Will lines of inquiry in the interview with one dyad member be followed up in the interview with their partner? If so, how will confidentiality be maintained?		
	Interviewer	Will dyad members be interviewed by the same individual or different individuals?		
		***If interviewed by the same individual***:	***If interviewed by different individuals***:	
		• How will confidentiality be maintained? • How will the interviewer account for the possibility of being influenced by the answers of the participant interviewed first when interviewing their partner?	• Will the same interviewer consistently interview one type of dyad member, or will interviewers alternate between dyad member type?	
	Interview timing	Will dyad members be interviewed consecutively or simultaneously? Will dyads who request to be interviewed together still be interviewed?		
		***If interviewed consecutively***:	***If interviewed simultaneously***:	
		• How much time will be permitted to pass between interviews? • Will the order in which participants are interviewed be varied?	• Are two interviewers available to conduct both interviews concurrently? • Are two private spaces available to conduct the interviews separately?	
Data analysis and reporting	Sequence of analysis	Will analysis at the individual level be conducted prior to at the dyadic level? If individual analysis precedes dyadic analysis: • What method of analysis will be used and how does it align with the chosen methodology? • Will analysis at the individual level inform the analysis at the dyadic level?		
	Dyadic analysis	How will the accounts of each dyad member be synthesized together *(for example*,* through side-by-side readings to compare themes*,* codes*,* or accounts; matrices to visually compare each dyad member’s data*,* dyad by dyad; written dyad summaries; creation of a dyad-level code book to code interactions or dynamics; visually mapping data; or quantifying codes and then using statistical comparison techniques)*?		
		What is the focus of the dyadic analysis *(for example*,* to explore agreement and disagreement*,* overlap and contrast*,* or convergence and divergence etc. within dyads)*? How will dissonant data be evaluated in a way that is consistent with epistemological assumptions? How will topics discussed by one partner, but not the other, be analyzed?		
		Will the analysis be at the descriptive level *(comparing the ways dyads discuss and present their experiences and perception*,* and not attempting to go beyond these representations)* or extended to an interpretive level *(going beyond each partner’s individual responses to create a credible interpretative perspective of how their experiences intersect)* and is this decision consistent with the underlying methodology and guiding epistemology?		
		When will the analysis begin? If prior to completion of data collection, will analysis at the individual and/or dyadic level inform data collection, and is this consistent with the underlying methodology?		
		If two researchers conducted interviews, will both participate in data analysis? If yes: how will the risk that each interviewer might have a more complete and comprehensive understanding of the perspective of his or her interviewees be mitigated? If no: how will the single researcher/interviewer account for having a more complete understanding of one set of interviewees prior to analysis?		
		How will a clear audit trail be maintained? How will the analytic process be reflexively documented?		
	Saturation	Will saturation be determined at the dyadic level *(for example*,* when dyads bring similar findings and further dyads do not bring new findings that had not already been identified)* or at the individual level prior to dyadic analysis *(for example*,* interviewing participants until saturation in the individual-level analysis is attained followed by analysis at the dyadic level without further consideration of saturation)*?		
	Data reporting	If analyses at both the individual and dyadic level were conducted, will the results be reported separately or will they be integrated together? How will confidentiality be ensured *(for example*,* ensuring that dyad partners cannot identify one another from a series of quotes or comments*,* particularly if quotes are presented from each dyad member to illustrate a finding)*?		

With the recent acceleration in studies incorporating dyadic analysis, clearer methodological guidance is needed to support this research approach. Future research should examine how different analytic approaches, including how their integration with methods from different qualitative traditions, influence outcomes and might better suit specific types of research questions. Dyadic data reporting, methods of analysis of complex units that extend beyond the dyad, and methodological rigour in qualitative dyadic studies are also topics for future inquiry.

## Supplementary Information

Additional file1: Appendix A. PRIMSA-ScR checklist. Appendix B. Search strategies. Appendix C. Definition and elaboration document for full text screening. Appendix D. Data synthesis process- sequence of analysis, dyadic analysis step, and justifications for dyadic analysis. Appendix E. Template for dyadic study conceptualization at different research steps. Appendix F. Sources excluded following full-text review. Appendix G. Supplementary tables and figures.

## Data Availability

Data sharing is not applicable to this article as no new data were created or analyzed in this study.

## Abbreviations

CINAHL: Cumulative Index to Nursing and Allied Health Literature
JBI: Joanna Briggs Institute
PRESS: Peer Review of Electronic Search Strategies
PRISMA-ScR: Preferred Reporting Items for Systematic reviews and Meta-Analyses extension for Scoping Reviews
