# Supplementary material for: Qualitative dyadic analysis in care partnership research: a scoping review
Source: BMC Med Res Methodol. 2025 Dec 11;26:7. doi: 10.1186/s12874-025-02722-y (PMC12801508; doi:10.1186/s12874-025-02722-y)
Supplement: Supplementary file 1 — Additional file1: Appendix A. PRIMSA-ScR checklist. Appendix B. Search strategies. Appendix C. Definition and elaboration document for full text screening. Appendix D. Data synthesis process- sequence of analysis, dyadic analysis step, and justifications for dyadic analysis. Appendix E. Template for dyadic study conceptualization at different research steps. Appendix F. Sources excluded following full-text review. Appendix G. Supplementary tables and figures. [file 12874_2025_2722_MOESM1_ESM.docx]

**Additional File 1**

**Table of Contents**

|  | Page |
| --- | --- |
| Appendix A: PRIMSA-ScR checklist | 2 |
| Appendix B: Search strategies | 4 |
| Appendix C: Definition and elaboration document for full text screening | 24 |
| Appendix D: Data synthesis process- sequence of analysis, dyadic analysis step, and justifications for dyadic analysis | 25 |
| Appendix E: Template for dyadic study conceptualization at different research steps | 26 |
| Appendix F: Sources excluded following full-text review | 27 |
| Appendix G: Supplementary tables and figures | 64 |

**Appendix A: PRISMA-ScR checklist**

| **SECTION** | **ITEM** | **PRISMA-ScR CHECKLIST ITEM** | **REPORTED ON PAGE #** |
| --- | --- | --- | --- |
| **TITLE** | | | |
| Title | 1 | Identify the report as a scoping review. | 1 |
| **ABSTRACT** | | | |
| Structured summary | 2 | Provide a structured summary that includes (as applicable): background, objectives, eligibility criteria, sources of evidence, charting methods, results, and conclusions that relate to the review questions and objectives. | 2 |
| **INTRODUCTION** | | | |
| Rationale | 3 | Describe the rationale for the review in the context of what is already known. Explain why the review questions/objectives lend themselves to a scoping review approach. | 4-7 |
| Objectives | 4 | Provide an explicit statement of the questions and objectives being addressed with reference to their key elements (e.g., population or participants, concepts, and context) or other relevant key elements used to conceptualize the review questions and/or objectives. | 6-7 |
| **METHODS** | | | |
| Protocol and registration | 5 | Indicate whether a review protocol exists; state if and where it can be accessed (e.g., a Web address); and if available, provide registration information, including the registration number. | 7 |
| Eligibility criteria | 6 | Specify characteristics of the sources of evidence used as eligibility criteria (e.g., years considered, language, and publication status), and provide a rationale. | 7-8 |
| Information sources | 7 | Describe all information sources in the search (e.g., databases with dates of coverage and contact with authors to identify additional sources), as well as the date the most recent search was executed. | 8 |
| Search | 8 | Present the full electronic search strategy for at least 1 database, including any limits used, such that it could be repeated. | Appendix B |
| Selection of sources of evidence | 9 | State the process for selecting sources of evidence (i.e., screening and eligibility) included in the scoping review. | 8-9 |
| Data charting process | 10 | Describe the methods of charting data from the included sources of evidence (e.g., calibrated forms or forms that have been tested by the team before their use, and whether data charting was done independently or in duplicate) and any processes for obtaining and confirming data from investigators. | 9 |
| Data items | 11 | List and define all variables for which data were sought and any assumptions and simplifications made. | 9 |
| Critical appraisal of individual sources of evidence | 12 | If done, provide a rationale for conducting a critical appraisal of included sources of evidence; describe the methods used and how this information was used in any data synthesis (if appropriate). | NA |
| Synthesis of results | 13 | Describe the methods of handling and summarizing the data that were charted. | 9-10 |
| **RESULTS** | | | |
| Selection of sources of evidence | 14 | Give numbers of sources of evidence screened, assessed for eligibility, and included in the review, with reasons for exclusions at each stage, ideally using a flow diagram. | Figure 2 |
| Characteristics of sources of evidence | 15 | For each source of evidence, present characteristics for which data were charted and provide the citations. | 10-12  Supplementary Tables 1-3 |
| Critical appraisal within sources of evidence | 16 | If done, present data on critical appraisal of included sources of evidence (see item 12). | NA |
| Results of individual sources of evidence | 17 | For each included source of evidence, present the relevant data that were charted that relate to the review questions and objectives. | Supplementary Tables 1-3 |
| Synthesis of results | 18 | Summarize and/or present the charting results as they relate to the review questions and objectives. | 12-20  Appendix G |
| **DISCUSSION** | | | |
| Summary of evidence | 19 | Summarize the main results (including an overview of concepts, themes, and types of evidence available), link to the review questions and objectives, and consider the relevance to key groups. | 20-24  Supplementary Table 8 |
| Limitations | 20 | Discuss the limitations of the scoping review process. | 24-25 |
| Conclusions | 21 | Provide a general interpretation of the results with respect to the review questions and objectives, as well as potential implications and/or next steps. | 25-26 |
| **FUNDING** | | | |
| Funding | 22 | Describe sources of funding for the included sources of evidence, as well as sources of funding for the scoping review. Describe the role of the funders of the scoping review. | 27 |

**Appendix B: Search strategies**

Ovid MEDLINE(R) ALL <1946 to February 07, 2024>

| **#** | **Searches** | **Results** |
| --- | --- | --- |
| 1 | (dyad* adj2 (study* or studie*)).ti,ab,kf. | 746 |
| 2 | (dyad* adj3 (analys* or analyz* or analytic*)).ti,ab,kf. | 1327 |
| 3 | (dyad* adj3 experience*).ti,ab,kf. | 282 |
| 4 | (dyad* adj4 perspective*).ti,ab,kf. | 274 |
| 5 | (dyad* adj4 sampl*).ti,ab,kf. | 627 |
| 6 | (dyad* adj2 approach*).ti,ab,kf. | 372 |
| 7 | (dyad* adj3 unit).ti,ab,kf. | 85 |
| 8 | (dyad* adj4 participant*).ti,ab,kf. | 783 |
| 9 | (dyad* adj3 (level or levels)).ti,ab,kf. | 618 |
| 10 | (dyad* adj7 interview*).ti,ab,kf. | 922 |
| 11 | (within adj2 dyad*).ti,ab,kf. | 741 |
| 12 | ((multiperspectiv* or multi-perspectiv* or "multiple perspective*") adj3 (analys* or analyz*)).ti,ab,kf. | 112 |
| 13 | ((multiperspectiv* or multi-perspectiv* or "multiple perspective*") adj2 (study* or studie*)).ti,ab,kf. | 150 |
| 14 | ((multiperspectiv* or multi-perspectiv* or "multiple perspective*") adj1 approach*).ti,ab,kf. | 71 |
| 15 | ((multiperspectiv* or multi-perspectiv* or "multiple perspective*") adj3 interview*).ti,ab,kf. | 31 |
| 16 | ("multi family member" adj2 (interview* or study or studies)).ti,ab,kf. | 4 |
| 17 | "couple level".ti,ab,kf. | 267 |
| 18 | ((couples or couple) adj3 interview*).ti,ab,kf. | 538 |
| 19 | ((couples or couple) adj3 experience*).ti,ab,kf. | 1009 |
| 20 | (separate* adj1 (interview* or "semi-structured interview*")).ti,ab,kf. | 568 |
| 21 | or/1-20 | 8105 |
| 22 | Caregivers/ | 51998 |
| 23 | (caregiver* or care-giver* or care giver* or caregiving or carer* or care-partner* or (care adj2 partner*) or (care adj1 giving) or (care adj2 provider*) or (support adj2 provider*) or (support adj2 partner*)).ti,ab,kf. | 200625 |
| 24 | Interpersonal Relations/ or family relations/ or family support/ or intergenerational relations/ or parent-child relations/ or father-child relations/ or mother-child relations/ | 148362 |
| 25 | ("family relation*" or "interpersonal relation*" or "intergenerational relation*" or "inter-generational relation*" or "family support*" or "family assistance" or "family encouragement" or "marital relation*" or "marital adjustment*").ti,ab,kf. | 28836 |
| 26 | Family/ or Nuclear Family/ | 91154 |
| 27 | Spouses/ | 11725 |
| 28 | parents/ or fathers/ or mothers/ or grandparents/ | 144778 |
| 29 | Siblings/ | 13563 |
| 30 | Adult children/ | 1882 |
| 31 | ("family member*" or spouse* or husband* or wife or wives or "domestic partner*" or "registered partner*" or "civil partner*" or parent* or father* or mother* or grandparent* or grand-parent* or grandmother* or grand-mother* or grandfather* or grand-father* or brother* or sister* or sibling* or daughter* or son or sons or children or child).ti,ab,kf. | 2219540 |
| 32 | or/22-31 | 2504646 |
| 33 | exp Empirical Research/ or Interviews as Topic/ or Personal Narratives as Topic/ or Focus Groups/ or exp Narration/ or Nursing Methodology Research/ or Narrative Medicine/ | 184657 |
| 34 | (Interview or Personal Narrative).pt. | 37134 |
| 35 | interview*.ti,ab,kf. | 473382 |
| 36 | qualitative.ti,ab,kf,jw. | 346596 |
| 37 | (theme* or thematic).ti,ab,kf. | 181269 |
| 38 | ethnological research.ti,ab,kf. | 8 |
| 39 | ethnograph*.ti,ab,kf. | 14515 |
| 40 | ethnomedicine.ti,ab,kf. | 1057 |
| 41 | ethnonursing.ti,ab,kf. | 130 |
| 42 | phenomenol*.ti,ab,kf. | 35610 |
| 43 | (grounded adj (theor* or study or studies or research or analys?s)).ti,ab,kf. | 15986 |
| 44 | life stor*.ti,ab,kf. | 1631 |
| 45 | (emic or etic or hermeneutic* or heuristic* or semiotic*).ti,ab,kf. | 23059 |
| 46 | (data adj1 saturat$).ti,ab,kf. | 2292 |
| 47 | participant observ*.ti,ab,kf. | 5690 |
| 48 | (social construct* or postmodern* or post-structural* or post structural* or poststructural* or post modern* or post-modern*).ti,ab,kf. | 4463 |
| 49 | (action research or cooperative inquir* or co operative inquir* or co-operative inquir*).ti,ab,kf. | 5985 |
| 50 | (humanistic or existential or experiential or paradigm*).ti,ab,kf. | 202040 |
| 51 | (field adj (study or studies or research or work)).ti,ab,kf. | 22087 |
| 52 | (human science or social science).ti,ab,kf. | 7206 |
| 53 | biographical method.ti,ab,kf. | 24 |
| 54 | theoretical sampl*.ti,ab,kf. | 967 |
| 55 | ((purpos* adj4 sampl*) or (focus adj group*)).ti,ab,kf. | 89613 |
| 56 | (open-ended or narrative* or textual or texts or semi-structured).ti,ab,kf. | 197241 |
| 57 | (life world* or life-world* or conversation analys?s or personal experience* or theoretical saturation).ti,ab,kf. | 18674 |
| 58 | ((lived or life) adj experience*).ti,ab,kf. | 20950 |
| 59 | cluster sampl*.ti,ab,kf. | 9873 |
| 60 | observational method*.ti,ab,kf. | 1023 |
| 61 | content analysis.ti,ab,kf. | 46166 |
| 62 | (constant adj (comparative or comparison)).ti,ab,kf. | 6234 |
| 63 | ((discourse* or discurs*) adj3 analys?s).ti,ab,kf. | 3424 |
| 64 | (heidegger* or colaizzi* or spiegelberg* or merleau* or husserl* or foucault* or ricoeur or glaser*).ti,ab,kf. | 5050 |
| 65 | (van adj manen*).ti,ab,kf. | 578 |
| 66 | (van adj kaam*).ti,ab,kf. | 45 |
| 67 | (corbin* adj2 strauss*).ti,ab,kf. | 466 |
| 68 | or/33-67 | 1230183 |
| 69 | 21 and 32 and 68 | 2463 |
| 70 | exp animals/ not humans.sh. | 5193858 |
| 71 | 69 not 70 | 2462 |
| 72 | limit 71 to yr="1860 - 2009" | 558 |
| 73 | limit 71 to yr="2010 -Current" | 1904 |

Embase Classic+Embase <1947 to 2024 February 07>

| **#** | **Searches** | **Results** |
| --- | --- | --- |
| 1 | (dyad* adj2 (study* or studies)).ti,ab,kf. | 714 |
| 2 | (dyad* adj3 (analys* or analyz* or analytic*)).ti,ab,kf. | 1456 |
| 3 | (dyad* adj3 experience*).ti,ab,kf. | 330 |
| 4 | (dyad* adj4 perspective*).ti,ab,kf. | 321 |
| 5 | (dyad* adj4 sampl*).ti,ab,kf. | 730 |
| 6 | (dyad* adj2 approach*).ti,ab,kf. | 420 |
| 7 | (dyad* adj3 unit).ti,ab,kf. | 96 |
| 8 | (dyad* adj4 participant*).ti,ab,kf. | 936 |
| 9 | (dyad* adj3 (level or levels)).ti,ab,kf. | 648 |
| 10 | (dyad* adj7 interview*).ti,ab,kf. | 1206 |
| 11 | (within adj2 dyad*).ti,ab,kf. | 863 |
| 12 | ((multiperspectiv* or multi-perspectiv* or "multiple perspective*") adj3 (analys* or analyz*)).ti,ab,kf. | 111 |
| 13 | ((multiperspectiv* or multi-perspectiv* or "multiple perspective*") adj2 (study* or studies)).ti,ab,kf. | 171 |
| 14 | ((multiperspectiv* or multi-perspectiv* or "multiple perspective*") adj1 approach*).ti,ab,kf. | 90 |
| 15 | ((multiperspectiv* or multi-perspectiv* or "multiple perspective*") adj3 interview*).ti,ab,kf. | 39 |
| 16 | ("multi family member" adj2 (interview* or study or studies)).ti,ab,kf. | 6 |
| 17 | "couple level".ti,ab,kf. | 275 |
| 18 | ((couples or couple) adj3 interview*).ti,ab,kf. | 708 |
| 19 | ((couples or couple) adj3 experience*).ti,ab,kf. | 1344 |
| 20 | (separate* adj1 (interview* or "semi-structured interview?")).ti,ab,kf. | 809 |
| 21 | or/1-20 | 9618 |
| 22 | caregiver/ | 118942 |
| 23 | care behavior/ or "care and caring"/ | 11935 |
| 24 | (caregiver* or care-giver* or care giver* or caregiving or carer* or care-partner* or (care adj2 partner*) or (care adj1 giving) or (care adj2 provider*) or (support adj2 provider*) or (support adj2 partner*)).ti,ab,kf. | 275389 |
| 25 | family relation/ or family support/ or human relation/ or child parent relation/ or father child relation/ or mother child relation/ | 207026 |
| 26 | ("family relation*" or "interpersonal relation*" or "intergenerational relation*" or "inter-generational relation*" or "family support*" or "family assistance" or "family encouragement" or "marital relation*" or "marital adjustment*").ti,ab,kf. | 35170 |
| 27 | family/ or nuclear family/ | 112916 |
| 28 | spouse/ or domestic partner/ or husband/ or wife/ | 26087 |
| 29 | parent/ or father/ or mother/ or grandparent/ or grandmother/ or grandfather/ | 257709 |
| 30 | sibling/ or brother/ or sister/ | 60358 |
| 31 | adult child/ | 2148 |
| 32 | daughter/ or son/ | 11662 |
| 33 | ("family member*" or spouse* or husband* or wife or wives or "domestic partner*" or "registered partner*" or "civil partner*" or parent* or father* or mother* or grandparent* or grand-parent* or grandmother* or grand-mother* or grandfather* or grand-father* or brother* or sister* or sibling* or daughter* or son or sons or children or child).ti,ab,kf. | 3089353 |
| 34 | or/22-33 | 3475501 |
| 35 | exp empirical research/ or exp interview/ or narrative/ or nursing methodology research/ or narrative medicine/ or exp qualitative research/ or storytelling/ | 498018 |
| 36 | interview*.ti,ab,kf. | 601380 |
| 37 | qualitative.ti,ab,kf,jx. | 434542 |
| 38 | (theme* or thematic).ti,ab,kf. | 226163 |
| 39 | ethnological research.ti,ab,kf. | 12 |
| 40 | ethnograph*.ti,ab,kf. | 15941 |
| 41 | ethnomedicine.ti,ab,kf. | 1724 |
| 42 | ethnonursing.ti,ab,kf. | 132 |
| 43 | phenomenol*.ti,ab,kf. | 40376 |
| 44 | (grounded adj (theor* or study or studies or research or analys?s)).ti,ab,kf. | 19723 |
| 45 | life stor*.ti,ab,kf. | 2050 |
| 46 | (emic or etic or hermeneutic* or heuristic* or semiotic*).ti,ab,kf. | 25306 |
| 47 | (data adj1 saturat$).ti,ab,kf. | 2989 |
| 48 | participant observ*.ti,ab,kf. | 6405 |
| 49 | (social construct* or postmodern* or post-structural* or post structural* or poststructural* or post modern* or post-modern*).ti,ab,kf. | 5381 |
| 50 | (action research or cooperative inquir* or co operative inquir* or co-operative inquir*).ti,ab,kf. | 7113 |
| 51 | (humanistic or existential or experiential or paradigm*).ti,ab,kf. | 251817 |
| 52 | (field adj (study or studies or research or work)).ti,ab,kf. | 25865 |
| 53 | (human science or social science).ti,ab,kf. | 8101 |
| 54 | biographical method.ti,ab,kf. | 35 |
| 55 | theoretical sampl*.ti,ab,kf. | 1102 |
| 56 | ((purpos* adj4 sampl*) or (focus adj group*)).ti,ab,kf. | 113830 |
| 57 | (open-ended or narrative* or textual or texts or semi-structured).ti,ab,kf. | 238411 |
| 58 | (life world* or life-world* or conversation analys?s or personal experience* or theoretical saturation).ti,ab,kf. | 29356 |
| 59 | ((lived or life) adj experience*).ti,ab,kf. | 26948 |
| 60 | cluster sampl*.ti,ab,kf. | 12107 |
| 61 | observational method*.ti,ab,kf. | 1398 |
| 62 | content analysis.ti,ab,kf. | 52163 |
| 63 | (constant adj (comparative or comparison)).ti,ab,kf. | 7789 |
| 64 | ((discourse* or discurs*) adj3 analys?s).ti,ab,kf. | 3879 |
| 65 | (heidegger* or colaizzi* or spiegelberg* or merleau* or husserl* or foucault* or ricoeur or glaser*).ti,ab,kf. | 5664 |
| 66 | (van adj manen*).ti,ab,kf. | 661 |
| 67 | (van adj kaam*).ti,ab,kf. | 50 |
| 68 | (corbin* adj2 strauss*).ti,ab,kf. | 511 |
| 69 | or/35-68 | 1581548 |
| 70 | 21 and 34 and 69 | 3183 |
| 71 | (exp animal/ or animal experiment/ or nonhuman/) not (exp human/ or human experiment/) | 8156056 |
| 72 | 70 not 71 | 3180 |
| 73 | limit 72 to yr="1883 - 2009" | 634 |
| 74 | limit 72 to yr="2010 -Current" | 2541 |
| 75 | 73 or 74 | 3175 |
| 76 | 72 not 75 | 5 |

APA PsycInfo <1806 to February Week 2 2024>

| **#** | **Searches** | **Results** |
| --- | --- | --- |
| 1 | Dyads/ | 9050 |
| 2 | (dyad* adj2 (study* or studie*)).ti,ab,id. | 1084 |
| 3 | (dyad* adj3 (analys* or analyz* or analytic*)).ti,ab,id. | 2356 |
| 4 | (dyad* adj3 experience*).ti,ab,id. | 452 |
| 5 | (dyad* adj4 perspective*).ti,ab,id. | 572 |
| 6 | (dyad* adj4 sampl*).ti,ab,id. | 798 |
| 7 | (dyad* adj2 approach*).ti,ab,id. | 519 |
| 8 | (dyad* adj3 unit).ti,ab,id. | 84 |
| 9 | (dyad* adj4 participant*).ti,ab,id. | 972 |
| 10 | (dyad* adj3 (level or levels)).ti,ab,id. | 1239 |
| 11 | (dyad* adj7 interview*).ti,ab,id. | 1088 |
| 12 | (within adj2 dyad*).ti,ab,id. | 1212 |
| 13 | ((multiperspectiv* or multi-perspectiv* or "multiple perspective*") adj3 (analys* or analyz*)).ti,ab,id. | 126 |
| 14 | ((multiperspectiv* or multi-perspectiv* or "multiple perspective*") adj2 (study* or studie*)).ti,ab,id. | 117 |
| 15 | ((multiperspectiv* or multi-perspectiv* or "multiple perspective*") adj1 approach*).ti,ab,id. | 141 |
| 16 | ((multiperspectiv* or multi-perspectiv* or "multiple perspective*") adj3 interview*).ti,ab,id. | 33 |
| 17 | ("multi family member" adj2 (interview* or study or studies)).ti,ab,id. | 6 |
| 18 | couples/ | 17107 |
| 19 | "couple level".ti,ab,id. | 360 |
| 20 | ((couples or couple) adj3 interview*).ti,ab,id. | 1054 |
| 21 | ((couples or couple) adj3 experience*).ti,ab,id. | 1783 |
| 22 | (separate* adj1 (interview* or "semi-structured interview*")).ti,ab,id. | 673 |
| 23 | or/1-22 | 33609 |
| 24 | caregivers/ | 38350 |
| 25 | caregiving/ | 8401 |
| 26 | partners/ | 6667 |
| 27 | (caregiver* or care-giver* or care giver* or caregiving or carer* or care-partner* or (care adj2 partner*) or (care adj1 giving) or (care adj2 provider*) or (support adj2 provider*) or (support adj2 partner*)).ti,ab,id. | 109562 |
| 28 | family relations/ or interpersonal relationships/ or marital relations/ or intergenerational relations/ or parent-child relations/ or father child relations/ or mother child relations/ | 134083 |
| 29 | ("family relation*" or "interpersonal relation*" or "intergenerational relation*" or "inter-generational relation*" or "family support*" or "family assistance" or "family encouragement" or "marital relation*" or "marital adjustment*").ti,ab,id. | 51195 |
| 30 | family/ or family members/ or nuclear family/ | 82825 |
| 31 | spouses/ or husbands/ or wives/ | 17176 |
| 32 | parents/ or fathers/ or mothers/ or grandparents/ | 104978 |
| 33 | siblings/ or brothers/ or sisters/ | 9055 |
| 34 | adult offspring/ | 4655 |
| 35 | daughters/ or sons/ | 5194 |
| 36 | ("family member*" or spouse* or husband* or wife or wives or "domestic partner*" or "registered partner*" or "civil partner*" or parent* or father* or mother* or grandparent* or grand-parent* or grandmother* or grand-mother* or grandfather* or grand-father* brother* or sister* or sibling* or daughter* or son or sons or children or child).ti,ab,id. | 957126 |
| 37 | or/24-36 | 1098813 |
| 38 | Empirical Methods/ or exp Qualitative Methods/ or exp Interviews/ or Interviewing/ or Narratives/ or Storytelling/ | 74906 |
| 39 | interview*.ti,ab,id. | 403332 |
| 40 | qualitative.ti,ab,jx,id. | 233104 |
| 41 | (theme* or thematic).ti,ab,id. | 178501 |
| 42 | ethnological research.ti,ab,id. | 13 |
| 43 | ethnograph*.ti,ab,id. | 33904 |
| 44 | ethnomedicine.ti,ab,id. | 61 |
| 45 | ethnonursing.ti,ab,id. | 75 |
| 46 | phenomenol*.ti,ab,id. | 55310 |
| 47 | (grounded adj (theor* or study or studies or research or analys?s)).ti,ab,id. | 20674 |
| 48 | life stor*.ti,ab,id. | 4344 |
| 49 | (emic or etic or hermeneutic* or heuristic* or semiotic*).ti,ab,id. | 31760 |
| 50 | (data adj1 saturat$).ti,ab,id. | 586 |
| 51 | participant observ*.ti,ab,id. | 10162 |
| 52 | (social construct* or postmodern* or post-structural* or post structural* or poststructural* or post modern* or post-modern*).ti,ab,id. | 20387 |
| 53 | (action research or cooperative inquir* or co operative inquir* or co-operative inquir*).ti,ab,id. | 10758 |
| 54 | (humanistic or existential or experiential or paradigm*).ti,ab,id. | 156157 |
| 55 | (field adj (study or studies or research or work)).ti,ab,id. | 12850 |
| 56 | (human science or social science).ti,ab,id. | 13928 |
| 57 | biographical method.ti,ab,id. | 64 |
| 58 | theoretical sampl*.ti,ab,id. | 721 |
| 59 | ((purpos* adj4 sampl*) or (focus adj group*)).ti,ab,id. | 60446 |
| 60 | (open-ended or narrative* or textual or texts or semi-structured).ti,ab,id. | 191332 |
| 61 | (life world* or life-world* or conversation analys?s or personal experience* or theoretical saturation).ti,ab,id. | 18050 |
| 62 | ((lived or life) adj experience*).ti,ab,id. | 38590 |
| 63 | cluster sampl*.ti,ab,id. | 2154 |
| 64 | observational method*.ti,ab,id. | 1237 |
| 65 | content analysis.ti,ab,id. | 33702 |
| 66 | (constant adj (comparative or comparison)).ti,ab,id. | 5838 |
| 67 | ((discourse* or discurs*) adj3 analys?s).ti,ab,id. | 11207 |
| 68 | (heidegger* or colaizzi* or spiegelberg* or merleau* or husserl* or foucault* or ricoeur or glaser*).ti,ab,id. | 9692 |
| 69 | (van adj manen*).ti,ab,id. | 663 |
| 70 | (van adj kaam*).ti,ab,id. | 555 |
| 71 | (corbin* adj2 strauss*).ti,ab,id. | 788 |
| 72 | or/38-71 | 975430 |
| 73 | 23 and 37 and 72 | 5567 |
| 74 | exp animals/ not humans.sh. | 380842 |
| 75 | 73 not 74 | 5556 |
| 76 | limit 75 to yr="1860 - 2009" | 2069 |
| 77 | limit 75 to yr="2010 -Current" | 3486 |
| 78 | 76 or 77 | 5555 |
| 79 | 75 not 78 | 1 |

CINAHL

|  | Thursday, February 08, 2024 6:08:00 PM |
| --- | --- |
| Interface - EBSCOhost Research Databases  Search Screen - Advanced Search  Database - CINAHL Plus with Full Text |  |

| **#** | **Query** | **Limiters/Expanders** | **Results** |
| --- | --- | --- | --- |
| S32 | S21 AND S28 AND S29 | Limiters - Publication Date: 20100101-20241231  Search modes - Boolean/Phrase | 1,961 |
| S31 | S21 AND S28 AND S29 | Limiters - Publication Date: 19800101-20091231  Search modes - Boolean/Phrase | 528 |
| S30 | S21 AND S28 AND S29 | Search modes - Boolean/Phrase | 2,489 |
| S29 | MH Qualitative Studies OR MH Grounded theory OR MH Narratives OR MH Interviews+ OR MH Audiorecording OR MH Focus Groups OR MH Research, Nursing OR MH Discourse Analysis OR MH Content Analysis OR MH Ethnographic Research OR MH Ethnonursing Research OR MH Constant Comparative Method OR MH Qualitative Validity+ OR MH Purposive Sample OR MH Observational Methods+ OR MH Field Studies OR MH Theoretical Sample OR MH Phenomenology OR MH Phenomenological Research OR MH Life Experiences+ OR MH Cluster Sample+ OR TI qualitative OR AB qualitative OR TI interview* OR AB interview* OR TI (theme* or thematic) OR AB (theme* or thematic) OR TI ("ethnological research") OR AB ("ethnological research") OR TI ethnonursing OR AB ethnonursing OR TI ethnograph* OR AB ethnograph* OR TI phenomenol* OR AB phenomenol* OR TI "focus group*" OR AB "focus group*" OR TI (grounded N1 (theor* OR analys?s OR research OR studies OR study)) OR AB (grounded N1 (theor* OR analys?s OR research OR studies OR study)) OR TI ("life stor*") OR AB ("life stor*") OR TI (emic OR etic OR hermeneutic* OR heuristic* OR semiotic) OR AB (emic OR etic OR hermeneutic* OR heuristic* OR semiotic) OR TI (data N1 saturat*) OR AB (data N1 saturat*) OR TI ("participant observ*") OR AB ("participant observ*") OR TI ("social construct*" OR postmodern* OR "post-structural*" OR poststructural* OR "post-modern*" OR feminis*) OR AB ("social construct*" OR postmodern* OR "post-structural*" OR poststructural* OR "post-modern*" OR feminis*) OR TI ("action research" OR "cooperative inquir*" OR "co-operative inquir*") OR AB ("action research" OR "cooperative inquir*" OR "co-operative inquir*") OR TI (humanistic OR existential OR experiential OR paradigm*) OR AB (humanistic OR existential OR experiential OR paradigm*) OR TI (field N1 (research OR study OR studies)) OR AB (field N1 (research OR study OR studies)) OR TI "human science" OR AB "human science" OR TI "biographical method" OR AB "biographical method" OR TI ("theoretical sampl*") OR AB ("theoretical sampl*") OR TI ("purpos* N4 sampl*") OR AB ("purpos* N4 sampl*") OR TI ("open-ended" OR narrative* OR textual OR texts OR "semi-structured") OR AB ("open-ended" OR narrative* OR textual OR texts OR "semi-structured") OR TI ("life world" OR "life-world" OR "conversation analys?s" OR "personal experience*" OR "theoretical saturation") OR AB ("life world" OR "life-world" OR "conversation analys?s" OR "personal experience*" OR "theoretical saturation") OR TI ((life OR lived) N1 experience*) OR AB ((life OR lived) N1 experience*) OR TI ("cluster sampl*") OR AB ("cluster sampl*") OR TI ("observational method*") OR AB ("observational method*") OR TI ("content analysis") OR AB ("content analysis") OR TI ((discurs* OR discourse*) N3 analys?s) OR AB ((discurs* OR discourse*) N3 analys?s) OR TI (constant N1 (comparison OR comparative)) OR AB (constant N1 (comparison OR comparative)) OR TI ("narrative analys?s") OR AB ("narrative analys?s") OR TI (heidegger* OR colaizzi* OR spiegelberg* OR merleau* OR husserl* OR foucault* OR ricoeur OR glaser*) OR AB (heidegger* OR colaizzi* OR spiegelberg* OR merleau* OR husserl* OR foucault* OR ricoeur OR glaser*) OR TI (van N1 manen*) OR AB (van N1 manen*) OR TI (van N1 kaam*) OR AB (van N1 kaam*) OR TI (Corbin* N2 strauss*) OR AB (Corbin* N2 strauss*) | Search modes - Boolean/Phrase | 759,192 |
| S28 | S22 OR S23 OR S24 OR S25 OR S26 OR S27 | Search modes - Boolean/Phrase | 960,595 |
| S27 | TI("family member*" or spouse* or husband* or wife or wives or "domestic partner*" or "registered partner*" or "civil partner*" or parent* or father* or mother* or grandparent* or grand-parent* or grandmother* or grand-mother* or grandfather* or grand-father* brother* or sister* or sibling* or daughter* or son or sons or children or child) OR AB ("family member*" or spouse* or husband* or wife or wives or "domestic partner*" or "registered partner*" or "civil partner*" or parent* or father* or mother* or grandparent* or grand-parent* or grandmother* or grand-mother* or grandfather* or grand-father* brother* or sister* or sibling* or daughter* or son or sons or children or child) | Search modes - Boolean/Phrase | 746,429 |
| S26 | (MH "Family") OR (MH "Adult Children") OR (MH "Nuclear Family") OR (MH "Daughters") OR (MH "Parents") OR (MH "Fathers") OR (MH "Mothers") OR (MH "Grandparents") OR (MH "Siblings") OR (MH "Sons") OR (MH "Spouses") | Search modes - Boolean/Phrase | 158,907 |
| S25 | TI("family relation*" or "interpersonal relation*" or "intergenerational relation*" or "inter-generational relation*" or "family support*" or "family assistance" or "family encouragement" or "marital relation*" or "marital adjustment*") OR AB("family relation*" or "interpersonal relation*" or "intergenerational relation*" or "inter-generational relation*" or "family support*" or "family assistance" or "family encouragement" or "marital relation*" or "marital adjustment*") | Search modes - Boolean/Phrase | 16,037 |
| S24 | (MH "Family Relations") OR (MH "Family Support") OR (MH "Interpersonal Relations") OR (MH "Intergenerational Relations") OR (MH "Parent-Child Relations") OR (MH "Father-Child Relations") OR (MH "Mother-Child Relations") | Search modes - Boolean/Phrase | 118,801 |
| S23 | TI(caregiver* or care-giver* or "care giver*" or caregiving or carer* or care-partner* or (care N2 partner*) or (care N1 giving) or (care N2 provider*) or (support N2 provider*) or (support N2 partner*)) OR AB(caregiver* or care-giver* or "care giver*" or caregiving or carer* or care-partner* or (care N2 partner*) or (care N1 giving) or (care N2 provider*) or (support N2 provider*) or (support N2 partner*)) | Search modes - Boolean/Phrase | 143,901 |
| S22 | (MH "Caregivers") | Search modes - Boolean/Phrase | 44,404 |
| S21 | S1 OR S2 OR S3 OR S4 OR S5 OR S6 OR S7 OR S8 OR S9 OR S10 OR S11 OR S12 OR S13 OR S14 OR S15 OR S16 OR S17 OR S18 OR S19 OR S20 | Search modes - Boolean/Phrase | 6,465 |
| S20 | TI (separate* N1 (interview* or "semi-structured interview*")) OR AB (separate* N1 (interview* or "semi-structured interview*")) | Search modes - Boolean/Phrase | 452 |
| S19 | TI ((couples or couple) N3 experience*) OR AB ((couples or couple) N3 experience*) | Search modes - Boolean/Phrase | 912 |
| S18 | TI ((couples or couple) N3 interview*) OR AB ((couples or couple) N3 interview*) | Search modes - Boolean/Phrase | 521 |
| S17 | TI ("couple level") OR AB ("couple level") | Search modes - Boolean/Phrase | 157 |
| S16 | TI ("multi family member" N2 (interview* or study or studies)) OR AB ("multi family member" N2 (interview* or study or studies)) | Search modes - Boolean/Phrase | 5 |
| S15 | TI ((multiperspectiv* or multi-perspectiv* or "multiple perspective*") N3 interview*) OR AB ((multiperspectiv* or multi-perspectiv* or "multiple perspective*") N3 interview*) | Search modes - Boolean/Phrase | 21 |
| S14 | TI ((multiperspectiv* or multi-perspectiv* or "multiple perspective*") N1 approach*) OR AB ((multiperspectiv* or multi-perspectiv* or "multiple perspective*") N1 approach*) | Search modes - Boolean/Phrase | 47 |
| S13 | TI ((multiperspectiv* or multi-perspectiv* or "multiple perspective*") N2 (study* or studie*)) OR AB ((multiperspectiv* or multi-perspectiv* or "multiple perspective*") N2 (study* or studie*)) | Search modes - Boolean/Phrase | 120 |
| S12 | TI ((multiperspectiv* or multi-perspectiv* or "multiple perspective*") N3 (analys* or analyz*)) OR AB ((multiperspectiv* or multi-perspectiv* or "multiple perspective*") N3 (analys* or analyz*)) | Search modes - Boolean/Phrase | 53 |
| S11 | TI (within N2 dyad*) OR AB (within N2 dyad*) | Search modes - Boolean/Phrase | 486 |
| S10 | TI (dyad* N7 interview*) OR AB (dyad* N7 interview*) | Search modes - Boolean/Phrase | 757 |
| S9 | TI (dyad* N3 (level or levels)) OR AB (dyad* N3 (level or levels)) | Search modes - Boolean/Phrase | 407 |
| S8 | TI (dyad* N4 participant*) OR AB (dyad* N4 participant*) | Search modes - Boolean/Phrase | 682 |
| S7 | TI (dyad* N3 unit) OR AB (dyad* N3 unit) | Search modes - Boolean/Phrase | 69 |
| S6 | TI (dyad* N2 approach*) OR AB (dyad* N2 approach*) | Search modes - Boolean/Phrase | 288 |
| S5 | TI (dyad* N4 sampl*) OR AB (dyad* N4 sampl*) | Search modes - Boolean/Phrase | 766 |
| S4 | TI (dyad* N4 perspective*) OR AB (dyad* N4 perspective*) | Search modes - Boolean/Phrase | 228 |
| S3 | TI (dyad* N3 experience*) OR AB (dyad* N3 experience*) | Search modes - Boolean/Phrase | 244 |
| S2 | TI (dyad* N3 (analys* or analyz* or analytic*)) OR AB dyad* N3 (analys* or analyz* or analytic*)) | Search modes - Boolean/Phrase | 950 |
| S1 | TI (dyad* N2 (study* or studie*)) OR AB (dyad* N2 (study* or studie*)) | Search modes - Boolean/Phrase | 768 |

Sociological abstracts

| **Select all** | [**Set**](https://www.proquest.com/recentsearches.recentsearchtabview.recentsearchesgridview:toggellistorder?site=sociologicalabstracts&t:ac=RecentSearches) | | **Search** | | **Results** | |
| --- | --- | --- | --- | --- | --- | --- |
| Select item 48 | **S48** | | [[S23] AND [S40] AND [S44]](https://www.proquest.com/recentsearches.recentsearchtabview.recentsearchesgridview.scrolledrecentsearchlist.checkdbssearchlink:rerunsearch/4C66D2A6E7E54C66PQ/None/$N?site=sociologicalabstracts&t:ac=RecentSearches)Limits applied  *2010-2029 | | [**2,596**](https://www.proquest.com/recentsearches.recentsearchtabview.recentsearchesgridview.scrolledrecentsearchlist.checkdbssearchlink_0:rerunsearch/4C66D2A6E7E54C66PQ/None/$N?site=sociologicalabstracts&t:ac=RecentSearches) | |
| Select item 47 | **S47** | | [[S23] AND [S40] AND [S44]](https://www.proquest.com/recentsearches.recentsearchtabview.recentsearchesgridview.scrolledrecentsearchlist.checkdbssearchlink:rerunsearch/A5C98249E675468CPQ/None/$N?site=sociologicalabstracts&t:ac=RecentSearches) | | [**4,114**](https://www.proquest.com/recentsearches.recentsearchtabview.recentsearchesgridview.scrolledrecentsearchlist.checkdbssearchlink_0:rerunsearch/A5C98249E675468CPQ/None/$N?site=sociologicalabstracts&t:ac=RecentSearches) | |
| Select item 46 | **S46** | | [[S23] AND [S40] AND [S44]](https://www.proquest.com/recentsearches.recentsearchtabview.recentsearchesgridview.scrolledrecentsearchlist.checkdbssearchlink:rerunsearch/1CF6D942BB834938PQ/None/$N?site=sociologicalabstracts&t:ac=RecentSearches)Limits applied  *1950-2009 | | [**1,518**](https://www.proquest.com/recentsearches.recentsearchtabview.recentsearchesgridview.scrolledrecentsearchlist.checkdbssearchlink_0:rerunsearch/1CF6D942BB834938PQ/None/$N?site=sociologicalabstracts&t:ac=RecentSearches) | |
| Select item 45 | **S45** | | [[S23] AND [S40] AND [S44]](https://www.proquest.com/recentsearches.recentsearchtabview.recentsearchesgridview.scrolledrecentsearchlist.checkdbssearchlink:rerunsearch/9C5AEBD7F6045B5PQ/None/$N?site=sociologicalabstracts&t:ac=RecentSearches) | | [**4,114**](https://www.proquest.com/recentsearches.recentsearchtabview.recentsearchesgridview.scrolledrecentsearchlist.checkdbssearchlink_0:rerunsearch/9C5AEBD7F6045B5PQ/None/$N?site=sociologicalabstracts&t:ac=RecentSearches) | |
| Select item 44 | **S44** | | [[S41] OR [S42] OR [S43]](https://www.proquest.com/recentsearches.recentsearchtabview.recentsearchesgridview.scrolledrecentsearchlist.checkdbssearchlink:rerunsearch/776578BC13C949D8PQ/None/$N?site=sociologicalabstracts&t:ac=RecentSearches) | | [**680,337**](https://www.proquest.com/recentsearches.recentsearchtabview.recentsearchesgridview.scrolledrecentsearchlist.checkdbssearchlink_0:rerunsearch/776578BC13C949D8PQ/None/$N?site=sociologicalabstracts&t:ac=RecentSearches) | |
| Select item 43 | **S43** | | [noft(qualitative OR interview* OR theme* OR thematic OR ("ethnological research") OR ethnonursing OR ethnograph* OR phenomenol* OR (("focus group" OR "focus groups")) OR (grounded NEAR/1 (theor* OR analys?s OR research OR studies OR study)) OR (("life stories" OR "life story")) OR emic OR etic OR hermeneutic* OR heuristic* OR semiotic OR (data NEAR/1 saturat*) OR (("participant observation" OR "participant observer")) OR (("social construct" OR "social construction" OR "social constructionism" OR "social constructionist" OR "social constructionists" OR "social constructions" OR "social constructive" OR "social constructivism" OR "social constructivist" OR "social constructivists" OR "social constructs")) OR postmodern* OR ("post-structural*") OR poststructural* OR ("post-modern*") OR feminis* OR ("action research") OR ("cooperative inquir*") OR ("co-operative inquir*") OR humanistic OR existential OR experiential OR paradigm* OR (field NEAR/1 (research OR study OR studies)) OR ("human science") OR ("biographical method") OR AB "biographical method" OR ("theoretical sampl*") OR ("purpos* NEAR/4 sampl*") OR ("open-ended") OR narrative* OR textual OR texts OR ("semi-structured") OR ("life world") OR ("life-world") OR ("conversation analys?s") OR (("personal experience" OR "personal experiences")) OR ("theoretical saturation") OR ((life OR lived) NEAR/1 experience*) OR (("cluster sampling")) OR (("observational method" OR "observational methods")) OR ("content analysis") OR ((discurs* OR discourse*) NEAR/3 analys?s) OR (constant NEAR/1 (comparison OR comparative)) OR ("narrative analys?s") OR heidegger* OR colaizzi* OR spiegelberg* OR merleau* OR husserl* OR foucault* OR ricoeur OR glaser* OR (van NEAR/1 manen*) OR (van NEAR/1 kaam*) OR (Corbin* NEAR/2 strauss*))](https://www.proquest.com/recentsearches.recentsearchtabview.recentsearchesgridview.scrolledrecentsearchlist.checkdbssearchlink:rerunsearch/B378BE847A3F4823PQ/None/$N?site=sociologicalabstracts&t:ac=RecentSearches) | | [**668,141**](https://www.proquest.com/recentsearches.recentsearchtabview.recentsearchesgridview.scrolledrecentsearchlist.checkdbssearchlink_0:rerunsearch/B378BE847A3F4823PQ/None/$N?site=sociologicalabstracts&t:ac=RecentSearches) | |
| Select item 42 | **S42** | | [MAINSUBJECT.EXACT.EXPLODE("Interviews")](https://www.proquest.com/recentsearches.recentsearchtabview.recentsearchesgridview.scrolledrecentsearchlist.checkdbssearchlink:rerunsearch/E1205CF7CD184361PQ/None/$N?site=sociologicalabstracts&t:ac=RecentSearches) | | [**14,580**](https://www.proquest.com/recentsearches.recentsearchtabview.recentsearchesgridview.scrolledrecentsearchlist.checkdbssearchlink_0:rerunsearch/E1205CF7CD184361PQ/None/$N?site=sociologicalabstracts&t:ac=RecentSearches) | |
| Select item 41 | **S41** | | [MAINSUBJECT.EXACT("Qualitative research" OR "Grounded Theory" OR "Narratives" OR "Focus groups" OR "Discourse analysis" or "Content analysis" OR "Ethnography" OR "Autoethnography" OR "Ethnomethodology" or "Observational studies" OR "Fieldwork" OR "Phenomenology" OR "Interpretive sociology" OR "Life history" OR "Storytelling" OR "Research Methodology")](https://www.proquest.com/recentsearches.recentsearchtabview.recentsearchesgridview.scrolledrecentsearchlist.checkdbssearchlink:rerunsearch/D30C7131B12B4603PQ/None/$N?site=sociologicalabstracts&t:ac=RecentSearches) | | [**126,119**](https://www.proquest.com/recentsearches.recentsearchtabview.recentsearchesgridview.scrolledrecentsearchlist.checkdbssearchlink_0:rerunsearch/D30C7131B12B4603PQ/None/$N?site=sociologicalabstracts&t:ac=RecentSearches) | |
| Select item 40 | **S40** | | [[S24] OR [S25] OR [S26] OR [S27] OR [S28] OR [S29] OR [S30] OR [S31] OR [S32] OR [S33] OR [S34] OR [S35] OR [S36] OR [S37] OR [S38] OR [S39]](https://www.proquest.com/recentsearches.recentsearchtabview.recentsearchesgridview.scrolledrecentsearchlist.checkdbssearchlink:rerunsearch/714422AFB4FC480FPQ/None/$N?site=sociologicalabstracts&t:ac=RecentSearches) | | [**458,831**](https://www.proquest.com/recentsearches.recentsearchtabview.recentsearchesgridview.scrolledrecentsearchlist.checkdbssearchlink_0:rerunsearch/714422AFB4FC480FPQ/None/$N?site=sociologicalabstracts&t:ac=RecentSearches) | |
| Select item 39 | **S39** | | [noft("family member*" or spouse* or husband* or wife or wives or "domestic partner*" or "registered partner*" or "civil partner*" or parent* or father* or mother* or grandparent* or grand-parent* or grandmother* or grand-mother* or grandfather* or grand-father* or brother* or sister* or sibling* or daughter* or son or sons or children or child)](https://www.proquest.com/recentsearches.recentsearchtabview.recentsearchesgridview.scrolledrecentsearchlist.checkdbssearchlink:rerunsearch/6799EB4A9E3E4F8BPQ/None/$N?site=sociologicalabstracts&t:ac=RecentSearches) | | [**414,521**](https://www.proquest.com/recentsearches.recentsearchtabview.recentsearchesgridview.scrolledrecentsearchlist.checkdbssearchlink_0:rerunsearch/6799EB4A9E3E4F8BPQ/None/$N?site=sociologicalabstracts&t:ac=RecentSearches) | |
| Select item 38 | **S38** | | [MAINSUBJECT.EXACT("Adult children")](https://www.proquest.com/recentsearches.recentsearchtabview.recentsearchesgridview.scrolledrecentsearchlist.checkdbssearchlink:rerunsearch/12A16307AA4748EBPQ/None/$N?site=sociologicalabstracts&t:ac=RecentSearches) | | [**4,486**](https://www.proquest.com/recentsearches.recentsearchtabview.recentsearchesgridview.scrolledrecentsearchlist.checkdbssearchlink_0:rerunsearch/12A16307AA4748EBPQ/None/$N?site=sociologicalabstracts&t:ac=RecentSearches) | |
| Select item 37 | **S37** | | [MAINSUBJECT.EXACT("Siblings") OR MAINSUBJECT.EXACT("Daughters") OR MAINSUBJECT.EXACT("Sons")](https://www.proquest.com/recentsearches.recentsearchtabview.recentsearchesgridview.scrolledrecentsearchlist.checkdbssearchlink:rerunsearch/90967E8E6C3C4E26PQ/None/$N?site=sociologicalabstracts&t:ac=RecentSearches) | | [**7,125**](https://www.proquest.com/recentsearches.recentsearchtabview.recentsearchesgridview.scrolledrecentsearchlist.checkdbssearchlink_0:rerunsearch/90967E8E6C3C4E26PQ/None/$N?site=sociologicalabstracts&t:ac=RecentSearches) | |
| Select item 36 | **S36** | | [MAINSUBJECT.EXACT("Parents & parenting") OR MAINSUBJECT.EXACT("Mothers") OR MAINSUBJECT.EXACT("Fathers") OR MAINSUBJECT.EXACT("Grandparents")](https://www.proquest.com/recentsearches.recentsearchtabview.recentsearchesgridview.scrolledrecentsearchlist.checkdbssearchlink:rerunsearch/D92DDAEAAD504088PQ/None/$N?site=sociologicalabstracts&t:ac=RecentSearches) | | [**70,497**](https://www.proquest.com/recentsearches.recentsearchtabview.recentsearchesgridview.scrolledrecentsearchlist.checkdbssearchlink_0:rerunsearch/D92DDAEAAD504088PQ/None/$N?site=sociologicalabstracts&t:ac=RecentSearches) | |
| Select item 35 | **S35** | | [MAINSUBJECT.EXACT("Spouses") OR MAINSUBJECT.EXACT("Husbands") OR MAINSUBJECT.EXACT("Wives")](https://www.proquest.com/recentsearches.recentsearchtabview.recentsearchesgridview.scrolledrecentsearchlist.checkdbssearchlink:rerunsearch/FBC6DE92203941FEPQ/None/$N?site=sociologicalabstracts&t:ac=RecentSearches) | | [**10,960**](https://www.proquest.com/recentsearches.recentsearchtabview.recentsearchesgridview.scrolledrecentsearchlist.checkdbssearchlink_0:rerunsearch/FBC6DE92203941FEPQ/None/$N?site=sociologicalabstracts&t:ac=RecentSearches) | |
| Select item 34 | **S34** | | [noft("family relation*" or "interpersonal relation*" or "intergenerational relation*" “inter-generational relation*” or "family support*" or "family assistance" or "family encouragement" or "marital relation*" or "marital adjustment*")](https://www.proquest.com/recentsearches.recentsearchtabview.recentsearchesgridview.scrolledrecentsearchlist.checkdbssearchlink:rerunsearch/1148B4F543BD4A65PQ/None/$N?site=sociologicalabstracts&t:ac=RecentSearches) | | [**48,984**](https://www.proquest.com/recentsearches.recentsearchtabview.recentsearchesgridview.scrolledrecentsearchlist.checkdbssearchlink_0:rerunsearch/1148B4F543BD4A65PQ/None/$N?site=sociologicalabstracts&t:ac=RecentSearches) | |
| Select item 33 | **S33** | | [MAINSUBJECT.EXACT("Intergenerational relationships")](https://www.proquest.com/recentsearches.recentsearchtabview.recentsearchesgridview.scrolledrecentsearchlist.checkdbssearchlink:rerunsearch/898181333F71403EPQ/None/$N?site=sociologicalabstracts&t:ac=RecentSearches) | | [**3,793**](https://www.proquest.com/recentsearches.recentsearchtabview.recentsearchesgridview.scrolledrecentsearchlist.checkdbssearchlink_0:rerunsearch/898181333F71403EPQ/None/$N?site=sociologicalabstracts&t:ac=RecentSearches) | |
| Select item 32 | **S32** | | [MAINSUBJECT.EXACT("Parent-child relations")](https://www.proquest.com/recentsearches.recentsearchtabview.recentsearchesgridview.scrolledrecentsearchlist.checkdbssearchlink:rerunsearch/6CEC0B9621784820PQ/None/$N?site=sociologicalabstracts&t:ac=RecentSearches) | | [**15,548**](https://www.proquest.com/recentsearches.recentsearchtabview.recentsearchesgridview.scrolledrecentsearchlist.checkdbssearchlink_0:rerunsearch/6CEC0B9621784820PQ/None/$N?site=sociologicalabstracts&t:ac=RecentSearches) | |
| Select item 31 | **S31** | | [MAINSUBJECT.EXACT("Marital adjustment") OR MAINSUBJECT.EXACT("Marital relations")](https://www.proquest.com/recentsearches.recentsearchtabview.recentsearchesgridview.scrolledrecentsearchlist.checkdbssearchlink:rerunsearch/EC1A3CD8D3484721PQ/None/$N?site=sociologicalabstracts&t:ac=RecentSearches) | | [**3,571**](https://www.proquest.com/recentsearches.recentsearchtabview.recentsearchesgridview.scrolledrecentsearchlist.checkdbssearchlink_0:rerunsearch/EC1A3CD8D3484721PQ/None/$N?site=sociologicalabstracts&t:ac=RecentSearches) | |
| Select item 30 | **S30** | | [MAINSUBJECT.EXACT("Family support")](https://www.proquest.com/recentsearches.recentsearchtabview.recentsearchesgridview.scrolledrecentsearchlist.checkdbssearchlink:rerunsearch/6F97D4809E2A4722PQ/None/$N?site=sociologicalabstracts&t:ac=RecentSearches) | | [**1,293**](https://www.proquest.com/recentsearches.recentsearchtabview.recentsearchesgridview.scrolledrecentsearchlist.checkdbssearchlink_0:rerunsearch/6F97D4809E2A4722PQ/None/$N?site=sociologicalabstracts&t:ac=RecentSearches) | |
| Select item 29 | **S29** | | [MAINSUBJECT.EXACT("Family relations")](https://www.proquest.com/recentsearches.recentsearchtabview.recentsearchesgridview.scrolledrecentsearchlist.checkdbssearchlink:rerunsearch/600920839E1D4B00PQ/None/$N?site=sociologicalabstracts&t:ac=RecentSearches) | | [**13,058**](https://www.proquest.com/recentsearches.recentsearchtabview.recentsearchesgridview.scrolledrecentsearchlist.checkdbssearchlink_0:rerunsearch/600920839E1D4B00PQ/None/$N?site=sociologicalabstracts&t:ac=RecentSearches) | |
| Select item 28 | **S28** | | [MAINSUBJECT.EXACT("Interpersonal relations")](https://www.proquest.com/recentsearches.recentsearchtabview.recentsearchesgridview.scrolledrecentsearchlist.checkdbssearchlink:rerunsearch/5FCC2A58869B4533PQ/None/$N?site=sociologicalabstracts&t:ac=RecentSearches) | | [**10,855**](https://www.proquest.com/recentsearches.recentsearchtabview.recentsearchesgridview.scrolledrecentsearchlist.checkdbssearchlink_0:rerunsearch/5FCC2A58869B4533PQ/None/$N?site=sociologicalabstracts&t:ac=RecentSearches) | |
| Select item 27 | **S27** | | [MAINSUBJECT.EXACT("Family roles")](https://www.proquest.com/recentsearches.recentsearchtabview.recentsearchesgridview.scrolledrecentsearchlist.checkdbssearchlink:rerunsearch/A936A648A55C41D4PQ/None/$N?site=sociologicalabstracts&t:ac=RecentSearches) | | [**6,745**](https://www.proquest.com/recentsearches.recentsearchtabview.recentsearchesgridview.scrolledrecentsearchlist.checkdbssearchlink_0:rerunsearch/A936A648A55C41D4PQ/None/$N?site=sociologicalabstracts&t:ac=RecentSearches) | |
| Select item 26 | **S26** | | [noft(caregiver* or care-giver* or ("care giver" OR "care givers") or caregiving or carer* or care-partner* or (care NEAR/2 partner*) or (care NEAR/1 giving) or (care NEAR/2 provider*) or (support NEAR/2 provider*) or (support NEAR/2 partner*))](https://www.proquest.com/recentsearches.recentsearchtabview.recentsearchesgridview.scrolledrecentsearchlist.checkdbssearchlink:rerunsearch/DEF093B98F9646EFPQ/None/$N?site=sociologicalabstracts&t:ac=RecentSearches) | | [**44,696**](https://www.proquest.com/recentsearches.recentsearchtabview.recentsearchesgridview.scrolledrecentsearchlist.checkdbssearchlink_0:rerunsearch/DEF093B98F9646EFPQ/None/$N?site=sociologicalabstracts&t:ac=RecentSearches) | |
| Select item 25 | **S25** | | [MAINSUBJECT.EXACT("Caregiving")](https://www.proquest.com/recentsearches.recentsearchtabview.recentsearchesgridview.scrolledrecentsearchlist.checkdbssearchlink:rerunsearch/153A01C119114C29PQ/None/$N?site=sociologicalabstracts&t:ac=RecentSearches) | | [**2,795**](https://www.proquest.com/recentsearches.recentsearchtabview.recentsearchesgridview.scrolledrecentsearchlist.checkdbssearchlink_0:rerunsearch/153A01C119114C29PQ/None/$N?site=sociologicalabstracts&t:ac=RecentSearches) | |
| Select item 24 | **S24** | | [MAINSUBJECT.EXACT("Caregivers")](https://www.proquest.com/recentsearches.recentsearchtabview.recentsearchesgridview.scrolledrecentsearchlist.checkdbssearchlink:rerunsearch/216D03B443B4AB0PQ/None/$N?site=sociologicalabstracts&t:ac=RecentSearches) | | [**21,755**](https://www.proquest.com/recentsearches.recentsearchtabview.recentsearchesgridview.scrolledrecentsearchlist.checkdbssearchlink_0:rerunsearch/216D03B443B4AB0PQ/None/$N?site=sociologicalabstracts&t:ac=RecentSearches) | |
| Select item 23 | **S23** | | [[S1] OR [S2] OR [S3] OR [S4] OR [S5] OR [S6] OR [S7] OR [S8] OR [S9] OR [S10] OR [S11] OR [S12] OR [S13] OR [S14] OR [S15] OR [S16] OR [S17] OR [S18] OR [S19] OR [S20] OR [S21] OR [S22]](https://www.proquest.com/recentsearches.recentsearchtabview.recentsearchesgridview.scrolledrecentsearchlist.checkdbssearchlink:rerunsearch/B1A8D597C16344A9PQ/None/$N?site=sociologicalabstracts&t:ac=RecentSearches) | | [**18,685**](https://www.proquest.com/recentsearches.recentsearchtabview.recentsearchesgridview.scrolledrecentsearchlist.checkdbssearchlink_0:rerunsearch/B1A8D597C16344A9PQ/None/$N?site=sociologicalabstracts&t:ac=RecentSearches) | |
|  |  | |  | |  | |
| Select item 22 | | **S22** | | [noft(separate* NEAR/1 (interview* or "semi-structured interview" or “semi-structured interviews”))](https://www.proquest.com/recentsearches.recentsearchtabview.recentsearchesgridview.scrolledrecentsearchlist.checkdbssearchlink:rerunsearch/C51D436EFFE943F1PQ/None/$N?site=sociologicalabstracts&t:ac=RecentSearches) | | [**392**](https://www.proquest.com/recentsearches.recentsearchtabview.recentsearchesgridview.scrolledrecentsearchlist.checkdbssearchlink_0:rerunsearch/C51D436EFFE943F1PQ/None/$N?site=sociologicalabstracts&t:ac=RecentSearches) |
| Select item 21 | | **S21** | | [noft((couples or couple) NEAR/3 experience*)](https://www.proquest.com/recentsearches.recentsearchtabview.recentsearchesgridview.scrolledrecentsearchlist.checkdbssearchlink:rerunsearch/E6E3361878524C82PQ/None/$N?site=sociologicalabstracts&t:ac=RecentSearches) | | [**1,052**](https://www.proquest.com/recentsearches.recentsearchtabview.recentsearchesgridview.scrolledrecentsearchlist.checkdbssearchlink_0:rerunsearch/E6E3361878524C82PQ/None/$N?site=sociologicalabstracts&t:ac=RecentSearches) |
| Select item 20 | | **S20** | | [noft((couples or couple) NEAR/3 interview*)](https://www.proquest.com/recentsearches.recentsearchtabview.recentsearchesgridview.scrolledrecentsearchlist.checkdbssearchlink:rerunsearch/A37B97618463462BPQ/None/$N?site=sociologicalabstracts&t:ac=RecentSearches) | | [**1,124**](https://www.proquest.com/recentsearches.recentsearchtabview.recentsearchesgridview.scrolledrecentsearchlist.checkdbssearchlink_0:rerunsearch/A37B97618463462BPQ/None/$N?site=sociologicalabstracts&t:ac=RecentSearches) |
| Select item 19 | | **S19** | | [noft("couple level")](https://www.proquest.com/recentsearches.recentsearchtabview.recentsearchesgridview.scrolledrecentsearchlist.checkdbssearchlink:rerunsearch/83FB9F725E7E44C2PQ/None/$N?site=sociologicalabstracts&t:ac=RecentSearches) | | [**293**](https://www.proquest.com/recentsearches.recentsearchtabview.recentsearchesgridview.scrolledrecentsearchlist.checkdbssearchlink_0:rerunsearch/83FB9F725E7E44C2PQ/None/$N?site=sociologicalabstracts&t:ac=RecentSearches) |
| Select item 18 | | **S18** | | [MAINSUBJECT.EXACT("Couples")](https://www.proquest.com/recentsearches.recentsearchtabview.recentsearchesgridview.scrolledrecentsearchlist.checkdbssearchlink:rerunsearch/DC2511F4C480489FPQ/None/$N?site=sociologicalabstracts&t:ac=RecentSearches) | | [**13,676**](https://www.proquest.com/recentsearches.recentsearchtabview.recentsearchesgridview.scrolledrecentsearchlist.checkdbssearchlink_0:rerunsearch/DC2511F4C480489FPQ/None/$N?site=sociologicalabstracts&t:ac=RecentSearches) |
| Select item 17 | | **S17** | | [noft("multi family member" NEAR/2 (interview* or study or studies))](https://www.proquest.com/recentsearches.recentsearchtabview.recentsearchesgridview.scrolledrecentsearchlist.checkdbssearchlink:rerunsearch/48F63CFCD4EC4463PQ/None/$N?site=sociologicalabstracts&t:ac=RecentSearches) | | [**5**](https://www.proquest.com/recentsearches.recentsearchtabview.recentsearchesgridview.scrolledrecentsearchlist.checkdbssearchlink_0:rerunsearch/48F63CFCD4EC4463PQ/None/$N?site=sociologicalabstracts&t:ac=RecentSearches) |
| Select item 16 | | **S16** | | [noft((multiperspectiv* or multi-perspectiv* or "multiple perspective" or “multiple perspectives”) NEAR/3 interview*)](https://www.proquest.com/recentsearches.recentsearchtabview.recentsearchesgridview.scrolledrecentsearchlist.checkdbssearchlink:rerunsearch/988EB06389014B8APQ/None/$N?site=sociologicalabstracts&t:ac=RecentSearches) | | [**16**](https://www.proquest.com/recentsearches.recentsearchtabview.recentsearchesgridview.scrolledrecentsearchlist.checkdbssearchlink_0:rerunsearch/988EB06389014B8APQ/None/$N?site=sociologicalabstracts&t:ac=RecentSearches) |
| Select item 15 | | **S15** | | [noft((multiperspectiv* or multi-perspectiv* or "multiple perspective" or “multiple perspectives”) NEAR/1 approach*)](https://www.proquest.com/recentsearches.recentsearchtabview.recentsearchesgridview.scrolledrecentsearchlist.checkdbssearchlink:rerunsearch/59B97A3007C14CCEPQ/None/$N?site=sociologicalabstracts&t:ac=RecentSearches) | | [**83**](https://www.proquest.com/recentsearches.recentsearchtabview.recentsearchesgridview.scrolledrecentsearchlist.checkdbssearchlink_0:rerunsearch/59B97A3007C14CCEPQ/None/$N?site=sociologicalabstracts&t:ac=RecentSearches) |
| Select item 14 | | **S14** | | [noft((multiperspectiv* or multi-perspectiv* or "multiple perspective" or “multiple perspectives”) NEAR/2 (study* or studie*))](https://www.proquest.com/recentsearches.recentsearchtabview.recentsearchesgridview.scrolledrecentsearchlist.checkdbssearchlink:rerunsearch/67F2366C72144806PQ/None/$N?site=sociologicalabstracts&t:ac=RecentSearches) | | [**67**](https://www.proquest.com/recentsearches.recentsearchtabview.recentsearchesgridview.scrolledrecentsearchlist.checkdbssearchlink_0:rerunsearch/67F2366C72144806PQ/None/$N?site=sociologicalabstracts&t:ac=RecentSearches) |
| Select item 13 | | **S13** | | [noft((multiperspectiv* or multi-perspectiv* or "multiple perspective" or "multiple perspectives") NEAR/3 (analys* or analyz*))](https://www.proquest.com/recentsearches.recentsearchtabview.recentsearchesgridview.scrolledrecentsearchlist.checkdbssearchlink:rerunsearch/4ED9F792FA1E4DE7PQ/None/$N?site=sociologicalabstracts&t:ac=RecentSearches) | | [**78**](https://www.proquest.com/recentsearches.recentsearchtabview.recentsearchesgridview.scrolledrecentsearchlist.checkdbssearchlink_0:rerunsearch/4ED9F792FA1E4DE7PQ/None/$N?site=sociologicalabstracts&t:ac=RecentSearches) |
| Select item 12 | | **S12** | | [noft(within NEAR/2 dyad*)](https://www.proquest.com/recentsearches.recentsearchtabview.recentsearchesgridview.scrolledrecentsearchlist.checkdbssearchlink:rerunsearch/2AA1ACE20C22407DPQ/None/$N?site=sociologicalabstracts&t:ac=RecentSearches) | | [**400**](https://www.proquest.com/recentsearches.recentsearchtabview.recentsearchesgridview.scrolledrecentsearchlist.checkdbssearchlink_0:rerunsearch/2AA1ACE20C22407DPQ/None/$N?site=sociologicalabstracts&t:ac=RecentSearches) |
| Select item 11 | | **S11** | | [noft(dyad* NEAR/7 interview*)](https://www.proquest.com/recentsearches.recentsearchtabview.recentsearchesgridview.scrolledrecentsearchlist.checkdbssearchlink:rerunsearch/2093EB7B63BD439EPQ/None/$N?site=sociologicalabstracts&t:ac=RecentSearches) | | [**443**](https://www.proquest.com/recentsearches.recentsearchtabview.recentsearchesgridview.scrolledrecentsearchlist.checkdbssearchlink_0:rerunsearch/2093EB7B63BD439EPQ/None/$N?site=sociologicalabstracts&t:ac=RecentSearches) |
| Select item 10 | | **S10** | | [noft(dyad* NEAR/3 level*)](https://www.proquest.com/recentsearches.recentsearchtabview.recentsearchesgridview.scrolledrecentsearchlist.checkdbssearchlink:rerunsearch/91969B6D6DAF44AAPQ/None/$N?site=sociologicalabstracts&t:ac=RecentSearches) | | [**492**](https://www.proquest.com/recentsearches.recentsearchtabview.recentsearchesgridview.scrolledrecentsearchlist.checkdbssearchlink_0:rerunsearch/91969B6D6DAF44AAPQ/None/$N?site=sociologicalabstracts&t:ac=RecentSearches) |
| Select item 9 | | **S9** | | [noft(dyad* NEAR/4 participant*)](https://www.proquest.com/recentsearches.recentsearchtabview.recentsearchesgridview.scrolledrecentsearchlist.checkdbssearchlink:rerunsearch/803161F73CFD468DPQ/None/$N?site=sociologicalabstracts&t:ac=RecentSearches) | | [**360**](https://www.proquest.com/recentsearches.recentsearchtabview.recentsearchesgridview.scrolledrecentsearchlist.checkdbssearchlink_0:rerunsearch/803161F73CFD468DPQ/None/$N?site=sociologicalabstracts&t:ac=RecentSearches) |
| Select item 8 | | **S8** | | [noft(dyad* NEAR/3 unit)](https://www.proquest.com/recentsearches.recentsearchtabview.recentsearchesgridview.scrolledrecentsearchlist.checkdbssearchlink:rerunsearch/131B736F74D94C7CPQ/None/$N?site=sociologicalabstracts&t:ac=RecentSearches) | | [**64**](https://www.proquest.com/recentsearches.recentsearchtabview.recentsearchesgridview.scrolledrecentsearchlist.checkdbssearchlink_0:rerunsearch/131B736F74D94C7CPQ/None/$N?site=sociologicalabstracts&t:ac=RecentSearches) |
| Select item 7 | | **S7** | | [noft(dyad* NEAR/2 approach*)](https://www.proquest.com/recentsearches.recentsearchtabview.recentsearchesgridview.scrolledrecentsearchlist.checkdbssearchlink:rerunsearch/A7EAB07C6BED4ED6PQ/None/$N?site=sociologicalabstracts&t:ac=RecentSearches) | | [**209**](https://www.proquest.com/recentsearches.recentsearchtabview.recentsearchesgridview.scrolledrecentsearchlist.checkdbssearchlink_0:rerunsearch/A7EAB07C6BED4ED6PQ/None/$N?site=sociologicalabstracts&t:ac=RecentSearches) |
| Select item 6 | | **S6** | | [noft(dyad* NEAR/4 sampl*)](https://www.proquest.com/recentsearches.recentsearchtabview.recentsearchesgridview.scrolledrecentsearchlist.checkdbssearchlink:rerunsearch/2E1FEEB5F925455DPQ/None/$N?site=sociologicalabstracts&t:ac=RecentSearches) | | [**429**](https://www.proquest.com/recentsearches.recentsearchtabview.recentsearchesgridview.scrolledrecentsearchlist.checkdbssearchlink_0:rerunsearch/2E1FEEB5F925455DPQ/None/$N?site=sociologicalabstracts&t:ac=RecentSearches) |
| Select item 5 | | **S5** | | [noft(dyad* NEAR/4 perspective*)](https://www.proquest.com/recentsearches.recentsearchtabview.recentsearchesgridview.scrolledrecentsearchlist.checkdbssearchlink:rerunsearch/5A76713F0EB74123PQ/None/$N?site=sociologicalabstracts&t:ac=RecentSearches) | | [**199**](https://www.proquest.com/recentsearches.recentsearchtabview.recentsearchesgridview.scrolledrecentsearchlist.checkdbssearchlink_0:rerunsearch/5A76713F0EB74123PQ/None/$N?site=sociologicalabstracts&t:ac=RecentSearches) |
| Select item 4 | | **S4** | | [noft(dyad* NEAR/3 experience*)](https://www.proquest.com/recentsearches.recentsearchtabview.recentsearchesgridview.scrolledrecentsearchlist.checkdbssearchlink:rerunsearch/6A25EC8DB0F64B3DPQ/None/$N?site=sociologicalabstracts&t:ac=RecentSearches) | | [**145**](https://www.proquest.com/recentsearches.recentsearchtabview.recentsearchesgridview.scrolledrecentsearchlist.checkdbssearchlink_0:rerunsearch/6A25EC8DB0F64B3DPQ/None/$N?site=sociologicalabstracts&t:ac=RecentSearches) |
| Select item 3 | | **S3** | | [noft(dyad* NEAR/3 (analys* or analyz* or analytic*))](https://www.proquest.com/recentsearches.recentsearchtabview.recentsearchesgridview.scrolledrecentsearchlist.checkdbssearchlink:rerunsearch/FC5E5276FEAD406EPQ/None/$N?site=sociologicalabstracts&t:ac=RecentSearches) | | [**818**](https://www.proquest.com/recentsearches.recentsearchtabview.recentsearchesgridview.scrolledrecentsearchlist.checkdbssearchlink_0:rerunsearch/FC5E5276FEAD406EPQ/None/$N?site=sociologicalabstracts&t:ac=RecentSearches) |
| Select item 2 | | **S2** | | [noft(dyad* NEAR/2 (study* or studie*))](https://www.proquest.com/recentsearches.recentsearchtabview.recentsearchesgridview.scrolledrecentsearchlist.checkdbssearchlink:rerunsearch/97AE78498D8E4920PQ/None/$N?site=sociologicalabstracts&t:ac=RecentSearches) | | [**498**](https://www.proquest.com/recentsearches.recentsearchtabview.recentsearchesgridview.scrolledrecentsearchlist.checkdbssearchlink_0:rerunsearch/97AE78498D8E4920PQ/None/$N?site=sociologicalabstracts&t:ac=RecentSearches) |
| Select item 1 | | **S1** | | [MAINSUBJECT.EXACT.EXPLODE("Dyads")](https://www.proquest.com/recentsearches.recentsearchtabview.recentsearchesgridview.scrolledrecentsearchlist.checkdbssearchlink:rerunsearch/2F3F46A63DD3406BPQ/None/$N?site=sociologicalabstracts&t:ac=RecentSearches) | | [**1,413**](https://www.proquest.com/recentsearches.recentsearchtabview.recentsearchesgridview.scrolledrecentsearchlist.checkdbssearchlink_0:rerunsearch/2F3F46A63DD3406BPQ/None/$N?site=sociologicalabstracts&t:ac=RecentSearches) |

Name:

08Feb2024_CareDyadComps[Edit name](https://www.proquest.com/sociologicalabstracts/myresearch/savedsearches/SavedSearches/0?accountid=14771)

Searched for:

2010 onward

Name:

([S23] AND [S40] AND [S44]) AND yr(2010-2029)[Edit name](https://www.proquest.com/sociologicalabstracts/myresearch/savedsearches/SavedSearches/0?accountid=14771)

Searched for:

[((MAINSUBJECT.EXACT.EXPLODE("Dyads") OR noft(dyad* NEAR/2 (study* OR studie*)) OR noft(dyad* NEAR/3 (analys* OR analyz* OR analytic*)) OR noft(dyad* NEAR/3 experience*) OR noft(dyad* NEAR/4 perspective*) OR noft(dyad* NEAR/4 sampl*) OR noft(dyad* NEAR/2 approach*) OR noft(dyad* NEAR/3 unit) OR noft(dyad* NEAR/4 participant*) OR noft(dyad* NEAR/3 level*) OR noft(dyad* NEAR/7 interview*) OR noft(within NEAR/2 dyad*) OR noft((multiperspectiv* OR multi-perspectiv* OR "multiple perspective" OR "multiple perspectives") NEAR/3 (analys* OR analyz*)) OR noft((multiperspectiv* OR multi-perspectiv* OR "multiple perspective" OR "multiple perspectives") NEAR/2 (study* OR studie*)) OR noft((multiperspectiv* OR multi-perspectiv* OR "multiple perspective" OR "multiple perspectives") NEAR/1 approach*) OR noft((multiperspectiv* OR multi-perspectiv* OR "multiple perspective" OR "multiple perspectives") NEAR/3 interview*) OR noft("multi family member" NEAR/2 (interview* OR study OR studies)) OR MAINSUBJECT.EXACT("Couples") OR noft("couple level") OR noft((couples OR couple) NEAR/3 interview*) OR noft((couples OR couple) NEAR/3 experience*) OR noft(separate* NEAR/1 (interview* OR "semi-structured interview" OR "semi-structured interviews"))) AND (MAINSUBJECT.EXACT("Caregivers") OR MAINSUBJECT.EXACT("Caregiving") OR noft(caregiver* OR care-giver* OR ("care giver" OR "care givers") OR caregiving OR carer* OR care-partner* OR (care NEAR/2 partner*) OR (care NEAR/1 giving) OR (care NEAR/2 provider*) OR (support NEAR/2 provider*) OR (support NEAR/2 partner*)) OR MAINSUBJECT.EXACT("Family roles") OR MAINSUBJECT.EXACT("Interpersonal relations") OR MAINSUBJECT.EXACT("Family relations") OR MAINSUBJECT.EXACT("Family support") OR (MAINSUBJECT.EXACT("Marital adjustment") OR MAINSUBJECT.EXACT("Marital relations")) OR MAINSUBJECT.EXACT("Parent-child relations") OR MAINSUBJECT.EXACT("Intergenerational relationships") OR noft("family relation*" OR "interpersonal relation*" OR "intergenerational relation*" "inter-generational relation*" OR "family support*" OR "family assistance" OR "family encouragement" OR "marital relation*" OR "marital adjustment*") OR (MAINSUBJECT.EXACT("Spouses") OR MAINSUBJECT.EXACT("Husbands") OR MAINSUBJECT.EXACT("Wives")) OR (MAINSUBJECT.EXACT("Parents & parenting") OR MAINSUBJECT.EXACT("Mothers") OR MAINSUBJECT.EXACT("Fathers") OR MAINSUBJECT.EXACT("Grandparents")) OR (MAINSUBJECT.EXACT("Siblings") OR MAINSUBJECT.EXACT("Daughters") OR MAINSUBJECT.EXACT("Sons")) OR MAINSUBJECT.EXACT("Adult children") OR noft("family member*" OR spouse* OR husband* OR wife OR wives OR "domestic partner*" OR "registered partner*" OR "civil partner*" OR parent* OR father* OR mother* OR grandparent* OR grand-parent* OR grandmother* OR grand-mother* OR grandfather* OR grand-father* OR brother* OR sister* OR sibling* OR daughter* OR son OR sons OR children OR child)) AND (MAINSUBJECT.EXACT("Qualitative research" OR "Grounded Theory" OR "Narratives" OR "Focus groups" OR "Discourse analysis" OR "Content analysis" OR "Ethnography" OR "Autoethnography" OR "Ethnomethodology" OR "Observational studies" OR "Fieldwork" OR "Phenomenology" OR "Interpretive sociology" OR "Life history" OR "Storytelling" OR "Research Methodology") OR MAINSUBJECT.EXACT.EXPLODE("Interviews") OR noft(qualitative OR interview* OR theme* OR thematic OR ("ethnological research") OR ethnonursing OR ethnograph* OR phenomenol* OR (("focus group" OR "focus groups")) OR (grounded NEAR/1 (theor* OR analys?s OR research OR studies OR study)) OR (("life stories" OR "life story")) OR emic OR etic OR hermeneutic* OR heuristic* OR semiotic OR (data NEAR/1 saturat*) OR (("participant observation" OR "participant observer")) OR (("social construct" OR "social construction" OR "social constructionism" OR "social constructionist" OR "social constructionists" OR "social constructions" OR "social constructive" OR "social constructivism" OR "social constructivist" OR "social constructivists" OR "social constructs")) OR postmodern* OR ("post-structural*") OR poststructural* OR ("post-modern*") OR feminis* OR ("action research") OR ("cooperative inquir*") OR ("co-operative inquir*") OR humanistic OR existential OR experiential OR paradigm* OR (field NEAR/1 (research OR study OR studies)) OR ("human science") OR ("biographical method") OR AB "biographical method" OR ("theoretical sampl*") OR ("purpos* NEAR/4 sampl*") OR ("open-ended") OR narrative* OR textual OR texts OR ("semi-structured") OR ("life world") OR ("life-world") OR ("conversation analys?s") OR (("personal experience" OR "personal experiences")) OR ("theoretical saturation") OR ((life OR lived) NEAR/1 experience*) OR (("cluster sampling")) OR (("observational method" OR "observational methods")) OR ("content analysis") OR ((discurs* OR discourse*) NEAR/3 analys?s) OR (constant NEAR/1 (comparison OR comparative)) OR ("narrative analys?s") OR heidegger* OR colaizzi* OR spiegelberg* OR merleau* OR husserl* OR foucault* OR ricoeur OR glaser* OR (van NEAR/1 manen*) OR (van NEAR/1 kaam*) OR (Corbin* NEAR/2 strauss*)))) AND yr(2010-2029)](https://www.proquest.com/myresearch/savedsearches.checkdbssearchlink:rerunsearch/2547937/SavedSearches/$N?site=sociologicalabstracts&t:ac=SavedSearches)

**Appendix C: Definition and elaboration document for full text screening**

|  |  | Notes |
| --- | --- | --- |
| 1 | **Publication type**  Is this a peer reviewed English article published in full?  **NO: exclude Pub type** | -Exclude if abstract only or dissertation  -Exclude if not peer reviewed  -Exclude if not English |
| 2 | **Study design**  Is this a primary research study?  **NO**  OR  Is this a methodological paper or review?*  **NO**  **exclude Study design** | -Exclude if perspective, editorial, opinion, commentary, presentation, etc.  ***If methodological paper, skip 3-6 and go directly to 7*** |
| 3 | **Care partnership dyads**  Is ≥1 dyad(s) studied where 1 member has a health condition^1^ and shares a close relationship with the other member (not a health professional^2^) who is affected by the health condition in some way? (i.e. provides informal care or support, jointly copes or make decisions, etc.)  AND  Are both members 18 years of age or older?  **NO: exclude Population: no CP** | Exclude if:  -dyad under study is **not a** **care partnership**  -health professional is only care partner  -interviewed care partners and patients who do not share relationship (i.e. interviewed patient but not THEIR partner or vice versa)  -unclear if both members of dyad were included  Note: old age is not a health condition  *^1^Old age is not considered a health condition*  *^2^A health professional is someone who studies, advises on or provides preventive, curative, rehabilitative and promotional health services (i.e. nurse, physician, personal support worker, patient navigator, etc.)* |
| 4 | **How was data collected?** | Exclude if data not collected through **independent and separate interviews from BOTH members of dyad** |
|  | **No individual interviews:** dyads interviewed jointly only OR no interview with dyad  **Exclude Data collection: no ind intvw** | -Exclude if only joint interviews  -Exclude if joint interviews + individual interviews with 1 member of dyad only (i.e. joint interviews + individual interviews with patients but no individual interviews with care partners)  -Exclude if joint with dyads and individual interviews with some participants who were not clearly care partners)  -Exclude if data collected exclusively through a structured clinical interview, survey, or questionnaire |
|  | **Indeterminant:** unclear if individual interviews  **Exclude Data collection: intvw NS** | -Exclude if interview method was not reported and could not be determined based on interview guide or other info |
|  | **Individual AND joint interviews with all dyads**  **Exclude Data collection: joint + ind** | -Exclude if all dyads were interviewed jointly and individually |
| 5 | Were individual interviews for each dyad member analyzed dyadically^3^?  **NO: exclude at No DA** | -Exclude if does not report **qualitative** **analysis** of **individual interviews from person with health condition and care partner** at the **level of the dyad/couple**  -Exclude if analysis at aggregate/group level only (all patients analyzed together and all care partners analyzed together)  -Exclude if ‘across’ dyads only (not ‘within’ dyads)  *^3^A qualitative analysis of 2 individual interviews as a unit, where accounts of both members are analyzed together and synthesized to understand the joint experience* |
|  | **NO:** **analysis of triads or dyads that were not care partnerships**  **Exclude at analysis triadic (DA not CP)** | Exclude if triads analyzed at triadic level  Exclude if multiperspective with a dyadic analysis that is not of care partnership (i.e. dyadic analysis of mothers + fathers of person with health condition) and then 3+ perspectives including CP analyzed (i.e. mother + father+ person with health condition) |
| 6 | Is the process of HOW the dyadic analysis was conducted described in detail?  **NO: exclude Analysis: insuff info DA** | Must provide details regarding how an analysis of separate interviews at the level of the dyad was conducted such that the procedure could be replicated |
| 7 | **For methodological papers or reviews:**  Is this a methodological paper or review describing the dyadic analysis of care partnerships?  **NO: exclude Methodological: insuff desc/CP** | Exclude if methodologic about triadic  Exclude if methodologic about dyads who are not care partnerships  *Care partnership as defined in #3; dyadic analysis as defined in #5)* |

**Appendix D: Data synthesis process- sequence of analysis, dyadic analysis step, and justifications for dyadic analysis**

| **Extracted data** | **Analytic summary** | **Open coding** (2) | **Category development** |  |
| --- | --- | --- | --- | --- |
| ***From Demirtepe-Saygılı, 2022: p. 2081*** (1)***:***  *A dyadic qualitative analysis was conducted to investigate the research questions. In this way, contrasts and overlaps can be revealed, helping the researchers observe the couple’s experience of a phenomenon as an individual and as a unit of a couple (Eisikovits and Koren, 2010). This approach provides a rich perspective especially about shared phenomena, such as parenting (e.g. Reed, 2006) or adaptation to disorders (e.g. Antoine et al., 2018). Moreover, having information from multiple sources enhances trustworthiness. After investigating the scripts individually, a dyadic analysis was conducted as suggested by Eisikovits and Koren (2010). That is, content analysis was conducted at an individual level and the themes were identified. Then, the themes from the couple’s scripts were compared. In this way, the answers were evaluated both as an individual and as a part of a couple. The contrasts and overlaps were taken into consideration. The themes were then categorized as convergent and divergent based on their similarities and differences. The codings which cannot be categorized into either category were treated as independent themes that refer to different issues on different topics provided by either the participant with MS [multiple sclerosis]or the partner. The independent themes were not evaluated further, as it is beyond the scope of the study.* | **Analytic steps:**   - Content analysis was conducted at an individual level and themes identified. - Then, themes from couple’s scripts [from individual analysis] were compared and categorized as convergent or divergent based on their similarities and differences. Codings that could not be categorized into either category were treated as independent themes, which were not evaluated further. - No analysis across dyads. | 1. Analysis at individual level  2. Analysis at dyadic level using themes from individual level | Analysis at individual level, then at dyadic level informed by results from analysis at individual level | **Sequence of analysis** |
|  |  | Themes from individual analysis compared and categorized as convergent or divergent based on their similarities and differences. | Side-by-side readings to compare and categorize themes, codes, or accounts within each dyad | **Dyadic analysis step** |
|  | **Justifications for dyadic analysis:**   - To help the researchers observe the couple’s experience of a phenomenon as an individual and as a unit of a couple. - To provide a richer perspective about a shared phenomena. - To enhance trustworthiness by having information from multiple sources. | To observe the couple’s experience as an individual and as a unit. | To understand experience of both partners as individuals and as a unit. | **Justifications for dyadic analysis** |
|  |  | To provide a richer perspective about a shared phenomena. | To capture and enhance understanding of the experiences of the dyad. |  |
|  |  | To enhance trustworthiness by having information from multiple sources. | To enhance trustworthiness or validity. |  |

1. Demirtepe-Saygılı D. Multiple sclerosis experiences of couples: Examination of patient and partner perspectives. Journal of Health Psychology. 2022;27(9):2079-90.
2. Pollock D, Peters MDJ, Khalil H, McInerney P, Alexander L, Tricco AC, et al. Recommendations for the extraction, analysis, and presentation of results in scoping reviews. JBI Evid Synth. 2023;21(3):520-32.

**Appendix E: Template for dyadic study conceptualization at different research steps**

| **Research steps** | | | **Coding Scheme** |
| --- | --- | --- | --- |
| **Study Design** | **Study aim(s)** (1-4) | Study/research aim(s) concerned with shared experience of or relationship between dyad members | **Dyadic** |
|  |  | Study/research aim(s) not concerned with shared experience of or relationship between dyad members | **Non-dyadic** |
|  |  | Study aim not reported | **NR** |
|  | **Methodological approach** (5-8) | Dyadic or multiperspective approach to study design reported | **Dyadic** |
|  |  | Methodological approach reported but not dyadic or multiperspective | **Non-dyadic** |
|  |  | Methodological approach not reported | **NR** |
| **Participant selection** | **Participant selection** (9) | Both dyad members required to participate  Inclusion or exclusion criteria directed at dyad | **Dyadic** |
|  |  | Participation of corresponding dyad member not required | **Non-dyadic** |
|  |  | Participant selection not reported | **NR** |
|  | **Sampling strategy or framework** (3) | Sampling directed at dyad  Sampling directed at dyad and one dyad member | **Dyadic** |
|  |  | Sampling directed at one dyad member | **Non-dyadic** |
|  |  | Sampling reported but not who it was directed towards | **Unclear** |
|  |  | Sampling not reported | **NR** |
|  | **Sample** | Dyads only | **Dyadic** |
|  |  | Dyads and singletons (included participants without corresponding partner)  Dyads and triads | **Non-dyadic** |
| **Data collection** | **Interview guide/ questions** (5) | Dyad members asked similarly themed, related, or same questions (dyadic method of questioning) | **Dyadic** |
|  |  | Dyad members asked unrelated questions | **Non-dyadic** |
|  |  | Interview questions not reported | **NR** |
|  | **Saturation (level)** (6, 10) | Saturation at dyad level | **Dyadic** |
|  |  | Saturation at aggregate (group) level  Saturation at individual level | **Non-dyadic** |
|  |  | Unclear at what level | **Unclear** |
|  |  | Saturation not reported | **NR** |
| **Rigour** | **Steps taken to establish trustworthiness and rigour** (5, 9) | Reports steps to establish trustworthiness and rigour in reference to dyadic data collection and/or analysis | **Dyadic** |
|  |  | Reports steps to establish trustworthiness and rigour but not in reference to dyadic data collection and/or analysis | **Non-dyadic** |
|  |  | Steps to establish trustworthiness and rigor not reported | **NR** |

1. Eisikovits Z, Koren C. Approaches to and Outcomes of Dyadic Interview Analysis. Qualitative Health Research. 2010;20(12):1642-55.
2. Larkin M, Shaw R, Flowers P. Multiperspectival designs and processes in interpretative phenomenological analysis research. Qualitative Research in Psychology. 2019;16(2):182-98.
3. Thompson L, Walker AJ. The dyad as the unit of analysis: Conceptual and methodological issues. Journal of Marriage and the Family. 1982;44(4):889-900.
4. Koren C. A Complex Unit Interviews Analysis Approach in Qualitative Social Work Research. British Journal of Social Work. 2023;53(6):3258-76.
5. Hudson N, Law C, Culley L, Mitchell H, Denny E, Raine-Fenning N. Conducting dyadic, relational research about endometriosis: A reflexive account of methods, ethics and data analysis. Health (London, England : 1997). 2020;24(1):79-93.
6. Reczek C. Conducting a multi family member interview study. Fam Process. 2014;53(2):318-35.
7. Yosha AM, Carroll JK, Hendren S, Salamone CM, Sanders M, Fiscella K, et al. Patient navigation from the paired perspectives of cancer patients and navigators: a qualitative analysis. Patient Educ Couns. 2011;82(3):396-401.
8. McCarthy JR, Holland J, Gillies V. Multiple perspectives on the 'family' lives of young people: Methodological and theoretical issues in case study research. International Journal of Social Research Methodology. 2003;6(1):1-23.
9. Ummel D, Achille M. How Not to Let Secrets Out When Conducting Qualitative Research With Dyads. Qualitative Health Research. 2016;26(6):807-15.
10. Boeije H. A Purposeful Approach to the Constant Comparative Method in the Analysis of Qualitative Interviews. Quality and Quantity. 2002;36(4):391-409.

**Appendix F: Sources excluded following full-text review**

**Incorrect publication type**

1. Dias T, Cravo P, Santos J, Gomes C, Santiago M, Pereira CM. The impact of COVID-19 on community-dwelling people post-stroke and informal caregivers: a qualitative study. medRxiv. 2023.

**No care partnership**

2. Barrett B, Phillips SL, Bulat T, Lind JD, Ballistrea L, Ramrattan A, et al. Evaluation of a new assistive technology: the StandBar. Disability and rehabilitation Assistive technology. 2022:1-11.

3. De Jong Gierveld J. Intra-couple Caregiving of Older Adults Living Apart Together: Commitment and Independence. Canadian journal on aging = La revue canadienne du vieillissement. 2015;34(3):356-65.

4. Fleming J, Farquhar M, Brayne C, Barclay S, Buck J, Dening T, et al. Death and the oldest old: Attitudes and preferences for end-of-life care - Qualitative research within a population-based cohort study. PLoS ONE. 2016;11(4):e0150686.

5. Fletcher JR. Distributed selves: Shifting inequities of impression management in couples living with dementia. Symbolic Interaction. 2020;43(3):405-27.

6. Fletcher JR. Renegotiating relationships: Theorising shared experiences of dementia within the dyadic career. Dementia (14713012). 2020;19(3):708-20.

7. Hawkey AJ, Ussher JM, Perz J, Parton C, Patterson P, Bateson D, et al. The impact of cancer-related fertility concerns on current and future couple relationships: People with cancer and partner perspectives. European Journal of Cancer Care. 2021;30(1).

8. Jaeger S, Huther F, Steinert T. Refusing medication therapy in involuntary inpatient treatment-A multiperspective qualitative study. Frontiers in Psychiatry. 2019;10(MAY):295.

9. Koenig TL, Lee JH, Macmillan KR, Fields NL, Spano R. Older adult and family member perspectives of the decision-making process involved in moving to assisted living. Qualitative Social Work: Research and Practice. 2014;13(3):335-50.

10. Koren C, Simhi S, Lipman-Schiby S, Fogel S. The partner in late-life repartnering: caregiving expectations from an intergenerational perspective. International Psychogeriatrics. 2016;28(9):1555-65.

11. Lee YEC, Stretton‐Smith PA, Tamplin J, Sousa TV, Baker FA. Therapeutic music interventions with people with dementia living in residential aged care: Perspectives of residents, family members and care home staff from a cluster randomised controlled trial. International Journal of Older People Nursing. 2022;17(3):1-15.

12. Macedo T, Sousa L, Ribeiro O. Aged 70 and still a child: complexities, strains and gains of older children caring for their (near) centenarian mothers. Age & Ageing. 2022;51(1):1-8.

13. Musheke M, Bond V, Merten S. Couple experiences of provider-initiated couple HIV testing in an antenatal clinic in Lusaka, Zambia: lessons for policy and practice. BMC Health Services Research. 2013;13(1):97-.

14. Neate SL, Taylor KL, Jelinek GA, De Livera AM, Simpson S, Jr., Bevens W, et al. On the path together: Experiences of partners of people with multiple sclerosis of the impact of lifestyle modification on their relationship. Health & Social Care in the Community. 2019;27(6):1515-24.

15. O'Hora KA, Roberto KA. Navigating emotions and relationship dynamics: Family life review as a clinical tool for older adults during a relocation transition into an assisted living facility. Aging & Mental Health. 2019;23(4):404-10.

16. Parker L, Pettifor A, Maman S, Sibeko J, MacPhail C. Concerns about partner infidelity are a barrier to adoption of HIV-prevention strategies among young South African couples. Culture, Health & Sexuality. 2014;16(7):792-805.

17. Pecchioni LL. Interruptions to Cultural Life Scripts: Cancer Diagnoses, Contextual Age, and Life Narratives. Research on Aging. 2012;34(6):758-80.

18. Pokharel M, Elrick A, Canary HE, Clayton MF, Sukovic M, Champine M, et al. Health communication roles in Latino, Pacific Islander, and Caucasian Families: A qualitative investigation. Journal of Genetic Counseling. 2020;29(3):399-409.

19. Pollard LC, Graves H, Scott DL, Kingsley GH, Lempp H. Perceived barriers to integrated care in rheumatoid arthritis: views of recipients and providers of care in an inner-city setting. BMC Musculoskeletal Disorders. 2011;12(1):19-.

20. Sessanna L, Pomeroy SH, Askew Y, McDonald-Shanahan K, Couche M. The Experience of Working With a Faith Community Nurse Liaison of Care in a Primary Care Practice Among Older Adult Clients and Their Informal Caregiver. Journal of Holistic Nursing. 2021;39(3):272-84.

21. Sherman MD, Usset T, Voecks C, Harris JI. Roles of religion and spirituality among veterans who manage PTSD and their partners. Psychology of Religion and Spirituality. 2018;10(4):368-74.

22. Shtompel N, Ruggiano N, Thomlison B, Fant K. Dyadic, Self-Administered Cognitive Intervention for Healthy Older Adults: Participants' Perspectives. Activities, Adaptation & Aging. 2020;44(3):246-65.

23. Thomeer MB, Donnelly R, Reczek C, Umberson D. Planning for Future Care and the End of Life: A Qualitative Analysis of Gay, Lesbian, and Heterosexual Couples. Journal of Health & Social Behavior. 2017;58(4):473-87.

24. van Wijngaarden EJ, Leget CJW, Goossensen A. Till Death Do Us Part: The Lived Experience of an Elderly Couple Who Chose to End Their Lives by Spousal Self-euthanasia. Gerontologist. 2016;56(6):1062-71.

25. Villar-Loubet OM, Bruscantini L, Shikwane ME, Weiss S, Peltzer K, Jones DL, et al. HIV disclosure, sexual negotiation and male involvement in prevention-of-mother-to-child-transmission in South Africa. Culture, Health & Sexuality. 2013;15(3):253-68.

26. Voss H, Vogel A, Wagemans AMA, Francke AL, Metsemakers JFM, Courtens AM, et al. What is important for advance care planning in the palliative phase of people with intellectual disabilities? A multi‐perspective interview study. Journal of Applied Research in Intellectual Disabilities. 2020;33(2):160-71.

27. Ware NC, Pisarski EE, Haberer JE, Wyatt MA, Tumwesigye E, Baeten JM, et al. Lay social resources for support of adherence to antiretroviral prophylaxis for HIV prevention among serodiscordant couples in sub-Saharan Africa: A qualitative study. AIDS and Behavior. 2015;19(5):811-20.

28. Will CM, Henwood F, Weiner K, Williams R. Negotiating the practical ethics of 'self-tracking' in intimate relationships: Looking for care in healthy living. Social Science & Medicine. 2020;266.

29. Wright J, Maliwichi-Senganimalunje L. Pluralism and practicality: village health workers' responses to contested meanings of mental illness in Southern Malawi. Anthropology & Medicine. 2020;27(1):32-48.

**No individual interviews**

30. ‘Living the life of the other’: carers’ perspectives on changes in carer strain during the rehabilitation trajectory. International Journal of Care and Caring. 2022;6(4):528.

31. Ågård AS, Egerod I, Tønnesen E, Lomborg K. From spouse to caregiver and back: a grounded theory study of post-intensive care unit spousal caregiving. Journal of Advanced Nursing (John Wiley & Sons, Inc). 2015;71(8):1892-903.

32. Ahn S, Cobb SJ, Crouter SE, Lee CE, Crane MK, Anderson JG. Physical activity together for couples living with mild cognitive impairment (PAT-MCI): A feasibility study. Geriatric Nursing. 2024;55:221-8.

33. Albert SC, Eduardo Martinelli J, Costa Pessoa MS. Couples living with Alzheimer's disease talk about sex and intimacy: A phenomenological qualitative study. Dementia (14713012). 2023;22(2):390-404.

34. Allison TA, Gubner JM, Oh A, Harrison KL, Pham K, Barnes DE, et al. Meaningful Activities and Sources of Meaning for Community-Dwelling People Living with Dementia. Journal of the American Medical Directors Association. 2022;23(7):1191-.

35. Aurooj A, Mahmood Z. Subjective Experiences of Alzheimer's Disease in the Pakistani Cultural Context: An Exploratory Study. Journal of Religion & Health. 2022;61(1):125-38.

36. Barrado-Martín Y, Heward M, Polman R, Nyman SR. Adherence to the Class-Based Component of a Tai Chi Exercise Intervention for People Living With Dementia and Their Informal Carers. Journal of Aging & Physical Activity. 2021;29(5):721-34.

37. Barrado-Martín Y, Heward M, Polman R, Nyman SR. People living with dementia and their family carers' adherence to home-based Tai Chi practice. Dementia (14713012). 2021;20(5):1586-603.

38. Beaudet L, Ducharme F. Living with moderate-stage Parkinson disease: intervention needs and preferences of elderly couples. Journal of Neuroscience Nursing. 2013;45(2):88-95.

39. Benbow SM, Kingston P. Spontaneous concerns about risk and abuse reported by people with dementia and their carers. The Journal of Adult Protection. 2017;19(2):92.

40. Benidir A, Levert M-J, Bilodeau K. The Role of Islamic Beliefs in Facilitating Acceptance of Cancer Diagnosis. Current Oncology. 2023;30(9):7789-801.

41. Berridge C, Turner NR, Liu L, Karras SW, Chen A, Fredriksen-Goldsen K, et al. Advance Planning for Technology Use in Dementia Care: Development, Design, and Feasibility of a Novel Self-administered Decision-Making Tool. JMIR aging. 2022;5(3):e39335.

42. Bielsten T, Keady J, Kullberg A, Lasrado R, Hellström I. Couples' experiences of using DemPower in everyday life. Quality in Ageing & Older Adults. 2020;21(3):169-80.

43. Bielsten T, Lasrado R, Keady J, Kullberg A, Hellström I. Living Life and Doing Things Together: Collaborative Research With Couples Where One Partner Has a Diagnosis of Dementia. Qualitative Health Research. 2018;28(11):1719-34.

44. Bodschwinna D, Weissflog G, Dohner H, Niederwieser D, Mehnert-Theuerkauf A, Gundel H, et al. Couples Coping With Hematological Cancer: Support Within and Outside the Couple - Findings From a Qualitative Analysis of Dyadic Interviews. Frontiers in psychology. 2022;13:855638.

45. Boothby CA, Santana MJ, Norris CM, Campbell TS, Rabi DM. Sexual activity after acute coronary syndrome: A qualitative approach to patient and partner experiences. Journal of Cardiovascular Nursing. 2021;36(5):E71-E9.

46. Borregaard Myrhoj C, Novrup Clemmensen S, Sax Rogind S, Jarden M, Toudal Viftrup D. Serious illness conversations in patients with multiple myeloma and their family caregivers-A qualitative interview study. European journal of cancer care. 2022;31(1):e13537.

47. Bosco A, Schneider J, Coleston-Shields DM, Orrell M. Narrative inquiry on case studies of crisis in dementia. Quality in Ageing & Older Adults. 2020;21(3):181-91.

48. Bragadottir GH, Halldorsdottir BS, Ingadottir TS, Jonsdottir H. Patients and families realising their future with chronic obstructive pulmonary disease-A qualitative study. Journal of Clinical Nursing (John Wiley & Sons, Inc). 2018;27(1-2):57-64.

49. Brignon M, Vioulac C, Boujut E, Delannoy C, Beauvais C, Kivits J, et al. Patients and relatives coping with inflammatory arthritis: Care teamwork. Health Expectations. 2020;23(1):137-47.

50. Buck HG, Hupcey J, Watach A. Pattern Versus Change: Community-Based Dyadic Heart Failure Self-Care. Clinical Nursing Research. 2018;27(2):148-61.

51. Bucki B, Spitz E, Baumann M. Emotional and social repercussions of stroke on patient-family caregiver dyads: Analysis of diverging attitudes and profiles of the differing dyads. PLoS ONE. 2019;14(4):e0215425.

52. Burke MM, Chung eun L, Hall SA, Rossetti Z, Lee CE. Understanding Decision Making Among Individuals With Intellectual and Developmental Disabilities (IDD) and Their Siblings. Intellectual & Developmental Disabilities. 2019;57(1):26-41.

53. Chung C, Hwang E. Couples' experiences of breast cancer in Korea: a descriptive qualitative study. Cancer Nursing. 2012;35(3):211-20.

54. Clark IN, Baker FA, Tamplin J, Lee Y-EC, Cotton A, Stretton-Smith PA. "Doing Things Together Is What It's About": An Interpretative Phenomenological Analysis of the Experience of Group Therapeutic Songwriting From the Perspectives of People With Dementia and Their Family Caregivers. Frontiers in psychology. 2021;12:598979.

55. Clark IN, Stretton-Smith PA, Baker FA, Lee Y-EC, Tamplin J. "It's Feasible to Write a Song": A Feasibility Study Examining Group Therapeutic Songwriting for People Living With Dementia and Their Family Caregivers. Frontiers in psychology. 2020;11:1951.

56. Conway L, Wolverson E, Clarke C. Shared Experiences of Resilience Amongst Couples Where One Partner Is Living With Dementia-A Grounded Theory Study. Frontiers in Medicine. 2020;7:219.

57. Dahms R, Haesner M. Importance of music in biographies of people with dementia. Advances in gerontology = Uspekhi gerontologii. 2018;31(2):285-92.

58. Davies JC. Preserving the ‘‘us identity’’ through marriage commitment while living with early-stage dementia. Dementia (14713012). 2011;10(2):217-34.

59. de Boer M, Zeiler K, Slatman J. Sharing lives, sharing bodies: partners negotiating breast cancer experiences. Medicine, Health Care & Philosophy. 2019;22(2):253-65.

60. Donkers H, Vernooij-Dassen M, Van Der Veen D, Van Der Sanden MN, Graff M. Social participation perspectives of people with cognitive problems and their care-givers: a descriptive qualitative study. Ageing & Society. 2019;39(7):1485-511.

61. Elliott K-EJ, Scott JL, Monsour M, Nuwayhid F. Profiles of dyadic adjustment for advanced prostate cancer to inform couple-based intervention. Psychology & Health. 2015;30(11):1259-73.

62. Falke SI, Lawson L. Couples with diabetes and health-care providers: a grounded theory of preferential relating. Health Expectations. 2015;18(6):3136-46.

63. Farina N, Williams A, Clarke K, Hughes LJ, Thomas S, Lowry RG, et al. Barriers, motivators and facilitators of physical activity in people with dementia and their family carers in England: dyadic interviews. Aging & Mental Health. 2021;25(6):1115-24.

64. Fasse L, Flahault C, Vioulac C, Lamore K, Van Wersch A, Quintard B, et al. The decision-making process for breast reconstruction after cancer surgery: Representations of heterosexual couples in long-standing relationships. British Journal of Health Psychology. 2017;22(2):254-69.

65. Ferguson CC, Jung SE, Lawrence JC, Douglas JW, Halli-Tierney A, Bui C, et al. A Mixed Methods Exploration of the Impact of the COVID-19 Pandemic on Food-Related Activities and Diet Quality in People with Parkinson Disease. International Journal of Environmental Research and Public Health. 2022;19(18):11741.

66. Freeman C, Cassidy B, Hay-Smith E. Couple's Experiences of Relationship Maintenance and Intimacy in Acute Spinal Cord Injury Rehabilitation: An Interpretative Phenomenological Analysis. Sexuality & Disability. 2017;35(4):433-44.

67. Frontini R, Sousa H, Ribeiro O, Figueiredo D. "What do we fear the most?": Exploring fears and concerns of patients, family members and dyads in end-stage renal disease. Scandinavian journal of caring sciences. 2021;35(4):1216-25.

68. Gallagher E, Beard RL. Buffer or Blade: Perceived relationship closeness in couples navigating Alzheimer's. Journal of aging studies. 2020;52:100832.

69. Gofton TE, Chum M, Schulz V, Gofton BT, Sarpal A, Watling C. Challenges facing palliative neurology practice: A qualitative analysis. Journal of the Neurological Sciences. 2018;385:225-31.

70. Golan M, Vilchinsky N, Wolf H, Abuhazira M, Ben-Gal T, Naimark A. Couples' Coping Strategies with Left Ventricular Assist Device Implantation: A Qualitative Dyadic Study. Qualitative Health Research. 2023;33(8/9):741-52.

71. Gong J, Chen M, Cao Q, Lin Y, Loke AY, Li Q. A qualitative study about colorectal cancer patients and spousal caregivers' experience and needs during COVID-19: implications for self-efficacy intervention. Asia-Pacific journal of oncology nursing. 2023;10(2):100179.

72. Grunberg VA, Bannon SM, Popok P, Reichman M, Dickerson BC, Vranceanu A-M. A race against time: couples' lived diagnostic journeys to young-onset dementia. Aging & Mental Health. 2022;26(11):2223-32.

73. Grunberg VA, Bannon SM, Reichman M, Popok PJ, Vranceanu A-M. Psychosocial treatment preferences of persons living with young-onset dementia and their partners. Dementia (14713012). 2022;21(1):41-60.

74. Habermann B, Shin JY, Shearer G. Dyadic Decision-Making in Advanced Parkinson's Disease: A Mixed Methods Study. Western Journal of Nursing Research. 2020;42(5):348-55.

75. Hallward L, Chemtob K, Lambert SD, Duncan LR. Prostate cancer survivors' and caregivers' experiences using behavior change techniques during a web-based self-management and physical activity program: A qualitative study. Journal of Clinical Medicine. 2020;9(10):1-16.

76. Halpin SN, Dillard RL, Puentes WJ. Socio-Emotional Adaptation Theory: Charting the Emotional Process of Alzheimer's Disease. Gerontologist. 2017;57(4):696-706.

77. Haywood A, Barnes S, Marsh H, Parker SG. Does the Design of Settings Where Acute Care Is Delivered Meet the Needs of Older People? Perspectives of Patients, Family Carers, and Staff. Health Environments Research & Design Journal (HERD) (Sage Publications, Ltd). 2018;11(2):177-88.

78. Heffernan B, Forbes E, Seeberger L, Shattuck J, Cook M, Ayele R, et al. Perceptions of palliative care in Huntington's disease: A qualitative study. Parkinsonism and Related Disorders. 2024;120:106007.

79. Hellqvist C, Berterö C. Support supplied by Parkinson's disease specialist nurses to Parkinson's disease patients and their spouses. Applied Nursing Research. 2015;28(2):86-91.

80. Hernandez E, Spencer B, Ingersoll-Dayton B, Faber A, Ewert A. "We are a team": Couple identity and memory loss. Dementia: The International Journal of Social Research and Practice. 2019;18(3):1166-80.

81. Hersh D, Kong SJ, Smith J. It's quite good fun: A qualitative study of a singing/songwriting programme for people with Parkinson's disease and their spouses. International Journal of Language & Communication Disorders. 2023;58(6):2103-16.

82. Hewetson R, Cornwell P, Shum DHK. Relationship and Social Network Change in People With Impaired Social Cognition Post Right Hemisphere Stroke. American Journal of Speech-Language Pathology. 2021;30:962-73.

83. Hickman R, Zdrodowska MA, Kellner S, Cersonsky TEK, Trujillo Diaz D, Louis ED, et al. The "Caring Giver": Emotional Caregiving in the Setting of Essential Tremor. Research on Aging. 2020;42(2):83-91.

84. Hillebregt C, Trappenburg M, Tonkens E. ‘Let us be’. Social support needs of people with acquired long‐term disabilities and their caregivers in rehabilitation practice in the Netherlands. Health & Social Care in the Community. 2022;30(6):e4384-e94.

85. Hoel V, Wolf-Ostermann K, Ambugo EA. Social Isolation and the Use of Technology in Caregiving Dyads Living With Dementia During COVID-19 Restrictions. Frontiers in public health. 2022;10:697496.

86. Horn BL, Albers EA, Mitchell LL, Jutkowitz E, Finlay JM, Millenbah AN, et al. Can Technology-Based Social Memory Aids Improve Social Engagement? Perceptions of a Novel Memory Aid for Persons With Memory Concerns. Journal of Applied Gerontology. 2023;42(3):399-408.

87. Horning MA, Shin JY, DiFusco LA, Norton M, Habermann B. Symptom progression in advanced Parkinson's disease: Dyadic perspectives. Applied nursing research : ANR. 2019;50:151193.

88. Huang S, Zhao Q, Liu X, Jin Y. The role of care-giver and mental health for older adults with disabilities: a mixed-method study. Ageing & Society. 2023;43(10):2239-63.

89. Hudson J, Reblin M, Clayton MF, Ellington L. Addressing cancer patient and caregiver role transitions during home hospice nursing care. Palliative & Supportive Care. 2019;17(5):523-30.

90. HvaliČ-Touzery S, Smole-Orehek K, Dolničar V. EXPLORING RECIPROCITY IN PERCEPTIONS ON TELECARE WITHIN THE INFORMAL CARER-CARE RECEIVER DYAD **. Teorija in Praksa. 2021;58(3):840-59.

91. Isac C, Lee P. Transitional care for older adults with chronic illness: A qualitative inquiry. International journal of older people nursing. 2024;19(1):e12599.

92. Isenberg SR, Killackey T, Saunders S, Scott M, Ernecoff NC, Bush SH, et al. "Going Home [Is] Just a Feel-Good Idea With No Structure": A Qualitative Exploration of Patient and Family Caregiver Needs When Transitioning From Hospital to Home in Palliative Care. Journal of Pain & Symptom Management. 2021;62(3):e9-e19.

93. Johnston L, Terp DM. Dynamics in Couples Facing Early Alzheimer’s Disease. Clinical Gerontologist. 2015;38(4):283-301.

94. Jones KF, Dorsett P, Simpson G, Briggs L. Moving forward on the journey: Spirituality and family resilience after spinal cord injury. Rehabilitation Psychology. 2018;63(4):521-31.

95. Ju E, Burton C, Kim J, Guo Y, Park JI, Qu A, et al. Sleep disturbances and interrelationship between persons with dementia and family caregivers: The lived experiences of Korean American Dyads. Geriatric Nursing. 2024;55:144-51.

96. Kim SW, Langer S, Ahern M, Larkey L, Todd M, Martin D, et al. Hematopoietic Cell Transplantation Patient-Caregiver Dyad Perspectives on Participation in a Digital Storytelling Intervention: A Qualitative Approach. Transplantation and Cellular Therapy. 2023;29(8):520.e1-.e7.

97. Kwan CML, Chun KM, Huang P, Chesla CA. Concerns About Professional Chinese Medicine Among Chinese Immigrants With Type 2 Diabetes. Diabetes Spectrum. 2013;26(4):247-53.

98. Lambert SD, Duncan LR, Ellis J, Schaffler JL, Loban E, Robinson JW, et al. Acceptability and usefulness of a dyadic, tailored, web-based, psychosocial and physical activity self-management program (Tempo): A qualitative study. Journal of Clinical Medicine. 2020;9(10):1-24.

99. Lamore K, Vioulac C, Fasse L, Flahault C, Quintard B, Untas A. Couples' Experience of the Decision-Making Process in Breast Reconstruction After Breast Cancer: A Lexical Analysis of Their Discourse. Cancer Nursing. 2020;43(5):384-95.

100. Laranjeira C, Dixe MA, Semeao I, Rijo S, Faria C, Querido A. "Keeping the Light On": A Qualitative Study on Hope Perceptions at the End of Life in Portuguese Family Dyads. International Journal of Environmental Research and Public Health. 2022;19(3):1561.

101. Leavey G, Corry DS, Waterhouse-Bradley B, Curran E, Todd S, McIlfatrick S, et al. Acceptability and use of a patient-held communication tool for people living with dementia: a longitudinal qualitative study. BMJ open. 2020;10(5):e036249.

102. Lee K, Park M. Keeping care fully alive-An ethnography of Moving-with carers and persons living with dementia. Journal of Aging Studies. 2021;57:1-9.

103. Lee SC, Marks EG, Sanders JM, Wiebe DJ. Elucidating patient-perceived role in "decision-making" among African Americans receiving lung cancer care through a county safety-net system. Journal of cancer survivorship : research and practice. 2016;10(1):153-63.

104. Lehmkuhl L, Munck MS, Rothmann MJ, Sorknaes AD. Exploring critically ill patients' and their relatives' experiences of intensive care unit during COVID-19: A qualitative study. Nursing in critical care. 2024.

105. Lelaka CM, Moyo I, Tshivhase L, Mavhandu-Mudzusi AH. Psychosocial support for HIV serodiscordant couples. Health Psychology and Behavioral Medicine. 2022;10(1):537-56.

106. Li Q, Lin Y, Chen Y, Loke AY. Mutual Support and Challenges Among Chinese Couples Living With Colorectal Cancer: A Qualitative Study. Cancer Nursing. 2018;41(5):E50-E60.

107. Lingard RJ, Court J. Can Couples Find a Silver Lining Amid the Dark Cloud of ME/CFS: A Pilot Study. The Family Journal: Counseling and Therapy for Couples and Families. 2014;22(3):304-10.

108. LoBuono DL, Shea KS, Reed M, Tovar A, Leedahl SN, Xu F, et al. The Facilitators and Barriers to Digital Health for Managing Nutrition in People With Parkinson's Disease and Their Caregivers: A Formative, Qualitative Study. Journal of Nutrition Education & Behavior. 2023;55(8):553-63.

109. LoBuono DL, Shea KS, Tovar A, Leedahl SN, Mahler L, Xu F, et al. Acceptance and perception of digital health for managing nutrition in people with Parkinson's disease and their caregivers and their digital competence in the United States: A mixed-methods study. Health Science Reports. 2021;4(4):e412.

110. LoBuono DL, Shea KS, Tovar A, Leedahl SN, Mahler L, Xu F, et al. Diet Quality and Nutrition Concerns of People with Parkinson's Disease and Their Informal Caregivers: A Mixed Methods Study. Journal of nutrition in gerontology and geriatrics. 2022;41(1):1-21.

111. Lou S, Carstensen K, Møldrup M, Shahla S, Zakharia E, Nielsen CP. Early supported discharge following mild stroke: a qualitative study of patients' and their partners' experiences of rehabilitation at home. Scandinavian Journal of Caring Sciences. 2017;31(2):302-11.

112. Maisel NC, Rauer AJ, Marshall GN, Karney BR. Predicting support from an intimate partner after a traumatic injury. Journal of Applied Social Psychology. 2011;41(8):2044-75.

113. Manne S, Etz RS, Hudson SV, Medina-Forrester A, Boscarino JA, Bowen DJ, et al. A qualitative analysis of couples' communication regarding colorectal cancer screening using the Interdependence Model. Patient Education & Counseling. 2012;87(1):18-22.

114. Mastwyk M, Ames D, Ellis KA, Chiu E, Dow B. Disclosing a dementia diagnosis: what do patients and family consider important? International Psychogeriatrics. 2014;26(8):1263-72.

115. Matchar BG, Gwyther LP. Resilience in Early-Stage Dementia. Topics in Geriatric Rehabilitation. 2014;30(3):170-5.

116. Mathur A, Hoffman AS, Weston J, Crocker LC, Holman DA, Bradford A, et al. How do couples and spouses/partners consider fertility preservation decisions during cancer treatment planning? A qualitative analysis of dyadic decision making. Journal of Psychosocial Oncology. 2021;39(2):268-84.

117. Mattos MK, Nilsen ML, Lingler JH. Experiences Surrounding an Early-Stage Cognitive Diagnosis in Rural-Dwelling Older Adults. Research in Gerontological Nursing. 2018;11(4):181-9.

118. Maxted C, Simpson J, Weatherhead S. An Exploration of the Experience of Huntington's Disease in Family Dyads: An Interpretative Phenomenological Analysis. Journal of Genetic Counseling. 2014;23(3):339-49.

119. McCarthy MJ, Craddock WS, Acquavita SP, Black K. A mixed-methods study of smoking attitudes and behaviors among dual-smoker stroke survivor–caregiver dyads. Journal of Health Psychology. 2018;23(13):1659-67.

120. McCarthy MJ, Lyons KS, Powers LE. Relational Factors Associated With Depressive Symptoms Among Stroke Survivor-Spouse Dyads. Journal of Family Social Work. 2012;15(4):303-20.

121. McCaughan E, McKenna S, McSorley O, Parahoo K. The experience and perceptions of men with prostate cancer and their partners of the CONNECT psychosocial intervention: a qualitative exploration. Journal of Advanced Nursing (John Wiley & Sons, Inc). 2015;71(8):1871-82.

122. McCurley JL, Funes CJ, Zale EL, Lin A, Jacobo M, Jacobs JM, et al. Preventing Chronic Emotional Distress in Stroke Survivors and Their Informal Caregivers. Neurocritical Care. 2019;30(3):581-9.

123. McDonnell KK, Bullock LF, Kozower BD, Hollen PJ, Heath J, Rovnyak V. A Decision Aid to Improve Smoking Abstinence for Families Facing Cancer. Oncology Nursing Forum. 2014;41(6):649-58.

124. McGovern J. Capturing the significance of place in the lived experience of dementia. Qualitative Social Work. 2017;16(5):664.

125. McMahon K, McFerran K, Clark IN, Odell-Miller H, Stensaeth K, Tamplin J, et al. Learning to use music as a resource: the experiences of people with dementia and their family care partners participating in a home-based skill-sharing music intervention: a HOMESIDE sub-study. Frontiers in Medicine. 2023;10:1205784.

126. McParland J, Camic PM. How do lesbian and gay people experience dementia? Dementia (London, England). 2018;17(4):452-77.

127. McPhee DP, Robinson WD. Couples Living with Chronic Migraines: A Phenomenological Study. Contemporary Family Therapy: An International Journal. 2020;42(3):271-83.

128. Meeker MA, Waldrop DP, Schneider J, Case AA. Contending with advanced illness: patient and caregiver perspectives. Journal of Pain & Symptom Management. 2014;47(5):887-95.

129. Merrick K, Camic PM, O’Shaughnessy M. Couples constructing their experiences of dementia: A relational perspective. Dementia (14713012). 2016;15(1):34-50.

130. Mounce G, Allan HT, Carey N. 'Just have some IVF!': A longitudinal ethnographic study of couples' experiences of seeking fertility treatment. Sociology of Health & Illness. 2022;44(2):308-27.

131. Mowll J, Lobb EA, Lane L, Lacey J, Chochinov HM, Kelly B, et al. A preliminary study to develop an intervention to facilitate communication between couples in advanced cancer. Palliative & Supportive Care. 2015;13(5):1381-90.

132. Myrhøj CB, Viftrup DT, Jarden M, Clemmensen SN. Interdisciplinary collaboration in serious illness conversations in patients with multiple myeloma and caregivers – a qualitative study. BMC Palliative Care. 2023;22(1):1-12.

133. Nelson KE, Saylor MA, Anderson A, Buck H, Davidson PM, DeGroot L, et al. "We're all we got is each other": Mixed-methods analysis of patient-caregiver dyads' management of heart failure. Heart & Lung. 2022;55:24-8.

134. Niedling K, Hamel K. Longing for normalcy in couple relationships: How chronic illness and care dependency change the relationship of long-married couples. Frontiers in public health. 2023;11:1117786.

135. Nielsen AH, Egerod I, Hansen TB, Angel S. Intensive care unit diaries: Developing a shared story strengthens relationships between critically ill patients and their relatives: A hermeneutic-phenomenological study. International journal of nursing studies. 2019;92:90-6.

136. Nielsen IH, Poulsen I, Larsen K, Larsen NS. Life goals as a driving force in traumatic brain injury rehabilitation: a longitudinal dyadic perspective. Brain Injury. 2022;36(9):1158-66.

137. Nimbley E, Gillespie-Smith K, Duffy F, Maloney E, Ballantyne C, Sharpe H. "It's not about wanting to be thin or look small, it's about the way it feels": an IPA analysis of social and sensory differences in autistic and non-autistic individuals with anorexia and their parents. Journal of eating disorders. 2023;11(1):89.

138. O'Connor CM, Clemson L, Brodaty H, Low L-F, Jeon Y-H, Gitlin LN, et al. The tailored activity program (TAP) to address behavioral disturbances in frontotemporal dementia: a feasibility and pilot study. Disability & Rehabilitation. 2019;41(3):299-310.

139. O'Leary AM, Landers AL, Jackson JB. "I'm fighting with BPD instead of my partner": A dyadic interpretative phenomenological analysis of the lived experience of couples navigating borderline personality disorder. Journal of marital and family therapy. 2024;50(1):45-70.

140. Olthof-Nefkens Maria WLJ, Kruse H, Derksen E, de Swart Bert JM, Nijhuis-van der Sanden Maria WG, Kalf Johanna G. Improving Communication between Persons with Mild Dementia and Their Caregivers: Qualitative Analysis of a Practice-Based Logopaedic Intervention. Folia Phoniatrica et Logopaedica. 2018;70(3/4):124-33.

141. Opsomer S, Joossens S, De Wit C, Lauwerier E, Pype P. Losing Health Symbols Because of Nutrition-Related Problems in Advanced Cancer: An Interpretative Phenomenological Analysis. Journal of Hospice & Palliative Nursing. 2018;20(5):492-9.

142. Opsomer S, Joossens S, De Wit C, Lauwerier E, Pype P. Couples coping with nutrition-related problems in advanced cancer: A qualitative study in primary care. European Journal of Oncology Nursing. 2019;38:76-84.

143. Overgaard D, Kaldan G, Marsaa K, Nielsen TL, Shaker SB, Egerod I. The lived experience with idiopathic pulmonary fibrosis: A qualitative study. European Respiratory Journal. 2016;47(5):1472-80.

144. Parker AM, Nelliot A, Chessare CM, Malik AM, Koneru M, Hosey MM, et al. Usability and acceptability of a mobile application prototype for a combined behavioural activation and physical rehabilitation intervention in acute respiratory failure survivors. Australian Critical Care. 2020;33(6):511-7.

145. Patinadan PV, Tan-Ho G, Choo PY, Low CX, Ho AHY. 'Food for Life and Palliation (FLiP)': A qualitative study for understanding and empowering dignity and identity for terminally ill patients in Asia. BMJ Open. 2021;11(4):e038914.

146. Patterson JM, Rapley T, Carding PN, Wilson JA, McColl E. Head and neck cancer and dysphagia; caring for carers. Psycho-Oncology. 2013;22(8):1815-20.

147. Peach T, Pollock K, Van Der Wardt V, Das Nair R, Logan P, Harwood RH. Attitudes of older people with mild dementia and mild cognitive impairment and their relatives about falls risk and prevention: A qualitative study. PLoS ONE. 2017;12(5):e0177530.

148. Peak T, Gast J, Novak JR. Caregiving and caring with pride: Health behavior work among older gay married couples. Journal of Gay & Lesbian Social Services: The Quarterly Journal of Community & Clinical Practice. 2021;33(1):123-36.

149. Pedersen M, Overgaard D, Andersen I, Baastrup M, Egerod I. Experience of exclusion: A framework analysis of socioeconomic factors affecting cardiac rehabilitation participation among patients with acute coronary syndrome. European Journal of Cardiovascular Nursing. 2017;16(8):715-23.

150. Pedersen M, Støier L, Egerod I, Overgaard D. Mastery of everyday life and social support needs in older vulnerable women with myocardial infarction and their relatives: a qualitative study. European Journal of Cardiovascular Nursing. 2021;20(7):641-7.

151. Perngmark P, Waebuesa N, Holroyd E. Collaborative Approaches to Promote Family Caregiving for Thai-Muslim Older Adults. Journal of Holistic Nursing. 2023;41(1):17-29.

152. Petersen JJ, Ostergaard B, Svavarsdottir EK, Rosenstock SJ, Brodsgaard A. A challenging journey: The experience of elderly patients and their close family members after major emergency abdominal surgery. Scandinavian journal of caring sciences. 2021;35(3):901-10.

153. Pickering J, Crooks VA, Snyder J, Milner T. Relational, community‐based and practical: Support systems used by Canadian spousal caregivers living seasonally in the United States. Health & Social Care in the Community. 2022;30(6):2311-9.

154. Pisu M, Demark-Wahnefried W, Kenzik KM, Oster RA, Lin CP, Manne S, et al. A dance intervention for cancer survivors and their partners (RHYTHM). Journal of Cancer Survivorship. 2017;11(3):350-9.

155. Porter LS, Weiner DK, Ramos K, Barnes DE, Schmader KE, Gwyther L, et al. Partnering to cope with pain: A pilot study of a caregiver-assisted pain coping skills intervention for patients with cognitive impairment and dementia. Palliative & supportive care. 2022;20(6):785-93.

156. Puaschitz NGS, Jacobsen FF, Berge LI, Husebo BS. Access to, use of, and experiences with social alarms in home-living people with dementia: results from the LIVE@Home.Path trial. Frontiers in Aging Neuroscience. 2023:1-11.

157. Rapaport P, Burton A, Palomo M, Griffiths J, Kelleher D, Leverton M, et al. A mixed-methods feasibility study of a goal-focused manualised intervention to support people with dementia to stay living independently at home with support from family carers: NIDUS (New Interventions for Independence in Dementia Study) Family. Aging & Mental Health. 2021;25(8):1463-74.

158. Rauer A, Cooke WM, Haselschwerdt M, Winters-Stone K, Hornbuckle L. From Organizing Medicine to Cooking With More Leafy Greens: A Dyadic, Qualitative Analysis of How Older African American Couples Take Care of Each Other's Health. Research on aging. 2024:1640275241227557.

159. Riekkola J, Rutberg S, Lilja M, Isaksson G. Strategies of older couples to sustain togetherness. Journal of aging studies. 2019;48:60-6.

160. Riffin C, Van Ness PH, Iannone L, Fried T. Patient and Caregiver Perspectives on Managing Multiple Health Conditions. Journal of the American Geriatrics Society. 2018;66(10):1992-7.

161. Robinson CA. 'Our best hope is a cure.' Hope in the context of advance care planning. Palliative & Supportive Care. 2012;10(2):75-82.

162. Roelofs TSM, Luijkx KG, Embregts PJCM. Love, Intimacy and Sexuality in Residential Dementia Care: A Client Perspective. Clinical Gerontologist. 2021;44(3):288-98.

163. Rosenstrom S, Risom SS, Hove JD, Brodsgaard A. Living with Atrial Fibrillation: A Family Perspective. Nursing research and practice. 2022;2022:7394445.

164. Ryan T, McKeown J. Couples affected by dementia and their experiences of advance care planning: A grounded theory study. Ageing & Society. 2020;40(2):439-60.

165. Scherrer KS, Ingersoll-Dayton B, Spencer B. Constructing couples' stories: Narrative practice insights from a dyadic dementia intervention. Clinical Social Work Journal. 2014;42(1):90-100.

166. Schunk M, Schulze F, Bausewein C. What Constitutes Good Health Care for Patients with Breathlessness? Perspectives of Patients, Caregivers, and Health Care Professionals. Journal of Palliative Medicine. 2019;22(6):656-62.

167. Sharabi LL, Delaney AL, Knobloch LK. In their own words. Journal of Social & Personal Relationships. 2016;33(4):421-48.

168. Simpson DB, Jose K, English C, Gall SL, Breslin M, Callisaya ML. "Factors influencing sedentary time and physical activity early after stroke: a qualitative study". Disability & Rehabilitation. 2022;44(14):3501-9.

169. Sinclair C, Gersbach K, Hogan M, Blake M, Bucks R, Auret K, et al. "A Real Bucket of Worms": Views of People Living with Dementia and Family Members on Supported Decision-Making. Journal of Bioethical Inquiry. 2019;16(4):587-608.

170. Soto SH, Berry DC, Callahan LF. Qualitative Exploration of Dyadic Influence on Physical Activity Between Latina Patients With Osteoarthritis and a Supporter of Their Physical Activity. Arthritis Care and Research. 2022;74(2):281-90.

171. Sousa H, Ribeiro O, Figueiredo D. End-stage renal disease is not yours, is not mine, is OURS: Exploring couples lived experiences through dyadic interviews. Hemodialysis International. 2021;25(3):361-71.

172. Sowden E, Hossain M, Chew-Graham C, Blakeman T, Tierney S, Wellwood I, et al. Understanding the management of heart failure with preserved ejection fraction: a qualitative multiperspective study. British Journal of General Practice. 2020;70(701):e880-e9.

173. Stamou V, La Fontaine J, O'Malley M, Jones B, Parkes J, Carter J, et al. Helpful post‐diagnostic services for young onset dementia: Findings and recommendations from the Angela project. Health & Social Care in the Community. 2022;30(1):142-53.

174. Stenhouse E, Letherby G, Stephen N. Women with pre-existing diabetes and their experiences of maternity care services. Midwifery. 2013;29(2):148-53.

175. Stockwell-Smith G, Moyle W, Kellett U. The impact of early-stage dementia on community-dwelling care recipient/carer dyads' capacity to self-manage. Journal of clinical nursing. 2019;28(3-4):629-40.

176. Stolee P, Ashbourne J, Elliott J, Main S, Holland N, Edick C, et al. Whole Person, Whole Journey: Developing a Person-Centred Regional Dementia Strategy. Canadian journal on aging = La revue canadienne du vieillissement. 2021;40(3):436-50.

177. Sun L, Liu J-E, Ji M, Wang Y, Chen S, Wang L. Coping with multiple chronic conditions among Chinese older couples: A community of shared destiny. Geriatric Nursing. 2022;48:214-23.

178. Sweeney L, Wolverson E, Clarke C. Understanding the shared experiences of creating a digital life story with individuals with dementia and their spouse. Dementia (14713012). 2021;20(5):1791-813.

179. Tan-Ho G, Choo PY, Patinadan PV, Low CX, Ho AHY. Blessings or burdens: an Interpretative Phenomenological Analysis (IPA) study on the motivations and their impact on end-of-life caregiving among Asian family caregivers. BMC Palliative Care. 2020;19(1):N.PAG-N.PAG.

180. Tankha H, Cano A, Corley A, Dillaway H, Lumley MA, Clark S. A novel couple-based intervention for chronic pain and relationship distress: A pilot study. Couple and Family Psychology: Research and Practice. 2020;9(1):13-32.

181. Tankha H, Caño A, Dillaway H. "Now I have hope": Rebuilding relationships affected by chronic pain. Families, Systems & Health: The Journal of Collaborative Family HealthCare. 2020;38(1):51-6.

182. Tarran-Jones A, Summers SJ, Dexter-Smith S, Craven-Staines S. Team psychological formulation to create a shared understanding of distress: a qualitative study in an older people's mental health inpatient setting. Quality in Ageing & Older Adults. 2019;20(2):67-79.

183. Tate AM, Martire LM, Zhaoyang R. Spousal understanding and marital satisfaction in pain patients and their spouses. Personal Relationships. 2019;26(1):42-53.

184. Tchakounte C, Nkenfou CN, Tchouangueu TF, Ngoufack NM, Tchuandom SB, Ngono OD, et al. HIV Serodiscordance among Couples in Cameroon: Effects on Sexual and Reproductive Health. International journal of MCH and AIDS. 2020;9(3):330-6.

185. Thomeer MB, Clark KO. The development of gendered health-related support dynamics over the course of a marriage. Journal of Women & Aging. 2021;33(2):153-69.

186. Thomson R, Martin JL, Sharples S. The experience of couples being given an oxygen concentrator to use at home: A longitudinal interpretative phenomenological analysis. Journal of health psychology. 2017;22(6):798-810.

187. Timm A, Kragelund Nielsen K, Alvesson HM, Jensen DM, Maindal HT. Motivation for Behavior Change among Women with Recent Gestational Diabetes and Their Partners—A Qualitative Investigation among Participants in the Face-It Intervention. Nutrients. 2023;15(18):3906.

188. Timmermans L, Boeykens D, Sirimsi MM, Decat P, Foulon V, Van Hecke A, et al. Self-management support in flemish primary care practice: the development of a preliminary conceptual model using a qualitative approach. BMC Primary Care. 2022;23(1):1-12.

189. Titler MG, Shuman C, Dockham B, Harris M, Northouse L. Acceptability of a Dyadic Psychoeducational Intervention for Patients and Caregivers. Oncology Nursing Forum. 2020;47(3):342-51.

190. Toft BS, Rodkjaer LO, Sorensen L, Saugbjerg MR, Bekker HL, Modrau IS. Feasibility of early digital health rehabilitation after cardiac surgery in the elderly: a qualitative study. BMC health services research. 2024;24(1):113.

191. Torge CJ. "Being in-between": Spouses that cohabit with and provide care for their partners in nursing homes. Journal of Applied Gerontology. 2020;39(4):377-84.

192. Tu J, Liu Y, Wu X, Xu D, Liao J. Dyadic appraisal and coping with illness among older Chinese adults with type 2 diabetes mellitus: a qualitative study. Age & Ageing. 2021;50(3):928-35.

193. Unadkat S, Camic PM, Vella-Burrows T. Understanding the Experience of Group Singing for Couples Where One Partner Has a Diagnosis of Dementia. Gerontologist. 2017;57(3):469-78.

194. van Corven CTM, Bielderman A, Wijnen M, Leontjevas R, Lucassen PLBJ, Graff MJL, et al. Defining empowerment for older people living with dementia from multiple perspectives: A qualitative study. International Journal of Nursing Studies. 2021;114:N.PAG-N.PAG.

195. van Dam K, Gielissen M, Reijnders R, van der Poel A, Boon B. Experiences of Persons With Executive Dysfunction in Disability Care Using a Social Robot to Execute Daily Tasks and Increase the Feeling of Independence: Multiple-Case Study. JMIR rehabilitation and assistive technologies. 2022;9(4):e41313.

196. Vester LB, Dreyer P, Holm A, Lorentzen V. The experience of being a couple during an intensive care unit admission. Nursing in Critical Care. 2020;25(4):238-44.

197. Walker LM, Robinson JW. A description of heterosexual couples' sexual adjustment to androgen deprivation therapy for prostate cancer. Psycho-Oncology. 2011;20(8):880-8.

198. Wawrziczny E, Antoine P, Ducharme F, Kergoat M-J, Pasquier F. Couples' experiences with early-onset dementia: An interpretative phenomenological analysis of dyadic dynamics. Dementia (14713012). 2016;15(5):1082-99.

199. Wawrziczny E, Picard S, Buquet A, Traversac E, Puisieux F, Pasquier F, et al. Hypnosis intervention for couples confronted with Alzheimer's disease: Promising results of a first exploratory study. Journal of Alzheimer's Disease. 2022;89(4):1351-66.

200. Wilson M, Doyle J, Turner J, Nugent C, O'Sullivan D. Designing technology to support greater participation of people living with dementia in daily and meaningful activities. Digital health. 2024;10:20552076231222427.

201. Wolkowski A, Carr SM. Does respite care address the needs of palliative care service users and carers? Their perspectives and experiences. International Journal of Palliative Nursing. 2017;23(4):174-85.

202. Womack JL, Isaksson G, Lilja M. Care partner dyad strategies to support participation in community mobility. Scandinavian Journal of Occupational Therapy. 2016;23(3):220-9.

203. Wyatt C, Murray C, Davies J, Jomeen J. Postpartum psychosis and relationships: their mutual influence from the perspective of women and significant others. Journal of Reproductive and Infant Psychology. 2015;33(4):426-42.

204. Ye L, Mages MA, Jimison HB, Patel SR. Developing OurSleepKit: A couple-focused mHealth tool to support adherence to positive airway pressure treatment. Behavioral Sleep Medicine. 2022;20(6):695-705.

205. Ying LY, Wu LH, Loke AY. The experience of Chinese couples undergoing in vitro fertilization treatment: Perception of the treatment process and partner support. PLoS ONE. 2015;10(10):e0139691.

206. Yorgason JB, Roper SO, Wheeler B, Crane K, Byron R, Carpenter L, et al. Older couples' management of multiple-chronic illnesses: individual and shared perceptions and coping in Type 2 diabetes and osteoarthritis. Families, Systems & Health: The Journal of Collaborative Family HealthCare. 2010;28(1):30-47.

207. Yun-Hee J, Krein L, Simpson JM, Szanton SL, Clemson L, Naismith SL, et al. Feasibility and potential effects of interdisciplinary home-based reablement program (I-HARP) for people with cognitive and functional decline: a pilot trial. Aging & Mental Health. 2020;24(11):1916-25.

208. Zamanipoor Najafabadi AH, Van De Mortel JPM, Lobatto DJ, Brandsma DR, Peul WC, Biermasz N, et al. Unmet needs and recommendations to improve meningioma care through patient, partner, and health care provider input: A mixed-method study. Neuro-Oncology Practice. 2020;7(2):239-48.

**Interview type not specified**

209. Berk L, Warmenhoven F, Stiekema APM, van Oorsouw K, van Os J, de Vugt M, et al. Mindfulness-based intervention for people with dementia and their partners: Results of a mixed-methods study. Frontiers in Aging Neuroscience. 2019;11.

210. Brady J, Mouneimne M, Milaney K. Environmental and systems experiences of persons with spinal cord injury and their caregivers when transitioning from acute care to community living during the COVID-19 pandemic: a comparative case study. Spinal Cord Series and Cases. 2023;9(1):8.

211. Carlander I, Ternestedt BM, Sahlberg-Blom E, Hellstrom I, Sandberg J. Being me and being us in a family living close to death at home. Qualitative health research. 2011;21(5):683-95.

212. Collins AL, Love AW, Bloch S, Street AF, Duchesne GM, Dunai J, et al. Cognitive Existential Couple Therapy for newly diagnosed prostate cancer patients and their partners: a descriptive pilot study. Psycho-Oncology. 2013;22(2):465-9.

213. de Graaff FM, Francke AL, van den Muijsenbergh METC, van der Geest S. Understanding and improving communication and decision-making in palliative care for Turkish and Moroccan immigrants: a multiperspective study. Ethnicity & Health. 2012;17(4):363-84.

214. Di Lorito C, Bosco A, Godfrey M, Dunlop M, Lock J, Pollock K, et al. Mixed-Methods Study on Caregiver Strain, Quality of Life, and Perceived Health. Journal of Alzheimer's Disease. 2021;80(2):799-811.

215. Dionne-Odom JN, Kono A, Frost J, Jackson L, Ellis D, Ahmed A, et al. Translating and Testing the ENABLE: CHF-PC Concurrent Palliative Care Model for Older Adults with Heart Failure and Their Family Caregivers...Educate, Nurture, Advise, Before Life Ends. Journal of Palliative Medicine. 2014;17(9):995-1004.

216. Evans N, Boyd H, Harris N, Noonan K, Ingram T, Jarvis A, et al. The experience of using prompting technology from the perspective of people with Dementia and their primary carers. Aging & Mental Health. 2021;25(8):1433-41.

217. Gaveras EM, Kristiansen M, Worth A, Irshad T, Sheikh A. Social support for South Asian Muslim parents with life-limiting illness living in Scotland: A multiperspective qualitative study. BMJ Open. 2014;4(2):e004252.

218. Haley WE, Marino VR, Sheehan OC, Rhodes JD, Kissela B, Roth DL. Stroke Survivor and Family Caregiver Reports of Caregiver Engagement in Stroke Care. Rehabilitation Nursing. 2019;44(6):302-10.

219. Harle K, Borjeson S, Hallbook O, Myrelid P, Thylen I. Putting life on hold: A longitudinal phenomenological-hermeneutic study of living with [or close to someone with] an enterocutaneous fistula before and after reconstructive surgery. Journal of clinical nursing. 2023;32(15-16):4663-76.

220. Hsieh C-J, Yin P-F, Chiu C-Y, Hsiao Y-P, Hsiao Y-L. Support and Empowerment for Older Adult Spousal Caregiving of People with Mild and Moderate Dementia: A Participatory Action Research. Healthcare (Basel, Switzerland). 2022;10(3).

221. Hyden KF, Coats HL, Meek PM. Home-based palliative care: Perspectives of chronic obstructive pulmonary disease patients and their caregivers. Chronic Obstructive Pulmonary Diseases. 2020;7(4):327-35.

222. Killen A, Flynn D, O'Brien N, Taylor J-P. The feasibility and acceptability of a psychosocial intervention to support people with dementia with Lewy bodies and family care partners. Dementia (14713012). 2022;21(1):77-93.

223. Li X, Keady J, Ward R. Transforming lived places into the connected neighbourhood: a longitudinal narrative study of five couples where one partner has an early diagnosis of dementia. Ageing & Society. 2021;41(3):605-27.

224. McCarthy MJ, Garcia YE, Dunn DJ, Lyons KS, Bakas T. Development and validation of a quality of relationship intervention for stroke survivor-family caregiver dyads. Topics in Stroke Rehabilitation. 2020;27(4):305-15.

225. McIntyre M, Ehrlich C, Kendall E. Informal care management after traumatic brain injury: perspectives on informal carer workload and capacity. Disability & Rehabilitation. 2020;42(6):754-62.

226. McMullen CK, Schneider J, Altschuler A, Grant M, Hornbrook MC, Liljestrand P, et al. Caregivers as healthcare managers: health management activities, needs, and caregiving relationships for colorectal cancer survivors with ostomies. Supportive Care in Cancer. 2014;22(9):2401-8.

227. Mendez-Luck CA, Miranda J, Mangione CM, Yoon J, VanGarde A. The Juntos Pilot Study: A Diabetes Management Intervention for Latino Caregiving Dyads. Diabetes Educator. 2019;45(5):507-19.

228. Mesa‐Melgarejo L, Carreño Moreno S, Chaparro‐Diaz L, Quintero González LA, Garcia‐Quintero D, Carrillo‐Algarra AJ, et al. Effectiveness of a case management model for people with multimorbidity: Mixed methods study. Journal of Advanced Nursing (John Wiley & Sons, Inc). 2022;78(11):3830-46.

229. Meyer CJ, Koh SSH, Hill AJ, Conway ER, Ryan BJ, McKinnon ER, et al. Hear–Communicate–Remember: Feasibility of delivering an integrated intervention for family caregivers of people with dementia and hearing impairment via telehealth. Dementia (14713012). 2020;19(8):2671-701.

230. Mmeje O, Njoroge B, Akama E, Leddy A, Breitnauer B, Darbes L, et al. Perspectives of healthcare providers and HIV-affected individuals and couples during the development of a Safer Conception Counseling Toolkit in Kenya: Stigma, fears, and recommendations for the delivery of services. AIDS Care. 2016;28(6):750-7.

231. Moosvi K, Schoppee TM, Xavier S, Henderson K, Suarez ML, Yao Y, et al. Feasibility and Burden of Lay Caregivers Providing Daily Massages to Patients With Cancer Receiving Hospice and Palliative Care. American Journal of Hospice & Palliative Medicine. 2022;39(12):1475-83.

232. O'Connor S, Bouamrane M-M, O'Donnell CA, Mair FS. Barriers to Co-Designing Mobile Technology with Persons with Dementia and Their Carers. Studies in Health Technology & Informatics. 2016;225:1028-9.

233. Salehi V, Hanson N, Smith D, McCloskey R, Jarrett P, Veitch B. Modeling and analyzing hospital to home transition processes of frail older adults using the functional resonance analysis method (FRAM). Applied Ergonomics. 2021;93:N.PAG-N.PAG.

234. Smith KM, Scerpella D, Guo A, Hussain N, Colburn JL, Cotter VT, et al. Perceived Barriers and Facilitators of Implementing a Multicomponent Intervention to Improve Communication With Older Adults With and Without Dementia (SHARING Choices) in Primary Care: A Qualitative Study. Journal of Primary Care & Community Health. 2022:1-11.

235. Stern A, Valaitis R, Weir R, Jadad AR. Use of home telehealth in palliative cancer care: a case study. Journal of Telemedicine & Telecare. 2012;18(5):297-300.

236. Tamplin J, Clark IN, Lee Y-EC, Baker FA. Remini-Sing: A Feasibility Study of Therapeutic Group Singing to Support Relationship Quality and Wellbeing for Community-Dwelling People Living With Dementia and Their Family Caregivers. Frontiers in medicine. 2018;5:245.

237. Van Diest E, Oldenmenger WH, Eland M, Taal W. Evaluation of an online tool about the expected course of disease for glioblastoma patients - A qualitative study. Neuro-Oncology Practice. 2022;9(5):411-9.

238. Ware NC, Wyatt MA, Haberer JE, Baeten JM, Kintu A, Psaros C, et al. What's love got to do with it? Explaining adherence to oral antiretroviral pre-exposure prophylaxis for HIV-serodiscordant couples. JAIDS Journal of Acquired Immune Deficiency Syndromes. 2012;59(5):463-8.

239. Yates LA, Orgeta V, Phuong L, Spector A, Orrell M, Leung P. Field-testing phase of the development of individual cognitive stimulation therapy (iCST) for dementia. BMC Health Services Research. 2016;16:1-11.

240. Yea-Jyh C, Petrinec A, Stephenson PS, Radziewicz RM, Sheehan D. Home-Based Reiki by Informal Caregivers: A Mixed-Methods Pilot Study. Holistic Nursing Practice. 2023;37(5):285-97.

241. Zhou J, Chen X, Wang Z, Lin C, Zhao J, Loke AY, et al. Mutual communication processes within Chinese colorectal cancer patient-spousal caregiver dyads: A qualitative study of taking gender into account. European Journal of Oncology Nursing. 2023;67:N.PAG-N.PAG.

**No dyadic analysis of separate care partner interviews**

242. Abshire MA, Bidwell JT, Pavlovic N, DeGroot L, Mammos D, Larsen L, et al. picture is worth a thousand words: exploring the roles of caregivers and the home environment of ventricular assist device patients. European Journal of Cardiovascular Nursing. 2021;20(8):782-91.

243. Akyar I, Dionne-Odom JN, Bakitas MA. Using Patients and Their Caregivers Feedback to Develop ENABLE CHF-PC: An Early Palliative Care Intervention for Advanced Heart Failure. Journal of Palliative Care. 2019;34(2):103-10.

244. Anderson MA, Buffo C, Ketcher D, Nguyen H, MacKenzie JJ, Reblin M, et al. Applying the RISE Model of Resilience in Partners Post-Stroke: A Qualitative Analysis. Annals of Behavioral Medicine. 2022;56(3):270-81.

245. Anderson S, Keating N, Wilson D. Reconciling Marriage and Care after Stroke. Canadian Journal on Aging. 2017;36(3):386.

246. Anderson S, Keating NC, Wilson DM. Staying married after stroke: a constructivist grounded theory qualitative study. Topics in Stroke Rehabilitation. 2017;24(7):479-87.

247. Andréasson F, Mattsson T, Hanson E. ‘The balance in our relationship has changed’: everyday family living, couplehood and digital spaces in informal spousal care. Journal of Family Studies. 2023;29(2):719-37.

248. Anne-Claire D, Coarelli G, Heinzmann A, Verdon B, Manuella DL, Petit E, et al. End-of-Life Discussions With Patients and Caregivers Affected By Neurogenetic Diseases. Neurology Clinical practice. 2023;13(6):e200199.

249. Aramburu Alegría C, Larsen B. Contextual care of the patient following weight-loss surgery: Relational views and maintenance activities of couples. Journal of the American Association of Nurse Practitioners. 2017;29(1):17-25.

250. Armstrong MJ, Rastgardani T, Gagliardi AR, Marras C. The experience of off periods: Qualitative analysis of interviews with persons with Parkinson's and carepartners. Clinical Parkinsonism and Related Disorders. 2019;1:31-6.

251. Armstrong MJ, Rastgardani T, Gagliardi AR, Marras C. Impact of off Periods on Persons with Parkinson Disease and Care Partners: A Qualitative Study. Neurology: Clinical Practice. 2021;11(3):E232-E8.

252. Arshad H, Malik S. Exploring perception of chronic Hepatitis C: An idiographic case study. Pakistan Journal of Psychological Research. 2019;34(4):735-51.

253. Atta-Konadu E, Keller HH, Daly K. The food-related role shift experiences of spousal male care partners and their wives with dementia. Journal of Aging Studies. 2011;25(3):305-15.

254. Attard MC, Loupis Y, Togher L, Rose ML. Experiences of people with severe aphasia and spouses attending an Interdisciplinary Community Aphasia Group. Disability & Rehabilitation. 2020;42(10):1382-96.

255. Baber W, Chang CYM, Yates J, Dening T. The experience of apathy in dementia: A qualitative study. International Journal of Environmental Research and Public Health. 2021;18(6):3325.

256. Badr H, Herbert K, Reckson B, Rainey H, Sallam A, Gupta V. Unmet needs and relationship challenges of head and neck cancer patients and their spouses. Journal of Psychosocial Oncology. 2016;34(4):336-46.

257. Bélanger E, Couch E, Carroll MS, DePasquale N, Gadbois EA, Shepherd-Banigan M, et al. Advance directives among cognitively impaired persons who had an amyloid PET scan and their care partners: a mixed-methods study. BMC Palliative Care. 2022;21(1):1-25.

258. Bennich BB, Konradsen H, Renaberg TP, Boesen J, Wind G. The significance of home-based portable pump chemotherapy for family caregivers to newly diagnosed patients with acute myeloid leukemia: A qualitative thematic analysis. European journal of oncology nursing : the official journal of European Oncology Nursing Society. 2022;61:102200.

259. Berry CE, Montgomery SH, Santulli R, Cullinan A. Adapting the Serious Illness Conversation Guide for Dementia Care. The American journal of hospice & palliative care. 2023:10499091231200214.

260. Berry E, Davies M, Dempster M. Exploring the perceptions of emotional distress among couples living with Type 2 diabetes and among diabetes healthcare providers, and consideration of support needs. Diabetic Medicine. 2020;37(10):1669-78.

261. Bonds Johnson K, Epps FR, Song M, Lyons KS, Driessnack M. Using poetry as data to explore daily and formal care decision making within African American dementia dyads. Geriatric Nursing. 2021;42(4):919-25.

262. Bowers B, Pollock K, Barclay S. Simultaneously reassuring and unsettling: a longitudinal qualitative study of community anticipatory medication prescribing for older patients. Age & Ageing. 2022;51(12):1-11.

263. Breitkopf CR, Ridgeway JL, Asiedu GB, Carroll K, Tenney M, Jatoi A. Ovarian cancer patients' and their family members' perspectives on novel vaccine and virotherapy trials. Clinical Trials. 2016;13(6):660-4.

264. Breton M, Wankah P, Guillette M, Couturier Y, Belzile L, Gagnon D, et al. Multiple Perspectives Analysis of the Implementation of an Integrated Care Model for Older Adults in Quebec. International journal of integrated care. 2019;19(4):6.

265. Bristol AA, Litchman M, Berg C, Grigorian E, Small D, Glazener A, et al. Using Continuous Glucose Monitoring and Data Sharing to Encourage Collaboration Among Older Adults With Type 1 Diabetes and Their Care Partners: Qualitative Descriptive Study. JMIR nursing. 2023;6:e46627.

266. Buckland S, Kaminskiy E, Bright P. Individual and family experiences of loss after acquired brain injury: A multi-method investigation. Neuropsychological Rehabilitation. 2021;31(4):531-51.

267. Canzona MR, Fisher CL, Garcia DS, Dong T, Ledford CJW. Partner presence in clinical conversations about sexual health: Breast cancer survivors', partners', and providers' perspectives of triadic interactions. Journal of Psychosocial Oncology. 2023;41(2):166-81.

268. Caswell G, Seymour J, Crosby V, Hussain A, Manderson C, Farnan S, et al. Lung cancer diagnosed following an emergency admission: exploring patient and carer perspectives on delay in seeking help. Supportive Care in Cancer. 2017;25(7):2259-66.

269. Cawley D, Billings J, Oliver D, Kendall M, Pinnock H. Potential triggers for the holistic assessment of people with severe chronic obstructive pulmonary disease: analysis of multiperspective, serial qualitative interviews. BMJ supportive & palliative care. 2014;4(2):152-60.

270. Chapman F, McManus A. Hepatitis C treatment - Better outcomes through partner support. Australasian Medical Journal. 2012;5(11):585-8.

271. Chawak S, Chittem M, Dhillon H, Huilgol N, Butow P. Treatment-related communication experiences and expectations among Indian cancer patients receiving radiation therapy and their family members: A qualitative study. Patient Education & Counseling. 2022;105(9):2913-22.

272. Chen H, Habermann B. Ready or not: planning for health declines in couples with advanced multiple sclerosis. Journal of Neuroscience Nursing. 2013;45(1):38-43.

273. Chen L. Decisions for institutionalization among nursing home residents and their children in Shanghai. Qualitative health research. 2015;25(4):458-69.

274. Chen L, Xiao LD, Chamberlain D. Exploring the shared experiences of people with stroke and caregivers in preparedness to manage post‐discharge care: A hermeneutic study. Journal of Advanced Nursing (John Wiley & Sons, Inc). 2022;78(9):2983-99.

275. Chow EOW, Wong YY, Fok DYH, Liao X, Li C. Positive life stories of Stroke-Survivor's spousal caregiving in Hong Kong: Lessons for policy and practice. Social Science and Medicine. 2021;291:114476.

276. Chung J, Boyle J, Pretzer-Aboff I, Knoefel J, Young HM, Wheeler DC. Using a GPS Watch to Characterize Life-Space Mobility in Dementia: A Dyadic Case Study. Journal of Gerontological Nursing. 2021;47(10):15-22.

277. Churchill L, Pollock M, Lebedeva Y, Pasic N, Bryant D, Howard J, et al. Optimizing outpatient total hip arthroplasty: perspectives of key stakeholders. Canadian journal of surgery Journal canadien de chirurgie. 2018;61(6):370-6.

278. Clark S, Prescott T, Murphy G. The lived experiences of dementia in married couple relationships. Dementia (London, England). 2019;18(5):1727-39.

279. Close H, Sidhu K, Genn H, Ling J, Hawkins C. Qualitative investigation of patient and carer experiences of everyday legal needs towards end of life. BMC Palliative Care. 2021;20(1):1-13.

280. Coady V, Warren N, Bilkhu N, Ayton D. Preferences for rural specialist health care in the treatment of Parkinson's disease: exploring the role of community-based nursing specialists. Australian Journal of Primary Health. 2019;25(1):49-53.

281. Cole L, Drennan VM, Manthorpe J, Hatzidimitriadou E, Iliffe S. Experiences of intimate continence care and the impact on the family dyad relationship for people living at home with dementia and their co-resident family members. Dementia (14713012). 2022;21(5):1556-73.

282. Corbett CF, Combs EM, Wright PJ, Owens OL, Stringfellow I, Nguyen T, et al. Virtual home assistant use and perceptions of usefulness by older adults and support person dyads. International Journal of Environmental Research and Public Health. 2021;18(3):1-13.

283. Corney R, Puthussery S, Swinglehurst J. Couple relationships in families with dependent children after a diagnosis of maternal breast cancer in the United Kingdom: Perspectives from mothers and fathers. Journal of Psychosocial Oncology. 2016;34(5):413-31.

284. Cousins E, de Vries K, Harrison Dening K. ' Four walls and a garden ': Exploring the experiences of families affected by dementia during the COVID-19 pandemic. Dementia (14713012). 2022;21(3):810-29.

285. Cox CG, Ryan BAMM, Gillen DL, Grill JD. A Preliminary Study of Clinical Trial Enrollment Decisions Among People With Mild Cognitive Impairment and Their Study Partners. American Journal of Geriatric Psychiatry. 2019;27(3):322-32.

286. Croteau C, McMahon‐Morin P, Le Dorze G, Baril G. Impact of aphasia on communication in couples. International Journal of Language & Communication Disorders. 2020;55(4):547-57.

287. Crowley JL, Miller LE. "Who's gonna love a junkie? But he does": Exploring couples' identity negotiations and dyadic coping in the context of opioid use disorder. Journal of Social & Personal Relationships. 2020;37(5):1634-52.

288. Culley L, Law C, Hudson N, Mitchell H, Denny E, Raine-Fenning N. A qualitative study of the impact of endometriosis on male partners. Human Reproduction. 2017;32(8):1667-73.

289. Cup EHC, Kinebanian A, Satink T, Pieterse AJ, Hendricks HT, Oostendorp RAB, et al. Living with myotonic dystrophy; what can be learned from couples? A qualitative study. BMC Neurology. 2011;11.

290. Dainty KN, Bianca Seaton M, Richard Verbeek P. Moving from physical survival to psychologic recovery: a qualitative study of survivor perspectives on long-term outcome after sudden cardiac arrest. Resuscitation plus. 2021;5:100055.

291. Dale CM, McKim D, Amin R, Carbone S, Fisher T, Goldstein R, et al. Education Experiences of Adult Subjects and Caregivers for Mechanical Insufflation-Exsufflation at Home. Respiratory Care. 2020;65(12):1889-96.

292. Daveson BA, Harding R, Shipman C, Mason BL, Epiphaniou E, Higginson IJ, et al. The real-world problem of care coordination: A longitudinal qualitative study with patients living with advanced progressive illness and their unpaid caregivers. PLoS ONE. 2014;9(5):e95523.

293. De Vincentis G, Monari F, Baldari S, Salgarello M, Frantellizzi V, Salvi E, et al. Narrative medicine in metastatic prostate cancer reveals ways to improve patient awareness & quality of care. Future Oncology. 2018;14(27):2821-32.

294. Doucet S, Letourneau N, Blackmore ER. Support Needs of Mothers Who Experience Postpartum Psychosis and Their Partners. JOGNN: Journal of Obstetric, Gynecologic & Neonatal Nursing. 2012;41(2):236-45.

295. Dube A, Renju J, Wamoyi J, Hassan F, Seeley J, Chimukuche RS, et al. Consequences of male partner engagement policies on HIV care-seeking in three African countries: Findings from the SHAPE UTT study. Special Issue: Understanding the health systems impacts of "Test and Treat" in sub-Saharan Africa: Findings from the SHAPE study. 2021;16(2):216-26.

296. Dunning T, Harris JM, Sandall J. Women and their birth partners' experiences following a primary postpartum haemorrhage: a qualitative study. BMC pregnancy and childbirth. 2016;16:80.

297. Einerson J, Lundstrom LK, Allen BK, Sefandonakis A, Terrill AL. Learning to flourish in a new reality: a thematic analysis of couples' experience of participation in a positive psychology intervention post-stroke. Disability & Rehabilitation. 2023;45(16):2612-9.

298. Ek K, Ternestedt B-M, Andershed B, Sahlberg-Blom E. Shifting life rhythms: couples' stories about living together when one spouse has advanced chronic obstructive pulmonary disease. Journal of Palliative Care. 2011;27(3):189-97.

299. Ekstam L, Johansson U, Guidetti S, Eriksson G, Ytterberg C. The combined perceptions of people with stroke and their carers regarding rehabilitation needs 1 year after stroke: A mixed methods study. BMJ Open. 2015;5(2):e006784.

300. Emanuel N, Simon MA, Burt M, Joseph A, Sreekumar N, Kundu T, et al. Economic Impact of Terminal Illness and the Willingness to Change It. Journal of Palliative Medicine. 2010;13(8):941-4.

301. Engblom-Deglmann ML, Hamilton J. The Impact of Spinal Cord Injury on the Couple Relationship: A Grounded Theory Exploration of the Adjustment Process. Journal of Couple & Relationship Therapy. 2020;19(3):250-75.

302. Esandi N, Nolan M, Alfaro C, Canga-Armayor A. Keeping Things in Balance: Family Experiences of Living With Alzheimer's Disease. Gerontologist. 2018;58(2):e56-e67.

303. Evans N, Collier L. An exploration of the experience of using calendar reminders for people with dementia and family carers. Dementia: The International Journal of Social Research and Practice. 2019;18(5):1912-33.

304. Feeley N, Bell L, Hayton B, Zelkowitz P, Carrier M-E. Care for postpartum depression: What do women and their partners prefer? Perspectives in Psychiatric Care. 2016;52(2):120-30.

305. Fetherstonhaugh D, Rayner JA, Tarzia L. Hanging on to Some Autonomy in Decisionmaking: How do Spouse Carers Support this? Dementia (London, England). 2019;18(4):1219-36.

306. Fowler NR, Shaaban CE, Torke AM, Lane KA, Saba S, Barnato AE. "I'm Not Sure We Had A Choice": Decision Quality and The Use of Cardiac Implantable Electronic Devices In Older Adults With Cognitive Impairment. Cardiology and cardiovascular medicine. 2018;2(1):10-26.

307. Gadbois EA, Bélanger E, Shield RR, Plassman BL, Van Houtven CH, Wetle TF. "Now at least we have something to call it": Patient and care partner experiences receiving an amyloid PET scan. Journal of the American Geriatrics Society. 2022;70(10):2938-47.

308. Galimidi N, Shamai M. Interactions between mental illness recovery processes in the family. Family Relations: An Interdisciplinary Journal of Applied Family Studies. 2022;71(1):408-25.

309. Gately L, McLachlan SA, Dowling A, Philip J. Surviving glioblastoma and a sense of disconnection. Journal of Clinical Neuroscience. 2020;81:284-9.

310. Gawinski L, Stiel S, Schneider N, Herbst FA. Communication in dyads of adult children at the end of life with their parents and parents at the end of life with their adult children: Findings from a mixed-methods study. Psycho-Oncology. 2021;30(9):1535-43.

311. Genoe R, Dupuis SL, Keller HH, Martin LS, Cassolato C, Edward HG. Honouring identity through mealtimes in families living with dementia. Journal of Aging Studies. 2010;24(3):181-93.

312. George DE, Dholakia S, Tharyan P. Participation in randomised controlled trials: perspectives of psychiatric patients and key relatives. Indian journal of medical ethics. 2018;3(1):9-15.

313. Gettel CJ, Hayes K, Shield RR, Guthrie KM, Goldberg EM. Care Transition Decisions After a Fall-related Emergency Department Visit: A Qualitative Study of Patients' and Caregivers' Experiences. Academic Emergency Medicine. 2020;27(9):876-86.

314. Gibbons SW, Ross A, Wehrlen L, Klagholz S, Bevans M. Enhancing the cancer caregiving experience: Building resilience through role adjustment and mutuality. European journal of oncology nursing : the official journal of European Oncology Nursing Society. 2019;43:101663.

315. Gill P, Lowes L. Renal transplant failure and disenfranchised grief: Participants' experiences in the first year post-graft failure -- a qualitative longitudinal study. International Journal of Nursing Studies. 2014;51(9):1271-80.

316. Goldberg EM, Gettel CJ, Hayes K, Shield RR, Guthrie KM. GAPcare: The Geriatric Acute and Post-Acute Fall Prevention Intervention for Emergency Department Patients - A Qualitative Evaluation. OBM geriatrics. 2019;3(4).

317. Gomersall T, Smith SK, Blewett C, Astell A. 'It's definitely not Alzheimer's': Perceived benefits and drawbacks of a mild cognitive impairment diagnosis. British Journal of Health Psychology. 2017;22(4):786-804.

318. Granek L, Danan D, Bersudsky Y, Osher Y. Living with bipolar disorder: The impact on patients, spouses, and their marital relationship. Bipolar Disorders. 2016;18(2):192-9.

319. Granek L, Danan D, Bersudsky Y, Osher Y. Hold on tight: Coping strategies of persons with bipolar disorder and their partners. Family Relations: An Interdisciplinary Journal of Applied Family Studies. 2018;67(5):589-99.

320. Griffiths F, Mason V, Boardman F, Dennick K, Haywood K, Achten J, et al. Evaluating recovery following hip fracture: A qualitative interview study of what is important to patients. BMJ Open. 2015;5(1):e005406.

321. Grill JD, Cox CG, Kremen S, Mendez MF, Teng E, Shapira J, et al. Patient and caregiver reactions to clinical amyloid imaging. Alzheimer's and Dementia. 2017;13(8):924-32.

322. Groen-van de Ven L, Smits C, Oldewarris K, Span M, Jukema J, Eefsting J, et al. Decision Trajectories in Dementia Care Networks: Decisions and Related Key Events. Research on Aging. 2017;39(9):1039-71.

323. Groen-van de Ven L, Smits C, Span M, Jukema J, Coppoolse K, de Lange J, et al. The challenges of shared decision making in dementia care networks. International Psychogeriatrics. 2018;30(6):843-57.

324. Gu J, Wang H, Pei J, Meng J, Song Y. The dyadic coping experience of ICU transfer patients and their spouses: A qualitative study. Nursing in critical care. 2023.

325. Habermann B, Shin JY. Preferences and concerns for care needs in advanced Parkinson's disease: a qualitative study of couples. Journal of Clinical Nursing (John Wiley & Sons, Inc). 2017;26(11-12):1650-6.

326. Hall AJ, Burrows L, Lang IA, Endacott R, Goodwin VA. Are physiotherapists employing person-centred care for people with dementia? An exploratory qualitative study examining the experiences of people with dementia and their carers. BMC Geriatrics. 2018;18(1):1-.

327. Hampanda K, Matenga TFL, Nkwemu S, Shankalala P, Chi BH, Darbes LA, et al. Designing a couple-based relationship strengthening and health enhancing intervention for pregnant women living with HIV and their male partners in Zambia: Interview findings from the target community. Social Science and Medicine. 2021;283:114029.

328. Harding E, Sullivan MP, Woodbridge R, Yong KXX, McIntyre A, Gilhooly ML, et al. 'Because my brain isn't as active as it should be, my eyes don't always see': A qualitative exploration of the stress process for those living with posterior cortical atrophy. BMJ Open. 2018;8(2):e018663.

329. Harrison M, Ryan T, Gardiner C, Jones A. Psychological and emotional needs, assessment, and support post-stroke: a multi-perspective qualitative study. Topics in Stroke Rehabilitation. 2017;24(2):119-25.

330. Hartman M-E, Irvine J, Currie KL, Ritvo P, Trachtenberg L, Louis A, et al. Exploring gay couples' experience with sexual dysfunction after radical prostatectomy: a qualitative study. Journal of Sex & Marital Therapy. 2014;40(3):233-53.

331. He X, Zhang M, Wu J, Xu S, Jiang X, Wang Z, et al. Differences in lung cancer treatment preferences among oncologists, patients and family members: A semi-structured qualitative study in China. Patient Preference and Adherence. 2021;15:775-83.

332. Heaton J, Martyr A, Nelis SM, Markova IS, Morris RG, Roth I, et al. Future outlook of people living alone with early-stage dementia and their non-resident relatives and friends who support them. Ageing & Society. 2021;41(11):2660-80.

333. Henderson L, Bain H, Allan E, Kennedy C. An Exploratory Multi-Case Study of the Health and Wellbeing Needs, Relationships and Experiences of Health and Social Care Service Users and the People who Support them at Home. International journal of integrated care. 2023;23(1):11.

334. Hendriksen E, Rivera A, Williams E, Lee E, Sporn N, Cases MG, et al. Manifestations of anxiety and coping strategies in patients with metastatic lung cancer and their family caregivers: a qualitative study. Psychology & Health. 2019;34(7):886-99.

335. Hendriksen E, Williams E, Sporn N, Greer J, DeGrange A, Koopman C. Worried together: a qualitative study of shared anxiety in patients with metastatic non-small cell lung cancer and their family caregivers. Supportive Care in Cancer. 2015;23(4):1035-41.

336. Hennelly N, O'Shea E. A multiple perspective view of personhood in dementia. Ageing & Society. 2022;42(9):2103-21.

337. Henry D, Rosenthal L. "Listening for his breath:" The significance of gender and partner reporting on the diagnosis, management, and treatment of obstructive sleep apnea. Social Science & Medicine. 2013;79(1):48.

338. Henshaw EJ, Durkin KM, Snell RJ. First-time parents’ shared representation of postpartum depressive symptoms: A qualitative analysis. Social Science & Medicine. 2016;160:102-10.

339. Herbst FA, Gawinski L, Schneider N, Stiel S. 'Mums are sacred, and mums don't die': A mixed-methods study of adult child–parent dyadic relationships at the end of life. Journal of Psychosocial Oncology. 2022;40(2):152-68.

340. Heyes SM, Prior KN, Whitehead D, Bond MJ. Toward an understanding of patients' and their partners' experiences of bladder cancer. Cancer Nursing. 2020;43(5):E254-E63.

341. Hiatt J, Young A, Brown T, Banks M, Bauer J. Exploring dyadic management of nutrition care throughout and beyond head and neck cancer treatment. Journal of Clinical Nursing (John Wiley & Sons, Inc). 2022;31(19/20):2774-83.

342. Hodson T, Gustafsson L, Cornwell P. "Just got to live life as it comes": A case study of the spousal-dyad longitudinal mild stroke transitional experience. Brain Injury. 2019;33(9):1200-7.

343. Hoppe R, Winter MA, Graap K, Albrecht TA. Impact of a Hematologic Malignancy Diagnosis and Treatment on Patients and Their Family Caregivers. Oncology Nursing Forum. 2022;49(5):445-53.

344. Howard AF, Crowe S, Choroszewski L, Kovatch J, Haynes AJ, Ford J, et al. Health-related expectations of the chronically critically ill: a multi-perspective qualitative study. BMC Palliative Care. 2021;20(1):1-12.

345. Howard AF, Crowe S, Choroszewski L, Kovatch J, Kelly M, Haljan G. When chronic critical illness is a family affair: A multi-perspective qualitative study of family involvement in long-term care. Chronic Illness. 2023;19(4):804-16.

346. Howard AF, Lynch K, Beck S, Torrejón M-J, Avery J, Thorne S, et al. At the Heart of It All: Emotions of Consequence for the Conceptualization of Caregiver-Reported Outcomes in the Context of Colorectal Cancer. Current Oncology. 2021;28(5):4184-202.

347. Hsu M-C, Huang C-Y, Tu C-H. Violence and mood disorder: Views and experiences of adult patients with mood disorders using violence toward their parents. Perspectives in Psychiatric Care. 2014;50(2):111-21.

348. Hudson N, Law C, Culley L, Mitchell H, Denny E, Norton W, et al. Men, chronic illness and healthwork: accounts from male partners of women with endometriosis. Sociology of Health & Illness. 2020;42(7):1532-47.

349. Hugar LA, Geiss C, Chavez MN, Gore LR, Thirlwell S, Reblin M, et al. Exploring knowledge, perspectives, and misperceptions of palliative care: A mixed methods analysis. Urologic Oncology: Seminars and Original Investigations. 2023;41(7):327.e19-.e26.

350. Hyatt K, Davis LL, Barroso J. Chasing the care: soldiers experience following combat-related mild traumatic brain injury. Military Medicine. 2014;179(8):849-55.

351. Jensen AN, Bonnen KB, Kristiansen M. "We don't talk about his heart": Narrative sense-making and long-term readjustment among older out-of-hospital cardiac arrest survivors and their spouses. Resuscitation plus. 2020;3:100024.

352. Jiayun X, Adeboyejo O, Wagley E, Aubrecht J, Mi-Kyung S, Thiry L, et al. Daily burdens of recipients and family caregivers after lung transplant. Progress in Transplantation. 2012;22(1):41-8.

353. Johansson I-L, Samuelsson C, Müller N. Patients' and communication partners' experiences of communicative changes in Parkinson's disease. Disability & Rehabilitation. 2020;42(13):1835-43.

354. Johnston B, Bowman F, Carduff E, Donmez F, Lowndes A, McKeown A. 'Playlist for Life' at the end of life: a mixed-methods feasibility study of a personalised music listening intervention in the hospice setting. Pilot and Feasibility Studies. 2022;8(1):32.

355. Joshi R, Joseph A, Mihandoust S, Madathil KC, Cotten SR. A mobile application-based home assessment tool for patients undergoing joint replacement surgery: A qualitative feasibility study. Applied Ergonomics. 2022;103:N.PAG-N.PAG.

356. Kadi S, Rodrigues R, Kahlert R, Hofmann S, Bauer G. Does the family care best? Ideals of care in a familialistic care regime. Journal of Social Policy. 2024;53(1):45-62.

357. Keesing S, Rosenwax L, McNamara B. A dyadic approach to understanding the impact of breast cancer on relationships between partners during early survivorship. BMC Women's Health. 2016;16:1-14.

358. Keller HH, Martin LS, Dupuis S, Reimer H, Genoe R. Strategies to support engagement and continuity of activity during mealtimes for families living with dementia; a qualitative study. BMC Geriatrics. 2015;15(1):1-14.

359. Kelly-Campbell RJ, Wendel K. Measuring cognitive anxiety through the consultation process for hearing aids: Older adults and their significant others. Journal of Communication Disorders. 2015;56:29-39.

360. Kendall M, Carduff E, Lloyd A, Kimbell B, Cavers D, Buckingham S, et al. Different Experiences and Goals in Different Advanced Diseases: Comparing Serial Interviews With Patients With Cancer, Organ Failure, or Frailty and Their Family and Professional Carers. Journal of Pain & Symptom Management. 2015;50(2):216-24.

361. Killin LOJ, Russ TC, Surdhar SK, Yoon Y, McKinstry B, Gibson G, et al. Digital Support Platform: A qualitative research study investigating the feasibility of an internet-based, postdiagnostic support platform for families living with dementia. BMJ Open. 2018;8(4):e020281.

362. Kim JSR, Risbud R, Gray C, Banerjee D, Trivedi R. The Dyadic Experience of Managing Heart Failure: A Qualitative Investigation. Journal of Cardiovascular Nursing. 2020;35(1):12-8.

363. Kimbell B, Boyd K, Kendall M, Iredale J, Murray SA. Managing uncertainty in advanced liver disease: a qualitative, multiperspective, serial interview study. BMJ Open. 2015;5(11):e009241.

364. Kimmel HJ, Levine DA, Whitney RT, Forman J, Plassman BL, Fagerlin A, et al. A Mixed-Methods Study of the Impact of Mild Cognitive Impairment Diagnosis on Patient and Care Partner Perception of Health Risks. Journal of Alzheimer's Disease. 2022;85(3):1175-87.

365. Kinsey D, Orr N, Anderson R, Lang I. A conceptual model of the impact of including carers in museum programmes for people with dementia. Dementia (14713012). 2022;21(8):2584-600.

366. Kishino M, Koffman J, Nagatomi H, Yuasa M, Ellis-Smith C. Complexity and function of family involvement in advance care planning: A qualitative study of perspectives from people living with advanced cancer, family members and healthcare professionals. Palliative Medicine. 2023;37(9):1434-46.

367. Kitzmüller G, Häggström T, Asplund K, Gilje FL. THE EXISTENTIAL MEANING OF COUPLES' LONG-TERM EXPERIENCE OF LIVING WITH STROKE. Illness, Crisis & Loss. 2012;20(4):339-62.

368. Kjallman Alm A, Hellzen O, Norbergh KG. Experiences of long term ongoing structured support in early stage of dementia - a case study. International journal of older people nursing. 2014;9(4):289-97.

369. Klotz LK. Hope in relation to nursing interventions for HIV-infected patients and their significant others. Journal of the Association of Nurses in AIDS Care. 2010;21(4):345-55.

370. Knox L, Douglas JM, Bigby C. "I won't be around forever": Understanding the decision-making experiences of adults with severe TBI and their parents. Neuropsychological Rehabilitation. 2016;26(2):236-60.

371. Koehn SD, Donahue M, Feldman F, Drummond N. Fostering trust and sharing responsibility to increase access to dementia care for immigrant older adults. Ethnicity & Health. 2022;27(1):83-99.

372. Kristiansen M, Irshad T, Worth A, Bhopal R, Lawton J, Sheikh A. The practice of hope: a longitudinal, multi-perspective qualitative study among South Asian Sikhs and Muslims with life-limiting illness in Scotland. Ethnicity & Health. 2014;19(1):1-19.

373. Kung PC, Hsu WC, Huang HL, Chang HJ, Shyu YIL. Coexisting with anomie: Experiences of persons living with early-stage dementia: A grounded theory study. International journal of mental health nursing. 2023.

374. Lam ITY, Keller HH. Honoring Identity Through Mealtimes in Chinese Canadian Immigrants. American Journal of Alzheimer's Disease & Other Dementias. 2015;30(7):662-71.

375. LeBaron V, Bennett R, Alam R, Blackhall L, Gordon K, Hayes J, et al. Understanding the Experience of Cancer Pain From the Perspective of Patients and Family Caregivers to Inform Design of an In-Home Smart Health System: Multimethod Approach. JMIR formative research. 2020;4(8):e20836.

376. Lee H-TS, Chen T-R, Yang C-L, Chiu T-Y, Hu W-Y. Action research study on advance care planning for residents and their families in the long-term care facility. BMC palliative care. 2019;18(1):95.

377. Lee JI, Cutugno C, Pickering SP, Press MJ, Richardson JE, Unterbrink M, et al. The patient care circle: a descriptive framework for understanding care transitions. Journal of Hospital Medicine. 2013;8(11):619-26.

378. Leggett AN, Robinson-Lane SG, Oxford G, Leonard N, Carmichael AG, Baker E, et al. Barriers to and Facilitators of Family Caregiving of Patients With COVID-19 Early in the Pandemic. American Journal of Critical Care. 2023;32(6):431-9.

379. Leung P, Yates L, Orgeta V, Hamidi F, Orrell M. The experiences of people with dementia and their carers participating in individual cognitive stimulation therapy. International Journal of Geriatric Psychiatry. 2017;32(12):e34-e42.

380. Liao Z, Wang C, Lan X, Wu J, Yuan X, Wu Y, et al. Never forsake-The positive experiences of dyadic coping among patients with acute leukemia and their spouses: A qualitative study. European journal of oncology nursing : the official journal of European Oncology Nursing Society. 2023;62:102262.

381. Liao ZY, Haycock-Stuart E, Kean S. Biographical continuation: recovery of stroke survivors and their family caregivers in Taiwan. Primary health care research & development. 2024;25:e2.

382. Lin S, Wang C, Wang Q, Xie S, Tu Q, Zhang H, et al. The experience of stroke survivors and caregivers during hospital-to-home transitional care: A qualitative longitudinal study. International Journal of Nursing Studies. 2022;130:N.PAG-N.PAG.

383. Lindau ST, Surawska H, Paice J, Baron SR. Communication about sexuality and intimacy in couples affected by lung cancer and their clinical-care providers. Psycho-Oncology. 2011;20(2):179-85.

384. Lindell KO, Olshansky E, Song M, Zullo TG, Gibson KF, Kaminski N, et al. Impact of a disease-management program on symptom burden and health-related quality of life in patients with idiopathic pulmonary fibrosis and their care partners. Heart & Lung. 2010;39(4):304-13.

385. Linville D, Cobb E, Shen F, Stadelman S. Reciprocal Influence of Couple Dynamics and Eating Disorders. Journal of Marital & Family Therapy. 2016;42(2):326-40.

386. Lound A, Bruton J, Jones K, Shah N, Williams B, Gross J, et al. "I'd rather wait and see what's around the corner": A multi-perspective qualitative study of treatment escalation planning in frailty. PLoS ONE. 2023;18(9 September):e0291984.

387. Low C, Tejero I, Toledano N, Mariano C, Alibhai S, Lemonde M, et al. Impact of COVID-19 on older adults with cancer and their caregivers' cancer treatment experiences study: The ICE-OLD study. PLoS ONE. 2023;18(9 September):e0291756.

388. Marques MJ, Goncalves-Pereira M, de Vugt M, Verhey F, Woods B. The quality of family relationships in dementia: Mixed methods to unravel mixed feelings. Dementia (London, England). 2024;23(2):210-33.

389. Martin SC. Relational Issues Within Couples Coping With Parkinson's Disease: Implications and Ideas for Family-Focused Care. Journal of family nursing. 2016;22(2):224-51.

390. Mason B, Nanton V, Epiphaniou E, Murray SA, Donaldson A, Shipman C, et al. 'My body's falling apart.' Understanding the experiences of patients with advanced multimorbidity to improve care: serial interviews with patients and carers. BMJ supportive & palliative care. 2016;6(1):60-5.

391. Mazanderani F, Hughes N, Hardy C, Sillence E, Powell J. Health information work and the enactment of care in couples and families affected by Multiple Sclerosis. Sociology of Health & Illness. 2019;41(2):395-410.

392. McCarthy MJ, Bauer E. In Sickness and in Health: Couples Coping with Stroke across the Life Span. Health & Social Work. 2015;40(3):e92-e100.

393. McCauley CO, Bond RB, Ryan A, Mulvenna MD, Laird L, Gibson A, et al. Evaluating User Engagement with a Reminiscence App Using Cross-Comparative Analysis of User Event Logs and Qualitative Data. CyberPsychology, Behavior & Social Networking. 2019;22(8):543-51.

394. McCauley R, Ryan K, McQuillan R, Foley G. Patient and Caregiver Reciprocal Support: Impact on Decision Making in Specialist Palliative Care. Journal of Pain and Symptom Management. 2023;66(5):570-7.

395. McCluskey S, Brooks J, King N, Burton K. The influence of 'significant others' on persistent back pain and work participation: a qualitative exploration of illness perceptions. BMC Musculoskeletal Disorders. 2011;12(1):236-.

396. McCluskey S, de Vries H, Reneman M, Brooks J, Brouwer S. 'I think positivity breeds positivity': a qualitative exploration of the role of family members in supporting those with chronic musculoskeletal pain to stay at work. BMC Family Practice. 2015;16(1):1-7.

397. McDougall A, Goldszmidt M, Kinsella EA, Smith S, Lingard L. Collaboration and entanglement: An actor-network theory analysis of team-based intraprofessional care for patients with advanced heart failure. Social Science & Medicine. 2016;164:108-17.

398. McIntyre A, Reynolds F. There's no apprenticeship for Alzheimer's: the caring relationship when an older person experiencing dementia falls. Ageing & Society. 2012;32(5):873-96.

399. McPhillips MV, Petrovsky DV, Brewster GS, Li J, Gooneratne NS, Hodgson NA, et al. Recruiting persons with dementia and caregivers in a clinical trial: Dyads perceptions. Western Journal of Nursing Research. 2022;44(6):557-66.

400. McWilliams L, Farrell C, Keady J, Swarbrick C, Burgess L, Grande G, et al. Cancer-related information needs and treatment decision-making experiences of people with dementia in England: A multiple perspective qualitative study. BMJ Open. 2018;8(4):e020250.

401. Melis P, Galletta M, Aviles Gonzalez CI, Contu P, Jimenez Herrera MF. Experiencing communication related to knowing the cancer diagnosis and prognosis: A multi-perspective interpretative phenomenological study. European journal of oncology nursing : the official journal of European Oncology Nursing Society. 2021;51:101904.

402. Mendes Pereira C, Greenwood N, Jones F. "A proof of life" through transition from hospital to home after a stroke in a Portuguese setting - a multi-perspective, longitudinal qualitative study. International Journal of Qualitative Studies on Health & Well-Being. 2023;18(1):1-16.

403. Mendez-Luck CA, Amorim C, Anthony KP. Talk versus Action: Comparing the Narratives and Behaviors of Diabetes Self-Management in Latino Caregiving Dyads. The Diabetes educator. 2016;42(4):444-51.

404. Mengoni SE, Gates B, Parkes G, Wellsted D, Barton G, Ring H, et al. "Sometimes, it just stops me from doing anything": A qualitative exploration of epilepsy management in people with intellectual disabilities and their carers. Epilepsy & behavior : E&B. 2016;64(Pt A):133-9.

405. Meranius MS, Josefsson K. Health and social care management for older adults with multimorbidity: a multiperspective approach. Scandinavian journal of caring sciences. 2017;31(1):96-103.

406. Mesquita da Silva S, Place JM, Boivin J, Gameiro S. Failure after fertility treatment: regulation strategies when facing a blocked parenthood goal. Human Fertility. 2020;23(3):179-85.

407. Metzger M, Song MK, Ward S, Chang PPY, Hanson LC, Lin FC. A randomized controlled pilot trial to improve advance care planning for LVAD patients and their surrogates. Heart and Lung: Journal of Acute and Critical Care. 2016;45(3):186-92.

408. Milliron BJ, Klobodu C, Deutsch J, Martyn K, Dychtwald D, Riahi E, et al. "Keep Your Chin Up, and Keep Eating": Perceptions of Barriers and Facilitators to Healthful Dietary Behaviors Among Individuals With Gastrointestinal Cancer and Caregivers. Cancer Control. 2023;30.

409. Mindry D, Wanyenze RK, Beyeza-Kashesya J, Woldetsadik MA, Finocchario-Kessler S, Goggin K, et al. Safer conception for couples affected by HIV: Structural and cultural considerations in the delivery of safer conception care in Uganda. AIDS and Behavior. 2017;21(8):2488-96.

410. Mishu MP, Faisal MR, Macnamara A, Sabbah W, Peckham E, Newbronner L, et al. Exploring the contextual factors, behaviour change techniques, barriers and facilitators of interventions to improve oral health in people with severe mental illness: A qualitative study. Frontiers in Psychiatry. 2022;13:971328.

411. Mishu MP, Faisal MR, Macnamara A, Sabbah W, Peckham E, Newbronner L, et al. A Qualitative Study Exploring the Barriers and Facilitators for Maintaining Oral Health and Using Dental Service in People with Severe Mental Illness: Perspectives from Service Users and Service Providers. International Journal of Environmental Research and Public Health. 2022;19(7):4344.

412. Mitchell E, Kelly-Hanku A, Mek A, Trumb RN, Persson A, Worth H, et al. Caring masculinities in the context of HIV serodiscordant relationships in Papua New Guinea. Men and Masculinities. 2021;24(2):326-44.

413. Modi A, Kosambiya J, Trivedi S, Chaudhari V, Mehta A, Wells K. "My life is spoiled because of him..." A qualitative study of human immunodeficiency virus disclosure and male involvement in prevention of mother-to-child transmission program. Indian Journal of Community Medicine. 2019;44(4):322-7.

414. Mokoena AG, Poggenpoel M, Myburgh C, Temane A. Lived experiences of couples in a relationship where one partner is diagnosed with a mental illness. Curationis. 2019;42(1):1-7.

415. Molzahn AE, Schick‐Makaroff K, Bruce A, Sheilds L, White L, Antonio M. Living with dying: A narrative inquiry of people with chronic kidney disease and their family members. Journal of Advanced Nursing (John Wiley & Sons, Inc). 2019;75(1):129-37.

416. Moore C, Wearden A, Carter LA, Mitra S, Skevington SM. Development of a measure for patients preparing to start dialysis and their partners: The Starting Dialysis Questionnaire (SDQ). Health and Quality of Life Outcomes. 2020;18(1):358.

417. Morrisby C, Joosten A, Ciccarelli M. Needs of people with dementia and their spousal carers: A study of those living in the community. Australasian Journal on Ageing. 2019;38(2):e43-e9.

418. Mou H, Lam SKK, Chien WT. Effects of a family-focused dyadic psychoeducational intervention for stroke survivors and their family caregivers: a pilot study. BMC Nursing. 2022;21(1):1-17.

419. Mountain GA, Craig CL. What should be in a self-management programme for people with early dementia? Aging & Mental Health. 2012;16(5):576-83.

420. Moyle W, Jones C, Dwan T, Ownsworth T, Sung B. Using telepresence for social connection: views of older people with dementia, families, and health professionals from a mixed methods pilot study. Aging & Mental Health. 2019;23(12):1643-50.

421. Mushtaq N, Ali R. MARITAL SATISFACTION OF BREAST CANCER PATIENTS AND THEIR SPOUSES: A QUALITATIVE STUDY. Pakistan Journal of Women's Studies = Alam-e-Niswan = Alam-i Nisvan. 2019;26(2):65-87.

422. Muwanguzi PA, Nassuna LK, Voss JG, Kigozi J, Muganzi A, Ngabirano TD, et al. Towards a definition of male partner involvement in the prevention of mother-to-child transmission of HIV in Uganda: a pragmatic grounded theory approach. BMC Health Services Research. 2019;19(1):N.PAG-N.PAG.

423. Nakku-Joloba E, Pisarski EE, Wyatt MA, Muwonge TR, Asiimwe S, Celum CL, et al. Beyond HIV prevention: everyday life priorities and demand for PrEP among Ugandan HIV serodiscordant couples. Journal of the International AIDS Society. 2019;22(1):e25225.

424. Neal DP, Kuiper L, Pistone D, Osinga C, Nijland S, Ettema T, et al. FindMyApps eHealth intervention improves quality, not quantity, of home tablet use by people with dementia. Frontiers in Medicine. 2023;10:1152077.

425. Newman C, Persson A, Ellard J. When HIV figures in family life: Exploring the relational reach of serodiscordance. Sexuality & Culture: An Interdisciplinary Quarterly. 2018;22(4):1527-41.

426. Ngure K, Baeten JM, Mugo N, Curran K, Vusha S, Heffron R, et al. My intention was a child but I was very afraid: Fertility intentions and HIV risk perceptions among HIV-serodiscordant couples experiencing pregnancy in Kenya. AIDS Care. 2014;26(10):1283-7.

427. Ngure K, Mugo N, Celum C, Baeten JM, Morris M, Olungah O, et al. A qualitative study of barriers to consistent condom use among HIV-1 serodiscordant couples in Kenya. AIDS Care. 2012;24(4):509-16.

428. Ngure K, Vusha S, Mugo N, Emmanuel-Fabula M, Ngutu M, Celum C, et al. “I never thought that it would happen … ” Experiences of HIV seroconverters among HIV-discordant partnerships in a prospective HIV prevention study in Kenya. AIDS Care. 2016;28(12):1586-9.

429. Ngwenya N, Farquhar M, Ewing G. Sharing bad news of a lung cancer diagnosis: understanding through communication privacy management theory. Psycho-Oncology. 2016;25(8):913-8.

430. Nimmons D, Armstrong M, Pigott J, Walters K, Schrag A, Ogunleye D, et al. Exploring the experiences of people and family carers from under-represented groups in self-managing Parkinson's disease and their use of digital health to do this. Digital health. 2022;8:20552076221102261.

431. Nissen NK, Jónsdóttir M, Spindler H, Zwisler A-DO. Resistance to change: Role of relationship and communal coping for coronary heart disease patients and their partners in making lifestyle changes. Scandinavian Journal of Public Health. 2018;46(6):659-66.

432. Nutting R, Grafsky EL. Crohn’s Disease and the Young Couple: An Interpretative Phenomenological Analysis. Contemporary Family Therapy: An International Journal. 2018;40(2):176-87.

433. O'Brien AJ, Chesla CA, Humphreys JC. Couples' Experiences of Maternal Postpartum Depression. JOGNN: Journal of Obstetric, Gynecologic & Neonatal Nursing. 2019;48(3):341-50.

434. O'Connell B, Ghosh M, Dunham M, Smyth A. Evaluation of an educational program for people with dementia and their caregivers. Australasian Journal on Ageing. 2023;42(3):517-26.

435. Olander A, Andersson H, Sundler AJ, Hagiwara MA, Bremer A. The onset of sepsis as experienced by patients and family members: A qualitative interview study. Journal of Clinical Nursing (John Wiley & Sons, Inc). 2023;32(19/20):7402-11.

436. Oldfield M, MacEachen E, Kirsh B, MacNeill M. Impromptu everyday disclosure dances: how women with fibromyalgia respond to disclosure risks at work. Disability & Rehabilitation. 2016;38(15):1442-53.

437. Oldfield M, MacEachen E, MacNeill M, Kirsh B. ‘You want to show you’re a valuable employee’: A critical discourse analysis of multi-perspective portrayals of employed women with fibromyalgia. Chronic Illness. 2018;14(2):135-53.

438. Osborne JB, Rocchi MA, McBride CB, McKay R, Gainforth HL, Upper R, et al. Couples' experiences with sexuality after spinal cord injury. Disability & Rehabilitation. 2023;45(4):664-72.

439. Owen CL, Gaulton C, Roberts HC, Dennison L. Perceptions of people with Parkinson's and their caregivers of falling and falls-related healthcare services- a qualitative study. PLoS ONE. 2022;17(10 October):e0276588.

440. Patel RC, Stanford-Moore G, Odoyo J, Pyra M, Wakhungu I, Anand K, et al. "Since both of us are using antiretrovirals, we have been supportive to each other": facilitators and barriers of pre-exposure prophylaxis use in heterosexual HIV serodiscordant couples in Kisumu, Kenya. Journal of the International AIDS Society. 2016;19(1):21134.

441. Pearson CR, Forsyth F, Khair E, Sowden E, Borja Boluda S, Deaton C, et al. 'Keeping the plates spinning': a qualitative study of the complexity, barriers, and facilitators to caregiving in heart failure with preserved ejection fraction. European Journal of Cardiovascular Nursing. 2023;22(2):141-8.

442. Pentecost C, Collins R, Stapley S, Victor C, Quinn C, Hillman A, et al. Effects of social restrictions on people with dementia and carers during the pre‐vaccine phase of the COVID‐19 pandemic: Experiences of IDEAL cohort participants. Health & Social Care in the Community. 2022;30(6):e4594-e604.

443. Pereira CM, Greenwood N, Jones F. From recovery to regaining control of life – the perspectives of people with stroke, their carers and health professionals. Disability & Rehabilitation. 2021;43(20):2897-908.

444. Persson A, Newman CE, valentine k, Hamilton M, Bryant J, Wallace J. The embodied relationality of blood-borne viruses: How families matter in the context of a stigmatised viral infection. Social Science & Medicine. 2019;243.

445. Petrillo LA, Shimer SE, Zhou AZ, Sommer RK, Feldman JE, Hsu KE, et al. Prognostic communication about lung cancer in the precision oncology era: A multiple-perspective qualitative study. Cancer. 2022;128(16):3120-8.

446. Petry H, Ernst J, Steinbrüchel-Boesch C, Altherr J, Naef R. The acute care experience of older persons with cognitive impairment and their families: A qualitative study. International Journal of Nursing Studies. 2019;96:44-52.

447. Pintye J, Ngure K, Curran K, Vusha S, Mugo N, Celum C, et al. Fertility decision-making among Kenyan HIV-serodiscordant couples who recently conceived: Implications for safer conception planning. AIDS Patient Care and STDs. 2015;29(9):510-6.

448. Popok PJ, Reichman M, LeFeber L, Grunberg VA, Bannon SM, Vranceanu A-M. One diagnosis, two perspectives: Lived experiences of persons with young-onset dementia and their care-partners. The Gerontologist. 2022;62(9):1311-23.

449. Pratt MC, Moran O, Muyindike W, Kaida A, Marrazzo JM, Bangsberg DR, et al. ‘I still desire to have a child’: a qualitative analysis of intersectional HIV- and childlessness-related stigma in rural southwestern Uganda. Culture, Health & Sexuality. 2023;25(2):143-58.

450. Prick A-E, de Lange J, van 't Leven N, Pot AM. Process evaluation of a multicomponent dyadic intervention study with exercise and support for people with dementia and their family caregivers. Trials. 2014;15(1):401-.

451. Puts M, Sattar S, McWatters K, Lee K, Kulik M, MacDonald M-E, et al. Chemotherapy treatment decision-making experiences of older adults with cancer, their family members, oncologists and family physicians: a mixed methods study. Supportive Care in Cancer. 2017;25(3):879-86.

452. Quinn C, Clare L, McGuinness T, Woods RT. Negotiating the balance: The triadic relationship between spousal caregivers, people with dementia and Admiral Nurses. Dementia. 2013;12(5):588-605.

453. Radley J, Barlow J, Johns LC. A family perspective on parental psychosis: An interpretative phenomenological analysis study. Psychology & Psychotherapy: Theory, Research & Practice. 2023;96(2):347-63.

454. Ramazanu S, Chiang VCL, Valimaki M. The experiences and evaluation of a complex intervention for couples coping with stroke. Journal of Neuroscience Nursing. 2021;53(1):18-23.

455. Ramazanu S, Leung D, Chiang VCL. The Experiences of Couples Affected by Stroke and Nurses Managing Patient Rehabilitation: A Descriptive Study in Singapore. The journal of nursing research : JNR. 2020;28(5):e113.

456. Reaney M, Chmiel N, Churchill S. Foot care, ‘spousal’ support and type 2 diabetes: an exploratory qualitative study. Psychology & Health. 2018;33(9):1191-207.

457. Reblin M, McCormick R, Mansfield KJ, Wawrzynski SE, Ketcher D, Tennant KE, et al. Feasibility, usability, and acceptability of personalized web-based assessment of social network and daily social support interactions over time. Journal of cancer survivorship : research and practice. 2022;16(4):904-12.

458. Richardson A, Wagland R, Foster R, Symons J, Davis C, Boyland L, et al. Uncertainty and anxiety in the cancer of unknown primary patient journey: a multiperspective qualitative study. BMJ supportive & palliative care. 2015;5(4):366-72.

459. Risbud RD, Kim JS, Trivedi RB. It Takes a Village: Interpersonal Factors That Enhance Management of Heart Failure. Journal of Cardiovascular Nursing. 2022;37(5):E160-E8.

460. Rispel LC, Cloete A, Metcalf CA. 'We keep her status to ourselves': Experiences of stigma and discrimination among HIV-discordant couples in South Africa, Tanzania and Ukraine. SAHARA-J: Journal of Social Aspects of HIV / AIDS. 2015;12(1):10-7.

461. Rispel LC, Metcalf CA, Moody K, Cloete A, Caswell G. Sexual relations and childbearing decisions of HIV-discordant couples: an exploratory study in South Africa and Tanzania. Reproductive Health Matters. 2011;19(37):184-93.

462. Rivers BM, August EM, Gwede CK, Hart A, Jr., Donovan KA, Pow-Sang JM, et al. Psychosocial issues related to sexual functioning among African-American prostate cancer survivors and their spouses. Psycho-Oncology. 2011;20(1):106-10.

463. Rivers BM, August EM, Quinn GP, Gwede CK, Pow-Sang JM, Green BL, et al. Understanding the psychosocial issues of African American couples surviving prostate cancer. Journal of Cancer Education. 2012;27(3):546-58.

464. Robson M, Riley S, Gagen E, McKeogh D. Love and lifestyle: How 'relational healthism' structures couples' talk of engagement with lifestyle advice associated with a new diagnosis of coronary heart disease. Psychology & Health. 2023;38(12):1606-22.

465. Rosenberg NE, Gross R, Mtande T, Maman S, Golin CE, Saidi F, et al. "We have heard it together": A qualitative analysis of couple HIV testing and counselling recruitment in Malawi's Option B+ programme. African Journal of AIDS Research. 2017;16(3):215-23.

466. Saberi P, Gamarel KE, Neilands TB, Comfort M, Sheon N, Darbes LA, et al. Ambiguity, ambivalence, and apprehensions of taking HIV-1 pre-exposure prophylaxis among male couples in San Francisco: A mixed methods study. PLoS ONE. 2012;7(11).

467. Samsi K, Manthorpe J. Everyday decision-making in dementia: findings from a longitudinal interview study of people with dementia and family carers. International Psychogeriatrics. 2013;25(6):949-61.

468. Sandberg LJ. Too late for love? Sexuality and intimacy in heterosexual couples living with an Alzheimer's disease diagnosis. Sexual & Relationship Therapy. 2023;38(1):118-39.

469. Savisit W, Homchampa P, Sirithanawutichai T. Family-Based Palliative Care Model for Advanced Cancer Patients in Community Settings in the Northeast, Thailand. Journal of the Medical Association of Thailand. 2022;105(9):883-95.

470. Seah CEL, Zhang Z, Sun S, Wiskerke E, Daniels S, Porat T, et al. Designing Mindfulness Conversational Agents for People With Early-Stage Dementia and Their Caregivers: Thematic Analysis of Expert and User Perspectives. JMIR aging. 2022;5(4):e40360.

471. SerÇE YÜKsel Ö, GÜNÜŞEn N. Experiences of Common Unmet Psychosocial Care Needs of Hospitalized Individual with Hematological Cancers and Family Caregiver Dyads: A Qualitative Study. Turkiye Klinikleri Journal of Nursing Sciences. 2023;15(1):236-44.

472. Simin T, Jin Y, Aidi Z, Xiaofang T, Chunhong R, Lezhi L. Gender Comparison of Psychological Reaction Between Breast Cancer Survivors and Their Spouses. Frontiers in psychology. 2021;12:722877.

473. Sinclair C, Gersbach K, Hogan M, Bucks RS, Auret KA, Clayton JM, et al. How couples with dementia experience healthcare, lifestyle, and everyday decision-making. International Psychogeriatrics. 2018;30(11):1639-47.

474. Slepicková L. Couples undergoing infertility treatment in the Czech Republic: Broad range of possibilities in a traditional milieu. Social Theory & Health. 2010;8(2):151.

475. Slightam C, Risbud R, Guetterman TC, Nevedal AL, Nelson KM, Piette JD, et al. Patient, caregiving partner, and clinician recommendations for improving heart failure care in the Veterans Health Administration. Chronic Illness. 2022;18(2):330-42.

476. Smith KA, Van Pinxteren M, Mbokazi N, Majikela-Dlangamandla B, Delobelle P, Levitt N, et al. Intervention development of 'Diabetes Together' using the person-based approach: A couples-focused intervention to support self-management of type 2 diabetes in South Africa. BMJ Open. 2023;13(5):e069982.

477. Smith L, Shaw R, Smith LJ, Shaw RL. Learning to live with Parkinson's disease in the family unit: an interpretative phenomenological analysis of well-being. Medicine, Health Care & Philosophy. 2017;20(1):13-21.

478. Smith R, Netto J, Gribble NC, Falkmer M. 'At the end of the day, it's love': An exploration of relationships in neurodiverse couples. Journal of Autism and Developmental Disorders. 2021;51(9):3311-21.

479. Smorti M, Smorti A. Medical successes and couples' psychological problems in assisted reproduction treatment: A narrative based medicine approach. Journal of Maternal-Fetal and Neonatal Medicine. 2013;26(2):169-72.

480. Sousa H, Ribeiro O, Christensen AJ, Figueiredo D. Designing family-based interventions in kidney failure: The perspectives of the triad 'patients on haemodialysis/family caregivers/healthcare professionals. British journal of health psychology. 2023;28(3):672-89.

481. St. Cyr K, Liu JJW, Cramm H, Nazarov A, Hunt R, Forchuk C, et al. "You can't un-ring the bell": a mixed methods approach to understanding veteran and family perspectives of recovery from military-related posttraumatic stress disorder. BMC Psychiatry. 2022;22(1):37.

482. Stanton AM, Bwana M, Owembabazi M, Atukunda E, Musinguzi E, Ezegbe H, et al. Sexual and Relationship Benefits of a Safer Conception Intervention Among Men with HIV Who Seek to Have Children with Serodifferent Partners in Uganda. AIDS & Behavior. 2022;26(6):1841-52.

483. Starks H, Morris MA, Yorkston KM, Gray RF, Johnson KL. Being in- or out-of-sync: Couples' adaptation to change in multiple sclerosis. Disability and Rehabilitation: An International, Multidisciplinary Journal. 2010;32(3):196-206.

484. Stuckey HL, Desai U, King SB, Popadic L, Levinson W, Kirson NY, et al. The experience of a severe hypoglycaemic event from the perspective of people with diabetes and their caregivers: "What am I going to do?". Diabetic Medicine. 2022;39(4):1-12.

485. Sundstrom M, Jola C. "I'm Never Going to Be in Phantom of the Opera": Relational and Emotional Wellbeing of Parkinson's Carers and Their Partners in and Beyond Dancing. Frontiers in psychology. 2021;12:636135.

486. Tabana H, Doherty T, Rubenson B, Jackson D, Ekstrom AM, Thorson A. 'Testing Together Challenges the Relationship': Consequences of HIV Testing as a Couple in a High HIV Prevalence Setting in Rural South Africa. PLoS ONE. 2013;8(6):e66390.

487. Tabong PT-N, Adongo PB. Infertility and childlessness: a qualitative study of the experiences of infertile couples in Northern Ghana. BMC Pregnancy & Childbirth. 2013;13(1):72-.

488. Takashi N, Musumari PM, Techasrivichien T, Suguimoto SP, Ono-Kihara M, Kihara M, et al. Unmet needs in long-term outpatient rehabilitative care: a qualitative and multi-perspective study in Japan. Disability & Rehabilitation. 2023;45(22):3668-76.

489. Tarbi EC, Morgan B. Opportunities for Poetic Analysis in Qualitative Nursing Research. Nursing Research. 2022;71(4):322-7.

490. Taylor B. Experiences of sexuality and intimacy in terminal illness: a phenomenological study. Palliative medicine. 2014;28(5):438-47.

491. Taylor B. Does the caring role preclude sexuality and intimacy in coupled relationships? Sexuality and Disability. 2015;33(3):365-74.

492. Taylor LC. The Experience of Infertility Among African American Couples. Journal of African American Studies. 2018;22(4):357-72.

493. Torgé CJ. Whose Right to a “Reasonable Level of Living”. The Journal of Aging and Social Change. 2018;8(1):45.

494. Trivedi RB, Slightam C, Nevedal A, Guetterman TC, Fan VS, Nelson KM, et al. Comparing the barriers and facilitators of heart failure management as perceived by patients, caregivers, and clinical providers. Journal of Cardiovascular Nursing. 2019;34(5):399-409.

495. Turkmenoglu G, Karadag A. Problems Experienced by Spouses of Turkish Patients With a Stoma: A Descriptive, Cross-Sectional Study. Wound management & prevention. 2019;65(11):33-41.

496. Turner D, Nachtigall RD. The experience of infertility by low-income immigrant Latino couples: attitudes toward adoption. Adoption Quarterly. 2010;13(1):18-33.

497. Ussher JM, Perz J, May E. Pathology or source of power? The construction and experience of premenstrual syndrome within two contrasting cases. Feminism & Psychology. 2014;24(3):332-51.

498. Valente M, Chirico I, Girotti C, Ottoboni G, Chattat R. The Role of the Quality of Relationship in Couples Facing Treatment for Breast Cancer: A Qualitative Italian Study. American journal of clinical oncology. 2023;46(1):36-42.

499. van der Roest HG, Meiland FJM, Jonker C, Droes R-M. User evaluation of the DEMentia-specific Digital Interactive Socal Chart (DEM-DISC). A Pilot study among informal carers on its impact, user friendliness and, usefulness. Aging & Mental Health. 2010;14(4):461-70.

500. van Eenennaam RM, Rave N, Kruithof WJ, Kruitwagen-Van Reenen ET, van den Berg LH, Visser-Meily JA, et al. Control in the absence of choice: A qualitative study on decision-making about gastrostomy in people with amyotrophic lateral sclerosis, caregivers, and healthcare professionals. PLoS ONE. 2023;18(9 September):e0290508.

501. van Roij J, de Zeeuw B, Zijlstra M, Claessens N, Raijmakers N, de Poll-Franse Lv, et al. Shared Perspectives of Patients With Advanced Cancer and Their Informal Caregivers on Essential Aspects of Health Care: A Qualitative Study. Journal of Palliative Care. 2022;37(3):372-80.

502. Van TLN, de Lange J, van der Ploeg ES, Pot AM. Working mechanisms of dyadic, psychosocial, activating interventions for people with dementia and informal caregivers: A qualitative study. Clinical Interventions in Aging. 2018;13:1847-57.

503. Veenstra CM, Acosta J, Sharar R, Hawley ST, Morris AM. Partners' engagement in surveillance among survivors of colorectal cancer: A qualitative study. Cancer Medicine. 2021;10(4):1289-96.

504. Venetis MK, Bontempo AC, Catona D, Buckley de Meritens A, Devine KA, Greene K. Dilemmas and strategy when companion participation during appointments differs from patient and companion expectations. Health Communication. 2023:No-Specified.

505. Verma SJ, Gulati P, Injety RJ, Arora D, Dhasan A, Singhania A, et al. Secondary prevention by structured semi-interactive stroke prevention package in INDIA (SPRINT INDIA): Findings from the process evaluation of a randomized controlled trial. European Stroke Journal. 2023;8(4):1053-63.

506. Walker ER, Barmon C, McGee RE, Engelhard G, Sterk CE, DiIorio C, et al. A dyadic model of living with epilepsy based on the perspectives of adults with epilepsy and their support persons. Epilepsy and Behavior. 2015;53:1-9.

507. Wallhagen MI. The stigma of hearing loss. The Gerontologist. 2010;50(1):66-75.

508. Wang W, Pucciarelli G, Mei Y, Zhang Z, Vellone E. The dyadic self‐care experience of stroke survivors and their caregivers: A qualitative descriptive study. Health Expectations. 2023;26(6):2325-39.

509. Ware NC, Wyatt MA, Pisarski EE, Nalumansi A, Kasiita V, Kamusiime B, et al. How Central Ugandan HIV Clinics Adapted During COVID-19 Lockdown Restrictions to Promote Continuous Access to Care: A Qualitative Analysis. AIDS and Behavior. 2023;27(11):3725-34.

510. Warmoth K, Morgan-Trimmer S, Kudlicka A, Toms G, James IA, Woods B. Reflections on a personalized cognitive rehabilitation intervention: Experiences of people living with dementia and their carers participating in the GREAT trial. Neuropsychological rehabilitation. 2022;32(2):268-86.

511. Wass N, Chadwick R, Caygill L, O'Mara O. "It kind of strips down your relationship to its defining features......it just kind of shone a light on what was already there": A grounded theory of the impact of postpartum psychosis on the couple's relationship. Journal of Reproductive & Infant Psychology. 2024;42(2):281-98.

512. Williamson LE, Sleeman KE, Evans CJ. Exploring access to community care and emergency department use among people with dementia: A qualitative interview study with people with dementia, and current and bereaved caregivers. International Journal of Geriatric Psychiatry. 2023;38(7):1-11.

513. Wilson B, Beamish W, Hay S, Attwood T. Prompt dependency beyond childhood: Adults with Asperger's syndrome and intimate relationships. Journal of Relationships Research. 2014;5.

514. Withers M, Dworkin S, Harrington E, Kwena Z, Onono M, Bukusi E, et al. Fertility intentions among HIV-infected, sero-concordant couples in Nyanza province, Kenya. Culture, Health & Sexuality. 2013;15(10):1175-90.

515. Wittmann D, Carolan M, Given B, Skolarus TA, An L, Palapattu G, et al. Exploring the role of the partner in couples' sexual recovery after surgery for prostate cancer. Supportive Care in Cancer. 2014;22(9):2509-15.

516. Wolverson E, White C, Dunn R, Cunnah K, Howe D, Paulson K, et al. The use of a bespoke website developed for people with dementia and carers: Users' experiences, perceptions and support needs. Dementia (14713012). 2022;21(1):94-113.

517. Wong Y-LI, Kong D, Tu L, Frasso R. “My bitterness is deeper than the ocean”: understanding internalized stigma from the perspectives of persons with schizophrenia and their family caregivers. International Journal of Mental Health Systems. 2018;12(1):1-.

518. Wooldridge JS, Gray C, Pukhraj A, Geller J, Trivedi RB. Understanding communal coping among patients and informal caregivers with heart failure: A mixed methods secondary analysis of patient-caregiver dyads. Heart & Lung. 2019;48(6):486-95.

519. Wootton A, Starkey NJ, Barber CC. Unmoving and unmoved: experiences and consequences of impaired non-verbal expressivity in Parkinson's patients and their spouses. Disability & Rehabilitation. 2019;41(21):2516-27.

520. Wu JM, Tam MT, Buch K, Khairati F, Wilson L, Bannerman E, et al. The impact of respite care from the perspectives and experiences of people with amyotrophic lateral sclerosis and their care partners: a qualitative study. BMC Palliative Care. 2022;21(1):1-11.

521. Wylie K, Carrier HM, Loftus AM, Thilakaratne R, Cocks N. Barriers and Facilitators to Conversation: A Qualitative Exploration of the Experiences of People with Parkinson's and Their Close Communication Partners. Brain Sciences. 2022;12(7):944.

522. Wyse JJ, Ono SS, Kabat M, True G. Supporting family caregivers of Veterans: Participant perceptions of a federally-mandated caregiver support program. Healthcare. 2020;8(3):100441.

523. Xiao J, Chow KM, Chen J, Huang X, Tang S, Wang G, et al. Family-oriented dignity therapy for patients with lung cancer undergoing chemotherapy: How does it work better? Asia-Pacific journal of oncology nursing. 2023;10(1):100168.

524. Yang R, Jensen FB, Edelman LS, Cloyes KG, Donaldson GW, Sanders NA, et al. "Being careful": How much caution is enough? The relational context of fear of older adult falling in older adult-family caregiver dyads. Geriatric Nursing. 2023;51:129-35.

525. Yao H, Chan CHY, Hou Y, Chan CLW. Ambivalence experienced by infertile couples undergoing IVF: a qualitative study. Human Fertility. 2023;26(2):289-301.

526. Yao H, Hou Y, Zhang X, Zhang Q. Extrinsic emotional regulation experienced by lung cancer patients and their family caregivers during progression‐free survival. European Journal of Cancer Care. 2022;31(6):1-8.

527. Yi-Feng Carol L, Zelman DC, Wen-Tao C. Angry characters and frightened souls: Patients and family explanatory models of bipolar disorder in Taiwan. Transcultural Psychiatry. 2018;55(3):317-38.

528. Zagami SE, Roudsari RL, Janghorban R, Allan HT, Ebrahimzadeh Zagami S, Latifnejad Roudsari R. Trying for a second chance: Iranian infertile couples' experiences after failed ART. Journal of Psychosomatic Obstetrics & Gynecology. 2022;43(2):165-70.

529. Zhang Y, Qiu X, Jin Q, Ji C, Yuan P, Cui M, et al. Influencing factors of home exercise adherence in elderly patients with stroke: A multiperspective qualitative study. Frontiers in Psychiatry. 2023;14:1157106.

**Details of dyadic analysis not specified**

530. Baldeo N, D'Souza A, Haag H, Hanafy S, Quilico E, Archambault P, et al. A thematic analysis of patients' and their informal caregivers' gendered experiences in traumatic brain injury. Disability and Rehabilitation: An International, Multidisciplinary Journal. 2023;45(10):1636-45.

531. Boland P, Levack WMM, Hudson S, Bell E. A qualitative exploration of barriers and facilitators to coping experienced by couples when one has multiple sclerosis. International Journal of Therapy & Rehabilitation. 2018;25(5):240-6.

532. Boland P, Levack WMM, Hudson S, Bell EM. Coping with multiple sclerosis as a couple: 'peaks and troughs' - an interpretative phenomenological exploration. Disability & Rehabilitation. 2012;34(16):1367-75.

533. Chen L. Power and ambivalence in intergenerational communication: Deciding to institutionalize in Shanghai. Journal of aging studies. 2017;41:44-51.

534. Chhuom TW, Thompson HJ. Older Spousal Dyads and the Experience of Recovery in the Year After Traumatic Brain Injury. Journal of Neuroscience Nursing. 2021;53(2):57-62.

535. Connor C, Greenfield S, Lester H, Channa S, Palmer C, Barker C, et al. Seeking help for first-episode psychosis: a family narrative. Early Intervention in Psychiatry. 2016;10(4):334-45.

536. Gall TL, Kafi S. The Impact of Breast Cancer on the Mother–Daughter Relationship: Implications of Relationship With God on Attachment. Journal of Spirituality in Mental Health. 2014;16(2):111-32.

537. Graven LJ, Durante A, Abbott L, Bassi E, Howren MB, Grant JS. Self-care Problems and Management Strategies Experienced by Rural Patient/Caregiver Dyads Living With Heart Failure: A Qualitative Study. The Journal of cardiovascular nursing. 2023.

538. Koehn S, McCleary L, Garcia L, Spence M, Jarvis P, Drummond N. Understanding Chinese-Canadian pathways to a diagnosis of dementia through a critical-constructionist lens. Journal of Aging Studies. 2011;26(1):44-54.

539. McGregor LM, Dickson A, Flowers P, Hayes PC, O'Carroll RE. Reclaiming their lives: The decision-making process in living liver donation – An interpretative phenomenological case study analysis of one couple. Psychology & Health. 2014;29(12):1373-87.

540. McPherson CJ, Hadjistavropoulos T, Devereaux A, Lobchuk MM. A qualitative investigation of the roles and perspectives of older patients with advanced cancer and their family caregivers in managing pain in the home. BMC Palliative Care. 2014;13(1):39.

541. Moses Kelly K, Corbett EL, Choko AT, Chikovore J, Kruger K, Mwapasa M, et al. Post-test adverse psychological effects and coping mechanisms amongst HIV self-tested individuals living in couples in urban Blantyre, Malawi. PLoS One. 2019;14(6):e0217534.

542. Ryan M, Mendelsohn JB, Daftary A, Yang M, Bullock S, Lebouché B, et al. Dual pharmaceutical citizenship: Exploring biomedicalization in the daily lives of mixed HIV-serostatus couples in Canada. Social Science & Medicine. 2022;298:N.PAG-N.PAG.

543. Sánchez-Guzmán MA, Paz-Rodríguez F, Espinola Nadurille M, Trujillo-De Los Santos Z. Intimate Partner Violence in Persons With Parkinson's Disease. Journal of Interpersonal Violence. 2022;37(3/4):1732-48.

544. Thomeer MB, Reczek C, Umberson D. Relationship dynamics around depression in gay and lesbian couples. Social Science & Medicine. 2015;147:38-46.

545. Thomeer MB, Umberson D, Pudrovska T. Marital Processes around Depression: A Gendered and Relational Perspective. Society and mental health. 2013;3(3):151-69.

**Methodologic with no care partnerships or dyadic analysis details not specified**

546. Eisikovits Z, Koren C. Approaches to and Outcomes of Dyadic Interview Analysis. Qualitative Health Research. 2010;20(12):1642-55.

547. Haywood C, Lawlor MC. Understanding lived experiences through multiple perspectives: Caregiving as an exemplar. Journal of Occupational Science. 2019;26(1):128-39.

548. Hochman Y, Segev E, Levinger M. Five Phases of Dyadic Analysis: Stretching the Boundaries of Understanding of Family Relationships. Family Process. 2020;59(2):681-94.

549. Koren C. A Complex Unit Interviews Analysis Approach in Qualitative Social Work Research. British Journal of Social Work. 2023;53(6):3258-76.

550. Manning J, Kunkel A. Qualitative Approaches to Dyadic Data Analyses in Family Communication Research: An Invited Essay. Journal of Family Communication. 2015;15(3):185-92.

551. Norlyk A, Haahr A, Hall E. Interviewing with or without the partner present? - an underexposed dilemma between ethics and methodology in nursing research. Journal of Advanced Nursing (John Wiley & Sons, Inc). 2016;72(4):936-45.

552. Reczek C. Conducting a Multi Family Member Interview Study. Family Process. 2014;53(2):318-35.

553. Taylor B, de Vocht H. Interviewing Separately or as Couples? Considerations of Authenticity of Method. Qualitative Health Research. 2011;21(11):1576-87.

554. Van Parys H, Provoost V, De Sutter P, Pennings G, Buysse A. Multi family member interview studies: a focus on data analysis. Journal of Family Therapy. 2017;39(3):386-401.

555. Vogl S, Schmidt E-M, Zartler U. Triangulating perspectives: Ontology and epistemology in the analysis of qualitative multiple perspective interviews. International Journal of Social Research Methodology: Theory & Practice. 2019;22(6):611-24.

556. Vogl S, Zartler U, Schmidt E-M, Rieder I. Developing an analytical framework for multiple perspective, qualitative longitudinal interviews (MPQLI). International Journal of Social Research Methodology: Theory & Practice. 2018;21(2):177-90.

**Appendix G: Supplementary tables and figures**

Supplementary Table 1. Included sources of evidence characteristics

| **First author, Year** | **Country** | **Disease process, injury, illness condition** | **Study aim(s)** | **Overall study design** | **Epistemological position or theoretical perspective**^*^ | **Methodological approach** |
| --- | --- | --- | --- | --- | --- | --- |
| Abendschein, 2021 | USA | Stroke | To investigate interdependence in couples after a stroke, focusing on the nuances of contextually based relational changes. | Qualitative | Interpretive paradigm | Phronetic iterative approach |
| Abshire Saylor, 2023 | USA | Heart failure | To explore dyadic perspectives on the context of caregiving for persons with heart failure across multiple contextual domains. | Mixed methods | Naturalistic paradigm | Cross-sectional, convergent, mixed methods  Qualitative description for qualitative stage |
| Abulaiti, 2022 | China | Elderly individuals with disabilities | To describe the dyadic care experiences of elderly individuals with disabilities and their caregivers from the perspective of family resilience. | Qualitative | NR | Descriptive phenomenology |
| Allen, 2021 | USA | Type 1 diabetes | To understand couple-level interactions related to diabetes technology use. Speciﬁcally, to understand how partners supported people with diabetes in using insulin pumps and continuous glucose monitoring and how technology affected the couples’ coping and management of diabetes. | Qualitative^†^ | NR | Qualitative description |
| Allen, 2023 | USA | Type 1 diabetes | To examine the perceptions of a diabetes care and education intervention (SHARE plus) and its effects on communication, collaboration, and involvement in day-to-day diabetes management in older adults with Type 1 diabetes and their care partners. | Qualitative | NR | Qualitative description |
| Antoine, 2013 | France | Breast cancer | To identify, on an individual and dyadic level, the lived experience (psychological, emotional, familial, and social as well as couple functioning) of young couples undergoing hormone therapy for breast cancer. | Qualitative | NR | Interpretative phenomenological analysis used in an original dyadic perspective |
| Antoine, 2018 | France | Anorexia nervosa | To examine the functioning of couples in which one partner lives with anorexia nervosa (including their difficulties, impact of illness on their relational dynamics, and partners' individual and dyadic adjustment modes for coping with the illness). | Qualitative | NR | Qualitative approach (interpretative phenomenological analysis) in a dyadic way |
| Berridge, 2020 | USA | Multiple chronic conditions | To compare preferences of older adult women and their adult children for three remote monitoring technologies at the dyad and aggregate level. | Qualitative | NR | Dyadic study using cognitive-based interview probing and value-centered design |
| Birtwistle, 2022 | UK | Myocardial infarction | To explore the lived experiences of patients’ engagement with physical activity post-myocardial infarction, together with the experiences of their family. | Qualitative longitudinal | Phenomenology and hermeneutics | Qualitative longitudinal approach drawing on interpretative phenomenological analysis |
| Boulicault, 2023 | USA | Psychiatric disorders | To analyze the complex ways in which family relationships can affect deep brain stimulation (DBS) trial participation, and how DBS trial participation in turn influences family relationships. | Qualitative | Constructionist epistemology | NR |
| Boyle, 2013a | UK | Dementia | To explore social processes of everyday decision-making by people with dementia and their spouses living at home, focusing on the management of ﬁnances and ﬁnancial decision-making. | Qualitative | NR | Ethnography |
| Boyle, 2013b | UK | Dementia | To explore social processes of everyday decision-making by people with dementia and their spouses living at home, focusing on how much influence women with dementia exerted in decisions about what housework had to be done and who was responsible for doing this work. | Qualitative | NR | Ethnography |
| Boyle, 2013c | UK | Dementia | To explore social processes of everyday decision-making by people with dementia and their spouses living at home, focusing on the role of gendered support in inﬂuencing whether people with dementia are enabled to exercise their decision-making capacity and, in turn, if their decisional autonomy is facilitated. | Qualitative | NR | Ethnography |
| Boyle, 2014a | UK | Dementia | To explore social processes of everyday decision-making by people with dementia and their spouses living at home, focusing on the choice and control that women with dementia exercised over cooking. | Qualitative | NR | Ethnography |
| Boyle, 2014b | UK | Dementia | To explore social processes of everyday decision-making by people with dementia and their spouses living at home, focusing on the potential for at least basic agency in people with advanced dementia. | Qualitative | NR | Ethnography |
| Boyle, 2017 | UK | Dementia | To explore social processes of everyday decision-making by people with dementia and their spouses living at home, focusing on whether people with advanced dementia demonstrate emotional communication and reflexivity within their personal relationships. | Qualitative | NR | Ethnography |
| Brooks, 2014 | UK | Chronic fatigue syndrome/myalgic encephalomyelitis | To explore the beliefs and experiences of both chronic fatigue syndrome/myalgic encephalomyelitis patients and their signiﬁcant others recruited in dyads. | Qualitative | Phenomenology | Interpretative phenomenological analysis |
| Buck, 2013 | USA | Heart failure | To provide qualitative evidence for the validity of a typology of patient and caregiver dyadic interdependence in heart failure (HF) care. Specifically to: 1) present exemplar cases for each of the four dyadic types in the Dyadic HF care typology; 2) discussing how the patient’s HF self-care varies among types; and 3) conducting a content analysis of the patient and caregiver narratives. | Qualitative part of larger study | NR | Instrumental case study (case = dyad) |
| Chan, 2017 | Hong Kong | Schizophrenia | To explore discrepancies in conceptions of spirituality between patients and their family care-givers, as well as the way in which these affect the family dynamic and patients’ illness recovery. | Qualitative part of larger study | NR | Qualitative approach based on grounded-theory principles |
| Catona, 2022 | USA | Gynecological cancer | To describe the experiences of cancer caregivers and compare them with patients' assessment of physical, emotional, and financial demands of cancer caregiving. | Qualitative part of larger study | NR | NR |
| Chen, 2015 | China | Multiple chronic conditions | To describe what elders and their children in Shanghai experience during the decision-making process around institutionalization. | Qualitative | Phenomenology | Phenomenology |
| Collaço, 2019  Collaço, 2021a | UK | Prostate cancer | To explore the challenges on the family unit of younger couples affected by prostate cancer, to better understand family dynamics when affected by prostate cancer and inform how pathways of care can be developed to address their needs and that of their children (Collaço, 2019).  To explore the experiences and needs of younger men and their partners affected by prostate cancer to inform service provision and develop interventions appropriate to need (Collaço, 2021a).  *[Core theme from findings of Collaço, 2021a associated with the challenges faced by younger couples related to parenthood and family functioning published in Collaço et al., 2019.]* | Qualitative part of larger mixed methods study | NR | Multiperspective analysis methodology |
| Conroy, 2017 | South Africa | HIV | To examine how relationships could interfere with or support adherence in couples affected by HIV from rural KwaZulu-Natal, South Africa. | Qualitative | NR | NR |
| Conroy, 2018 | Malawi | HIV | To examine the interplay of marital inﬁdelity, food insecurity, and couple instability on dyadic coordination and adherence to antiretroviral therapy in Malawi. | Qualitative | NR | NR |
| Conroy, 2019 | Malawi | HIV | To examine how alcohol consumption may impact couple relationships and antiretroviral therapy adherence, how partners may impact each other’s alcohol consumption and antiretroviral therapy, and whether one partner’s alcohol use influences the other partner’s antiretroviral therapy adherence (in accordance with interdependence theory). | Qualitative part of larger study | NR | NR |
| Conroy, 2020 | Malawi | HIV | To understand barriers and facilitators of alcohol use, and potential intervention options with couples, to inform a future alcohol intervention for couples living with HIV to reduce alcohol use and improve adherence to antiretroviral therapy. | Qualitative part of larger mixed methods study | NR | NR |
| Constant, 2022 | France | Parkinson’s disease | To explore the experience of couples confronted with advanced Parkinson's Disease and to put into perspective the patient and his or her partner meaning-making regarding their couple functioning. | Qualitative | NR | Interpretative phenomenological analysis with dyadic/ multiperspective design |
| DeGroot, 2021 | USA | Heart failure | To explore congruence of ventricular assist device patient and caregiver perspectives regarding end of life, definitions of quality of life, and meaning in life while managing the uncertainty of living with a ventricular assist device. | Qualitative part of larger mixed methods study | NR | NR |
| Demirtepe-Saygili, 2022 | Turkey | Multiple sclerosis | To reveal the illness experiences of the couples including one partner with multiple sclerosis. | Qualitative part of larger study | NR | Dyadic qualitative methodology |
| Dobrina, 2016 | Italy | Cancer | To explore needs and wishes in the last week of life of patients at home and seek out the views of the family caregivers. | Qualitative | Phenomenology | Phenomenology (based on Husserl’s descriptive phenomenological perspective) |
| Ekelund, 2010 | Sweden | End-stage renal disease | To elicit psychological and psychosocial problems existing in patients in dialysis and their partners. | Qualitative longitudinal | NR | NR |
| Ekstam, 2011 | Sweden | Stroke | To identify and describe two couples’ approaches to changes in everyday life during the ﬁrst year after a stroke. | Qualitative longitudinal | NR | Prospective longitudinal case study (case = couple) |
| Engeli, 2016 | Switzerland | Malignant melanoma | To analyse resilience as per Antonovsky’s sense of coherence. | Qualitative longitudinal | NR | Longitudinal qualitative partner study |
| Eriksson, 2010 | Sweden | Myocardial infarction | To describe and interpret couples’ thoughts and expectations about their future life after the patient’s discharge following acute myocardial infarction. | Part of larger qualitative longitudinal study (single time point) | No single truth exists and need to piece narratives together to discover possible interpretation. | Descriptive design |
| Farquhar, 2017 | UK | Cancer and chronic obstructive pulmonary disease (COPD) | To identify the educational needs of carers of patients with breathlessness due to advanced disease (i.e. to establish what these carers want to learn about) in order to provide an evidence base for the intervention’s content. | Qualitative part of larger study | NR | NR |
| Ferreira, 2020 | Australia | Chronic obstructive pulmonary disease (COPD) | To understand the experience of living with, and responding to, severe chronic breathlessness in people with COPD from the perspective of the patient and their carer, before commencing on a trial medication. | Qualitative part of larger study | NR | NR |
| Fonner, 2021 | Tanzania | HIV | To understand and describe decision-making and dyadic-level influence among members of serodiscordant couples regarding (1) participation in a dyadic-based research study involving HIV self-testing and access to pre-exposure prophylaxis (PrEP), and (2) utilization of PrEP and antiretroviral therapy. | Qualitative sub-study within larger observational cohort study | NR | NR |
| Gamarel, 2016 | USA | HIV | To explore how partners in male couples who were coping with HIV described their orientation towards their involvement in each other’s health, and the association of their orientation with giving and receiving support.  To provide a more nuanced understanding of how a “we” perspective may be operationalized in male couples’ relationships, as well as how social support manifests among couples with more autonomous orientations. | Qualitative part of larger study | NR | NR |
| Goldsmith, 2016 | USA | Cancer | To examine family communication patterns among oncology patients and their caregivers and to identify common characteristics among four different types of family caregivers.  Speciﬁcally, to explore the extent to which oncology patient–caregiver dyads share a family communication pattern and to determine common communication characteristics of caregiver types based on speciﬁc family communication patterns. | Qualitative | NR | NR |
| Gorman, 2020 | USA | Breast cancer | To examine how young adult breast cancer survivors and their partners appraise and manage their sexual health and intimate relationships after cancer by examining and comparing couple members’ perspectives and experiences. | Qualitative | NR | NR |
| Grivel, 2023 | France | Digestive cancer | To explore the experience of being a caregiver of digestive cancer patients and compare the patient and caregiver perspectives to highlight differences and similarities. | Mixed methods | NR | Cross-sectional, prospective (quantitative) and phenomenological approach (qualitative) studies with convergent design where quantitative and qualitative data collected simultaneously but separate analysis of 2 types of data |
| Hedman, 2019 | Sweden | Alzheimer’s disease | To describe, from an individual perspective, how the agency and communion of persons with Alzheimer’s disease is viewed by both the persons with Alzheimer’s themselves and their spouses. | Qualitative part of larger study | NR | NR |
| Heid, 2016 | USA | Aging adult requiring admission for short-term rehabilitation | To address how older adults inﬂuence their daily care when their preferences conﬂict with those of their adult daughter caregivers. | Qualitative | NR | NR |
| Hellström, 2013 | Sweden | Dementia | To explore disclosure preferences and focus on what couples living with dementia want to know and tell about the disease. | Qualitative part of larger study | NR | NR |
| Hellström, 2016 | Sweden | Dementia | To explore how the future is understood by couples living with dementia. | Qualitative part of larger study | NR | NR |
| Hendryckx, 2024 | Canada | Traumatic brain injury | To (1) explore and conﬁrm the perspective of individuals with traumatic brain injury living in the community and their family caregivers on behaviours they consider challenging and, (2) identify overlapping or distinct views on challenging behaviours. | Qualitative | Pragmatic realism | Inductive qualitative descriptive design |
| Hopkinson, 2016 | UK | Cancer | To examine interdependency in the experience of involuntary weight loss and poor appetite of patients with advanced cancer and their family carers and to generate understanding that can inform clinical practice. | Qualitative sub-study of exploratory trial of intervention^†^ | NR | Interpretive phenomenological approach |
| Huang, 2021 | USA | HIV | To explore the dyadic aspects of family support among Asian Americans living with HIV (AALHIV) and their family caregivers. Specifically: (1) the perceptions of AALHIV regarding their experience of the family support they received; (2) from a caregiver’s perspective, what kind of care did they provide to the HIV-positive family members; and (3) whether perceived family support obtained by the AALHIV was expected. | Qualitative | NR | NR |
| Hudson, 2016 | UK | Endometriosis | To explore the concept of biographical disruption from a dyadic perspective, utilising data from an exploratory, qualitative study (ENDOPART) that investigated the impact of endometriosis on women and their male partners. | Qualitative | Interpretivist relational approach | NR |
| Iannarino, 2022 | USA | Cancer | To explore the shared decision-making styles of young adult patients, partners, parents, and other family members regarding fertility preservation before beginning cancer treatment based on the DECIDE typology. | Qualitative part of larger study | NR | NR |
| Jere, 2023 | Malawi | HIV and cardiometabolic disorders | To categorize and describe the explanatory models of cardiometabolic disorders held by persons living with HIV and cardiometabolic disorders (hypertension and/or diabetes) and their primary partners in southern Malawi. | Qualitative | NR | NR |
| Kano, 2022 | USA | Cancer | To compare the experiences of Sexual and Gender Minority cancer patients and their self-identiﬁed cancer caregivers with those of heterosexual, cisgender cancer patient/caregiver dyads receiving care at the University of New Mexico Comprehensive Cancer Center. | Qualitative part of larger multi-methods study | NR | Multi-methods research design |
| Kitko, 2015 | USA | Heart failure | To determine the prevalence of incongruence between heart failure patient–caregiver dyads, areas of incongruence, and the impact on individuals in the dyadic relationship. | Part of larger qualitative longitudinal study | NR | NR |
| Lamore, 2019 | France | Breast cancer | To explore couples’ experiences of mastectomy and breast reconstruction decision-making, the month following the mastectomy. | Qualitative part of larger study | Phenomenology | Interpretative phenomenological analysis |
| Loaring, 2015 | UK | Breast cancer | To explore and understand couples’ lived experiences of their embodied selves and intimate relationships. | Qualitative | Phenomenology  Differing personal perspectives on the world can be reconciled by a third party focusing on patterns of meaning-making. | Interpretative phenomenological analysis with multiperspectival design |
| Loup, 2023 | USA | Dementia | To demonstrate how rural patients’ daily life needs can be summarized through life-space map visualizations. | Qualitative^†^ | NR | NR |
| Manceau, 2023 | France | Parkinson’s disease | To explore the experience of couples and what it means when one of the partners is in the “honeymoon period” of Parkinson's Disease. | Qualitative | Phenomenology and hermeneutics  Each partner's experience and perceived meaning are indivisible and interdependent within the couple, which allows work on the couple dimension. | Interpretative phenomenological analysis with a dyadic multiperspectival design |
| Martin-Matthews, 2022 | Canada | Aging adult using or receiving home care | To examine how older people as clients, carers and home support workers collaborate to achieve an appropriate care composition as they negotiate the situated and concrete practical contingencies of people’s lives to optimise the home care experience and its outcomes.  To examine more explicitly the complexity, nuance and collaboration of care coordination among members of the ensemble paying particular attention to the role played by the older person as client, and we stress the bidirectional nature of care relationships. | Qualitative part of larger mixed methods study^†^ | NR | Generic qualitative research approach |
| McCarthy, 2015 | USA | Stroke | To investigate stroke survivors’ and caregiving spouses’ individual perspectives on survivor cognitive and physical functioning and the extent to which incongruence between partners’ perceptions affects spouse depressive symptoms and overall mental health. | Mixed methods^†^ | NR | Mixed methods, with quantitative survey data from 35 couples and qualitative interview data from a subsample of 13 couples  Interpretive description for qualitative part |
| McCarthy, 2020 | USA | Stroke | To enhance understanding about relationship challenges among stroke dyads and to identify implications for direct practice in social work. | Qualitative | NR | Interpretative description |
| McCarthy, 2022 | USA | Stroke | To provide an illustrative example of how social determinants of health manifest in a large Midwestern city, in the context of stroke survivors and family caregivers.  To explore barriers to and facilitators of successful adjustment to stroke and suggest implications for practice in social work and related disciplines. | Qualitative^†^ | NR | Case study (case = dyad) |
| Miller, 2013 | USA | Cancer | To address a specific challenge salient to patients and their partners in a variety of illness contexts: negotiating the nature of their relationship through treatment and survivorship. Specifically, to examine the notion of couple identity, which refers to the partners’ sense of who they are as a unit. | Qualitative | Rhetorical/normative theoretical approach | Rhetorical/normative approach |
| Mitchell, 2021 | USA | HIV | (1) To explore facilitators to decision-making to use HIV prevention strategies and (2) to describe the prevention strategies being used by same gender male couples in new relationships. | Qualitative | NR | NR |
| Moore, 2020 | UK | End-stage renal disease | To explore the impact of 3 early phases of renal dialysis, namely pre-dialysis, starting dialysis, and establishing dialysis, on dyadic relationships.  To explore the impact of early dialysis on spousal-type dyads, with a focus on the impact of dialysis on the dyadic relationship. | Qualitative | Critical realism | NR |
| Mosher, 2016 | USA | Colorectal cancer | To identify caregivers’ key challenges in coping with their family member’s advanced colorectal cancer. | Qualitative | NR | Qualitative approach that elicited both patient and caregiver perspectives |
| Mosher, 2017 | USA | Colorectal cancer | To identify positive changes in patients with advanced colorectal cancer and their primary family caregivers since the diagnosis.  To compare self-reported and patient-reported positive changes in caregivers as well as self-reported positive changes within each patient-caregiver dyad. | Qualitative | NR | NR |
| O’Keeffe, 2020 | UK | Traumatic brain injury | To explore the impact of traumatic brain injury on couple relationships, from the perspective of both injured and uninjured partners in the relationship. | Qualitative | Phenomenology and hermeneutics | Interpretative phenomenological analysis with dyadic/ multiperspective design |
| Ohlsson-Nevo, 2012 | Sweden | Colorectal cancer | To describe the experience of life the ﬁrst year after surgery from the perspective of persons treated for colorectal cancer and their partners. | Qualitative | NR | Qualitative descriptive interview study |
| Patel, 2016a | India | HIV | To examine factors that mitigate or heighten HIV risk among HIV-negative wives in serodiscordant relationships in Gujarat, India.  To understand the experiences of sex communication and behavior among serodiscordant couples within the context of dominant cultural norms to inform the development of a HIV prevention counseling program for serodiscordant couples in India. | Qualitative | NR | Grounded theory |
| Patel, 2016b | India | HIV | To present the challenges that affect a serodiscordant couple’s attempts to prevent HIV transmission to the HIV-negative partner. | Qualitative^†^ | NR | Intrinsic case study approach (case = dyad) |
| Rance, 2017 | Australia | Hepatitis C | To explore the particularities of acquiring and living with Hepatitis C among couples who inject drugs. | Qualitative part of larger study | NR | Methodology that positioned partnerships as the primary unit of analysis |
| Rando, 2022 | USA | Posttraumatic stress disorder (PTSD) | To descriptively explore veterans’ and partners’ attitudes, histories, and goals prior to partner involvement in the context of PTSD-focused treatment. | Qualitative part of larger study | NR | NR |
| Rapelli, 2023 | Italy | Heart failure | To explore the lived experience of patients with left ventricular device and their caregivers before discharge from the hospital in order to identify their needs and concerns related to this important and delicate life transition and the preparation of home care tasks.  To identify the impact of the illness on both members of the dyad exploring their reciprocal interdependence and giving voice to both the recipients of the cure and their caregivers, describing their personal experiences and analyzing similarities or discrepancies in the narratives. | Qualitative | NR | Dyadic phenomenological hermeneutic research design |
| Raybone, 2019 | UK | Axial spondyloarthritis | To explore how and why axial spondyloarthritis impacts on partner relationships according to individuals with axial spondyloarthritis and their partners. | Qualitative | NR | NR |
| Retrum, 2013 | USA | Heart failure | To examine ways in which patient-caregiver dyads talk about how they manage the challenges of living with heart failure, including heart failure care needs, the future, and coping with illness.  **Secondary objective:** to explore in what ways congruence or incongruence between patient and caregiver perspectives may emotionally impact the patient or caregiver (emotional distress) and whether the age or relationship of the dyad members is related to congruence in dyads. | Qualitative part of larger study | NR | NR |
| Roberto, 2013 | USA | Mild cognitive impairment | To investigate how cognitive problems aﬀect spousal relationships over time. | Part of larger qualitative longitudinal study | Symbolic interactionism | NR |
| Rodham, 2010 | UK | Fibromyalgia | To explore the lived experiences of both those with fibromyalgia syndrome and their spousal carers. | Qualitative | NR | Interpretative phenomenological analysis |
| Rodrigues, 2022 | Austria & Slovenia | Individuals receiving care services and informal care | To develop a framework for the analysis of pathways into intergenerational caregiving to older people provided by family members using life course concepts of key turning events in life, cumulative processes, and linked lives within the family realm. | Qualitative | NR | Dyadic study design |
| Rowland, 2018 | USA | Coronary arteria disease | To describe the couple-focused facilitators and barriers to healthy eating and physical activity behaviors in patients and spouses from both of their perspectives following cardiac rehabilitation. | Qualitative sub-study of randomized controlled trial | NR | Qualitative description |
| Ruark, 2024 | Malawi | HIV and cardiometabolic disorders | To examine the process of communal coping in Malawian couples living with HIV and cardiometabolic disorders as well as how aspects of couple functioning such as shared illness appraisal, support, collaboration, and relationship quality are connected to dyadic management of the disease. | Qualitative part of larger mixed methods study | NR | NR |
| Sauvé, 2020 | Canada | Infertility | To provide a more detailed portrait of the marital benefits derived during the course of fertility treatment in order to expand knowledge of their nature and meaning for each couple.  To describe each partner’s individual point of view within a dyadic perspective that considers the impact of each person’s reality on the other, thus providing access to the relational aspects involved in experiencing infertility as a couple.  To explore the meaning of marital benefits for each partner individually and also allowed to shed light on this meaning within the dyad.  To examine and contrast each partner’s account to explore factors that may have contributed to the emergence of marital benefits within each dyad, hence potentially providing key information about useful intervention targets. | Qualitative sub-study of larger quantitative study | Phenomenology and hermeneutics | Interpretative phenomenological analysis approach |
| Senden, 2015 | Belgium | Cancer | To explore lived experiences of older cancer patients, family caregivers and their interaction. | Qualitative | NR | Grounded theory approach |
| Serçe, 2020 | Turkey | Hematologic cancer | To explore (a) the interaction between patients receiving treatment for hematological cancer in a hematology-oncology clinic and their family caregivers and (b) perceived changes in lives of the affected patients and family caregivers. | Qualitative | NR | Qualitative descriptive design with a dyadic approach |
| Shilling, 2017 | UK | Cancer | To explore the impact of extended cancer survival on broader aspects of life and wellbeing such as occupational, financial and family life for patients with advanced cancer and their nominated informal caregivers.  *(Article focuses on one of the overarching themes, that of ‘uncertainty’, and how this construct pervaded many different aspects of patients’ and caregivers’ lives and how the general concept varied within the accounts of patient-caregiver dyads.)* | Qualitative part of larger study | NR | NR |
| Smith, 2022 | USA | Breast cancer | To explore the role of dyadic support across the contraceptive decision-making process between young adult breast cancer survivors and their partners.  To understand the role of partner support in contraceptive decision-making. | Qualitative part of larger study | NR | NR |
| Solomon, 2018 | USA | Terminal illness | To describe and interpret how mothers and daughters: (a) perceive relationship quality and (b) perceive how relationships have developed over time through health, chronic illness, and hospice. | Qualitative | Interpretive turn and social construction | Interpretative description |
| Spangler, 2018 | Kenya | HIV | To explore current perceptions of HIV stigma and self-disclosure, and their effects on engagement in prevention of mother-to-child transmission and HIV care among self-disclosed couples in southwest Kenya. | Qualitative | NR | NR |
| Steinberg, 2024 | USA | Autism | To compare dyadic perspectives (from within the same family unit) about what was important to parents versus autistic young adults in the transition to adulthood. Speciﬁcally, to explore the experience of transitioning to adulthood among parent–young adult dyads from an economically disadvantaged, urban school district and to understand how perspectives on the transition process converge and diverge for youth and their parents in the same families, especially when the autistic youth may have higher needs for support with daily tasks. | Qualitative part of larger study | NR | NR |
| Sterba, 2014 | USA | Breast cancer | To explore quality-of-life in African American breast cancer survivor–caregiver dyads at the end of primary treatment and in the post-treatment period with a focus on spiritual well-being. | Qualitative | NR | NR |
| Sud, 2021a | UK | Cardiometabolic syndrome and severe mental illness | To explore the impact of comorbid cardiometabolic risk, metabolic syndrome and related diseases and severe mental illness on the caring dyad. Specifically, to 1) explore the role of the caring dyad and 2) explore the utility of the caring dyad as the unit of analysis (as opposed to individual experiences) in this space. | Qualitative | NR | NR |
| Tan, 2018a | USA | HIV | To explore the extent to which Black Men who have Sex with Men in primary relationships may be involved in their partner’s HIV care and to describe these patterns of involvement. | Qualitative part of larger study | NR | NR |
| Tan, 2018b | USA | HIV | To explore dyadic HIV care among black Men who have Sex with Men in couples, to identify care engagement challenges and forms of partner support, and to explore couple’s dynamics around care engagement. | Qualitative part of larger study | NR | NR |
| Thomeer, 2015 | USA | Physical illness | To examine how mid- to later-life husbands and wives in long-term heterosexual marriage conceptualize their own—or their spouse's—emotion work during periods of their own—or their spouse's—physical health problems including how these explanations are different for husbands compared to wives. | Qualitative part of larger study | NR | NR |
| Thomson, 2020 | Australia | Parkinson’s disease | To examine the significance and meaning of deep brain stimulation related changes in personality and self for patients and caregivers. | Qualitative longitudinal | Realist paradigm | NR |
| Treloar, 2016 | Australia | Hepatitis C | To examine the experiences of couples who inject drugs in relation to knowledge of, decisions about and management of Hepatitis C treatment. | Qualitative | NR | NR |
| Tripathee, 2020 | UK | Obesity | To investigate how partner support is received and utilised by men trying to change their dietary practices and physical activity in order to lose weight through a weight management programme designed for obese/overweight men, explored from both partners’ perspectives, in order to facilitate deeper understanding of the complexities involved when behaviour change is attempted in the cohabiting couple context. | Qualitative | NR | NR |
| Umberson, 2016 | USA | Physical illness | To explore how men and women in gay, lesbian, and heterosexual marriages construct the illness experience, and how these co-constructions of illness shape their provision and/or receipt of care and illness-related stress when one of them is ill. | Qualitative | NR | Dyadic study design |
| Ummel, 2016a | Canada | Advanced renal failure | To provide a better understanding of how living kidney transplantation donors and recipients experience the transplantation process as an interactive dyad.  To examine the extent to which Mauss’s anthropological gift exchange could be helpful in framing the dyadic experience of living kidney transplantation. | Qualitative | Constructivist interpretativist paradigm adopting phenomenological stance | Interpretative phenomenological analysis |
| Vandenberg, 2024 | USA | Multiple chronic conditions including terminal illness | To understand communication behaviors used by assisted living residents and their informal caregivers (i.e., family members or friends) related to death and dying, and address communication needs or opportunities applicable to end of life care in assisted living. | Qualitative part of larger study^†^ | NR | NR |
| Wang, 2021 | China | Cognitive impairment | To explore and understand the dyadic experience of persons living with mild cognitive impairment and mild dementia and their care partners from a person-centered care lens in the social and cultural context of China. | Qualitative part of larger study | Phenomenology and hermeneutics | Descriptive qualitative approach |
| Wang, 2022 | China | Dementia | To explore dyadic experiences of people with dementia and their spousal caregivers and develop a culturally and contextually-sensitive understanding of person-centered dementia care in home-based settings. | Qualitative part of larger study | Naturalist philosophy | Descriptive qualitative approach |
| Werner, 2021 | Israel | Cystic fibrosis | To examine how adults with cystic fibrosis and their partners cope as a couple with the illness, and what meanings each partner and the couple as a unit attribute to the experience.  To expand our knowledge of couple relationships in case of cystic fibrosis in adulthood, in particular, dyadic coping. | Qualitative part of larger study | NR | Hermeneutic (interpretive) phenomenological approach |
| White, 2016 | UK | Colorectal cancer | To explore couples' experiences of colorectal (bowel) cancer, focusing on the transition after treatment. | Qualitative | NR | Qualitative methodology |
| Wise, 2010 | USA | End-stage renal disease | To study couples who had successfully adopted short daily home hemodialysis to understand shared illness experience of dyads to identify factors that can help dialysis centers develop support systems to meet the complex needs of couples. | Mixed methods | NR | Descriptive study combining quantitative and qualitative data |
| Wood, 2020 | USA | Multiple sclerosis | To explore spousal support and behavior in fostering self-determined physical activity engagement of adults with multiple sclerosis. | Qualitative | NR | NR |
| Wrubel, 2010 | USA | HIV | To explore how the relationship dynamics within gay male couples affect the giving and receiving of support for antiretroviral therapy adherence. | Qualitative part of larger study | NR | NR |

NR: not reported

^*^Philosophical stance that informed methodology reported by authors ^†^secondary analysis

Supplementary Table 2. Methods of analysis

| **First author, Year** | **Method(s) of analysis**  **Software** | **Summary of analytic steps** | **Sequence of analysis category** | **Dyadic step** |
| --- | --- | --- | --- | --- |
| Abendschein, 2021 | Phronetic iterative approach  Software: NR | - Assigned word or short phrase that captured the action in each portion of text and then create categories and codebook; codebook applied to data. - Interrogated connections among categories and literature on interdependence and stroke with higher level interpretations of data with goal of constructing explanations for the participants' explanations. - Analysed data at couple level for additional insights that emerge within couples by pairing data for all couples in which both members participated and had excerpts about interdependence in interview data; data for each couple examined for how couple talked about stroke and *then looked for notable experiences across couples.* | Dyadic analysis to further explore one or two relational themes or categories from analysis at individual level, then across cases | Transcripts analyzed from each dyad as a whole |
| Abshire Saylor, 2023 | Qualitative description approach  Software: F4analyse | - Initial coding of all data into contextual domains using qualitative descriptive approach. - Coding and quantizing dyads by domain by extracting quotes from both patient and caregiver related to each domain and examining agreement between patient and caregiver and noting any differences in perceptions of the domain. - Within dyad, read and rated (quantized) domains as positive, neutral, or challenge within the context of all other domains (“values” domain could not be quantized, and instead, key words summarizing values expressed displayed) - For most domains, value assignments were straightforward, but sometimes, an interpretive level of analysis through study team discussion and consensus was necessary to fully explore the interaction of overlapping contextual domains. - *Grouped dyads by quantitative number of challenges.* | Analysis at individual level, then at dyadic level informed by results from analysis at individual level, then across dyads | Side-by-side readings to compare and categorize themes, codes, or **accounts** within each dyad |
| Abulaiti, 2022 | Colaizzi method  Software: NVivo | - Data analyzed for each participant. - Focused on the perceptions and experiences of dyads, compared overlaps and contrasts, and enhanced understanding of the dyads’ relationships and experiences. | Analysis at individual level and then at dyadic level | Side-by-side readings to compare and categorize themes, codes, or **accounts** within each dyad |
| Allen, 2021  *Cite:*  *Boeije, 2002* | Constant comparison approach  Software: Microsoft Excel, NVivo | - Developed codebook to identify whether and how spouses supported person with diabetes (PWD) to use insulin pumps and continuous glucose monitoring (CGM) and how technology affected couples’ management of diabetes. - Second round of coding conducted to create a new codebook to speciﬁcally explore couple-level interactions with diabetes technology, including insulin pumps and CGM. - Codes on the PWD level, spouse level, and couple level compared, contrasted, and collapsed to develop corresponding themes through discussion. Sub-themes developed within each theme after identifying patterns in couples’ experiences from responses around diabetes technology. | Analysis at individual level then at dyadic and aggregate (group) levels | Coded within couples or dyadic-level codebook |
| Allen, 2023  *Cite:*  *Boeije, 2002*  *Eisikovits & Koren, 2010* | Constant comparison approach  Software: NVivo | - Initial code book developed by coding 3 partner interviews and then remaining partner and person with diabetes (PWD) interviews coded. Using an open coding approach, new codes were added ad hoc. - To identify patterns at the PWD, partner, and dyad levels, compared and contrasted codes within PWD interviews, partner interviews, and dyad interviews. - Codes were compared, contrasted, and collapsed to develop corresponding themes until thematic saturation was achieved. | Analysis at individual level, then at dyadic and aggregate (group) levels based on results from analysis at individual level | Side-by-side readings to compare and categorize themes, **codes,** or accounts within each dyad |
| Antoine, 2013 | IPA used in an original dyadic perspective  Software: NR | - Data analyzed for each participant to develop individual schema. - To report dyadic functioning, identified convergences and divergences in couple's interviews including processes that allowed them to adjust to situation. - At the end of each stage (patient-partner-couple), analyses combined to create global schema. | Analysis at individual level and then at dyadic level | Side-by-side readings to compare and categorize themes, codes, or **accounts** within each dyad |
| Antoine, 2018  *Cite:*  *Eisikovits & Koren, 2010* | IPA in a dyadic way  Software: NR | - For each couple, all possible themes and sub-themes were identified for the patient, the partner and the couple as a whole. - Identified shared themes, connections and discrepancies between partners. Based on these couples’ analyses, an overall list of themes and sub-themes was developed with reference to the transcripts of the couples. | Sequence not specified | Side-by-side readings to compare and categorize **themes,** codes, or accounts within each dyad |
| Berridge, 2020  *Cite:*  *Eisikovits & Koren, 2010* | Qualitative methods of dyadic analysis paired with techniques of grounded theory  Software: ATLAS.ti, Microsoft Excel | - Open and axial coding techniques of grounded theory were used to analyze the interviews individually, followed by analysis of each set of dyads:   - Analyzed each interview of dyad as single unit through open coding and production of themes. This enabled the next step of within-dyad and aggregated comparisons of the two participant groups. - Dyadic analysis by examining the themes and areas of agreement and disagreement from each dyad through side-by-side readings of individually coded dyad members’ responses to the same questions, dyad by dyad. Areas of agreement and disagreement were identified through closed coding and documented on an Excel spreadsheet matrix, indicating areas of agreement and disagreement for each dyad by each technology. | Analysis at individual level, then at dyadic and aggregate (group) levels based on results from analysis at individual level | Side-by-side readings to compare and categorize themes, **codes,** or accounts within each dyad |
| Birtwistle, 2022  *Cite:*  *Reczek, 2014* | IPA  Software: NR | - Longitudinal analysis of dyad experiences   - Transcripts analyzed from each dyad at times 1, 2 and 3 as a whole to develop themes. - *Cross-case analysis (cross-dyad analysis)* - *Themes developed for each dyad noted on paper with quotes and mapped onto pin board to search for patterns (convergence and divergence) across accounts to identify themes across dyads and create overall synthesis.* | Analysis at dyadic level and then across dyads | Transcripts analyzed from each dyad as a whole |
| Boulicault, 2023  *Cite:*  *Eisikovits & Koren, 2010*  *Manning & Kunkel, 2015* | Dyadic thematic analysis  Software: ATLAS.ti | - Each interview transcript coded in two different ways:   - First, as an independent interview, and   - Second, alongside the transcript of their family member, with the aim of identifying overlaps and differences between their perspectives and experiences. - In many instances, the use of a dyadic approach altered understanding of the meaning and significance of quotes from individual transcripts, leading to code updates. This process continued, going back and forth between updating codes at the individual and dyadic levels of analysis, until a set of key themes were determined. | Moving back and forth between individual, dyadic +/- aggregate or cross-dyad levels of analysis | Side-by-side readings to compare and categorize themes, codes, or **accounts** within each dyad |
| Boyle, 2013a | Thematic analysis with dialogical approach  Software: NR | - Key themes in each couple’s data were identified, informed partly by theoretical concepts but also open-ended to allow new concepts to emerge. The types of ﬁnancial decisions made were identiﬁed and the management and decisional processes used before and after the dementia were compared. - *Comparative analysis of the data from all the couples then undertaken to identify whether the ﬁnancial management and decision-making processes used and any support from spouses varied depending on social factors, such as gender.* A dialogical approach to data analysis allowed for inherent tensions within the explanatory process to be captured. | Analysis at dyadic level and then across dyads | Transcripts analyzed from each dyad as a whole |
| Boyle, 2013b | Thematic analysis  Software: NR | - For first level of coding, key themes in each couple’s data were identified, informed partly by theoretical concepts but also open-ended to allow new concepts to emerge. The carer-spouses’ perceptions of their partners’ decisional and practical abilities, their rationales and motivations for including or excluding them from decisions about housework and the degree of control that women with dementia exerted over this issue were explored. - *For second level of coding, comparative analysis of data from all couples to identify whether the support provided by the spouses varied depending on social factors (gender).* | Analysis at dyadic level and then across dyads | Transcripts analyzed from each dyad as a whole |
| Boyle, 2013c | Thematic analysis  Software: NR | - For first level of coding, key themes in each couple’s data were identified, informed partly by theoretical concepts but also open-ended to allow new concepts to emerge. The types of support given by the carer-spouses and its effects on the capacity of people with dementia were also examined. - *For second level of coding, comparative analysis of the data from all the couples was undertaken to identify whether the decision-making support provided by the carer spouses varied depending on social factors (such as gender).* | Analysis at dyadic level and then across dyads | Transcripts analyzed from each dyad as a whole |
| Boyle, 2014a | Thematic analysis  Software: NR | - For first level of coding, key themes in each couple’s data were identified, informed partly by theoretical concepts but also open-ended to allow new concepts to emerge. The couples’ previous and current decision-making styles were compared (that is, the approaches used before and after the onset of dementia). The carer-spouses’ perceptions of their partners’ decisional and practical abilities; their rationales and motivations for including or excluding them from decisions about cooking and the degree of control which women with dementia exerted over this issue were explored. - *For second level of coding, comparative analysis of the data from all the couples undertaken to identify whether the decisions about cooking and the related decision-making processes were inﬂuenced by social factors (notably gender).* | Analysis at dyadic level and then across dyads | Transcripts analyzed from each dyad as a whole |
| Boyle, 2014b | Thematic analysis  Software: NR | - Key themes in each couple’s data were identified, informed partly by theoretical concepts but also open-ended to allow new concepts to emerge. The levels and types of support given by the carer-spouses and its effects on the decisional capacity and agency of people with dementia were considered. The analysis primarily focused on the subgroup with more advanced dementia. | Analysis at dyadic level only | Transcripts analyzed from each dyad as a whole |
| Boyle, 2017 | Thematic analysis  Textual analysis  Software: NR | - Analysis focused primarily on the data obtained directly from the people with dementia themselves. The spouses’ knowledge of their partners’ personalities, habituated and changing routines and patterns of communication informed the researchers’ interpretations. However, in order to respect the equality of each partner and the trust accorded to us by the people with dementia, their spouses’ accounts were not used to ‘test’ the veracity of their standpoint. Instead, it was recognised that each partner had their own perspective, which often corresponded with the couple perspective, but might also diverge, and which could be influenced by the relationship to the illness (whether lived or observed). | Individual analysis of data from 1 dyad member with partners’ accounts used to inform analysis and interpretation | Side-by-side readings to compare and categorize themes, codes, or **accounts** within each dyad |
| Brooks, 2014  *Cite:*  *Eisikovits & Koren, 2010* | IPA  Software: NR | Transcripts were analysed using IPA:   - A master list of themes was produced for each transcript, which were examined together to identify themes operating across cases. A key overarching category emerged around experiences of social interactions. - Interview transcripts were considered as dyadic units, focusing on relational aspects of the accounts, in particular how they might be similar, where they might diﬀer, and how they may interact. Dyadic analysis led to reconstruction of theme (social interactions) from individual analysis. - The third dyadic version presented is one in which the investigators have played a key and openly acknowledged interpretative role. | Dyadic analysis to further explore one or two relational themes or categories from analysis at individual level | Side-by-side readings to compare and categorize themes, codes, or **accounts** within each dyad |
| Buck, 2013  *Cite:*  *Ayres et al., 2003* | Case analysis  Content analysis  Software: NR | - Dyadic type explored based on patient and caregiver’s description of the patient’s heart failure care in the interview:   - Both patient and caregiver asked to indicate who provided the majority of the patient’s care and were then invited to describe what they did. Interviews analyzed and then discussed by the investigators and an exemplar case from each type that best described both the unique and similar experiences of each dyadic type was chosen. To be considered for selection as a case of the type, dyad had to agree on their type. A fifth case, called incongruent and defined as a case where the patient and caregiver indicated different or incongruent dyadic types, was included to highlight that not all dyads, however, agree on their dyadic type and characteristics of this incongruent type discussed. - Content analysis on full sample: - After the cases were identified and analyzed for the typology, a content analysis was conducted on the narratives of the full sample to provide a deeper understanding of dyadic heart failure care from all of the dyads. Initially, key themes were identified by with-in case analysis then *examined across-cases in an iterative process.* | Analysis at dyadic level only | Transcripts analyzed from each dyad as a whole |
| Chan, 2017 | Procedures of grounded theory  Software: NR | - Coding of individual interviews and grouping of codes to form components of spirituality. - Constant comparisons of the components and their content across and within dyads. Similarities and differences in the understanding of spirituality among dyads were identified. - Exploration of family dynamics caused by the differences in the understanding of spirituality among a dyad. Patients’ and caregivers’ reactions induced by the differences were coded. In each group, similar reactions were grouped together. *Related reactions among dyads were linked together, forming a pattern of interaction caused by the differences in spirituality.* - In each dyad, influences of the family dynamics, as well as the dyads’ coping strategy, on patient’s psychological well-being were coded and categorised. | Analysis at individual level, then at dyadic and aggregate (group) levels based on results from analysis at individual level (and across dyads) | Side-by-side readings to compare and categorize themes, **codes**, or accounts within each dyad |
| Catona, 2022 | Inductive coding with analytic principles from grounded theory  Software: NR | - Inductive or flexible coding, including open and axial coding, to identify specific examples of caregiving-related challenges and/or consequences that emerged during analysis of the patient and caregiver interview transcripts. - Responses were coded as agreement when patient and caregiver pairs mentioned the same challenge and/or consequence. If a caregiving-related challenge and/or consequence was mentioned by only the patient or only the caregiver that was coded as disagreement. - *Proportion of agreeing and disagreeing dyads calculated.* | Analysis at individual level, then at dyadic level informed by results from analysis at individual level, then across dyads | Side-by-side readings to compare and categorize themes, codes, or **accounts** within each dyad |
| Chen, 2015 | Phenomenological analysis  Software: NR | - Identified signiﬁcant statements, themes, and patterns by identifying signiﬁcant statements pertaining directly to participants’ experiences of deciding to institutionalize. Meanings were formulated from these signiﬁcant statements, through which common themes regarding the decision-making process emerged. - Themes clustered to identify the basic decision-making pattern. After identifying the pattern, original transcripts reread to ensure that no main themes remained unidentiﬁed. - Guided by the dyadic perspective, comparison of intradyad characteristics to highlight how they strived to achieve positive social identity in intergenerational communication. - *Comparison of interdyad experiences throughout the 12 families.* | Analysis at individual level, then at dyadic level, then across dyads | Side-by-side readings to compare and categorize themes, codes, or **accounts** within each dyad |
| Collaço, 2019  Collaço, 2021a  Details regarding methods also extracted from Collaço, 2021b.  *Cite:*  *Eisikovits & Koren, 2010*  *Yosha et al., 2011* | Thematic analysis using the Framework Method  Dyadic analysis drawing upon multiperspective analysis methodology  Software: Microsoft Word, Microsoft Excel | - Each transcript was coded for the man with prostate cancer (PCa) and his partner separately. A table of general themes were created based on the questions asked and the codes from the participants’ transcripts. - Creation of a table of patient and partner quotes which was adapted for the Framework Method through creating tables consisting of codes that were developed to create themes and subthemes relevant to the man with prostate cancer and his partner (each participant was assigned a column and each subtheme assigned a row; a 3rd column assigned for dyadic code/summary created). - Dyadic summaries created based on initial codes and allowed analysis at the level of the couple (3rd column). Dyadic codes/summaries were created based on the themes and subthemes for the individual couple by exploring the extent of agreement between members of the dyad, and how each theme affected one another and possibly changed the experience depending on how each couple addressed a particular problem. Further codes were developed from the dyadic analysis which reflected the couples’ experiences and needs rather than individual experiences. Dyadic summaries were placed into framework matrices relevant to the themes. - The analytical framework was created after six tables of dyadic analyses had been developed from the couple transcripts. Having this coding framework allowed for the analytical framework to be tested on other dyads (table of themes consisting of dyadic codes/summaries) and therefore development of further codes as required. The initial themes in the coding framework aided in the development of three overarching themes. With the research question in mind, the overarching theme was further developed. - The working analytical framework was applied by indexing subsequent dyadic analyses (a word document consisting of partner and man with prostate cancer summaries) using existing categories and codes. - *The variation in experiences of couples was explored based on specific factors of interest and using dyadic theory to guide interpretation and analysis. Theoretical frameworks of adjustment and identifying overlaps and contrasts in dyadic narratives guided the analysis and interpretation further.* - The last step involved interpreting the data through exploring potential overlaps and contrasts between the interviews of each partner (informed by Eisikovits and Koren, 2010). | Analysis at individual level, then at dyadic level informed by results from analysis at individual level, then across dyads | Creation of matrices or summary tables to visually examine and compare **codes,** themes, concepts, or accounts within each dyad |
| Conroy, 2017 | Techniques of grounded theory and data matrices  Software: Dedoose | - Reviewed each set of couple transcripts for content and created memos describing couple dynamics and relationship-level influences on adherence. - Coded the all transcripts using open and axial coding. - Data matrices were created to visually examine themes within and across categories. When corresponding content was available from both partners, both partners’ information for each theme were presented and any discrepancies and what could be inferred was noted. | Analysis at individual level, then at dyadic level informed by results from analysis at individual level, then across dyads | Creation of matrices or summary tables to visually examine and compare codes, **themes,** concepts, or accounts within each dyad |
| Conroy, 2018 | Innovative analysis approach developed by authors to analyze data at the couple level  Software: NR | - Created couple summary tables with a row for each topic in the interview guide and two columns for what “she said” and “he said”. Authors then re-reviewed the couple's transcripts, and within each cell of the table, wrote a summary of what participants communicated about each topic. At the top of each couple summary, included a brief narrative highlighting perceptions of the couple's relationship quality, areas of consistency and discrepancy between partners, and major topics discussed. Discrepancies between partners were handled on a case-by-case basis by examining surrounding context of each partner's interviews and asking questions such as, “what was the level of detail provided?” “which partner could be more prone to social-desirability bias based on the content?” “is the partner's information consistent with other aspects of the interview?” - *Created data matrix with a row for each couple to summarize key factors aﬀecting dyadic coordination and adherence, and to identify patterns of factors across couples.* Included in this matrix was also a column with a brief description of the predominant pathway(s) between relationship factors, and dyadic coordination and adherence with supporting quotes from both partners. - *Identiﬁed similarities and diﬀerences between couples using the summaries, data matrices, and raw data, and common themes aﬀecting adherence.* After identifying couples who exempliﬁed these *themes, authors re-examined the raw data for sub-categories of patterns and couples categorized into three patterns of inﬁdelity and dyadic coordination.* | Analysis at dyadic level and then across dyads | Creation of matrices or summary tables to visually examine and compare codes, themes, concepts, or **accounts** within each dyad |
| Conroy, 2019 | Dyadic analysis approach based on framework analysis  Software: NR | - Created couple summary tables with a row for each topic in the interview guide and 2 columns indicating what "she said" and "he said" with a brief narrative highlighting each partner's reports on the couple's relationship, areas of consistency and discrepancy between partners, and major topics discussed. In comparatively analyzing couples’ accounts, noted areas in which partners had incongruent accounts and assessed these discrepancies by examining the level of detail provided, other issues in the relationship that might provide clues, whether the information provided was consistent with other segments of the interview, and whether social desirability bias might influence the reporting on certain behaviors. - *Created a data matrix with a row for each couple to summarize key factors affecting adherence (e.g., alcohol use, violence, food insecurity) with supporting quotes, and to identify patterns of factors across couples.* - *Discussed similarities and differences between couples using the couple summaries, data matrices, and raw data, and to identify common themes using the couple summaries, data matrices, and raw data, and to identify common themes affecting adherence.* | Analysis at dyadic level and then across dyads | Creation of matrices or summary tables to visually examine and compare codes, themes, concepts, or **accounts** within each dyad |
| Conroy, 2020  *Cite:*  *Eisikovits & Koren, 2010* | Framework analysis  Software: NVivo | - Identified a thematic framework based on the interview guide topics and issues raised by the couples themselves. - “Indexed” or coded the raw data and then “charted” the data simultaneously by creating a matrix for each couple, with a row for each of the codes and a column for the responses of each member of the couple. These matrices were populated with summaries of participants’ stories and verbatim quotes related to the codes. - *Created a summary matrix for the entire sample with a row for each couple, which noted areas of inconsistency and disagreement between partners on the codes.* - *Couple-specific matrices coded to facilitate comparisons across couples (e.g., different ways in which alcohol affected couples), between partners (e.g., discrepancies in descriptions of alcohol use patterns), and across individuals (e.g., intervention preferences).* - *Discussion of similarities and differences between couples using the data matrices and raw data to identify underlying themes.* | Analysis at dyadic level and then across dyads | Creation of matrices or summary tables to visually examine and compare **codes**, themes, concepts, or accounts within each dyad |
| Constant, 2022  *Cite:*  *Larkin et al., 2019* | IPA with multiperspective design  Software: NR | - Put into perspective each partner’s experience regarding their couple life by focusing on the divergences and convergences between each partner in their interview. The analyses were conducted through the diﬀerent steps recommended in IPA analysis. - First, verbatim transcripts were read several times for familiarization with the data. - Annotated and analyzed interview transcriptions through an idiographic conceptualization exploring the experience of each partner by focusing on the dyadic processes. - Then, put together into perspective the lived experience of both partners, and highlighted the salient dyadic process of the couple dynamic. The analyses were performed on a couple-by-couple basis, i.e., when a couple was analyzed, the lived experience and the underlying processes that emerged were assessed in conjunction with the previous cases. After each pair of analyses, summary created. - *At the end of the couple analyses, attempted to identify salient elements to achieve a synthesis of the couple experience and the associated dyadic processes. Hence, this process allowed the identiﬁcation of the typologies of the dyadic functioning underlying the couple’s lived experience while also taking into account the couple functioning prior to the disease.* | Analysis at individual level, then at dyadic level informed by results from analysis at individual level, then across dyads | Written dyad summaries |
| DeGroot, 2021  *Cite:*  *Boeije, 2002*  *Eisikovits & Koren, 2010* | Thematic analysis  Software: F4analyse | - Initial coding of individual transcripts. - Comparison of the patient group with the caregiver group to see whether the two shared a perspective overall. - Each individual dyad compared to examine within-dyad congruence regarding a topic. Congruence evaluated for each major theme. Dyadic congruence was defined as a shared understanding or aligned perspectives regarding a particular topic between the patient and the caregiver. | Analysis at individual level, then at dyadic and aggregate (group) levels based on results from analysis at individual level | Side-by-side readings to compare and categorize themes, **codes,** or accounts within each dyad |
| Demirtepe-Saygili, 2022  *Cite:*  *Eisikovits & Koren, 2010* | Content analysis  Dyadic qualitative analysis  Software: NR | - Content analysis conducted at an individual level and themes identified. - Themes from the couple’s scripts were compared. In this way, the answers were evaluated both as an individual and as a part of a couple. The contrasts and overlaps were taken into consideration. The themes were then categorized as convergent and divergent based on their similarities and differences. The codings which could not be categorized into either category were treated as independent themes referring to different issues on different topics provided by either partner. The independent themes were not evaluated further, as it was beyond the scope of the study. | Analysis at individual level, then at dyadic level informed by results from analysis at individual level | Side-by-side readings to compare and categorize **themes,** codes, or accounts within each dyad |
| Dobrina, 2016 | Phenomenological orientation applied to transcript pairs  Software: NR | - Transcripts read in pairs and emerging themes categorized with themes emerging from other interviews also read in pairs. | Analysis at dyadic level only | Side-by-side readings to compare and categorize **themes,** codes, or accounts within each dyad |
| Ekelund, 2010 | Qualitative and content-oriented terms using biographical analysis and discursive approach  Software: NR | - Initial interview of each patient ﬁrst read to identify and develop special response units or theme. - The transcripts for each patient were then reread, with a biographical perspective being explored. - The analysis was extended by including transcripts of the partner, these likewise being read thoroughly to identify possible differences between the patient and partner in the information obtained. Consideration of the information the partner provided also involved biographical analysis and made it possible to explore, through use of a discursive approach, the evolution over time of meaning and understanding on the part of the patient-and-partner dyad. | Individual analysis of data from 1 dyad member with partners’ accounts used to inform analysis and interpretation | Side-by-side readings to compare and categorize themes, codes, or **accounts** within each dyad |
| Ekstam, 2011 | Constant comparative method  Software: NR | - The interviews were coded incident by incident. - Each incident was then compared with 1) previous incidents in the same interview 2) and across all the interviews with the same participant 3) as well as his or her spouse’s interviews over time. | Analysis at individual level, then at dyadic level informed by results from analysis at individual level | Side-by-side readings to compare and categorize themes, **codes**, or accounts within each dyad |
| Engeli, 2016 | Qualitative content analysis  Software: NR | - Responses designated to 9 different code categories (subcategories) - Codes were allocated to 3 response categories in accordance with the 3 categories described by Antonovsky (comprehensibility, management/manageability and meaning/ meaningfulness) - Differences between patients’ and spouses’ responses and between the responses at the two data collection points (t1 and t2) were detected using paired t-tests, with P = 0.05 set as the threshold for statistical signiﬁcance and all tests two-tailed. | Analysis at individual level, then at dyadic level informed by results from analysis at individual level | Quantizing: frequency counts and statistical comparison |
| Eriksson, 2010 | Qualitative content analysis  Software: NR | - Manifest level:   - Meaning units relevant to the aim were identiﬁed from each interview. The meaning units were condensed, maintained close to the content and labelled with a code.   - The codes were compared and sorted into subcategories. A comparison was made of the subcategories in terms of differences and similarities. - Latent level:   - Considered each couple’s interviews as a whole. Read each interview separately with the intention of ﬁnding meaning units. The meaning units were highlighted and transferred to a sheet of paper divided into three columns. Those pertaining to the patient were placed in the left-hand column and their partners’ meaning units in the right-hand column. The statements were condensed and ﬁnally transformed into a short story about each couple in the middle column. This second phase of the analysis was performed at a more interpretative level.   - *When all of the couples’ short stories had been condensed into a whole, it was possible to interpret and group them into four positions.* | Analysis at individual level, then at dyadic level, then across dyads | Creation of matrices or summary tables to visually examine and compare **codes**, themes, concepts, or accounts within each dyad |
| Farquhar, 2017 | Framework analysis  Software: software facilitating data management | - Patients’ and carers’ transcripts were analysed separately (examining views within roles), in dyads (establishing differences/ comparability in preferences as these may impact on intervention development), and by disease group. This was an iterative process that began after the first two patient-carer dyad interviews were conducted. | Moving back and forth between individual, dyadic +/- aggregate or cross-dyad levels of analysis | Side-by-side readings to compare and categorize themes, codes, or **accounts** within each dyad |
| Ferreira, 2020  *Cite:*  *Boeije, 2002* | Constant comparative approach guided by grounded theory principles  Software: NVivo | - Open coding of the interview transcripts. - Codes were then grouped into themes; each theme was illustrated with several quotes to confirm coding validity. - Concepts emerging from the patients’ and carers’ data were further examined for any major differences between the two viewpoints. A matrix summarised the findings of patients and their carers. | Analysis at individual level, then at dyadic level informed by results from analysis at individual level | Creation of matrices or summary tables to visually examine and compare codes, themes, **concepts**, or accounts within each dyad |
| Fonner, 2021  *Cite:*  *Boeije, 2002* | Constant comparative method  Software: ATLAS.ti | - Data were coded using a coding structure informed by salient concepts derived from the initial analysis. [Unclear what "initial analysis" refers to.] - Concepts from the constant comparative method were used to compare within interviews, across subgroups (e.g., comparing males versus females, HIV-positive versus HIV-negative, participants versus nonparticipants, etc.) and across individuals within a couple, if both members of the couple were interviewed. | Analysis at individual level then at dyadic and aggregate (group) levels | Side-by-side readings to compare and categorize themes, **codes**, or accounts within each dyad |
| Gamarel, 2016 | Framework analysis  Software: Microsoft Excel | - Transcribed data were organized in a spreadsheet with one row for each interview question and responses arranged by participant ID number in columns. This method of organizing the data permitted the authors to easily compare study partners’ data, as their responses to questions were presented in adjoining columns. - Identified themes that emerged around partners’ involvement in each other’s health, and examined how these views were articulated within partnerships. | Analysis at dyadic level only | Creation of matrices or summary tables to visually examine and compare codes, themes, concepts, or **accounts** within each dyad |
| Goldsmith, 2016 | 2 phases of coding guided by grounded theory  Software: NR | - Phase 1:   - Coding of individual interviews for conversation pattern guided by conceptual framework of Family Communication Pattern Theory.   - To ascertain whether or not patient-caregiver dyads shared the same communication pattern, patients and caregivers were compared for pattern congruency. - Phase 2:   - *Family communication pattern used to identify specific caregiver type and transcripts grouped together* and thematically analyzed using four distinct grounded theory phases (open coding to identify unrestricted chunks of texts that suggested a theme, integration in which previously identiﬁed themes were connected, collapsed, or associated into thematic categories through the process of constant comparison by combining and organizing subcategories into a smaller number of categories, reviewing the transcripts again, and themes developed and reﬁned, construction of interpretive claims about the categories identiﬁed through discussion and conﬁrming data saturation). | Analysis at individual level, then at dyadic level informed by results from analysis at individual level, then across dyads | Side-by-side readings to compare and categorize themes, **codes**, or accounts within each dyad |
| Gorman, 2020  *Cite:*  *Thompson & Walker, 1982* | Thematic analysis with dyads as the primary unit of analysis  Software: NVivo | - Coded individual responses of survivors and partners using thematic analysis, where codes emerge from the raw data, to identify themes, sub-themes, and patterns in the data. - After coding the separate interviews, developed dyad-level codes to facilitate analysis within and across couple responses. - After examining individual and dyadic data across each interview topic, explored how themes mapped on to the theory of dyadic illness management to build a conceptual model specific to sexual health after cancer. | Analysis at individual level and then at dyadic level | Coded within couples or dyadic-level codebook |
| Grivel, 2023 | Thematic analysis  Software: NVivo | - Coded data and identified themes for individuals. - Each dyad compared one by one, to detect possible differences or similarities within the dyad. | Analysis at individual level and then at dyadic level | Side-by-side readings to compare and categorize themes, codes, or **accounts** within each dyad |
| Hedman, 2019 | Qualitative content analysis  Software: NR | - The parts in which a participant spoke about the person with dementia were then highlighted in the text and extracted. - The extracted texts were divided into meaning units, condensed, and coded for agency and communion in the past (before the debut of Alzheimer’s disease), the present, and the future. - To be able to compare the views of the spouses of each couple, a matrix was constructed. For each person, a short summary of the content concerning each area (agency and communion in the past, present, and future) was made and put in the matrix along with the interpreted meaning (in bold) of the content. | Analysis at individual level, then at dyadic level informed by results from analysis at individual level | Creation of matrices or summary tables to visually examine and compare codes, themes, concepts, or **accounts** within each dyad |
| Heid, 2016 | Content analysis  Software: NVivo | Each interview was coded independently (manifest content) with the developed themes regarding how parents inﬂuence their care, how daughters respond to such efforts of inﬂuence, and how dyads navigate differences in care goals, and then compared to his/her family member’s responses to examine dyadic responses in daily life. | Analysis at individual level, then at dyadic level informed by results from analysis at individual level | Side-by-side readings to compare and categorize **themes,** codes, or accounts within each dyad |
| Hellström, 2013 | Initial coding  Software: None | - First step of analysis entailed the systematization of the data in order to reach a preliminary understanding of what the informants expressed. - Coded each of the interviews on the basis of the variety of topics identified as relevant for the informants’ disclosure preferences. - Once the interviews were coded, proceeded to identify how topics were interrelated with one another for each of the informants, as well as for each of the couples. *This entailed looking for the overarching themes that these various topics had in common for the couples.* Table 2 can be regarded as the results of this second step of the analysis [Table 2 shows types of disclosure patterns found in data with row for each dyad and column for each of 5 types of disclosure pattern.] | Analysis at individual level, then at dyadic level informed by results from analysis at individual level, then across dyads | Side-by-side readings to compare and categorize **themes,** codes, or accounts within each dyad |
| Hellström, 2016 | Initial coding  Software: NR | - Identiﬁcation of the topics that were discussed when understandings of the future were verbalized. - Systematization of the topics identiﬁed per interview to reach a preliminary understanding of what each and every one of the informants expressed with regard to the future focussing on whether or not there were discrepancies between the understandings that were voiced by the persons with dementia and those expressed by their spouses. - Coded each of the interviews on the basis of the variety of topics that were deemed to be relevant for the study. - Looked for the overarching themes that various topics shared in common per couple. Table 3 is based on the results of this step of the analysis. [Table 3 shows patterns of understandings of the future with informants in rows and patterns of understandings as columns.] - Focused on the speciﬁc time-related expressions noted to be important when understandings of the future were being formulated. *Explored speciﬁc patterns that emerged between the diﬀerent understandings of the future that were revealed and the number of expressions (such as ‘‘not yet’’) that were used by both the persons with dementia and their spouses and the sample characteristics.* | Analysis at individual level, then at dyadic level informed by results from analysis at individual level, then across dyads | Creation of matrices or summary tables to visually examine and compare codes, **themes**, concepts, or **accounts** within each dyad |
| Hendryckx, 2024  *Cite:*  *Eisikovits & Koren, 2010* | Inductive qualitative analysis method  Software: Microsoft Word | - Coded all interviews with a list of codes that was inductively expanded throughout the analysis process and then themes and subthemes created. - Role-ordered matrices used to organize information and describe relationship dynamics according to participant roles and to compare varied experience. A ﬁrst role matrix was created, where rows corresponded to the sub-themes (from individual analysis), and columns corresponded to the points of view of people with traumatic brain injury (TBI) and caregivers. This allowed to identify the overlaps and discrepancies between the perceptions of caregivers and people with TBI more generally. - *Then created a second, ﬁner role-ordered matrix, where columns corresponded to the points of view of the two members of each dyad, to perform a dyadic analysis.* | Analysis at individual level, then at dyadic level informed by results from analysis at individual level, then across dyads | Creation of matrices or summary tables to visually examine and compare codes, **themes**, concepts, or accounts within each dyad |
| Hopkinson, 2016 | IPA  Software: NR | - Explored experience of each partner (interpretive phenomenological approach. - Shared and competing understandings from the ﬁrst stage of the analysis (analyzed using an interpretive phenomenological approach) were used to generate a summary description of interactions within each dyad, thereby locating the nature of interdependency in response to involuntary weight loss and eating problems. - *Thematic cross-case analysis to identify patterns in dyadic response to construct a conceptual model of dyadic experience.* | Analysis at individual level, then at dyadic level informed by results from analysis at individual level, then across dyads | Written dyad summaries |
| Huang, 2021  *Cite:*  *Eisikovits & Koren, 2010* | Qualitative dyadic approach  Software: Microsoft Excel | - Analyzed the data in the individual and dyadic levels using an inductive approach [but used framework]. - Compared and contrasted each individual’s experience and needs of family support that of the caregivers by using the qualitative dyadic analysis approach to discover overlaps between individual narratives and identify points of collaboration and contradiction between dyads, which formed the “unit of analysis.” - Cross-analyzed significant statements, sentences, and quotes, to formulate themes based on both dyad members’ perspectives on their supportive care received/provided. Comparing dyad perspectives allowed triangulation to derive a fuller, more contextualized understanding of the nature of family support. | Sequence not specified | Side-by-side readings to compare and categorize themes, codes, or **accounts** within each dyad |
| Hudson, 2016  Details regarding methods also extracted from Hudson, 2020  *Cite:*  *Eisikovits & Koren, 2010* | Thematic analysis followed by dyadic analysis  Software: NVivo | - Thematic analysis with development of coding framework. - Descriptive themes particularly signiﬁcant at the couple and relationship level were subject to a dyadic analysis, taking each couple as a unit of analysis and identifying overlaps and contrasts in partners’ accounts. - *These instances were compared across couple units in order to identify patterns across the data.* | Dyadic analysis to further explore one or two relational themes or categories from analysis at individual level, then across cases | Side-by-side readings to compare and categorize **themes,** codes, or accounts within each dyad |
| Iannarino, 2022  *Cite:*  *Eisikovits & Koren, 2010* | Dyadic analysis, thematic analysis  Software: NR | - Read each survivor participant’s responses sequentially with their partner’s to search for thematic overlaps and contrasts between each dyadic member’s individual experiences. - Open coding to identify and label any components, ranging from a few words to several paragraphs, that related to dyads’ conversations about fertility preservation decisions across entire transcripts. - Focused coding, in which one codebook created to code all data according to a priori concepts while also allowing new insights to emerge, which included how and why participants clustered into certain themes. | Analysis at dyadic level only | Side-by-side readings to compare and categorize themes, codes, or **accounts** within each dyad |
| Jere, 2023 | Individual-level and dyadic-level analyses using a framework analysis approach  Software: NR | - Conducted an individual-level analysis and dyadic-level analysis:   - Read and reviewed interview transcript pairs, created summary tables, and organized the data by couples and by larger categories from literature and interview guide. - Compared the responses within *and between* couples by including raw quotes from each partner and then summarizing all the participants’ responses by category and noting any agreements or disagreements between partners. | Sequence not specified | Creation of matrices or summary tables to visually examine and compare codes, themes, concepts, or **accounts** within each dyad |
| Kano, 2022 | Thematic analysis at individual and dyadic levels  Software: Dedoose | - Developed a codebook and then iterative analysis comparing and contrasting codes, grouping similar content or meaning into broader themes, describing linkages, at individual levels, dyadic levels, *and cross-dyadic* (sexual gender minority and heterosexual, cisgender) levels. | Moving back and forth between individual, dyadic +/- aggregate or cross-dyad levels of analysis | Side-by-side readings to compare and categorize themes, codes, or **accounts** within each dyad |
| Kitko, 2015 | Thematic analysis to identify incongruent dyads  Software: HyperResearch | - Read interviews to determine if incongruence between the dyad was present. Incongruence was established when the patient and caregiver expressed differing views on living with heart failure. - Interviews with incongruent dyads were further analyzed by reading transcripts independently and then as a dyad resulting in the identification of tentative areas of incongruence and the impact on the patient, caregiver, and heart failure experience. | Analysis at individual level, then at dyadic level informed by results from analysis at individual level | Side-by-side readings to compare and categorize themes, codes, or **accounts** within each dyad |
| Lamore, 2019 | IPA  Software: NR | - Initial coding and organization of codes into themes for each interview then across interviews. Interviews from women and men analyzed separately, and then themes analyzed for each couple. - Furthermore, writing IPA results is divided into two main sections: (1) a detailed presentation of the superordinate themes that emerged from the data and (2) a presentation of the interpretative account. The interpretative account reflects the principle of double hermeneutics. | Analysis at individual level, then at dyadic and aggregate (group) levels based on results from analysis at individual level | Side-by-side readings to compare and categorize **themes**, codes, or accounts within each dyad |
| Loaring, 2015 | IPA  Software: NR | - Open and free coding to identify both initial areas of interest and possible preconceptions (i.e., incorporating reflexive commentary). Line-by-line analysis (i.e., coding) of the experiential claims, concerns, and understandings of each participant. - Identification of the emergent patterns (i.e., themes) within experiential material emphasizing both convergence and divergence, commonality, and nuance first for single cases, then subsequently with pairs of scripts (couples), *then patterns across the individuals and couples.* - Development of a “dialogue” between the researchers, their coded data, and their psychological knowledge, about what it might mean for participants to have these concerns, in this context, leading in turn to the development of a more interpretative account. - Development of a structure, frame, or gestalt that illustrates the relationships between themes. - Organization of all of this material in a format that allows for coded data to be traced right through the analysis—from initial codes on the transcript, through initial clustering and thematic development, into the final structure of themes. - Use of supervision or collaboration, to audit, to help test, and to develop the coherence and plausibility of the interpretation and explore reflexivity. - Development of a narrative, evidenced by detailed commentary on data extracts, which takes the reader through this interpretation, usually theme-by-theme, and often supported by some form of visual guide (simple heuristic, diagram, or table). | Analysis at individual level, then at dyadic level informed by results from analysis at individual level, then across dyads | Side-by-side readings to compare and categorize **themes**, codes, or accounts within each dyad |
| Loup, 2023 | Rapid qualitative analysis (using thematic and content analysis) followed by life-space map visualizations  Software: Microsoft Excel | - First, rapid qualitative analysis to generate daily-life needs of the participants’ home and community context. This included coding participant responses that referenced needs and labeling if the needs therein were “met” or “unmet” using a rapid template design then evaluating them with both thematic and content analysis to iteratively create a codebook categorizing participants’ daily life activity and resource needs. - Summative content analysis was then employed to numerically tally each interview’s identiﬁed need codes. The resulting code counts provided ordered sets of needs for each dyad and individual relevant to the entire sample and other participants. - Next, life-space maps were developed to synthesize and visualize dyads’ met and unmet needs. While interview data from all participants were used to develop a thematic needs based codebook, life-space maps were only created for the dyadically participating individuals to demonstrate life-space mapping’s ability to visualize the combined context of a person with dementia and their caregiver in the home. | Analysis at individual level, then at dyadic level informed by results from analysis at individual level | Visual mapping of dyadic data |
| Manceau, 2023  *Cite:*  *Larkin et al., 2019* | IPA with dyadic multiperspectival analysis  Software: NR | - Analysis of experiential concerns and understanding of each partner and creation of individual schema summarizing processes. - Analysis of dyadic processes and schematized them by mirroring each individual analysis and identification of dyadic processes to better observe convergences and divergences between experiences. - Synthesis of salient processes to identify different patterns of dyadic experience. - To refine their analyses and nourish the reflective process, they then discussed their interpretations, shared their perceptions and feelings about the interviews and the analysis of each case (couple). Each schema was presented to the supervisor of the study, whose supervision allowed better coherence in the interpretation. This process was performed for each case (couple), one at a time. | Analysis at individual level, then at dyadic level informed by results from analysis at individual level | Side-by-side readings to compare and categorize **themes**, codes, or accounts within each dyad |
| Martin-Matthews, 2022 | Thematic analysis  Software: NVivo | - Topic of caregiving dynamics emerged through individual analysis. Interviews were fully (re)read and then coded based on the topics mentioned by the interviewees at different points of the conversation regarding caregiving dynamics. - Analysed all matched pairs of dyad interviews and identified themes that addressed the relational aspects of giving and receiving different types of care, and the coordination of tasks between client and carer. | Dyadic analysis to further explore one or two relational themes or categories from analysis at individual level | Side-by-side readings to compare and categorize **themes,** codes, or accounts within each dyad |
| McCarthy, 2015 | Interpretive description techniques  Software: NR | - Qualitative data were analyzed using interpretive description techniques including data emersion and synthesis of participant (i.e. couples) experiences across cases. Transcripts were analyzed at the dyad level, with matching survivor and spouse transcripts being treated as one unit. Each set of two transcripts (i.e. one survivor, one spouse) reviewed as a single unit to assess the degree to which partners expressed similar or dissimilar appraisals of the survivor’s functioning (i.e. incongruence) and the extent to which any differences manifested in emotional distress for spouses. | Analysis at dyadic level only | Side-by-side readings to compare and categorize themes, codes, or **accounts** within each dyad |
| McCarthy, 2020 | Constant comparison strategies through the lens of interpretive description  Software: Microsoft Word | - Reviewed a portion of the transcripts (3 sets of transcripts from spousal dyads and 3 sets of transcripts from adult child-parent dyads) to extract a preliminary set of themes and subthemes (where applicable) that cut across cases. - Completed detailed tables, which had columns for dyad type (spousal vs. adult child-parent), respondent (survivor vs. caregiver), 3-5 word description of theme, illustrative quote if applicable, and general reflections. - *Synthesized this initial pass at the data into two separate summary documents (one for spousal dyads, one for adult child-parent dyads) which were used to guide discussions about the suitability of the themes and subthemes, how they were labeled, whether they were too broad or too narrow, and whether they applied to one or both dyad types. Although differences were found between spousal and adult child-parent dyads, ultimately decided that the experiences of study participants with respect to the impact of stroke on the relationship were more similar than different.* - Codebook was developed that could be used to represent the experiences of both dyad types. The codebook was used to analyze the entirety of the transcripts. - Summary case reports were used throughout the analysis and write-up to refresh the researchers’ memories of the interviews and to contextualize understanding. | Analysis at dyadic level and then across dyads | Creation of matrices or summary tables to visually examine and compare codes, **themes**, concepts, or accounts within each dyad |
| McCarthy, 2022 | Case analysis informed by interpretive description  Software: NR | - Interview summary reports were written by each interviewer based upon field notes, for each dyad, immediately after the interview. - Case analysis tables created with columns for each dyad (survivor and caregiver) and case elements including demographic, clinical, and psychosocial characteristics, environment within and outside of the home, interview context, and other key issues or questions that surfaced). - Informed by an interpretive description approach, the case analysis involved a cyclical and iterative process to develop in-depth knowledge of each participant within each dyad, as well as to examine the cases as a whole. - Cases were composed based upon the “case and frame” method proceeding to “frame” the cases within existing research about social determinants of health in order to draw out potential lessons to inform social work practice and policy. | Analysis at dyadic level only | Creation of matrices or summary tables to visually examine and compare codes, themes, concepts, or **accounts** within each dyad |
| Miller, 2013 | Constant comparative methods  Software: NR | - Read each transcript and wrote research memos based on initial thoughts and interpretation of the data. - Prominent themes identified in different sets of transcripts. - Constant comparison of the emergent categories was conducted in order to expand and refine each categorical definition. This constant comparison continued until categories and subcategories were refined. Data were compared with other data in the same interview (e.g., responses were compared with other responses to assess consistency), and also compared with data in different interviews (e.g., data in survivors’ interview transcripts were compared with data in their partners’ transcripts). | Analysis at individual level then at dyadic and aggregate (group) levels | Side-by-side readings to compare and categorize themes, codes, or **accounts** within each dyad |
| Mitchell, 2021 | Framework analysis  Software: NR | - Codes for individual interviews developed using questions from interview guide. - Interpreted themes and contextualized their meaning *across* and within couples and developed overarching themes which were coded for facilitators of decision-making to use HIV prevention strategies. - Coded different HIV prevention strategies self-reported by one participant and whether their partner concurred. - *Summary counts calculated to describe concordance for each overarching facilitator of decision-making theme and prevention strategies.* | Analysis at individual level, then at dyadic level informed by results from analysis at individual level, then across dyads | Side-by-side readings to compare and categorize **themes, codes**, or accounts within each dyad |
| Moore, 2020  *Cite:*  *Eisikovits & Koren, 2010*  *Ummel & Achille, 2016* | Thematic analysis at dyad level  Software: NVivo | - Coded patient's transcripts individually. - Constructed a chart containing a table with two columns, patient and partner with rows for themes relating to the dyadic relationship, quality of life, and inductive emerging themes. Chart populated with short summaries or quotations to capture the patient’s experiences and then repeated the process in the second column for the partner. After this, the dyadic thematic analysis began. Notes made during the individual thematic analyses of instances where the patient or partner had overlaps or contrasts were added to each chart. Overlaps or contrasts occurred at both the descriptive (e.g., length of time on dialysis) and perceptual levels (e.g., attitudes toward dialysis). A summary of each dyad was written before proceeding to the next dyad. - *After all dyads had been analyzed in accordance with the above steps, the first author read and re-read all the charts to get a sense of similarities, differences, and key themes emerging from the data set. Visual aids (e.g., mind-maps, dyadic notecards) facilitated comparisons between dyads.* | Analysis at individual level, then at dyadic level informed by results from analysis at individual level, then across dyads | Creation of matrices or summary tables to visually examine and compare codes, **themes**, concepts, or accounts within each dyad |
| Mosher, 2016 | Thematic analysis  Software: ATLAS.ti | - Generated initial codes for each transcript. - Codes sorted into broader themes and compared within each patient-caregiver dyad. | Analysis at individual level, then at dyadic level informed by results from analysis at individual level | Side-by-side readings to compare and categorize themes, **codes**, or accounts within each dyad |
| Mosher, 2017 | Thematic analysis with comparison of themes within each dyad  Software: ATLAS.ti | - Generated initial codes for each transcript. - Codes sorted into broader themes and compared within each patient-caregiver dyad. | Analysis at individual level, then at dyadic level informed by results from analysis at individual level | Side-by-side readings to compare and categorize themes, **codes**, or accounts within each dyad |
| O’Keeffe, 2020  *Cite: Larkin et al., 2019* | IPA principles  Software: NR | - Interviews analyzed according to IPA principles where themes were extracted for each individual and subsequently clustered together for each dyad to identify emergent themes shared within and *across couples*, albeit from different perspectives. - Superordinate, overlapping themes shared within and across couples were identified as constitutive of the lived experience of couples where one partner had sustained a traumatic brain injury. As these three themes already represented a merging of data across individuals and couples, a subsequent breakdown into further sub-themes was considered unnecessary. | Analysis at individual level, then at dyadic level informed by results from analysis at individual level, then across dyads | Side-by-side readings to compare and categorize **themes,** codes, or accounts within each dyad |
| Ohlsson-Nevo, 2012 | Qualitative content analysis  Software: NVivo | - Each interview transcript was divided into meaning units (sentences or paragraphs) and coded. - Codes were compared, and based on differences and similarities, categories were identiﬁed for each interviewee. - Thereafter, mutual subthemes were identiﬁed in the categories for each couple. - Searched for subthemes and themes valid to all data. This ﬁnal step in the analysis included an interpretation of the underlying meaning of all the codes and categories and resulted in one main theme and three mutually exclusive subthemes. | Analysis at individual level, then at dyadic level informed by results from analysis at individual level | Side-by-side readings to compare and categorize **themes,** codes, or accounts within each dyad |
| Patel, 2016a | Grounded theory  Software: MAXQDA | - Textual data were coded within couples (because spouses were paired) using open coding followed by axial coding. - Constant comparisons between the codes and categories helped advance the conceptual understanding of factors that protect against and contribute to HIV transmission for HIV-negative wives. *The comparisons also helped identify whether there were patterns across codes and categories based on couple-level attributes.* - A framework was constructed reflecting pathways and key influences affecting HIV risk for HIV-negative women, and a conceptual framework was generated. | Analysis at dyadic level and then across dyads | Coded within couples or dyadic-level codebook |
| Patel, 2016b | Descriptive analytic approach of case  Software: NR | - One case (couple) from larger study (Patel, 2016a) identified and used descriptive analytic approach to organize the case around couple factors. | Analysis at dyadic level only | Transcripts analyzed from each dyad as a whole |
| Rance, 2017  *Cite:*  *Eisikovits & Koren, 2010* | Mix of inductive and deductive approaches that positioned "the partnership" as the basic unit of analysis and used elements of grounded analysis  Software: NVivo | - Coding frame was developed drawing on the data itself, the interview schedule, and the literature. Consistent with positioning “the partnership” as the basic unit of analysis, transcripts were entered as couples within a qualitative data management program. Consequently, any narrative detail extracted for analysis was readily identiﬁable as part of a broader story of partnership rather than simply an individual account. - Analysis was conducted using a mix of inductive (data-driven) and deductive (analyst-driven) approaches and summary accounts for each couple created. | Analysis at dyadic level only | Written dyad summaries |
| Rando, 2022 | Rapid analytic approach  Software: NR | - Developed a summary template comprised of a neutral domain name corresponding to each question of the semi-structured interview guide, as well as spaces to include key quotations and observations pertaining to emerging themes not captured by the other domains. - Transferred the summary templates into a matrix, allowing all participant comments to be viewed by domain. - "Paired” interviews from a single dyad were always analyzed consecutively so as to enhance the identiﬁcation of any overlap or contrasts in responses. - Coders were therefore not blinded to the dyadic nature of the data, and within-dyad patterns or contrasts were valid sources of themes. | Analysis at individual level, then at dyadic level informed by results from analysis at individual level | Side-by-side readings to compare and categorize themes, codes, or **accounts** within each dyad |
| Rapelli, 2023  *Cite:*  *Ummel & Achille, 2016* | Phenomenological hermeneutic dyadic approach  Software: None | - Analysis of interview of participant interviewed first followed by analysis of interview provided by partner, then across participants to identify themes. - Identified shared themes, connections, and discrepancies between patients and caregivers [aggregate]. - Each dyad was examined to explore within-dyad congruence regarding a topic and major themes. Dyadic congruence was defined as a shared understanding or aligned perspectives regarding a particular topic between the patient and the caregiver. | Analysis at individual level, then at dyadic and aggregate (group) levels based on results from analysis at individual level | Side-by-side readings to compare and categorize **themes**, codes, or accounts within each dyad |
| Raybone, 2019  *Cite:*  *Eisikovits & Koren, 2010* | Thematic analysis with dyadic analysis  Software: NVivo | - Individual interviews coded. - Accounts of one partner compared with other to note similarities and differences. | Analysis at individual level and then at dyadic level | Side-by-side readings to compare and categorize themes, codes, or **accounts** within each dyad |
| Retrum, 2013 | General inductive approach by dyad  Software: ATLAS.ti | - Patient and corresponding caregiver transcripts were viewed side-by-side. Initial dyad codes were created based on patient and caregiver responses to questions about physical and emotional symptoms and concerns about the future. Other codes were created to identify topics addressed by both members of dyads and to reflect communication processes, similar or dissimilar perspectives about a topic, type of congruence, and distress due to the behavior or perspective from the other dyad member. Patient and caregiver quotes that addressed similar topics were linked so they could be examined concurrently. - *Cross-dyad case comparative analysis of linked dyad quotes was also completed.* - To categorize dyads as congruent (or incongruent), the research team reviewed linked quotes to confirm that the quotes were congruent or incongruent. If more than half of the linked quotes for each dyad were congruent (or incongruent), they were categorized as congruent (or incongruent). If dyads appeared to have the same amount of congruence and incongruence, they were placed in a third category labeled ‘‘both.’’ Congruence was defined as consistency in perspective on various aspects of the patient illness and the implications surrounding the illness. - *Once the initial congruence analysis was complete, dyads were then classified according to nature of relationship, gender, and age to explore whether these contextual factors were associated with congruence.* | Analysis at dyadic level and then across dyads | Side-by-side readings to compare and categorize themes, codes, or **accounts** within each dyad |
| Roberto, 2013 | Grounded theory methods, constant comparative method  Software: ATLAS.ti | - Open coding of three waves of data using the constant comparative method. - Focused coding (two rounds):   - After coding all three waves of data, the research team identiﬁed emergent issues about the everyday experiences of couples, which led to the ﬁrst research question. Re-examined codes related to changes in stressors as associated with household responsibilities, assistance to the person with mild cognitive impairment (MCI), and MCI management. One theme that connected these stressors was that care partners discussed shifting roles and responsibilities when they perceived further decline in their spouses’ memory abilities.   - Based on the theme from first round of focused coding and the literature, and consistent with grounded theory methods, explored these shifts further. A new round of focused coding was begun by rereading the interviews of all married couples who had been interviewed three times (including the interviews with the person with MCI when available), looking for changes over time in the person with MCI, the care partner, and the marital relationship (second research question). Field notes written by interviewers were used to provide a richer understanding of the couples’ experiences and verify analytic insights. | Dyadic analysis to further explore one or two relational themes or categories from analysis at individual level | Coded within couples or dyadic-level codebook |
| Rodham, 2010 | IPA  Software: NR | - Followed the IPA ﬁve-step process in three waves:   - First with the transcripts from the people with fibromyalgia syndrome to develop a framework which we used to guide the analysis of the carers’ transcripts.   - Second with the spousal carer transcripts.   - Third with the four dyad transcripts to check whether the differences between the two groups *were reﬂected across the four dyads.* | Analysis at individual level then at dyadic and aggregate (group) levels | Transcripts analyzed from each dyad as a whole |
| Rodrigues, 2022  *Cite:*  *Eisikovits & Koren, 2010*  *Hudson et al., 2020* | Framework analysis with dyadic analysis  Software: MAXQDA | - First coded and analyzed each individual interview, before focusing on the dyad as a unit and systematically identifying consistencies, discrepancies, and omissions in their accounts. - Initial coding frame derived from theoretical framework and modified by codes arising from the interviews. - Data was summarized using thematic matrices, with each cell synthetizing information on a code (column) and individual (row). - Codes were examined for consistency and variation across the sample (e.g. patterns or outliers), aggregating codes where relevant to reflect higher abstraction and analysing the remaining events and processes mentioned by each dyad to identify main pathways and possible variation by socioeconomic status and gender patterns. - Drew conclusions from the patterns and exceptions found in the data. | Analysis at individual level, then at dyadic level informed by results from analysis at individual level | Creation of matrices or summary tables to visually examine and compare **codes**, themes, concepts, or accounts within each dyad |
| Rowland, 2018 | Line-by-line coding  Software: NR | - Each transcript was reviewed line by line to identify signiﬁcant statements about barriers and facilitators to heart healthy diet and physical activity. Couple-focused factors were differentiated from individual experiences if the facilitator or barrier occurred in 1 partner because of or in connection with the other. - Related codes were organized into couple-focused themes. | Analysis at individual level, then at dyadic level informed by results from analysis at individual level | Coded within couples or dyadic-level codebook |
| Ruark, 2024 | Framework analysis  Software: NVivo | - Conducted an individual-level analysis and dyadic-level analysis:   - Read through each pair of couple interviews and abstracted the data into matrices organized both by couple and by themes derived from the interview guides and the interview data.   - Compared within-couple accounts between partners, made notes about areas of agreement and disagreement, and wrote a memo for each couple summarizing key details of their accounts and how they represented themselves.   - Coded by gender and type of disease to examine whether shared illness appraisal and type and amount of support offered varied by disease and gender. - Quantified types of health behaviors and support interactions to explore patterns in the data, such as frequency by gender. | Sequence not specified | Creation of matrices or summary tables to visually examine and compare codes, themes, concepts, or **accounts** within each dyad |
| Sauvé, 2020  *Cite:*  *Ummel & Achille, 2016* | IPA  Software: NR | - Transcripts coded for each participant and analysis highlighted divergences and convergences in themes and meaning-making in partners' respective experience. - Analysis repeated for each couple where each individual account was reread to identify circularity in the information shared by each partner reaching a new understanding of their accounts resulting in a global perception of their shared experience as a couple. - New dyadic understanding re-compared to each partner's individual account to confirm validity of new interpretative deductions offering a relational perspective. | Moving back and forth between individual, dyadic +/- aggregate or cross-dyad levels of analysis | Side-by-side readings to compare and categorize **themes, codes,** or accounts within each dyad |
| Senden, 2015 | Constant comparative method based on grounded theory approach  Software: NVivo | - Interviews analyzed using constant comparative method with cyclic process of data collection and analysis with a constant forward backward movement was used in order to check ﬁndings and adjust sampling based on gained insights. - Analysis also focused on processes of mutual inﬂuence of the perception of patients and relatives. During the analysis, both [dyad] interviews were analyzed on the level of the lived experience of the patient, of the family caregiver and of the interaction between the two. - Through this process of systematic analysis, an inductively generated yet logically structured code tree of patients and relatives emerged. | Analysis at individual level then at dyadic and aggregate (group) levels | Transcripts analyzed from each dyad as a whole |
| Serçe, 2020  *Cite:*  *Eisikovits & Koren, 2010* | Content analysis with dyad coding  Software: NR | - Each individual’s responses to the interview questions were coded. - Common and dissociating points within dyads were investigated. - *After within-dyad analysis, further interpretations included seeking unifying ideas and patterns across dyads.* | Analysis at individual level, then at dyadic level, then across dyads | Side-by-side readings to compare and categorize themes, **codes,** or accounts within each dyad |
| Shilling, 2017 | Framework approach  Software: NVivo | - Developed thematic framework from an initial process of open coding. The framework was applied to the transcripts, indexed by themes and subthemes. - The data were then extracted and summarised in charts grouped by themes and subthemes, incorporating field and reflexive notes where appropriate. - The charts were used to compare and contrast within and between individual interviews, dyads and patient and caregiver groups. | Analysis at individual level, then at dyadic and aggregate (group) levels based on results from analysis at individual level | Creation of matrices or summary tables to visually examine and compare codes, **themes,** concepts, or accounts within each dyad |
| Smith, 2022  *Cite:*  *Eisikovits & Koren, 2010* | Thematic analysis with dyads as the primary unit of analysis  Software: NR | - Coded individual responses from the survivor and partner separately to develop codebook. - Developed dyadic level codebook in order to examine congruence and incongruence of data between each member of the dyad. For example, dyadic-level codes were applied to indicate whether current contraceptive use was described congruently (the same) or incongruently (differently). - Based on the final coding results, developed themes and subthemes in order to collapse emerging findings in to meaningful categories, creating a cohesive narrative. *At this level, researchers compared dyads based on developed thematic results and reported any differences between couples*, resulting in an analysis that included within and across couple differences. - *Each dyad was also categorized according to alignment between their reported contraceptive method and pregnancy intention in order to organize and explore the relationship between contraceptive decision-making and family planning.* | Analysis at individual level, then at dyadic level, then across dyads | Coded within couples or dyadic-level codebook |
| Solomon, 2018  *Cite:*  *Thompson & Walker, 1982* | Interpretive description combined with dyadic analysis and triangulation through constant comparison with novel intradyadic “crossover” technique  Software: NVivo | - Immediately after interviewing each pair, scribed a dyadic vignette. This specialized field note captured impressions of both individuals as well as the dyad as a relational unit. - Created preliminary descriptions and dyadic interpretations. - An intradyadic “crossover” technique developed, tightly analyzing one woman’s responses to questions alongside her partner’s. Line-by-line coding focused broadly on relationship quality: Daughter’s Perception of Relationship or Mother’s Perception of Relationship. Particular attention was paid to concordance and/or discordance between two individual descriptions of relationship quality- did both women construct the same relational experience, or subjectively construct two different relationships? - Compared and contrasted individual descriptions and dyadic interpretations within and *across dyads*. Themes collapsed further underneath emerging relationship quality styles continually returning to earlier transcripts, applying later emerging themes. To visualize analysis along this spectrum, matrices were created. Frequencies of styles, themes, and subthemes within and *across dyads* were assessed to verify findings. - Findings for all dyads were compared against initial dyadic vignettes and early interpretations for finalization. | Analysis at dyadic level and then across dyads | Creation of matrices or summary tables to visually examine and compare codes, themes, **concepts,** or **accounts** within each dyad |
| Spangler, 2018 | Constant comparative approach and thematic analysis  Software: Dedoose | - Broad domains and initial codes were agreed upon based on the semi-structured interview guide. - Once initial coding was complete, the transcripts were finely coded and themes identified using inductive thematic analysis. - Finalised themes were compared and contrasted across all participants in terms of sociodemographic characteristics, as well as among women, among men and within and *between* couples (seroconcordant and serodiscordant, monogamous and polygamous). | Analysis at individual level, then at dyadic and aggregate (group) levels based on results from analysis at individual level (and across dyads) | Side-by-side readings to compare and categorize themes, **codes,** or accounts within each dyad |
| Steinberg, 2024 | Thematic analysis  Software: NVivo | - All transcripts read holistically to identify general themes important for analysis. - General codes- those about independence, the transition, and interpersonal relationship- created. - Inductive thematic coding with reading the transcripts as parent-young adult dyads and reading by groups of parents and young adults. The quotes were assigned to each code and then organized in terms of topicality and ﬂow. This work was interpretive, and focused on meaning-making. | Analysis at individual level then at dyadic and aggregate (group) levels | Coded within couples or dyadic-level codebook |
| Sterba, 2014 | Template analysis  Software: NVivo | - Data analysis employed the template analysis technique where an initial codebook derived from theory and the literature is used, but additional codes can emerge as appropriate. - Explored themes across survivors and caregivers at the group level as well as within dyads (i.e., individual breast cancer survivors and their nominated caregivers). Final coding was conducted independently with routine meetings between coders. | Sequence not specified | Side-by-side readings to compare and categorize **themes,** codes, or accounts within each dyad |
| Sud, 2021a  *Cite:*  *Eisikovits & Koren, 2010* | Thematic analysis/ framework analysis at the dyad level  Software: NVivo | Analysis carried out by comparing and contrasting the transcripts of each individual within dyads to discern similarities and differences and identify patterned meanings. | Analysis at dyadic level only | Side-by-side readings to compare and categorize **themes,** codes, or accounts within each dyad |
| Tan, 2018a  *Cite:*  *Eisikovits & Koren, 2010* | Dyadic qualitative analysis  Software: Dedoose | - Compared and contrasted each individual’s account with that of his partner to discover overlaps between individual narratives and to identify points of corroboration and contradiction between relationship partners, with the couple as the “unit of analysis.” Cross-analyzed significant statements, sentences, and quotes, formulating themes based on both partners’ perspectives on their relationship, health and care engagement, and respective roles in their own and each other’s healthcare. - After examining the data at the dyadic level, conducted individual-level analysis by developing a codebook by reading all transcripts and iteratively generating and revising codes. | Analysis at dyadic level and then at individual level | Side-by-side readings to compare and categorize **themes,** codes, or accounts within each dyad |
| Tan, 2018b  *Cite:*  *Eisikovits & Koren, 2010* | Dyadic qualitative analysis  Software: Dedoose | - Data were ﬁrst analyzed at the couple-level using an approach similar to Eisikovits and Koren (2010) through development of a codebook by iteratively generating and revising codes based on discussions of major themes that emerged from reading all transcripts. To analyze the narratives at the couple-level, compared and contrasted each transcript with that of the partner to discover overlaps between narratives and to identify points of corroboration and contradiction. With the couple as the unit of analysis, important statements were cross-analyzed and themes formulated based on both partners’ perspectives on the role that their relationship played in their respective care engagement. Discrepancies in partners’ accounts were incorporated into the analysis (rather than disregarded) to indicate discord and to signal where closer examination was warranted. - After examining the data at the dyadic level, conducted individual-level analysis by developing a codebook by reading all transcripts highlighting sections of texts to derive themes based on narratives that described partner support offered or received in the context of HIV care. | Analysis at dyadic level and then at individual level | Side-by-side readings to compare and categorize **themes,** codes, or accounts within each dyad |
| Thomeer, 2015  *Cite:*  *Eisikovits & Koren, 2010* | Charmaz's qualitative analysis approach  Software: NVivo | - Line-by-line coding followed by examination of codes at a conceptual level and then identification of gender differences in the codes. Interviews analyzed with attention to contrasts and overlaps between spouse's versions of and ascribed meanings to similar events (dyadic patterns). | Sequence not specified | Side-by-side readings to compare and categorize themes, codes, or accounts within each dyad |
| Thomson, 2020 | Thematic analysis  Software: NR | - Initial list of codes created for interviews and continually revised, condensed, and arranged into meaningful groups that reflected the emerging primary and secondary themes. - Summaries for each interview from every dyad were also created, highlighting similarities and differences in perspectives across participants and time points. - Cross-coding was conducted on three sets of patient-caregiver interviews to develop final coding structure, which was then applied across all interviews. | Analysis at individual level and then at dyadic level | Dyadic summaries and coded within couples or dyadic-level codebook |
| Treloar, 2016  *Cite:*  *Eisikovits & Koren, 2010* | Analysis of couple as a unit  Software: NVivo | - Coding frame was developed and informed by authors' previous couples-related pilot study and by the existing literature. Summaries of coded data were produced. - All transcripts were organized, labelled and analysed in a way that identiﬁed both the participant and their partner. Data from partners were examined together, and then, *themes across couples were explored in three categories: HCV-negative seroconcordant, discordant and HCV-positive concordant.* | Analysis at dyadic level and then across dyads | Transcripts analyzed from each dyad as a whole |
| Tripathee, 2020  *Cite:*  *Eisikovits & Koren, 2010* | Framework approach and dyadic analysis  Software: NVivo | - Identified themes were discussed by authors in detail before coding data. Descriptive accounts were written based on the coded data and charted into framework matrices, which were reviewed by all authors. Each participant was assigned a row and each theme was presented in a column. Synthesising key categories and presenting them in matrices facilitated movement beyond descriptive accounts to provide explanations based on interpretations grounded in the data. - Both partners’ accounts were considered in each theme and coded for dyadic analysis. This exercise facilitated exploration of each partner’s individual accounts, whilst considering the context of their shared life, to understand the basis of their experience and perceptions. | Analysis at individual level, then at dyadic level informed by results from analysis at individual level | Creation of matrices or summary tables to visually examine and compare codes, **themes,** concepts, or accounts within each dyad |
| Umberson, 2016 | Inductive analysis that also included dyadic comparison  Software: NVivo | - All interviews were independently analyzed by the authors using a standardized method of inductive data analysis that emphasizes the dynamic construction of codes. - Line-by-line, data-driven categorization in order to summarize each piece of data as it related to the illness of one or both spouses. - Focused coding to develop categories regarding both the illness and perceptions of marital dynamics regarding the illness, connecting initial line-by-line codes together for conceptual purposes. - Creation of categories and subcategories that related to one another on a theoretical level. - Key component of analysis was a dyadic comparison and consideration of “concordance” and/or “discordance” between spouses, indicating degree of alignment between spouses in regard to constructions of illness and provision and receipt of care. | Sequence not specified | Side-by-side readings to compare and categorize themes, codes, or accounts within each dyad |
| Ummel, 2016a  Details regarding methods also extracted from Ummel, 2016b.  *Cite:*  *Eisikovits & Koren, 2010* | IPA  Software: NR | - Analysis of interview of participant interviewed first followed by analysis of interview provided by partner, then across participants to identify themes. - Mapped dyadic data visually along the timeline of the transplant trajectory to get an idea of the parallel progression between the donor’s and the recipient’s experience of the living kidney donation. By doing so, authors were able to identify overlap and contrast in the data. | Analysis at individual level and then at dyadic level | Visual mapping of dyadic data |
| Vandenberg, 2024  *Cite:*  *Eisikovits & Koren, 2010*  *Manning & Kunkle, 2015* | Thematic analysis  (Case studies)  Software: NR | - Individual transcripts coded. - Passages relating to death communication identified for each dyad partner and then compared statements by dyads for concordances and discordances. - Case studies generated and compared. Case studies were compared across the data set for larger patterns to develop a typology of communication. | Dyadic analysis to further explore one or two relational themes or categories from analysis at individual level | Side-by-side readings to compare and categorize themes, codes, or **accounts** within each dyad |
| Wang, 2021 | Directed content analysis  Software: NVivo | - In the first-cycle coding, a priori codes were developed from framework and codes expanded to reflect experiences specific to individuals with cognitive impairment and their care partners. Authors focused on their dyadic perceptions and experiences, compared overlaps and contrasts to enhance understanding of the nature of their relationships and experience. - In the second-cycle coding, the coded texts were arranged into categories and subcategories based on how they were related. Eventually, patterns were synthesized. | Analysis at dyadic level only | Side-by-side readings to compare and categorize themes, codes, or **accounts** within each dyad |
| Wang, 2022 | Conventional and directed content analysis  Analysis: ATLAS.ti | - A priori codes were developed from framework and codes expanded to reflect experiences specific to individuals with cognitive impairment and their care partners. Focused on their dyadic perceptions and experiences to enhance understanding of the nature of their relationships and experience. Patterns and differences were identiﬁed among participants *and across dyads.* - Patterns, themes, and relationships between categories were noted in analysis to develop ideas about themes and interpretation. | Analysis at dyadic level and then across dyads | Side-by-side readings to compare and categorize themes, codes, or **accounts** within each dyad |
| Werner, 2021  *Cite:*  *Hochman et al., 2020* | Dyadic analysis  Software: NR | - All interviews were read sequentially noting first impressions derived from the interviews and main topics that appeared. - Open coding of the data, aimed at detecting and identifying units of meaning arising from each interview separately, followed by a search for repeated patterns of meaning that emerged from the various interviews. - Assessed the associations between categories and grouped them into main themes. - Focused on dyads as the unit of analysis by examining the themes that emerged from each dyad, and assessing overlaps and contrasts between the partners. The aim was to reach an understanding of the shared dyad reality based on the way in which each individual perceived the other and the relationship as a whole. - *Conducted a cross-dyad analysis, aimed at identifying differences and similarities between all dyads in the study.* | Analysis at individual level, then at dyadic level informed by results from analysis at individual level, then across dyads | Side-by-side readings to compare and categorize **themes**, codes, or accounts within each dyad |
| White, 2016  *Cite:*  *Eisikovits & Koren, 2010* | Framework analysis and dyadic analysis  Software: NR | - Familiarization with the data through transcribing, repeatedly reading and reﬂecting on the data. - Development of an initial thematic framework based on key concepts from participants' accounts, which was used to begin organising and classifying the data. - Identification and indexing portions of data relating to speciﬁc themes, represented as codes. - Reorganising data into charts of the themes, using headings to compare across the whole dataset whilst keeping reference to the original context. - Searching for patterns and explanations and using diagrams to develop interpretations. - In addition to analysis of individual interviews, dyadic analysis compared overlaps and contrasts within and between couples. This highlighted differences in couples' relational styles as well as individual differences for the sample of patients compared with partners. | Sequence not specified | Creation of matrices or summary tables to visually examine and compare codes, **themes,** concepts, or accounts within each dyad |
| Wise, 2010 | Grounded theory  Software: NR | - The unit of analysis was the dyad. However, authors began by analyzing the patient’s and care partner’s interviews separately and then mapped them for narrative convergence, divergence, and/or elaboration.   - Phase 1: Open coding through line-by-line analysis of 4 interviews selected for their range of experience and participant characteristics, identification of themes and categorization of them into a preliminary model addressing the research questions.   - Phase 2: Coded the remaining interviews and reﬁned the model by incorporating survey data [mixed methods]. | Analysis at individual level and then at dyadic level | Side-by-side readings to compare and categorize themes, codes, or **accounts** within each dyad |
| Wood, 2020 | Framework analysis  Software: NR | - Couple's data was analyzed side by side to allow for greater insight across the pair. - Established preliminary codes based on the questions posed and theoretical framework. - Charting and organizing the data into broad semantic themes allowed discovery of repeated patterns of meaning, although these themes remained ﬂexible during the investigation and were continually discussed and reﬁned. - Thematic statements were analyzed and interpreted for relevance to each couple and participant quotes were then selected to best represent the research question and literature. | Analysis at dyadic level only | Transcripts analyzed from each dyad as a whole |
| Wrubel, 2010 | Narrative analysis  Case studies with cross-case analysis  Software: ATLAS.ti | - Coded at dyadic level (to identify characteristics of the couple relationship; (ii) to note narrative accounts of specific events that illustrated the couple dynamic; and (iii) to identify attitudes, actions, and strategies concerning taking medication in each case), in order to examine aspects across all cases. - Began narrative data analysis with broad questions: Which interactions illustrate relationship dynamics? What are the roles of HIV and antiretroviral therapy (ART) in the relationship? What are the couple dynamics around health issues; around medication adherence practices? - Developed codes that reflected what was said in the interviews. Codes were further refined into index codes to demarcate themes and marker codes to note the presence of actions, attitudes, feelings, and experiences that were relevant to the study questions. - Written case studies were completed by one team member. Interviews from both partners constituted a case. Case studies articulated the couple dynamic both in general and as it specifically related to health issues and ART adherence. Case studies addressed interactive dynamics that were thematic for the couple, with particular attention to health- and adherence-related support that was offered or not, and that was accepted or not. Case studies also examined personal meanings that were explicit in the narratives and that were relevant to the couple support dynamic and/ or individual adherence. - *Cross-case analysis in which the cases (couples) were grouped according to similarities in patterns of themes, personal meanings, attitudes, and actions completed data analyses.* | Analysis at dyadic level and then across dyads | Dyadic summaries and coded within couples or dyadic-level codebook |

Supplementary Table 3. Analytic advantages and challenges

| **First author, Year** | **Analytic advantages** | **Analytic challenges and ways of addressing them** |
| --- | --- | --- |
| Abshire Saylor, 2023 | NR | For most domains, value assignments were straightforward, but sometimes, an interpretive level of analysis through study team discussion and consensus was necessary to fully explore the interaction of overlapping contextual domains. |
| Braybrook, 2017 | NR | One challenge that couple researchers will likely face during analysis is differing accounts. Attempting to get a “complete” story from couples can be challenging for joint and individual interviews. |
| Collaço, 2021 | NR | Process was time-consuming  The analysis process of dyadic data was initially experimental, and we were therefore developing the process as the analysis continued. Initially, one-sentence summaries for the codes were created, but after further analysis and creation of codes, we realized that more information was needed in the dyadic codes/summaries, as the context was not always clear. As there were no quotes in the Excel spreadsheet of the framework matrices, the dyadic codes became less clear and lost contextual meaning; therefore, we went back to the original transcripts and ensured more detail was placed in the summary tables. Adding detailed quotes in the summary table provided more context and clarification of the summaries. Providing this level of detail at this stage reduced the need to look back at transcripts too often along the analysis process.  Bringing together the experiences of couples interviewed separately was a more complex process than anticipated. The first challenge was analyzing members of the couples’ different perceptions of one another’s experiences. A solution we developed to account for these differences in perception was to code these views under a general/broad term, for example, in this instance: “Relational communication.” When conducting Stage 8—interpreting the data of the analysis, the differing perceptions of certain parts of their experience were incorporated in the dyadic summary.  Another challenge of this analysis was an initial overlap of codes throughout the different parts of the couples’ experiences. For example, one partner described difficulties in communication in relation to the process of treatment decision making. Therefore, this extract could be coded under “relational communication” and “treatment decision making.” It highlighted the challenges in separating experiences into simplistic categories. Experiences interconnect in many ways and are part of the whole experience of the couple. We realized our categories needed to be broader so they could be applied more clearly. To address this, the dyadic summary code names were kept broad and incorporated context of overlapping codes to provide further detail and depth to that part of the experience or impact. |
| Hudson, 2020 | NR | Limitations of directive questioning.  The identification of what is omitted is insightful in understanding the factors that do and do not constitute individuals’ social worlds. Furthermore, including the analytic category ‘omission’ was necessary in order to avoid an incomplete coding exercise. It became apparent early on in the activity that if the data could only be coded to ‘contrast’ or ‘overlap’, this would have left a considerable amount of data uncoded.  Dyadic method of questioning (which involved asking partners similarly themed questions), and a sub-set of interview questions designed to allow more direct comparison of perspectives in analysis. |
| Kendall, 2010 | NR | Quantity of data generated can otherwise rapidly prove overwhelming. |
| Kitko, 2015 | NR | No data were collected on relationship quality or conflict resolution within the dyad which would have provided more depth to the analysis. The interview guide would have been strengthened with additional questions that focused on relational aspects within the dyad. |
| Martin-Matthews, 2022  Secondary analysis | Findings advance current understanding of the role of older clients in the dynamics of home care, as only few studies have specifically analysed client–carer dyads to investigate this issue. | The main limitation of the present research is that matching older clients and carers was not planned from the beginning of the project. Therefore, no specific questions to compare and contrast their experiences were included. |
| Moore, 2020 | Dyadic-level charting provided a systematic examination of individual and dyadic experiences and emerging patterns within and across dyads. | Of the three dyads who requested joint interviews, one partner whispered her thoughts to the first author when the patient left the room; the implications of these were considered during analysis.  Dyadic analysis posed issues when seeking participant validation and presenting the data in a way that maintained the confidentiality of the dyad. Furthermore, pseudonyms were not used nor were dyadic-level details provided which may prevent readers from seeing the complex and nuanced experiences occurring within individuals and dyads. |
| Retrum, 2013 | NR | Although instances of congruence or incongruence were easily identified, classifying dyads as entirely congruent or incongruent was not simple. |
| Ummel, 2016 | Protecting internal confidentiality can then be more challenging to achieve, and additional care must be taken to ensure details are rendered sufficiently anonymous to prevent either party from being exposed. If we presume that one member of the dyad recognizes his or her own words, it follows that the other dyad’s member anonymity is at risk. This loop in confidentiality is of particular relevance if members have shared neither their transcripts nor the content of their interviews with each other. This was definitely the main issue in our research. |  |
| Vandenberg, 2024  Secondary analysis) | NR | Analysis was not a speciﬁc aim of the parent study and secondary data analysis was used to explore the research question. |

Supplementary Table 4. Methodological approaches of studies reporting a guiding methodology^*^

| **Methodological approach** | | n / 106 (%) |
| --- | --- | --- |
|  | |  |
| **Dyadic or multiperspective methodological approach** | | **20 (18.9)** |
| *Used in combination with a second qualitative methodology* | | *12 (11.3)* |
|  | IPA with dyadic and/or multiperspectival design | 6 |
|  | Dyadic phenomenological hermeneutic research design | 1 |
|  | Case study (case = dyad) | 4 |
|  | Qualitative descriptive design with a dyadic approach | 1 |
|  |  |  |
| *Dyadic methodologies without reference to a second qualitative methodology* | | *8 (7.5)* |
|  | Dyadic study design | 3 |
|  | Multiperspective analysis methodology | 1 |
|  | Dyadic qualitative methodology | 1 |
|  | Longitudinal qualitative partner study | 1 |
|  | Qualitative approach that elicited both patient and caregiver perspectives | 1 |
|  | Methodology that positioned partnerships as primary unit of analysis | 1 |
|  | |  |
| **Non-dyadic methodological approach** | | **39 (36.8)** |
|  | Phenomenological research | 13 |
|  | Qualitative description | 7 |
|  | Ethnography | 6 |
|  | Grounded theory | 3 |
|  | Mixed or multi-methods | 5 |
|  | Other^†^ | 5 |
|  |  |  |
| **No methodological approach reported** | | **47 (44.3)** |

IPA: interpretative phenomenological analysis

^*^Apart from qualitative design

^†^Methodologies reported in single studies

Supplementary Table 5. Methods of analysis used in combination with dyadic analysis

| **Analytic method** | **n / 106 (%)** |
| --- | --- |
| Thematic analysis | 21 (19.8) |
| Constant comparative approach or procedures of grounded theory | 15 (14.2) |
| Framework analysis | 14 (13.2) |
| Interpretative phenomenological analysis | 13 (12.3) |
| Interpretive description | 2 (1.9) |
| Other phenomenological analysis methods | 4 (3.8) |
| Content analysis | 9 (8.5) |
| General inductive analysis | 3 (2.8) |
| ≥2 methods of analysis | 10 (9.4) |
| Other^*^ | 7 (6.6) |
| No other method reported | 8 (7.5) |

^*^Methods reported in single studies

Supplementary Table 6. Dyadic conceptualization of included studies

|  | **Participant selection** | | | | **Data collection** | | | **Trustworthiness and rigour** |
| --- | --- | --- | --- | --- | --- | --- | --- | --- |
| **First author, Year** | **Inclusion/ exclusion criteria** | **Recruitment** | **Sampling strategy or framework** | **Sample** | **Interview questions** | **Saturation (level)** | **Interview timing** |  |
| Abendschein, 2021 | Participation of corresponding dyad member not required | NR | Sampling reported but not who it was directed towards | Dyads and singletons | Similar, related, or same | Unclear | NR | Not in reference to dyadic data |
| Abshire Saylor, 2023 | Both dyad members required to participate | Stepwise approach where one dyad member recruited and then partner subsequently recruited | Sampling directed at dyad | Dyads only | Similar, related, or same | Unclear | NR | Not in reference to dyadic data |
| Abulaiti, 2022 | Participation of corresponding dyad member not required | NR | Sampling reported but not who it was directed towards | Dyads only | Similar, related, or same | Unclear | NR | Not in reference to dyadic data |
| Allen, 2021 | Both dyad members required to participate | NR | NR | Dyads only | Similar, related, or same | NR | NR | Not in reference to dyadic data |
| Allen, 2023 | Both dyad members required to participate | Stepwise approach where one dyad member recruited and then partner subsequently recruited | Sampling directed at one dyad member | Dyads only | NR | Unclear | NR | Not in reference to dyadic data |
| Antoine, 2013 | Both dyad members required to participate | Stepwise approach where one dyad member recruited and then partner subsequently recruited | Sampling directed at one dyad member | Dyads only | Similar, related, or same | NR | NR | NR |
| Antoine, 2018 | Dyad-level inclusion criteria | NR | NR | Dyads only | Similar, related, or same | NR | NR | NR |
| Berridge, 2020 | Participation of corresponding dyad member not required | Stepwise approach where one dyad member recruited and then partner subsequently recruited | NR | Dyads and singletons | Similar, related, or same | Aggregate | Sequential | Not in reference to dyadic data |
| Birtwistle, 2022 | Participation of corresponding dyad member not required | Recruitment directed at one dyad member | NR | Dyads only | Similar, related, or same | NR | NR | Not in reference to dyadic data |
| Boulicault, 2023 | Both dyad members required to participate | Recruitment directed at one dyad member | Sampling directed at one dyad member | Dyads only | Similar, related, or same | NR | NR | Not in reference to dyadic data |
| Boyle, 2013a | Dyad-level inclusion criteria | Dyad members recruited together | Sampling directed at dyad and one dyad member | Dyads only | NR | NR | NR | Not in reference to dyadic data |
| Boyle, 2013b | Dyad-level inclusion criteria | Dyad members recruited together | NR | Dyads only | NR | NR | NR | Not in reference to dyadic data |
| Boyle, 2013c | Dyad-level inclusion criteria | Dyad members recruited together | NR | Dyads only | NR | NR | NR | Not in reference to dyadic data |
| Boyle, 2014a | Dyad-level inclusion criteria | Dyad members recruited together | Sampling directed at dyad | Dyads only | NR | Dyadic | NR | Not in reference to dyadic data |
| Boyle, 2014b | Dyad-level inclusion criteria | Dyad members recruited together | Sampling directed at one dyad member | Dyads only | NR | Unclear | NR | NR |
| Boyle, 2017 | Dyad-level inclusion criteria | Dyad members recruited together | Sampling directed at dyad and one dyad member | Dyads only | NR | NR | NR | Not in reference to dyadic data |
| Brooks, 2014 | NR | Stepwise approach where one dyad member recruited and then partner subsequently recruited | NR | Dyads only | NR | NR | NR | Not in reference to dyadic data |
| Buck, 2013 | Dyad-level inclusion criteria | Dyad members recruited together | NR | Dyads only | Similar, related, or same | NR | Sequential | Not in reference to dyadic data |
| Chan, 2017 | Participation of corresponding dyad member not required | Stepwise approach where one dyad member recruited and then partner subsequently recruited | Sampling directed at one dyad member | Dyads and triads | Similar, related, or same | Aggregate | NR | **In reference to dyadic data** |
| Catona, 2022 | Participation of corresponding dyad member not required | Stepwise approach where one dyad member recruited and then partner subsequently recruited | NR | Dyads only | Similar, related, or same | Unclear | NR | NR |
| Chen, 2015 | Participation of corresponding dyad member not required | Stepwise approach where one dyad member recruited and then partner subsequently recruited | Sampling directed at one dyad member | Dyads only | NR | Unclear | Sequential | Not in reference to dyadic data |
| Collaço, 2019  Collaço, 2021a | Both dyad members required to participate | Stepwise approach where one dyad member recruited and then partner subsequently recruited | Sampling directed at one dyad member | Dyads only | Similar, related, or same | Unclear | NR | Not in reference to dyadic data |
| Conroy, 2017 | Dyad-level inclusion criteria | Stepwise approach where one dyad member recruited and then partner subsequently recruited | Sampling directed at one dyad member | Dyads only | Similar, related, or same | NR | Concurrent | NR |
| Conroy, 2018 | Dyad-level inclusion criteria | Stepwise approach where one dyad member recruited and then partner subsequently recruited | Sampling directed at dyad | Dyads only | Similar, related, or same | NR | Concurrent | Not in reference to dyadic data |
| Conroy, 2019 | Dyad-level inclusion criteria | Stepwise approach where one dyad member recruited and then partner subsequently recruited | Sampling directed at dyad | Dyads only | Similar, related, or same | NR | Concurrent | Not in reference to dyadic data |
| Conroy, 2020 | Dyad-level inclusion criteria | Stepwise approach where one dyad member recruited and then partner subsequently recruited | Sampling directed at dyad | Dyads only | Similar, related, or same | Dyadic | Concurrent | Not in reference to dyadic data |
| Constant, 2022 | Dyad-level inclusion criteria | Dyad members recruited together | NR | Dyads only | Similar, related, or same | NR | NR | Not in reference to dyadic data |
| DeGroot, 2021 | Dyad-level inclusion criteria | NR | Sampling directed at dyad | Dyads only | Similar, related, or same | Unclear | NR | Not in reference to dyadic data |
| Demirtepe-Saygili, 2022 | Dyad-level inclusion criteria | NR | NR | Dyads only | Similar, related, or same | Unclear | Concurrent | **In reference to dyadic data** |
| Dobrina, 2016 | Dyad-level inclusion criteria | NR | Sampling directed at one dyad member | Dyads only | Similar, related, or same | Dyadic | NR | Not in reference to dyadic data |
| Ekelund, 2010 | Participation of corresponding dyad member not required | Recruitment directed at one dyad member | Sampling directed at one dyad member | Dyads and singletons | Similar, related, or same | NR | Sequential | NR |
| Ekstam, 2011 | Participation of corresponding dyad member not required | NR | Sampling directed at dyad | Dyads only | Similar, related, or same | NR | NR | Not in reference to dyadic data |
| Engeli, 2016 | Both dyad members required to participate | NR | NR | Dyads only | Similar, related, or same | NR | NR | NR |
| Eriksson, 2010 | Both dyad members required to participate | Dyad members recruited together | Sampling directed at dyad | Dyads only | Similar, related, or same | NR | NR | Not in reference to dyadic data |
| Farquhar, 2017 | Participation of corresponding dyad member not required | Stepwise approach where one dyad member recruited and then partner subsequently recruited | NR | Dyads only | Similar, related, or same | Unclear | NR | Not in reference to dyadic data |
| Ferreira, 2020 | Participation of corresponding dyad member not required | NR | Sampling reported but not who it was directed towards | Dyads only | Similar, related, or same | Unclear | Sequential | Not in reference to dyadic data |
| Fonner, 2021 | Dyad-level inclusion criteria | NR | Sampling directed at dyad and one dyad member | Dyads and singletons | Similar, related, or same | Unclear | Concurrent | Not in reference to dyadic data |
| Gamarel, 2016 | Both dyad members required to participate | Dyad members recruited together | NR | Dyads only | Similar, related, or same | NR | NR | NR |
| Goldsmith, 2016 | Both dyad members required to participate | Dyad members recruited together | NR | Dyads only | Similar, related, or same | Unclear | Concurrent | Not in reference to dyadic data |
| Gorman, 2020 | Participation of corresponding dyad member not required | NR | Sampling directed at one dyad member | Dyads only | Similar, related, or same | Unclear | Concurrent | NR |
| Grivel, 2023 | Both dyad members required to participate | Stepwise approach where one dyad member recruited and then partner subsequently recruited | NR | Dyads only | Similar, related, or same | Unclear | Sequential | Not in reference to dyadic data |
| Hedman, 2019 | Dyad-level inclusion criteria | NR | NR | Dyads only | NR | NR | NR | NR |
| Heid, 2016 | Participation of corresponding dyad member not required | Dyad members recruited together | Sampling reported but not who it was directed towards | Dyads only | Similar, related, or same | Unclear | Sequential | Not in reference to dyadic data |
| Hellström, 2013 | Participation of corresponding dyad member not required | NR | NR | Dyads only | Similar, related, or same | NR | NR | **In reference to dyadic data** |
| Hellström, 2016 | Participation of corresponding dyad member not required | NR | NR | Dyads only | Similar, related, or same | NR | NR | **In reference to dyadic data** |
| Hendryckx, 2024 | Participation of corresponding dyad member not required | NR | Sampling reported but not who it was directed towards | Dyads and triads | Similar, related, or same | Individual | NR | **In reference to dyadic data** |
| Hopkinson, 2016 | Participation of corresponding dyad member not required | Stepwise approach where one dyad member recruited and then partner subsequently recruited | NR | Dyads only | Similar, related, or same | NR | NR | Not in reference to dyadic data |
| Huang, 2021 | Participation of corresponding dyad member not required | NR | NR | Dyads only | Similar, related, or same | NR | Sequential or concurrent | Not in reference to dyadic data |
| Hudson, 2016 | Dyad-level inclusion criteria | Dyad members recruited together | Sampling directed at dyad | Dyads only | Similar, related, or same | NR | Sequential or concurrent | NR |
| Iannarino, 2022 | Participation of corresponding dyad member not required | NR | Sampling reported but not who it was directed towards | Dyads only | Similar, related, or same | Dyadic | NR | NR |
| Jere, 2023 | Dyad-level inclusion criteria | Stepwise approach where one dyad member recruited and then partner subsequently recruited | NR | Dyads only | Similar, related, or same | NR | NR | NR |
| Kano, 2022 | Participation of corresponding dyad member not required | Stepwise approach where one dyad member recruited and then partner subsequently recruited | NR | Dyads and singletons | Similar, related, or same | NR | NR | NR |
| Kitko, 2015 | Both dyad members required to participate | NR | Sampling directed at dyad and one dyad member | Dyads only | Similar, related, or same | NR | NR | **In reference to dyadic data** |
| Lamore, 2019 | Both dyad members required to participate | Stepwise approach where one dyad member recruited and then partner subsequently recruited | NR | Dyads only | Similar, related, or same | NR | NR | Not in reference to dyadic data |
| Loaring, 2015 | Dyad-level inclusion criteria | Dyad members recruited together | Sampling reported but not who it was directed towards | Dyads only | Similar, related, or same | NR | Sequential | Not in reference to dyadic data |
| Loup, 2023 | Participation of corresponding dyad member not required | NR | NR | Dyads and singletons | Similar, related, or same | Individual | Sequential | Not in reference to dyadic data |
| Manceau, 2023 | Dyad-level inclusion criteria | Dyad members recruited together | Sampling directed at dyad | Dyads only | Similar, related, or same | NR | Concurrent | Not in reference to dyadic data |
| Martin-Matthews, 2022 | Participation of corresponding dyad member not required | Recruitment directed at one dyad member | Sampling directed at one dyad member | Dyads only | NR | NR | NR | NR |
| McCarthy, 2015 | Participation of corresponding dyad member not required | Dyad members recruited together | Sampling reported but not who it was directed towards | Dyads only | NR | NR | Concurrent | NR |
| McCarthy, 2020 | Dyad-level inclusion criteria | Dyad members recruited together | Sampling directed at dyad | Dyads only | Similar, related, or same | Dyadic | NR | Not in reference to dyadic data |
| McCarthy, 2022 | Dyad-level inclusion criteria | Dyad members recruited together | Sampling directed at dyad and one dyad member | Dyads only | Similar, related, or same | NA | Concurrent | Not in reference to dyadic data |
| Miller, 2013 | Dyad-level inclusion criteria | NR | Sampling directed at one dyad member | Dyads and singletons | Similar, related, or same | Unclear | NR | Not in reference to dyadic data |
| Mitchell, 2021 | Dyad-level inclusion criteria | Stepwise approach where one dyad member recruited and then partner subsequently recruited | NR | Dyads and singletons | Similar, related, or same | NR | NR | NR |
| Moore, 2020 | Both dyad members required to participate | Recruitment directed at one dyad member | Sampling directed at dyad and one dyad member | Dyads only | Similar, related, or same | NR | Sequential or concurrent | **In reference to dyadic data** |
| Mosher, 2016 | Participation of corresponding dyad member not required | Stepwise approach where one dyad member recruited and then partner subsequently recruited | NR | Dyads only | Similar, related, or same | Unclear | Sequential | NR |
| Mosher, 2017 | Participation of corresponding dyad member not required | Stepwise approach where one dyad member recruited and then partner subsequently recruited | NR | Dyads only | Similar, related, or same | Unclear | Sequential | NR |
| O’Keeffe, 2020 | Dyad-level inclusion criteria | NR | Sampling directed at one dyad member | Dyads and singletons | Similar, related, or same | NR | Sequential | Not in reference to dyadic data |
| Ohlsson-Nevo, 2012 | Participation of corresponding dyad member not required | Recruitment directed at one dyad member | NR | Dyads only | Similar, related, or same | NR | NR | Not in reference to dyadic data |
| Patel, 2016a | Dyad-level inclusion criteria | Recruitment directed at one dyad member | Sampling directed at dyad | Dyads only | Similar, related, or same | Dyadic | NR | **In reference to dyadic data** |
| Patel, 2016b | Dyad-level inclusion criteria | Recruitment directed at one dyad member | Sampling directed at dyad | Dyads only | Similar, related, or same | NA | NR | NR |
| Rance, 2017 | Both dyad members required to participate | NR | Sampling directed at dyad | Dyads only | Similar, related, or same | NR | Sequential | NR |
| Rando, 2022 | Both dyad members required to participate | Dyad members recruited together | NR | Dyads and singletons | Similar, related, or same | NR | Sequential | NR |
| Rapelli, 2023 | Participation of corresponding dyad member not required | Recruitment directed at one dyad member | Sampling directed at dyad | Dyads only | Similar, related, or same | Dyad | Sequential | **In reference to dyadic data** |
| Raybone, 2019 | Both dyad members required to participate | NR | Sampling directed at dyad | Dyads only | Similar, related, or same | Unclear | Sequential | Not in reference to dyadic data |
| Retrum, 2013 | Participation of corresponding dyad member not required | Recruitment directed at one dyad member | Sampling directed at one dyad member | Dyads only | Similar, related, or same | NR | NR | **In reference to dyadic data** |
| Roberto, 2013 | Both dyad members required to participate | Stepwise approach where one dyad member recruited and then partner subsequently recruited | Sampling directed at dyad | Dyads only | Similar, related, or same | NR | NR | **In reference to dyadic data** |
| Rodham, 2010 | Dyad-level inclusion criteria | Dyad members recruited together | NR | Dyads only | Similar, related, or same | NR | Sequential | Not in reference to dyadic data |
| Rodrigues, 2022 | Participation of corresponding dyad member not required | NR | Sampling directed at dyad | Dyads only | Similar, related, or same | NR | NR | Not in reference to dyadic data |
| Rowland, 2018 | Participation of corresponding dyad member not required | NR | Sampling directed at dyad | Dyads only | Similar, related, or same | NR | Sequential | Not in reference to dyadic data |
| Ruark, 2024 | Dyad-level inclusion criteria | Stepwise approach where one dyad member recruited and then partner subsequently recruited | Sampling directed at dyad and one dyad member | Dyads only | Similar, related, or same | NR | Concurrent | **In reference to dyadic data** |
| Sauvé, 2020 | Both dyad members required to participate | Separate | Sampling directed at dyad | Dyads only | Similar, related, or same | NR | Sequential | **In reference to dyadic data** |
| Senden, 2015 | Participation of corresponding dyad member not required | Recruitment directed at one dyad member | Sampling directed at one dyad member | Dyads and singletons | Similar, related, or same | Unclear | Sequential | Not in reference to dyadic data |
| Serçe, 2020 | Participation of corresponding dyad member not required | NR | Sampling reported but not who it was directed towards | Dyads only | Similar, related, or same | Unclear | Sequential | **In reference to dyadic data** |
| Shilling, 2017 | Both dyad members required to participate | Stepwise approach where one dyad member recruited and then partner subsequently recruited | Sampling directed at one dyad member | Dyads and singletons | Similar, related, or same | NR | NR | Not in reference to dyadic data |
| Smith, 2022 | Participation of corresponding dyad member not required | NR | NR | Dyads only | Similar, related, or same | Unclear | Concurrent | Not in reference to dyadic data |
| Solomon, 2018 | Both dyad members required to participate | Dyad members recruited together | Sampling reported but not who it was directed towards | Dyads only | Similar, related, or same | Dyadic | Sequential | **In reference to dyadic data** |
| Spangler, 2018 | Participation of corresponding dyad member not required | Stepwise approach where one dyad member recruited and then partner subsequently recruited | Sampling directed at one dyad member | Dyads only | Similar, related, or same | NR | NR | NR |
| Steinberg, 2024 | Dyad-level inclusion criteria | Dyad members recruited together | NR | Dyads only | Similar, related, or same | NR | NR | NR |
| Sterba, 2014 | Participation of corresponding dyad member not required | Stepwise approach where one dyad member recruited and then partner subsequently recruited | Sampling reported but not who it was directed towards | Dyads and singletons | Similar, related, or same | Unclear | Sequential | NR |
| Sud, 2021a | Participation of corresponding dyad member not required | Stepwise approach where one dyad member recruited and then partner subsequently recruited | NR | Dyads and triads | Similar, related, or same | NR | Sequential | NR |
| Tan, 2018a | Both dyad members required to participate | Recruitment directed at one dyad member | Sampling directed at dyad | Dyads only | Similar, related, or same | NR | Concurrent | NR |
| Tan, 2018b | Both dyad members required to participate | Recruitment directed at one dyad member | Sampling directed at dyad | Dyads only | NR | NR | Concurrent | NR |
| Thomeer, 2015 | Dyad-level inclusion criteria | NR | Sampling directed at dyad | Dyads only | Similar, related, or same | Unclear | NR | Not in reference to dyadic data |
| Thomson, 2020 | Both dyad members required to participate | Recruitment directed at one dyad member | Sampling directed at one dyad member | Dyads only | Similar, related, or same | Unclear | Sequential | NR |
| Treloar, 2016 | Both dyad members required to participate | NR | Sampling directed at dyad | Dyads and singletons | Similar, related, or same | NR | Sequential | NR |
| Tripathee, 2020 | Participation of corresponding dyad member not required | Stepwise approach where one dyad member recruited and then partner subsequently recruited | NR | Dyads only | Similar, related, or same | NR | Sequential or concurrent | NR |
| Umberson, 2016 | Dyad-level inclusion criteria | Dyad members recruited together | Sampling directed at dyad | Dyads only | Similar, related, or same | NR | NR | NR |
| Ummel, 2016a | Participation of corresponding dyad member not required | Dyad members recruited separately | Sampling directed at dyad and one dyad member | Dyads only | Similar, related, or same | Unclear | Sequential | NR |
| Vandenberg, 2024 | Participation of corresponding dyad member not required | NR | Sampling directed at one dyad member | Dyads only | NR | NR | NR | NR |
| Wang, 2021 | Dyad-level inclusion criteria | NR | Sampling directed at one dyad member | Dyads only | Similar, related, or same | NR | NR | **In reference to dyadic data** |
| Wang, 2022 | Participation of corresponding dyad member not required | NR | Sampling directed at dyad | Dyads only | Similar, related, or same | Dyadic | Sequential | NR |
| Werner, 2021 | NR | Stepwise approach where one dyad member recruited and then partner subsequently recruited | Sampling reported but not who it was directed towards | Dyads only | Similar, related, or same | NR | Sequential | **In reference to dyadic data** |
| White, 2016 | Dyad-level inclusion criteria | Recruitment directed at one dyad member | Sampling directed at dyad | Dyads only | Similar, related, or same | Individual and dyadic | Sequential | **In reference to dyadic data** |
| Wise, 2010 | Dyad-level inclusion criteria | Recruitment directed at one dyad member | NR | Dyads and singletons | Similar, related, or same | NR | NR | NR |
| Wood, 2020 | Dyad-level exclusion criteria | NR | Sampling directed at dyad | Dyads only | Similar, related, or same | Unclear | NR | Not in reference to dyadic data |
| Wrubel, 2010 | Participation of corresponding dyad member not required | Dyad members recruited together | NR | Dyads only | Similar, related, or same | NR | Concurrent | NR |


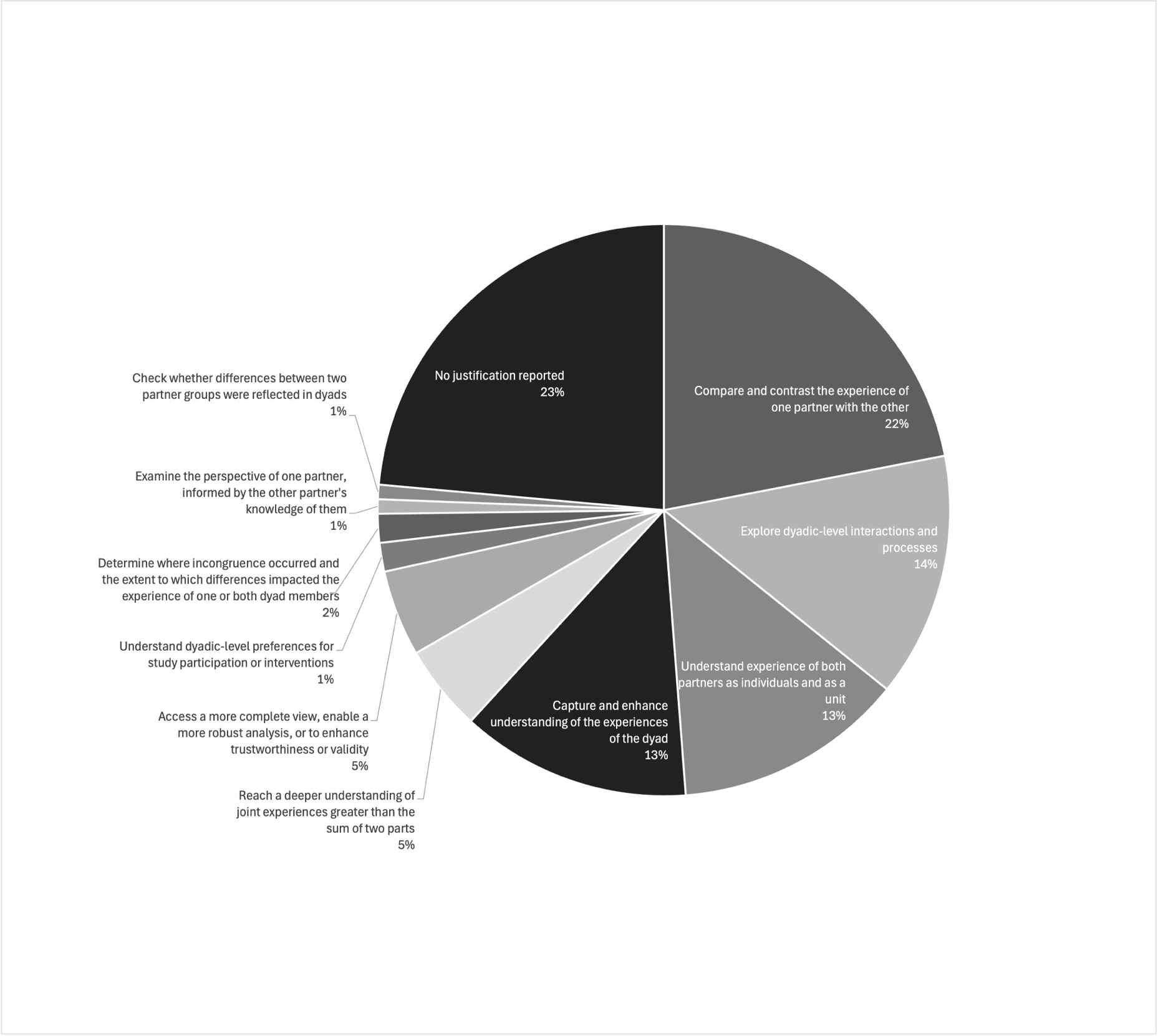


Supplementary Figure 1. Justifications for performing dyadic analysis


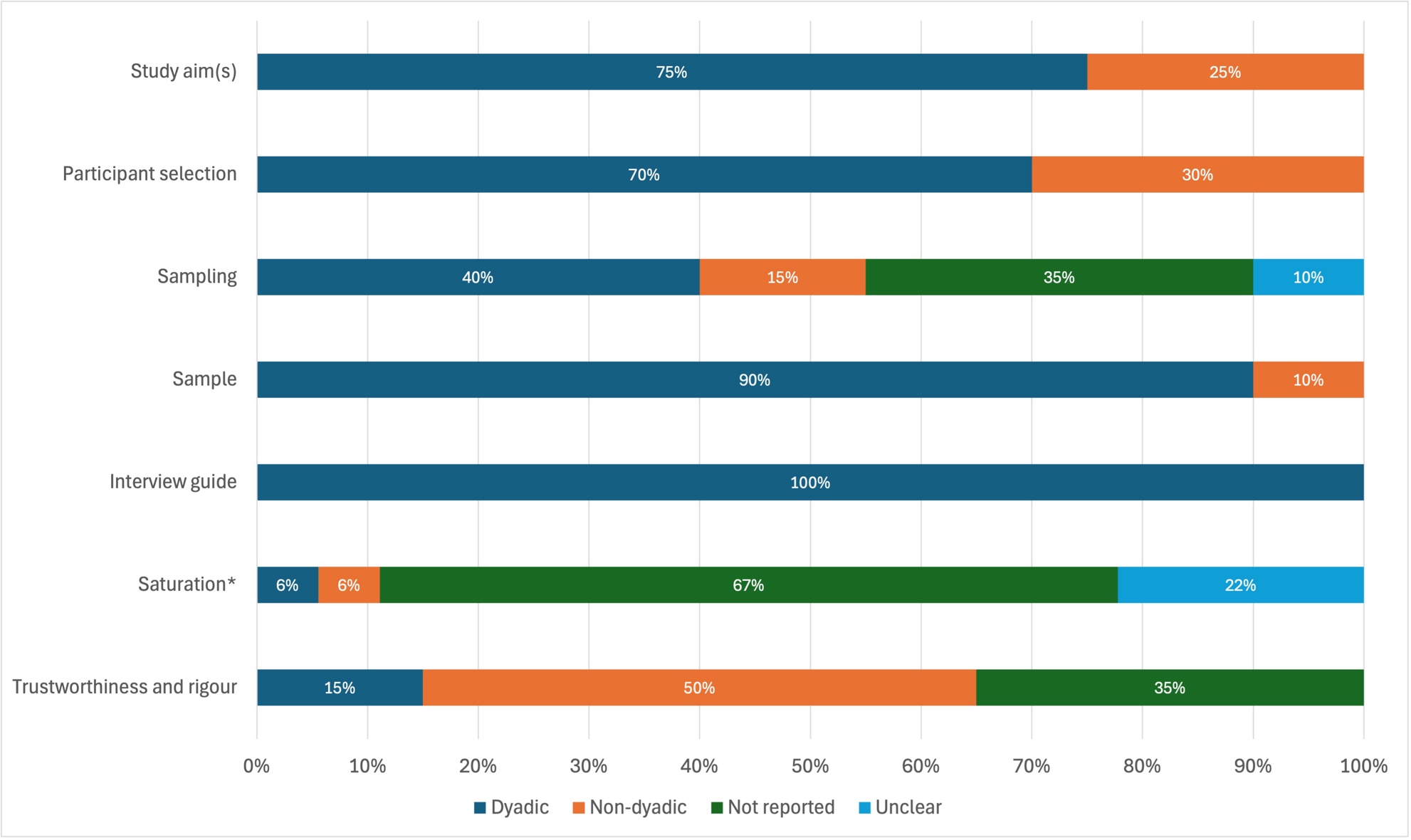


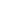


^*^n = 18 (excludes 2 secondary case studies)

Supplementary Figure 2. Dyadic conceptualization of different steps of research process for studies reporting a dyadic or multiperspective methodology (n = 20)
